# Studies on the selectivity of proline hydroxylases reveal new substrates including bicycles

**DOI:** 10.1016/j.bioorg.2019.103386

**Published:** 2020-01

**Authors:** Tristan J. Smart, Refaat B. Hamed, Timothy D.W. Claridge, Christopher J. Schofield

**Affiliations:** a Chemistry Research Laboratory, Department of Chemistry, University of Oxford, 12 Mansfield Road, Oxford OX1 3TA, United Kingdom; b School of Chemistry and Biosciences, Faculty of Life Sciences, University of Bradford, Richmond Rd, Bradford BD7 1DP, United Kingdom

**Keywords:** Biocatalysis, l-proline, Proline hydroxylase, 2-Oxoglutarate oxygenases, Amino acid oxidation

## Abstract

Studies on the substrate selectivity of recombinant ferrous-iron- and 2-oxoglutarate-dependent proline hydroxylases (PHs) reveal that they can catalyse the production of dihydroxylated 5-, 6-, and 7-membered ring products, and can accept bicyclic substrates. Ring-substituted substrate analogues (such hydroxylated and fluorinated prolines) are accepted in some cases. The results highlight the considerable, as yet largely untapped, potential for amino acid hydroxylases and other 2OG oxygenases in biocatalysis.

## Highlights

• Studies on proline hydroxylase selectivity reveals new products.
• Proline hydroxylases can produce dihydroxylated 5-, 6-, and 7-membered ring products.
• Proline hydroxylases can accept bicyclic substrates.
• Bicyclic products arise via bifurcation: two C-H bonds are accessible to the reactive oxidising species.
• The results have implications for other oxygenases, including those catalysing protein modifications.
• The results highlight the potential for amino acid hydroxylases in biocatalysis.

## Graphical abstract

**Figure:**
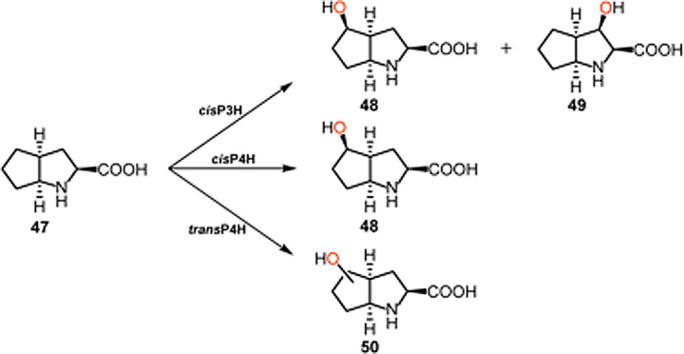

## 1 Introduction

Hydroxylated amino acids are common starting materials for the synthesis of pharmaceuticals and agrochemicals. They are also intermediates in the biosynthesis of natural products with interesting biomedicinal properties, including many antimicrobials [1], [2], [3]. Recent work has also expanded the set of identified hydroxylated protein residues, which, although not as large as that of hydroxylated amino acids, is extensive [1].

The ferrous iron- and 2-oxoglutarate (2OG)-dependent dioxygenases (ODDs) possibly catalyse the widest range of oxidations of amino acids and proteins of any identified enzyme family [1], [2], [3]. Prolyl hydroxylation of collagen was the first identified reaction catalysed by 2OG oxygenases and occurs to give C-3- and C-4-hydroxyprolyl protein products. Analogous prolyl hydroxylations of many collagen-like proteins have been identified [1]. *Trans*-C-4-prolyl hydroxylation of the hypoxia inducible transcription factor plays a key role in the adaptive response to hypoxia in animals [4]; related modifications have been identified in lower eukaryotes and prokaryotes [5], [6]. *Trans*-C-3-prolyl hydroxylation also occurs in a ribosomal protein (RPS23) in eukaryotes ranging from yeasts to humans [7], [8]. Interestingly, the yeast, but not the human, ribosomal prolyl hydroxylase catalyses a second hydroxylation of the same residue to give a dihydroxylated product [7].

In connection with the work on prolyl hydroxylation aimed at defining regio- and stereo-selectivities, we are interested in exploring the selectivity of proline (**1**) hydroxylases and using them to prepare standards for amino acid analyses, associated with the functional assignment of protein mono and di-hydroxylases. Aside from their use in preparing standards for structure validation, 2OG oxygenases have considerable potential as industrial scale biocatalysts, as demonstrated by the use of a recombinant l*-*proline (**1**) trans*-4-*hydroxylase (*trans P4H*)[9].

**Fig. 1:**
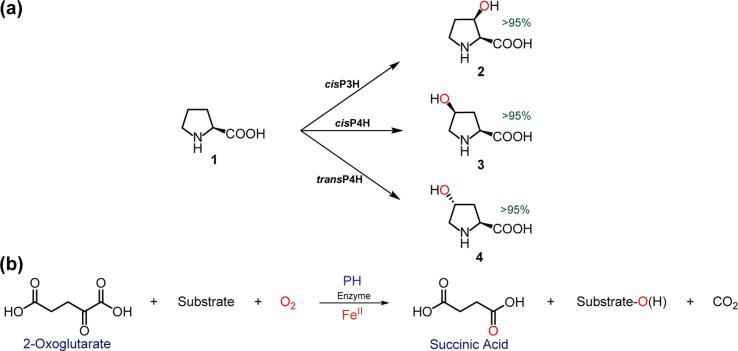
(a) Products of proline hydroxylase (PH) reactions using the natural substrate (l-proline, **1**) [(2*S*,3*S*)-*cis*-3-hydroxy**-**l-proline (**2**), (2*S*,4*S*)-*cis*-4-hydroxy**-**l-proline (**3**), and (2*S*,4*R*)-*trans*-4-hydroxy**-**l-proline (**4**)], and (b) the stoichiometry of the proline-hydroxylase reactions.

Hydroxyprolines and modified (e.g. by methylation) prolines are present in many peptide-based natural products [10], [11], [12]. In some cases, the proline-hydroxylations occur prior to peptide-synthetase-catalysed oligomerisation [10], [11], [12]. Following from pioneering labelling [10], [11] and purification studies [13], recombinant forms of proline-hydroxylases (PHs) have been produced (Fig. 1) [1], [14], [15], [16], [17], [18], [19], [20], [21], [22], [23], [24], [25], [26], [27]. Crystallography has revealed that the PHs contain a distorted, double-stranded, beta-helix (DSBH) fold, a His-X-(Asp/Glu)…His iron-binding motif, and a cosubstrate-binding mode, all of which are characteristic of a 2OG oxygenases [28], [29], [30]. Proline (**1**) hydroxylation occurs with retention of stereochemistry [31].

Several studies are reported on the selectivity of PHs – these reveal they can accept 3,4-dehyroproline to give epoxides [14], [20], [32], some methylated proline derivatives [23], [25], and azetidine- and piperidine C-2-carboxylates [20], [22], [26], [27], [32] to give hydroxylated products with different stereo- and regio-chemistries. Here, we report further studies on the substrate and product selectivities of three recombinant PHs: l-proline *cis*-3-hydroxylase type I (cisP3H) [15], [20], [33], l-proline *cis*-4-hydroxylase (cisP4H) [21], [26], [27], and l-proline *trans*-4-hydroxylase (*trans*P4H) [17], [18]. The results reveal PHs can catalyse the hydroxylation of substrates of varied ring sizes, substituted prolines, sizes, *N*-substituted prolines, including fluorinated and hydroxylated rings, and, importantly, bicyclic rings systems.

## 2 Results and discussion

### 2.1 Substrate analogue studies

Recombinant forms of the three bacterial PHs were produced in *Escherichia coli* using standard procedures (see Supplementary Information): cisP3H (from *Streptomyces spp*. [strain TH1]) [15], [20], [33], cisP4H (from *Sinorhizobium meliloti*) [21], [26], [27], and transP4H (from *Dactylosporangium* sp.) [17], [18]. We used LC/MS and ^1^H/^19^F NMR assays (^1^H: 500/700 MHz; ^19^F: 470 MHz) to monitor substrate turnover. Using the natural l-proline (**1**) substrate, the three recombinant PHs efficiently (>95% substrate conversion) produced hydroxylated products (**2**, **3**, and **4**) with the reported diastereoselectivities under standard conditions (Fig. 1) [15], [18], [20], [32], [33], as shown by LC/MS (using authentic standards) (Fig. 1). In these and subsequent reactions substrate hydroxylation was accompanied by turnover of 2OG to succinate. We then tested a range of substrate analogues for activity with the three PHs.

#### 2.1.1 Substrate analogue studies using substrates with different ring sizes

**Fig. 2:**
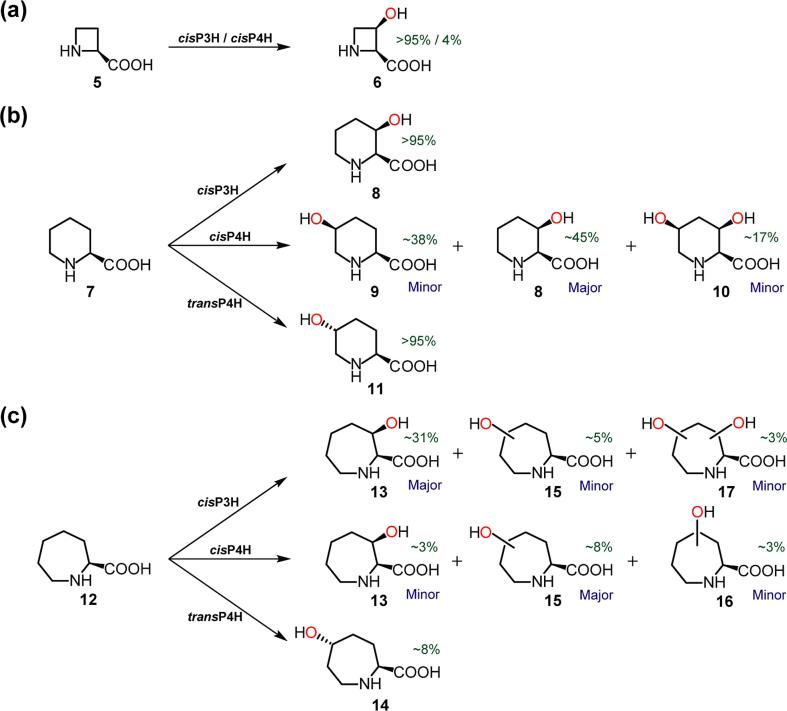
Products generated by proline hydroxylases using substrate analogues with different ring sizes (4-, 6- and 7-membered rings). In this and subsequent schemes all PH catalysed reactions are coupled to the oxidation of 2OG /O_2_ to succinate/CO_2._ Note the precise structures of some products are unassigned. See Supplementary Information for details of incubations, structure assignments and (approximate) yields (Table S7).

Consistent with literature reports, cisP3H and cisP4H catalyse the hydroxylation of (2S)-l-azetidine-2-carboxylic acid (**5**) to give (2*S*,3*R*)-*cis*-3-hydroxy-l-azetidine-2-carboxylic acid (**6**) (Fig. 2a) [14], [20], [26], [32]. We did not observe transP4H-catalysed hydroxylation of (2S)-l-azetidine-2-carboxylic acid (**5**). With (2S)-l*-*aziridine-2-carboxylic acid (Table S6 – Supporting Information), we did not observe hydroxylation to give a detectable product, though we cannot rule out oxidation to give an unstable product.

**Fig. 3:**
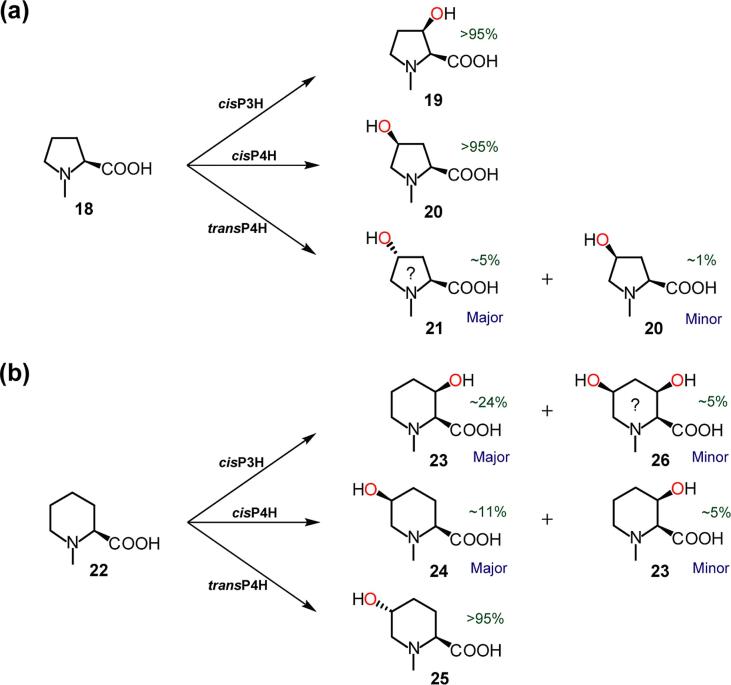
Products generated by proline hydroxylases using *N*-methylated substrates analogues: the products appear analogous to those produced with the unmethylated analogues (Fig. 1). See Supplementary Information for details of incubations, structure assignments, and (approximate) yields (Table S7). (? = provisionally assigned structure).

With (2*S*)-l-pipecolic acid (**7**), all 3 PHs generated singly hydroxylated products, as reported (Fig. 2b) [20], [22], [25], [26], [27], [32]. However, with *cis*P4H, in addition to the reported (2S,3S)-*cis*-3-hydroxy-l-pipecolic acid (**8**) and (2S,5S)-*cis*-5-hydroxy-l-pipecolic acid (**9**) products, we detected a dihydroxylated product (Fig. 2b), (2*S*,3*R*,5*S*)-3,5-dihydroxy-l-pipecolic acid (**10**). With *cis*P3H and *trans*P4H, (2S,3S)-*cis*-3-hydroxy-l-pipecolic acid (**8**) and (2*S*,5*S*)-*trans*-5-hydroxy-l-pipecolic acid (**11**) were observed (Fig. 2b), respectively. Interestingly, the 7-membered ring substrate analogue, (2*S*)-l-azepane-2-carboxylic acid (Azp) (**12**), was also hydroxylated, albeit in lower yields (~2.5–30%) (Fig. 2c). The 7-membered ring product observed in the highest yield was with *cis*P3H, *i.e.* (2*S*,3*R*)-*cis*-3-hydroxy-l-azepane-2-carboxylic acid (**13**), as measured by ^1^H NMR. LC/MS-analysis also implied that *cis*P3H reactions produced, albeit at relatively low levels, an additional mono-hydroxylated product, as well as a dihydroxylated product (**17**). With *cis*P4H, LC/MS detection showed that three hydroxylated products were generated, one of which had the same retention time as **13** (from the *cis*P3H reactions), suggesting this product was likely **13**; the other two *cis*P4H products (**15** and **16**) were likely C5/C4-hydroxylated species. Reactions catalysed by *trans*P4H produced a single product, which was characterised by NMR and assigned as (2*S*,5*R*)-*trans*-5-hydroxy-l-azepane-2-carboxylic acid (**14**). The observation of dihydroxylated products suggested that more complex substrates might be accepted by the PHs.

#### 2.1.2 Substrate analogue studies using *N*-functionalised substrates

The results indicate that substrates require a (2*S*)-amino acid to be accepted by the PHs, consistent with the literature [13]. Thus, we did not observe any hydroxylation of substrate analogues with the (2*R*)-stereochemistry, *N*-acyl groups, or carboxylic acid derivatives (e.g. esters/amides functionality) (see Supplementary Information). We did, however, detect PH-catalysed hydroxylation of *N*-methylated substrates, including (2*S*)-*N*-methyl-l-proline (*N*-Me-Pro) (**18**) and (2*S*)-*N*-methyl-l-pipecolic acid (*N*-Me-Pip) (**22**). LC/MS analysis of reactions with *cis*P3H and *cis*P4H manifested mono-hydroxylated products; NMR characterisation showed that these were (2*S*,3*R*)-*cis*-3-hydroxy-*N*-methyl-l-proline (**19**) and (2*S*,4*S*)-*cis*-4-hydroxy-*N*-methyl-l-proline (**20**), respectively (Fig. 3a). Interestingly, in the case of *trans*P4H, in addition to the major assigned (it was not isolated to high purity for definitive assignment by NMR) product (2*S*,4*R*)-*trans*-4-hydroxy-*N*-methyl-l-proline (**21**), LC/MS provided evidence for low-level production of a product with the same retention time as **20** (suggesting it was **20**) (Fig. 3a). With (2*S*)-*N*-methyl-l-pipecolic acid (**22**), the major assigned products (**23**, **24**, **25**) are analogous to those seen using a (2*S*)-pipecolic acid (**7**) substrate (Fig. 3b). In the case of *cis*P3H, LC/MS provided evidence for a dihydroxylated product, proposed to be (2*S*,3*R*,5*S*)-3,5-dihydroxy-*N*-methyl-l-pipecolic acid (**26**). Tested substrates with larger *N*-substitutions, such as (2*S*)-*N*-benzyl-l-proline (Table S6 – Supporting Information), were not accepted by the PHs.

#### 2.1.3 Substrate analogue studies using C-functionalised substrates

**Fig. 4:**
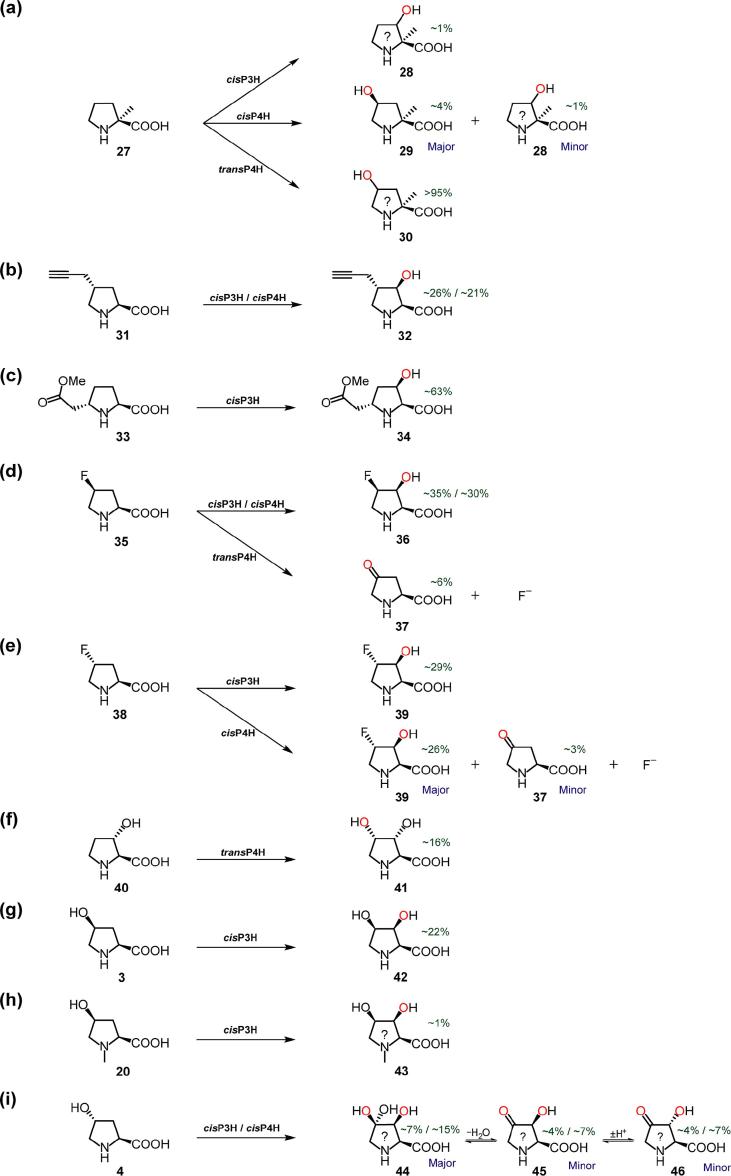
Products generated by proline hydroxylases using ring-substituted substrate analogues: note alkylated, hydroxylated, and fluorinated substrate analogues are substrates. See Supplementary Information for details of incubations, structure assignments, and (approximate) yields (Table S7). (? = provisionally assigned structure).

Recent studies have shown that *trans*-3-methyl-l-proline [23] and *cis*-3-hydroxy-l-proline [26] are PH substrates, suggesting that other ring-substituted proline analogues may be substrates. LC/MS analyses showed that (2*S*)-2-methyl-l-proline (2-Me-Pro) (**27**) was a substrate for all three PHs tested (Fig. 4a). LC/MS analysis showed that the *cis*P3H and *trans*P4H reactions produced singly hydroxylated products, likely (2*S*,3*R*)-*cis*-3-hydroxy-2-methyl-l-proline (**28**) and (2*S*,4*R*)-*trans*-4- hydroxy-2-methyl-l-proline (**30**), respectively.

LC/MS assays suggested that *cis*P4H produces two hydroxylation products: one with the same retention time as the *cis*P3H product (**28**) (suggesting it is the same) (‘minor’ product), and one assigned as (2*S*,4*S*)-*cis*-4-hydroxy-2-methyl-l-proline (**29**) (‘major’ product), by NMR (Fig. 4a). LC/MS-assays indicated that a substrate analogue with a larger C-2-functionality, *i.e.* (2*R*)-2-(4-methylbenzyl)-l-proline, was not hydroxylated, likely due to steric factors (Table S6 – Supporting Information).

LC/MS-analyses showed that a C-4-alkynylated substrate analogue, (2*S*,4*R*)-*trans*-4-(prop-2-yn-1-yl)-l-proline (**31**), was a substrate for *cis*P3H and *cis*P4H (but not *trans*P4H) (Fig. 4b); in both cases, a single-hydroxylation product was detected, assigned by NMR as (2*S*,3*R*,4*S*)-3-hydroxy-4-(prop-2-yn-1-yl)-l-proline (**32**) (Fig. 4b).

Proline analogues with C-5-/C-6-carboxyl functionalities were tested as PH substrates by LC/MS assays; (2*R*,5*S*)-5-carboxy-l-proline, (2*R*,5*S*)- 5-carboxy-*N*-methyl-l-proline, and (2*R*,6*S*)-6-carboxy-l-pipecolic acid were not PH substrates (Table S6 – Supporting Information). Substrate analogues with a C-5-carboxymethyl functionality were also tested as substrates, *i.e. cis*- and *trans*-5-(carboxymethyl)-l-proline (*c*-CMP and *t*-CMP); these compounds were also not PH-substrates (Table S6 – Supporting Information). However, the ‘carboxymethyl ester’ of *t*-CMP, *trans*-5-(2-methoxy-2-oxoethyl)-l-proline (‘Me-*t*-CMP’) (**33**), was found to be a *cis*P3H substrate by LC/MS assay; NMR characterisation showed that the product was (2*S*,3*R*,5*S*)-3-hydroxy-5-(2-methoxy-2-oxoethyl)-l-proline (**34**) (Fig. 4c). This result is interesting, because a CMP is an intermediate during the biosynthesis of the carbapenem antibiotics [34], [35], [36].

C-4-Fluorinated proline analogues were also assayed as PH substrates using LC/MS and NMR detection, *i.e. cis*-4-fluoro-l-proline (**35**) and *trans*-4-fluoro-l-proline (**38**). LC/MS and ^1^H/^19^F NMR data showed that **35** was a substrate of *cis*P3H and *cis*P4H (giving the same product), as well as *trans*P4H (Fig. 4d); NMR characterisation showed that the *cis*P3H product was (2*S*,3*S*,4*R*)-4-fluoro-3-hydroxy-l-proline (**36**); LC/MS and NMR analyses suggest the *trans*P4H product is a ketone, 4-oxo-l-proline (**37**). In the latter case, we propose that *trans*P4H hydroxylates the C-4-position of *cis*-4-F-Pro to produce an unstable intermediate, (2*S*,4*S*)-4-fluoro-4-hydroxy-l-proline, which fragments releasing fluoride. Analogous reactions have been observed previously with: (i) the human HIF prolyl hydroxylase, PHD2, which catalyses hydroxylation of a ‘*cis*-4-fluorinated-prolyl’ substrate analogue to produce a ‘4-keto-prolyl’ product [37]; and (ii) γ-butyrobetaine hydroxylase (BBOX), which hydroxylates (3S)-3-fluoro-γ-butyrobetaine (GBBF) to give 3-keto-γ-butyrobetaine following spontaneous fluoride release [38], [39]. LC/MS and ^1^H/^19^F NMR analysis showed that *trans*-4-F-l-proline (**38**) was a substrate for *cis*P3H and *cis*P4H; *cis*P3H catalyses production of one hydroxylation product, which was assigned by NMR as (2*S*,3*S*,4*S*)-4-fluoro-3-hydroxy-l-proline (**39**); *cis*P4H generates two products, *i.e.* (2*S*,3*S*,4*S*)-4-fluoro-3-hydroxy-l-proline (**39**) and 4-oxo-l-proline (**37**) (Fig. 4e). A synthetic sample of the ‘4-ketone’ product, 4-oxo-l-proline, was also tested as a PH substrate (with LC/MS and NMR assays), and was found to be inactive as a PH substrate.

Hydroxyprolines were also tested as PH substrates, initially assaying by LC-MS. In the case of (2*S*,3*S*)-*trans*-3-hydroxy-l-proline (**40**), *trans*P4H produced one product, assigned as (2*S*,3*R*,4*S*)-3,4-dihydroxy-l-proline (**41**) by NMR (Fig. 4f). In the case of *cis*-4-hydroxy-l-proline (**3**), *cis*P3H produced one hydroxylation product, which was assigned as (2*S*,3*S*,4*R*)-3,4-dihydroxy-l-proline (**42**) by NMR (Fig. 4g). LC/MS evidence suggested that, with (2*S*,4*S*)-*cis*-4-hydroxy-*N*-methyl-l-proline (**20**), *cis*P3H produces a single hydroxylation product, likely (2*S*,3*S*,4*R*)-3,4-dihydroxy-*N*-methyl-l-proline (**43**) (Fig. 4h). In the case of (2*S*,4*R*)-*trans*-4-hydroxy-l-proline (**4**), three products (**44**, **45**, and **46**) were detected (by LC/MS) for both *cis*P3H and *cis*P4H reactions (Fig. 4i). (Note that compared with the substrate, these products have mass increments of +32, +14, and +14 Da, respectively); one product is provisionally proposed to be a ‘C-3/C-4-dihydroxylated’ product, (2*S*,3*S*)-3,4,4′-trihydroxyproline (**44**) (*i.e.* the ‘hydrated’ form of a hydroxyketo species), and the other two products are proposed to be isomers of a C-3/C-4-hydroxyketo species (**45**, **46**).

#### 2.1.4 Substrate analogue studies using bicyclic substrates

**Fig. 5:**
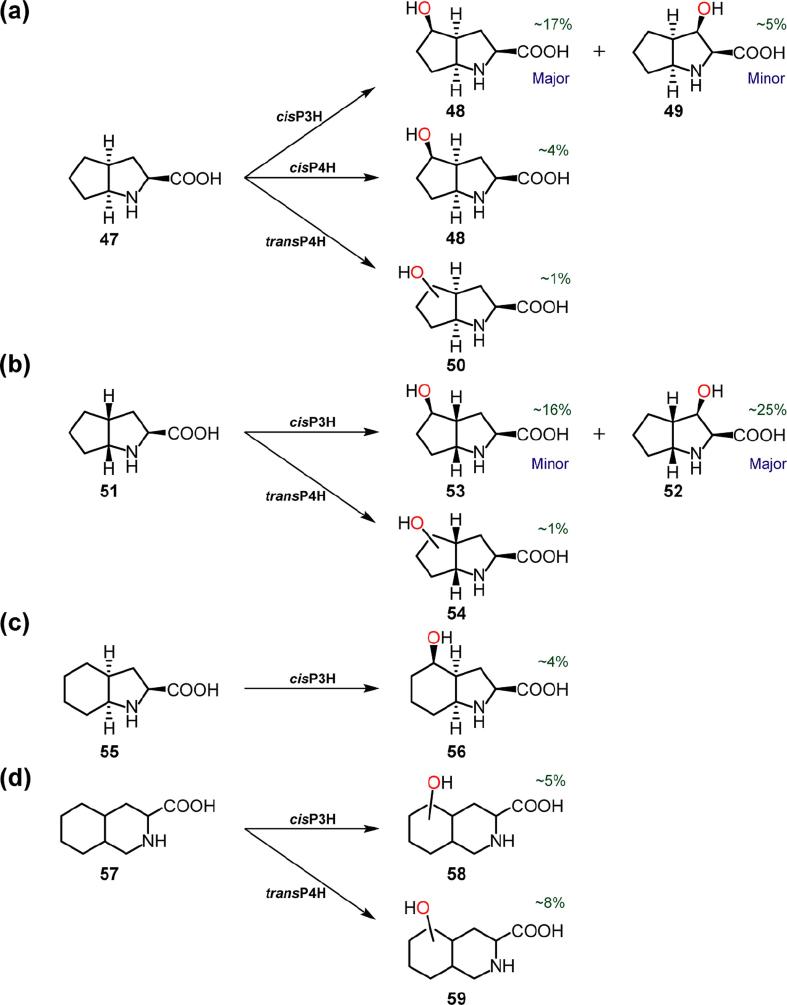
Products generated by proline hydroxylases using bicyclic substrate analogues. See Supplementary Information for details of incubations, structure assignments, and (approximate) yields (Table S7).

**Fig. 6:**
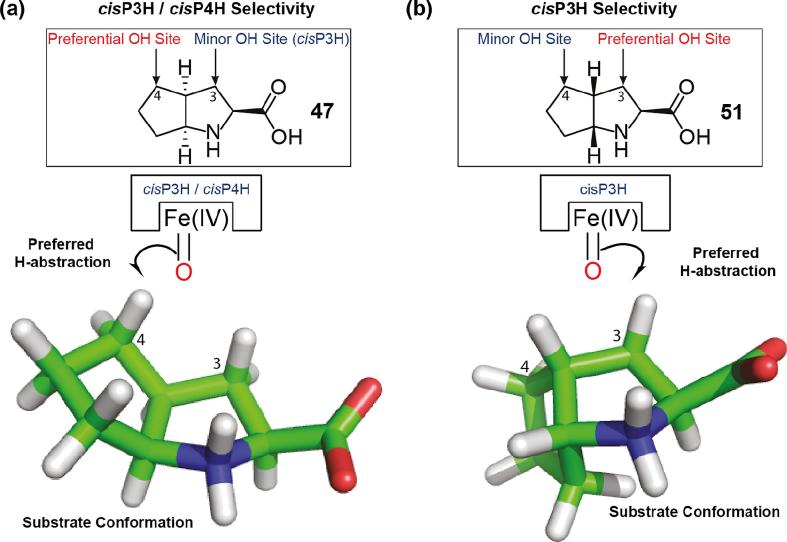
Proposed correlation between bicyclic ring confirmations and proline hydroxylase selectivity of reactions using **(a)** (2*S*,3a*S*,6a*S*)-octahydrocyclopenta[*b*]pyrrole-2-carboxylic acid (**47**) and **(b)** (2*S*,3a*R*,6a*R*)-octahydrocyclopenta[*b*]pyrrole-2-carboxylic acid (**51**).

The fact that *N*-methylated and carbon-backbone-functionalised substrates are accepted by the PHs suggested to us that bicyclic substrates may also be accepted by them. Whilst the bridged-ring systems of (1*R*,3*S*,4*S*)-2-azabicyclo[2.2.1]heptane-3-carboxylic acid (Table S6 – Supplementary Information) were not hydroxylated by the PHs, hydroxylation was observed with substrates containing two fused, 5-membered rings (fused at the C-4- and C-5-postions of proline (**1**)) (Fig. 5). With (2*S*,3a*S*,6a*S*)-octahydrocyclopenta[*b*]pyrrole-2-carboxylic acid (**47**), *cis*P4H and *trans*P4H produced different mono-hydroxylated products (by LC/MS) (**48** and **50**, respectively) (Fig. 5a). However, in the case of *cis*P3H, two hydroxylation products were detected (by LC/MS) (Fig. 5a); the major product (**48**) had the same retention time as the *cis*P4H product (suggesting they are the same). NMR characterisation showed that the hydroxylation sites were at the C-3 and C-4 positions of the starting material (**47**) (*i.e.* on the ‘proline’ and ‘cyclopentane’ rings, respectively); analysis showed that the major product was (2*S*,3a*S*,4*R*,6a*S*)-*cis*-4-hydroxy-octahydrocyclopenta[*b*]pyrrole-2-carboxylic acid (**48**), and the minor product was (2*S*,3*R*,3a*R*,6a*S*)-*cis*-3-hydroxy-octahydrocyclopenta[*b*]pyrrole-2-carboxylic acid (**49**). **48** represents the first report of a PH-catalysed hydroxylation of a ‘non-proline’-type ring position. Interestingly, this product is favoured over the ‘*cis*-3-hydroxy’ product analogue (**49**), which usually predominates in *cis*P3H reactions (Fig. 1a). The regioselectivity of this reaction may reflect the ‘butterfly-like’ conformation of this substrate (**47**) (Fig. 6), resulting in the cyclopentane ring being closer to the ferryl intermediate with *cis*P3H and *cis*P4H than the ‘proline’ ring; hence, the C-4 position is preferentially hydroxylated over the C-3 position.

The ring-junction stereoisomer of the (2*S*,3a*S*,6a*S*)-octahydrocyclopenta[*b*]pyrrole-2-carboxylic (**47**) acid substrate, *i.e.* (2*S*,3a*R*,6a*R*)-octahydrocyclopenta[*b*]pyrrole-2-carboxylic acid (**51**), was also a PH substrate by LC/MS assays (Fig. 5b); two hydroxylation products were detected for *cis*P3H (**52**, **53**), and one for *trans*P4H (**54**). NMR characterisation assigned the *cis*P3H products as (2*S*,3*R*,3a*S*,6a*R*)-*cis*-3-hydroxy-octahydrocyclopenta[*b*]pyrrole-2-carboxylic acid (**52**) (major) and (2*S*,3a*R*,4*R*,6a*R*)-*cis*-4-hydroxy-octahydrocyclopenta[*b*]pyrrole-2-carboxylic acid (**53**) (minor) (Fig. 5b). Thus, in this case, the anticipated, ‘*cis*-3-hydroxy’ product predominates, likely in part because the fused cyclopentane ring is ‘*trans*’ to the carboxylic acid group and is directed away from the ferryl intermediate with *cis*P3H, making the ‘natural substrate’ C-3 proton the closest available for abstraction (Fig. 6).

*cis*P3H also catalyses hydroxylation of (2*S*,3a*S*,7a*S*)-octahydro-1H-indole-2-carboxylic acid (**55**), which has fused 5- and 6-membered rings (Fig. 5c). In a manner analogous to the (2*S*,3a*R*,6a*R*)-octahydrocyclopenta[*b*]pyrrole-2-carboxylic acid (**51**) reaction, *cis*P3H catalysed hydroxylation of the cyclohexane ring of **55** to give the ‘*cis*-4-hydroxy’ product, (2*S*,3a*S*,4*R*,7a*S*)-*cis*-4-hydroxy-octahydro-1H-indole-2-carboxylic acid (**56**) (by NMR). A compound containing two fused, 6-membered rings, decan-hydroisoquinoline-3-carboxylic acid (**57**), was a substrate of *cis*P3H and *trans*P4H (by LC/MS); two hydroxylation products were detected for *cis*P3H and one for *trans*P4H (we were unable to assign the stereochemistry of these by NMR) (Fig. 5d).

#### 2.1.5 Substrate analogue studies using unsaturated substrates

**Fig. 7:**
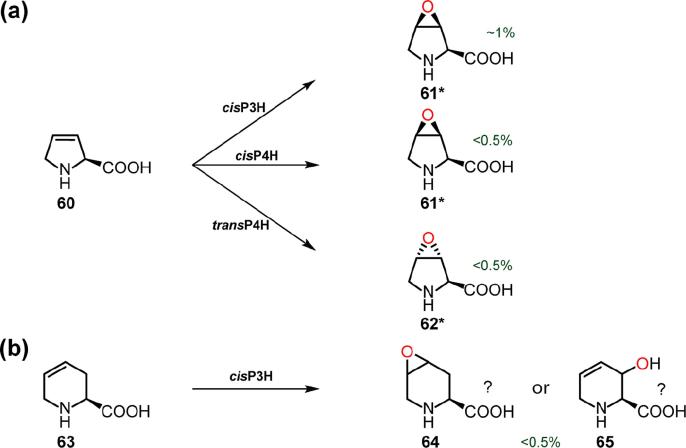
Products generated by proline hydroxylases using unsaturated compounds. See Supplementary Information for details of incubations, structure assignments, and yields (Table S7).

Unsaturated proline analogues were then tested as proline hydroxylase substrates. (2*S*)-3,4-dehydro-l-proline (**60**) was a substrate of *cis*P3H, *cis*P4H, and *trans*P4H by LC/MS analysis (Fig. 7). Consistent with previous studies, *cis*P3H and *cis*P4H produced (2*S*,3*S*,4*R*)-*cis*-3,4-epoxy-l-proline (**61**) [20], [26], [32], and *trans*P4H produced (2*S*,3*R*,4S)-*trans*-3,4-epoxy-l-proline (**62**) [14], [32] (Fig. 7a). (2*S*)-4,5-dehydro-l-pipecolic acid (**63**), was also a hydroxylation substrate of *cis*P3H by LC/MS analysis (Fig. 7b). However, because the product of this reaction was observed to coelute with the starting material (**63**) during purification, and NMR characterisation for the product was inconclusive, it is unclear whether an ‘epoxide’ was formed [*i.e.* (2*S*,4*S*,5*R*)-*cis*-4,5-epoxy-l-pipecolic acid (**64**)], or an allylic alcohol [e.g. (2*S*,3*S*)-*cis*-3-hydroxy-4,5-dehydro-l-pipecolic acid (**65**)].

## 3 Conclusions

2OG dependent amino acid hydroxylases have considerable value as industrial catalysts [9]. Our results substantially expand the biocatalytic potential of pH catalysed hydroxylations, including with the demonstration that certain *N*-alkylated amino acids and bicyclic ring systems are accepted as PH substrates. The products produced, including di- and tri-hydroxylated prolines should be useful in ongoing work concerning functional assignment studies on 2OG dependent protein hydroxylases [6], [7], [8].

The observation that substrate analogues with four, six, and seven membered ring containing substrate analogues undergo PH catalysed oxidations, with dihydroxylated products being produced in some cases, is of interest from a biocatalytic perspective (Fig. 4 and 5). Some substituted prolines, including *N*-methylated prolines, were also found to be PH substrates giving triply functionalised pyrrolidine rings (Fig. 4). However, *N*-acylated or (2*R*) – amino acids were not PH substrates (Table S6).

The observation of two products with some bicyclic substrate/PH combinations is mechanistically interesting, suggesting that the ferryl intermediate in 2OG-dependent PH catalysis [1] may be positioned approximately equidistant between different potentially oxidised C—H bonds (Fig. 6). However, a mechanism involving hydrogen abstraction at one site, followed by hydrogen atom transfer and hydroxylation at a second site cannot be ruled out. There is a possibility of such processes occurring in 2OG oxygenase catalysis in carbapenem antibiotic biosynthesis [34]. It is possible that such processes occur in 2OG oxygenase catalysis involving protein/nucleic oxidations.

2OG oxygenases have been shown [1], [2], [3] to be amenable to structure based protein engineering to produce enzymes with improved product selectivities with unnatural substrates [1], [9]. Although this work is at an early stage with PHs, it has been reported that a directed-evolution approach has led to a modified *cis*P4H (with 3 substitutions) with improved activity with respect to hydroxylation of l-pipecolic acid to give (2*S*,5*S*)-5-hydroxy-l-pipecolic acid [27]. The relaxed substrate/product selectivities of PHs, and some, but not all, other 2OG oxygenases (including some catalysing protein modifications, *e.g*. factor inhibiting HIF) suggests that they may be suitable for ‘late stage modifications’ of valuable chemicals; following the initial identification of a desired product, its oxygenase catalysed production may be optimised by protein engineering. There would, thus, appear to be considerable, as yet largely unexplored, potential for (engineered) PHs, and other 2OG oxygenases, in biocatalysis.

## 4 Materials and methods

DNA encoding for Streptomyces sp. (strain TH1) *cis*P3H type I, *Sinorhizobium meliloti cis*P4H, and *Dactylosporangium* sp. *trans*P4H (with 5′-*Nde*I and 5′-*Hin*dIII sites) was synthesised by Life Technologies’ GeneART, and codon-optimised for *E. coli* expression. The PH genes were then subcloned into a Takara pCOLD^TM^ I expression vector to produce *N*-terminally His_6_-tagged PHs. Sequence-verified clones (by The Gene Service, Source BioScience, Oxford, UK) were transformed into Stratagene BL21-Gold (DE3) competent cells for protein production. Reagents, casting equipment, and electrophoresis tanks for SDS-PAGE were obtained from Bio-Rab Laboratories; molecular-mass markers (Thermo PageRuler Plus) were from Thermo Scientific. Reagents for PH reactions were from Sigma-Aldrich and Fluka. Full details are given in the Supplementary Information.

## Declaration of Competing Interest

There is no conflict of interest.
