# Supplementary material for: Studies on the selectivity of proline hydroxylases reveal new substrates including bicycles
Source: Bioorg Chem. 2020 Jan;94:103386. doi: 10.1016/j.bioorg.2019.103386 (PMC6958525; doi:10.1016/j.bioorg.2019.103386)
Supplement: Supplementary Data 1 [file mmc1.docx]

**Studies on the Selectivity of Proline Hydroxylases Reveal New**

**Substrates Including Bicycles**

Tristan J. Smart (ORCID number 0000-0002-6184-1618) ^a^, Refaat B. Hamed (ORCID number 0000-0002-9160-9414) ^a,b,^, Timothy D. W. Claridge (ORCID number 0000-0001-5583-6460) ^a^, Christopher J. Schofield (ORCID number 0000-0002-0290-6565) ^a^*

1. *Chemistry Research Laboratory, Department of Chemistry, University of Oxford, 12 Mansfield Road, Oxford, OX1 3TA, United Kingdom.*
2. *School of Chemistry and Biosciences, Faculty of Life Sciences, University of Bradford, Richmond Rd, Bradford, BD7 1DP, United Kingdom*

Supporting Information

**Contents**

[Supporting Information 1](#_Toc20899314)

[1. Materials and Methods 2](#_Toc20899315)

[1.1. Chemicals and Consumables 2](#_Toc20899316)

[1.2. NMR Spectroscopy 2](#_Toc20899317)

[1.3. Cloning and Molecular Biology 3](#_Toc20899318)

[1.4. Proline Hydroxylase Production 3](#_Toc20899319)

[1.4.1. Cell Growth for Large-scale Production of *cis*P3H Type I 3](#_Toc20899320)

[1.4.2. FPLC Purification of *cis*P3H Type I 4](#_Toc20899321)

[1.4.3. Cell Growth for Large-Scale Production of *cis*P4H and *trans*P4H 7](#_Toc20899322)

[1.4.4. Purification of *cis*P4H and *trans*P4H 7](#_Toc20899323)

[1.4.5. LC/MS and NMR Proline Hydroxylase Assays 10](#_Toc20899324)

[1.4.5.1. LC/MS Assays 10](#_Toc20899325)

[1.4.5.2. Preparative-scale PH Reactions: LC/MS-purification Methods 11](#_Toc20899326)

[1.4.5.3. End-point NMR Assays 13](#_Toc20899327)

[2. Assignment of Reported Enzyme-catalysed Products 14](#_Toc20899328)

[2.1. Substrate Turnover Information 15](#_Toc20899329)

[2.2. Substrate Analogues with Different Ring Sizes 17](#_Toc20899336)

[2.3. *N*-Methylated Substrate Analogues 35](#_Toc20899337)

[2.4. Ring-substituted Substrate Analogues 49](#_Toc20899338)

[2.5. Bicyclic Substrate Analogues 79](#_Toc20899339)

[2.6. Unsaturated Substrate Analogues 93](#_Toc20899340)

[References 96](#_Toc20899341)

# Materials and Methods

## Chemicals and Consumables

Chemicals were from Sigma-Aldrich (Dorset, UK), Acros chemicals (Loughborough, UK), or Bachem (St. Helens, Merseyside, UK), except where stated and were used without further purification. HPLC grade solvents were from Rathburn (Walkerburn, UK) and used for chemical transformations, work-up, and chromatography without further purification. IPTG was from Melford Laboratories Ltd., electrophoresis grade agarose was from Bioline, and acrylamide/bis-acrylamide stock solution was from Sigma-Aldrich. Bacto Tryptone, Yeast Extract and Bacto Agar were from Oxoid and Difco. Plasmids and molecular biology enzymes were from Promega, Novagen, New England BioLabs, or Stratagen. Molecular weight markers for SDS-PAGE (prestained protein markers) were from Invitrogen. FPLC columns and equipment, and small-scale gel filtration columns (PD-10) were from Amersham Biosciences. Spin concentrators for protein concentration were from Amicon. Hilgenberg NMR tubes (1 mm or 2 mm diameter) were from CortecNet (Voisins-Le-Bretonneux, France). Deuterated solvents were from Sigma and Apollo Scientific Ltd.

Water was purified by a Millipore Milli-Q system fitted with a 0.22 µm filter at the outlet. All solutions used in molecular biology and microbiology were prepared according to standard procedures [[1](#_ENREF_1)] using Milli-Q water and were autoclaved or sterilised by filtration, as required.

‘Turnover yields’ (%) were calculated using an LC/MS internal standard (0.25 mM), *para*-aminosalicylic acid (*p*ASA) as reported [[2-6](#_ENREF_2)]. Other yields refer to purified, freeze-dried, and spectroscopically characterized compounds (except where stated).

## NMR Spectroscopy

All NMR spectra were recorded using Bruker AVIII 700 MHz machine (equipped with a ^1^H inverse TCI cryoprobe optimised for ^1^H-observation). Quenched assays were transferred to 5 mL tubes and analysed using a 500 MHz (^1^H)/470.4 MHz (^19^F) Bruker AVII 500 NMR spectrometer (equipped with a 5 mm TFI-^1^H/^19^F(^13^C) probe). Chemical shifts (δ) are given in ppm, relative to the solvent peak (HDO: δ_H_ = 4.701 ppm) or TSP-*d*_4_ (δ_H_ = 0.00 ppm). Instrument control and spectral processing was achieved using Bruker TopSpin 2.1 or TopSpin 3.0.

## Cloning and Molecular Biology

Molecular biology transformations were performed using autoclaved tips and sterile/nuclease-free Eppendorf tubes. Qiagen nuclease-free water was used as a reaction solvent for all molecular biology experiments. DNA encoding for full-length L-proline cis-3-hydroxylase type I (cisP3H) (from Streptomyces sp. [strain TH1]) [[7-9](#_ENREF_7)] was subcloned into the Novagen pET-24a(+) *E. coli* expression vector [[10](#_ENREF_10)]. Full-length constructs coding for *Sinorhizobium meliloti cis*P4H and *Dactylosporangium sp.* (strain RH1) *trans*P4H were designed with appropriate 5’- and 3’-restriction cloning sites (*NdeI* and *BamH*, respectively); the *E. coli* codon-optimised genes were then commercially synthesised by the GeneArt Gene Synthesis service (Life Technologies, Inc.). DNA encoding for *cis*P4H and *trans*P4H were subcloned into the Takara pCOLD I vector for production of recombinant *N*-terminally His_6_-tagged *cis*P4H and *trans*P4H; using combined NdeI and BamHI-HF restriction endonucleases, the PH genes were excised from the pMk-T plasmids, gel purified, and then ligated into the CIAP-dephosphorylated, linear Takara pCOLD I vector DNA. Analytical NdeI/BamH-HF restriction digests of plasmids (isolated from ligation-colony bacteria) revealed that the *cis*P4H and *trans*P4H genes were successfully subcloned the pCOLD I vector; all clones were also sequence verified by commercial sequencing (**Source BioScience**).

## Proline Hydroxylase Production

### Cell Growth for Large-scale Production of cisP3H Type I

4 L TB media (100 μg/mL kanamycin) cultures were inoculated with 10 mL of overnight 100 mL 2YT broth starter culture and incubated at 37 ºC with 220 rpm shaking until the OD_600_ reached approx. 0.65. Expression was induced with 0.5 mM IPTG; cultures were then incubated at 20 ºC for 16 h for cisP3H Type I [pET-24a(+)]. Cells were harvested using 1 L centrifuge tubes and a Beckman Coulter^TM^ Avanti J25 centrifuge with a JLA 9.1000 rotor spinning at 7,500 rpm (~8411*g* average) for 7 min at 3 ºC. The cell pellet was frozen at -80 ºC.

### FPLC Purification of cisP3H Type I

The pellet from a 4L growth was resuspended in 150 mL FPLC binding buffer containing 0.5 mM tris(2-carboxyethyl)phosphine (TCEP) and EDTA-free Protease Inhibitor Cocktail Tablets (Roche) at 4 ºC. Cells were lysed on ice by sonication using a Sonics Vibra Cell machine, applying ten 30 second bursts interrupted by 30 second pauses for (10 min total). The crude lysates were centrifuged (Beckman Coulter Avanti J25 centrifuge, JLA-25.50 rotor, 23,000 rpm [~43,400*g*], 20 min, 4 ºC), then treated with 5% (w/v) PEI solution dropwise on ice (4 ºC until the concentration reached 0.01% (w/v) (to precipitate DNA and nucleoproteins). The lysates were then clarified by centrifugation (~43,400*g* for 20 min at 4 ºC); the supernatant was then filtered through 0.22 μm filters using a 10 mL syringe.

A two-column, FPLC-purification method was then used: (i) ‘weak’ anion-exchange chromatography (DEAE Sepharose), followed by (ii) size-exclusion chromatography (Superdex 75). The lysate was loaded (6 mL/min) onto a 30 mL DEAE Sepharose FF column [Fig. S1 (a)]; the column was washed with binding buffer (A) until the UV-absorbance reached baseline [Fig. S1(a)]; the column was then washed with a mixture containing 45% (v/v) elution buffer (B) and 65% binding buffer (A) (225 mM NaCl) (until the UV-absorbance was reduced back to baseline) [Fig. S1 (a)]. *cis*P3H Type I was then eluted using a linear gradient of elution buffer from 45% to 100% over 2 and 1/3 column volumes (roughly 80 mL) [Fig. S1 (a) and (b)]; 5 mL fractions were collected. The purity of protein was assessed using SDS-PAGE [15% (w/v) acrylamide] [Fig. S1 (c)]; fractions containing highly purified *cis*P3H were pooled and concentrated using a 10 kDa-cut-off centrifugal concentrator (Amicon). The *cis*P3H fractions (~1 mL) were injected into a 2 mL sample loop and purified using a Pharmacia (320 mL) HiLoad 26/60 Superdex 75 column (Fig. S2). Highly purified fractions were concentrated (to ~51 mg/mL) using a 10 kDa-cut-off centrifugal concentrator; the *cis*P3H solution was then aliquoted (~15 μL) and frozen using liquid N_2_.


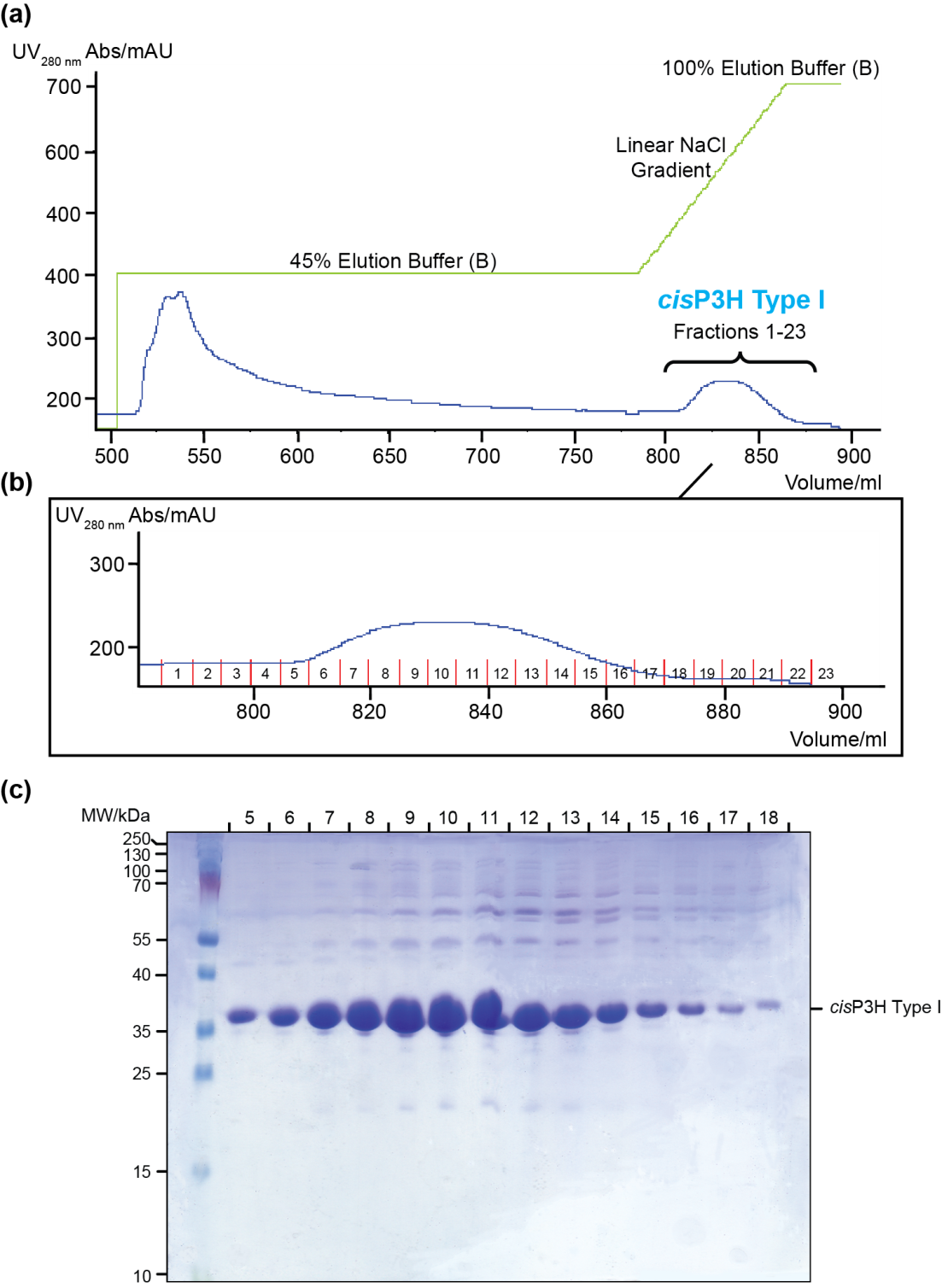


Fig. S1. Anion-exchange-chromatography purification of *cis*P3H Type I.

(a) and (b) UV_280 nm_-absorbance FPLC chromatogram for the *cis*P3H Type I AEC.

(c) Fractions were analysed by SDS-PAGE [15% (w/v) acrylamide].

Column: 30 mL Pharmacia DEAE Sepharose Fast Flow.

Buffer A: 50 mM MES-NaOH, 0.2 mM EDTA, 20% (v/v) glycerol, 0.1% (w/v) Tween 20, 0.5 mM TCEP, pH 6.0. Buffer B: 50 mM MES-NaOH, 500 mM NaCl, 0.2 mM EDTA, 10% (v/v) glycerol, pH 6.0.


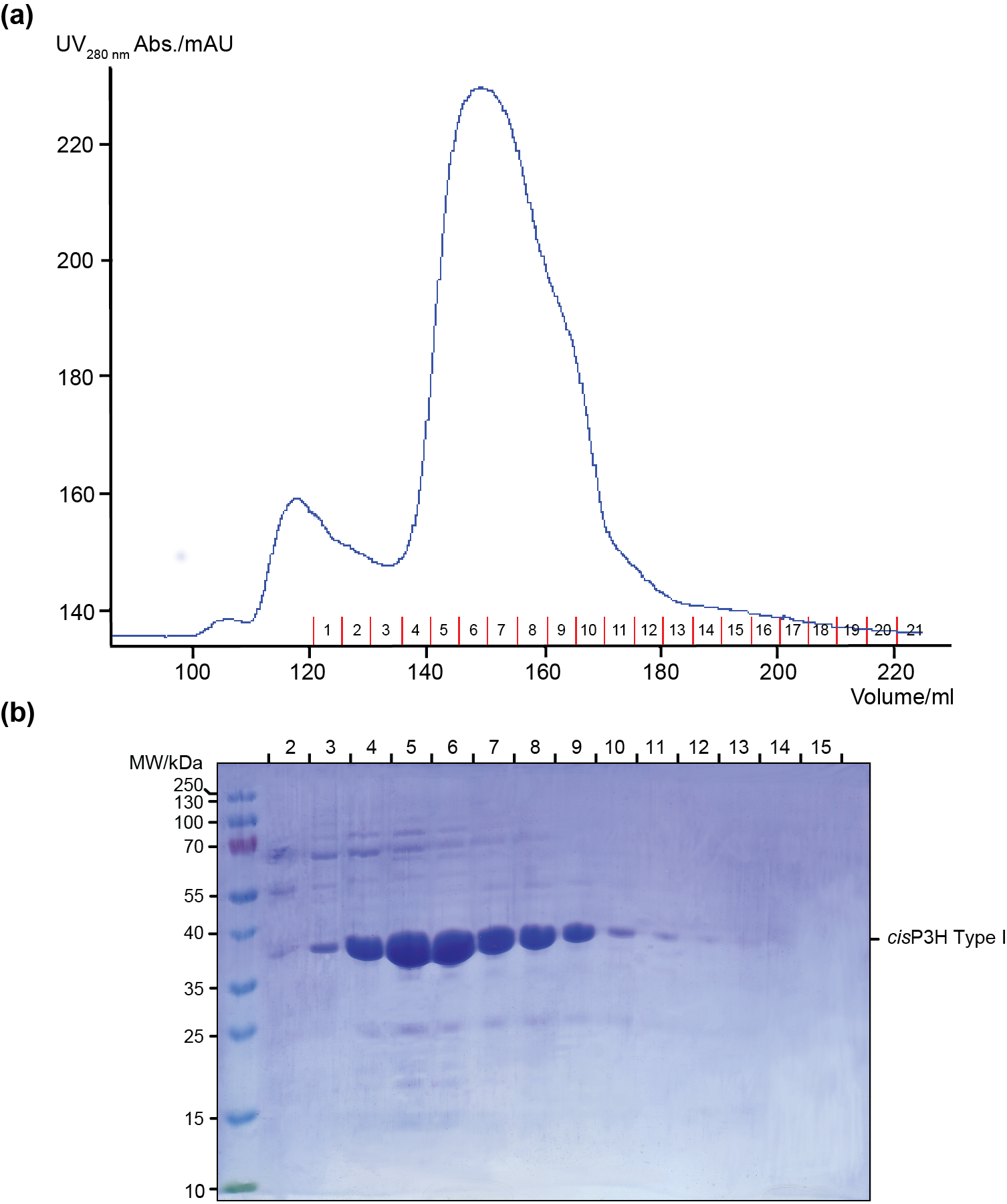


Fig. S2. Size-exclusion chromatography of *cis*P3H Type I.

(a) UV_280 nm_-absorbance FPLC chromatogram for the *cis*P3H Type I gel filtration fractions.

(b) Fractions were analysed by SDS-PAGE [12.5% (w/v) acrylamide].

Column: Pharmacia (320 mL) HiLoad 26/60 Superdex 75.

Buffer: 10 mM Tris-HCl, 150 mM NaCl, pH 7.5.

Buffer: 150 mM Tris-HCl, pH 7.5.

### Cell Growth for Large-Scale Production of cisP4H and transP4H

The cell growth methods for recombinant *cis*P4H and *trans*P4H production were the same as for *cis*P3H Type I, except that cultures were grown with 100 μg/mL ampicillin (sodium salt) (instead of kanamycin sulfate), and expression was induced at 15 ºC with 0.5 mM isopropyl β-D-1-thiogalactopyranoside (IPTG); after 24 h of incubation, the induced cell were harvested by centrifugation.

### Purification of cisP4H and transP4H

A 5 mL GE Healthcare HisTrap FF chromatography column was ‘charged’ with 20 mL of 50 mM NiSO_4_, washed with 10 mL of MilliQ distilled water, then with 50 mL of binding buffer prior to loading the cell lysate at 1.5 mL/min (230 mL). The column was then washed with binding buffer until the UV absorbance reached the baseline, then with 60 mL wash buffer. The recombinant enzyme was eluted with a linear gradient of elution buffer (B) (0-100% over 100 mL - 30mM to 400mM imidazole) [Fig. S3 (a) and Fig. S4 (a)]. Fractions containing purified protein [Fig. S3 (b) and Fig. S4 (b)] were concentrated and buffer exchanged (into 10 mM Tris-HCl, 150 mM NaCl, pH 7.5 buffer) by centrifuging Millipore Amicon Ultracell-10k concentrators [using a Beckmann Allegra 21R centrifuge equipped with a S4180 rotor, rotating at 4000 rpm (~1800*g*) at 4 ºC].


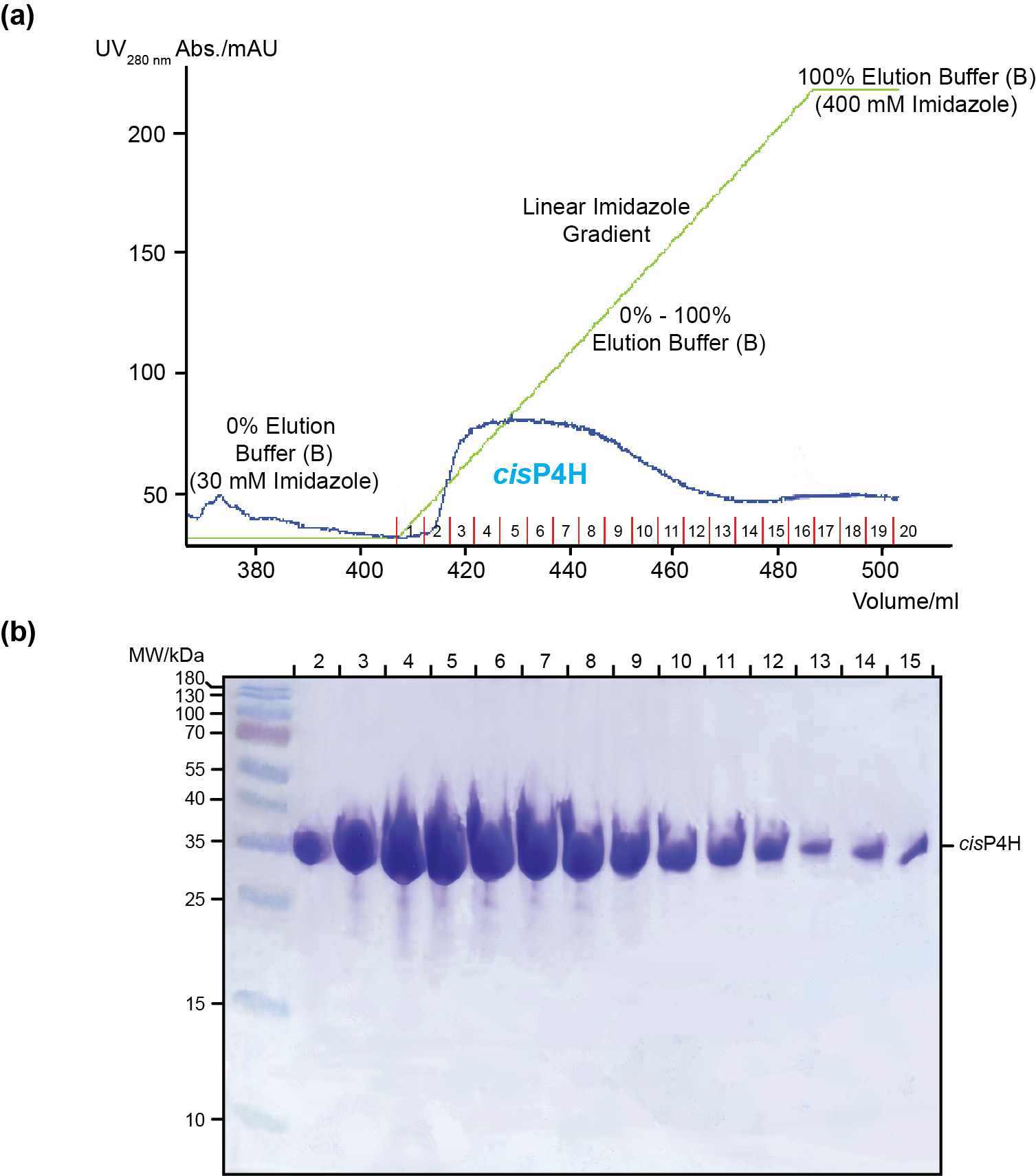


Fig. S3. Immobilised-nickel-affinity chromatography of *cis*P4H.

After the cell lysates had been loaded onto the column, the column was elated with excess binding buffer, then wash buffer until the UV_280nm_-absorbance trace reached baseline levels.

(a) The UV_280 nm_-absorbance FPLC chromatogram for the *cis*P4H; a linear gradient of increasing imidazole (30 mM-400 mM) to elute the *N*-His_6_-*cis*P4H, which manifested as a broad peak in the UV_280nm_-absorbance chromatogram.

(b) Tris-glycine SDS-PAGE [15% (w/v) acrylamide] analysis.

Column: 5 mL HisTrap FF.

Binding Buffer: 50 mM HEPES-NaOH, 500 mM NaCl, 5 mM Imidazole, 20% (v/v) glycerol, 0.1% Tween-20, 0.5 mM TCEP, pH 7.5.

Wash Buffer: 50 mM HEPES-NaOH, 500 mM NaCl, 30 mM Imidazole, 10% (v/v) glycerol, pH 7.5.

Elution Buffer (B): 50 mM HEPES-NaOH, 500 mM NaCl, 400 mM Imidazole, 10% (v/v) glycerol, pH 7.5.


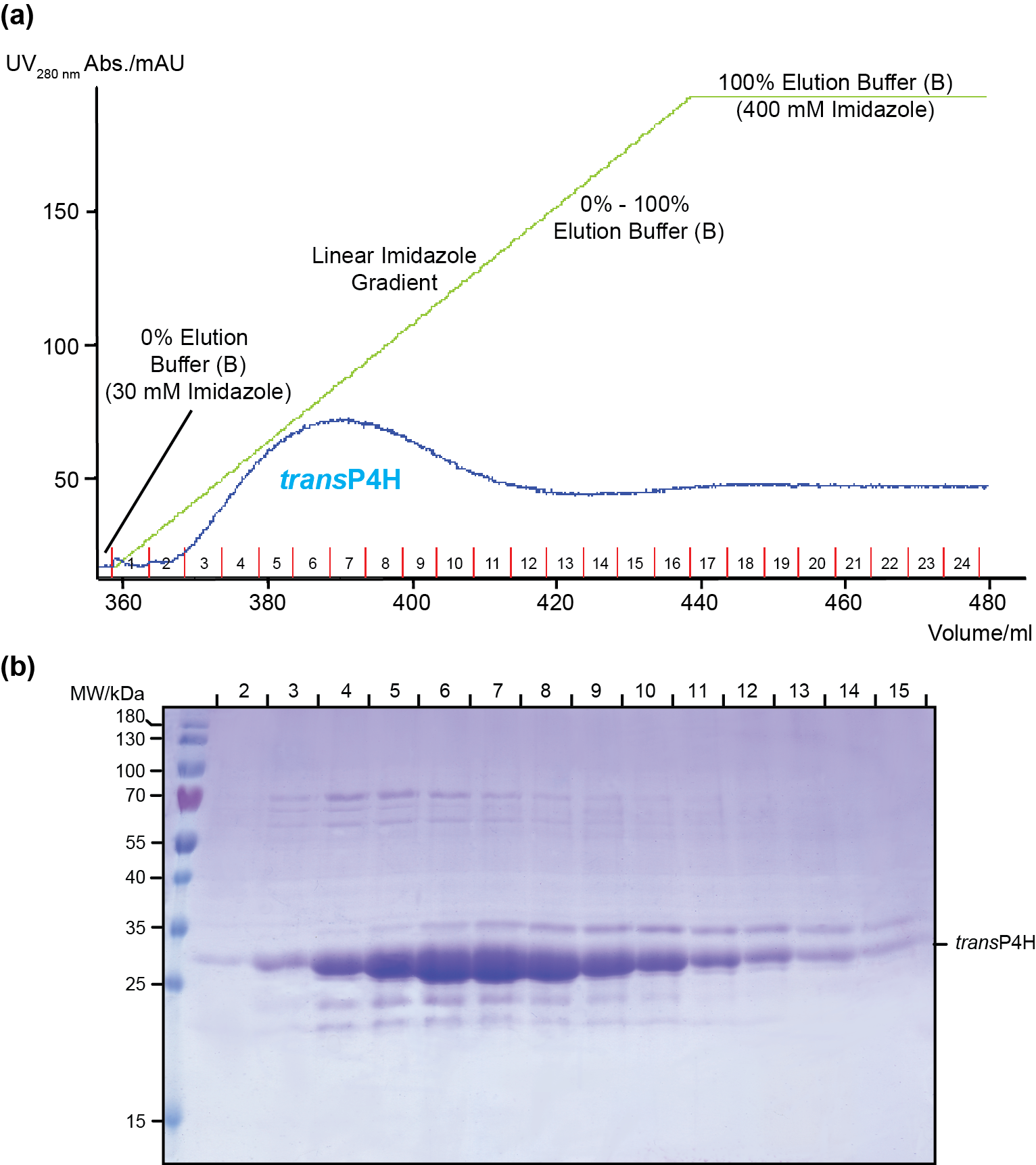


Fig. S4. Immobilised-nickel-affinity chromatography of *trans*P4H.

After the cell lysate had been loaded onto the column, the column was elated with excess binding buffer, then with wash buffer until the UV_280nm_-absorbance trace reached the baseline.

(a) UV_280 nm_-absorbance FPLC chromatogram for *cis*P4H; a linear gradient of increasing imidazole (30 mM-400 mM) to elute the *N*-His_6_-*trans*P4H.

(b) SDS-PAGE [15% (w/v) acrylamide] analysis.

Column: 5 mL HisTrap FF.

Binding Buffer: 50 mM HEPES-NaOH, 500 mM NaCl, 5 mM Imidazole, 20% (v/v) glycerol, 0.1% Tween-20, 0.5 mM TCEP, pH 7.5.

Wash Buffer: 50 mM HEPES-NaOH, 500 mM NaCl, 30 mM Imidazole, 10% (v/v) glycerol, pH 7.5.

Elution Buffer: 50 mM HEPES-NaOH, 500 mM NaCl, 400 mM Imidazole, 10% (v/v) glycerol, pH 7.5.

### LC/MS and NMR Proline Hydroxylase Assays

### LC/MS Assays

Analytical scale proline hydroxylase (PH) incubations were performed by sequential addition of the reagents in Table S1 to a 1.5 mL Eppendorf tube (100 μL final volume):

| **Reaction Component** | **Stock Concentrations** | **Final Concentration** | **Volume/μL** |
| --- | --- | --- | --- |
| 50 mM MES-NaOH, pH 6.5 | 50.0 mM | - | (76.0/77.0) / (75.0/76.0) / (76.3/77.3) μL |
| Sodium Ascorbate | 100 mM | 1.0 mM | 1.0 μL |
| (NH_4_)_2_Fe(SO_4_)_2_ | 5 mM*^a^* | 0.5 mM | 10.0 μL |
| 2-Oxoglutarate (2- OG) | 100 mM | 10.0 mM | 10.0 μL |
| L-Proline/Substrate | 50/100 mM | 1.0 mM | 2.0/1.0 μL |
| *cis*P3H / *cis*P4H / *trans*P4H | 51/71/25 mg/mL | ~15 μM (0.5 mg/mL) | 1.0/2.0/0.7 μL |
|  |  | **Total** | **100 μL** |

Table S1. Proline hydroxylase LC/MS assay conditions.

*^a^* (NH_4_)_2_Fe(SO_4_)_2_ powder was dissolved in 20 mM HCl_(aq)_ solution to give a 100 mM solution; a sample of the Fe^2+^ solution was then diluted 20-fold with deionised water to give a 5 mM NH_4_)_2_Fe(SO_4_)_2_ stock solution.

The incubation mixture was kept at 21 °C for 14 h (unless otherwise stated). To quench the reaction, an equal volume of methanol was added and the mixture cooled on ice for 10 min before centrifugation (13,000*g* for 3 min); the quenching methanol contained 0.25 mM *p*-aminosalicylic acid (*p*ASA) as an internal standard. The supernatant was decanted and analysed by an LC/MS. ‘Negative controls’ were performed in parallel, but with substitution of 50 mM MES-NaOH, pH 6.5 for the enzyme solution.

HPLC employed a SiELC Primesep 100 column (250 mm × 4.6 mm, 10 μm pore size) connected to a Waters 1525μ Binary HPLC Pump system with a Waters 2777 Sample Manager (equipped with a Hamilton 100 μL syringe); detection was achieved with a Micromass^®^ Quattro Micro™ API mass spectrometer (positive electrospray ionization [ESI-MS]). Typically, 20-80 μL of sample (from 100 μL) was injected.

*Analytical HPLC Method*

| Eluent **A**: | 0.1% (v/v) HCOOH in H_2_O |
| --- | --- |
| Eluent **B**: | 0.1% (v/v) HCOOH in MeCN |

The column was equilibrated at 1 mL/min with 5% eluent **B**. After 10 min, a linear gradient was run to 70% **B** over 20 min. The column was washed with 100% **B** for 10 min before the column was re-equilibrated at 5% **B** for 20 min (Table S2).

| **Time/min** | **Flow/mL min^-1^** | **% Buffer A** | **% Buffer B** |
| --- | --- | --- | --- |
| 0.00 | 1.00 | 95.0 | 5.0 |
| 15.00 | 1.00 | 30.0 | 70.0 |
| 24.00 | 1.00 | 0.0 | 100.0 |
| 29.00 | 1.00 | 0.0 | 100.0 |
| 30.00 | 1.00 | 95.0 | 5.0 |
| 40.00 | 1.00 | 95.0 | 5.0 |

Table S2. SiELC Primesep 100 HPLC Method for LC/MS analysis of assay samples.

Buffer A: 0.1% (v/v) FA in H_2_O.

Buffer B: 0.1% (v/v) in MeCN.

### Preparative-scale PH Reactions: LC/MS-purification Methods

Products for NMR analysis were typically produced by scaling up analytical reactions 20-fold (2×1 mL total), and kept at 21 °C for 14 h (unless otherwise stated). Reactions were quenched with cold methanol (1:1, v/v), then were cooled on ice for 10 min until precipitation was complete; and the solution was then clarified by centrifugation (13,000*g*). The supernatant solution was then diluted 2-fold with water and freeze-dried. Products were redissolved in 300 μL of 20% (v/v) MeOH in water for LC/MS guided purification.

*Preparative HPLC Methods*

Products with relatively weak polarity (*e.g.* when using substrate such as the *t*CMP methyl ester and the bicyclic compounds) were purified sequentially using:

(i) a Waters Spherisorb column (250 mm × 10 mm, 5 μ) pre-equilibrated in 5% (v/v) aqueous MeOH; elution was achieved by increasing the gradient to 10% aqueous MeOH with 0.1% (v/v) aqueous formic acid;

(ii) a preparative C18 column (250 mm x 22 mm, 15 μ) pre-equilibrated in 5% aqueous MeCN (ACN) with 0.1% (v/v) aqueous formic acid, then a gradient to 100 % MeCN (ACN) with 0.1% formic acid over 40 min. Elution was monitored using a Micromass^®^ Quattro micro™ API mass spectrometer (equipped with a Waters 1525μ Binary HPLC Pump system coupled to a Waters 2777 Sample Manager). Fractions (typically 5-15 mL) containing substances with *m*/*z* values corresponding to product) were collected in 50 mL Falcon tubes and freeze-dried.

Intermediate-polarity products were purified by two column steps (multiple injections) both using a SiELC Primesep 100 column (Table S3). Fractions containing products with the product *m*/*z* values were collected in 50 mL Falcon tubes and freeze-dried.

| **Time/min** | **Flow/mL min**^-1^ | **% Buffer A** | **% Buffer B** |
| --- | --- | --- | --- |
| 0.00 | 1.50 | 99.0 | 1.0 |
| 10.00 | 1.50 | 99.0 | 1.0 |
| 15.00 | 1.50 | 90.0 | 10.0 |
| 20.00 | 1.50 | 0.0 | 100.0 |
| 29.00 | 1.50 | 99.0 | 1.0 |
| 30.00 | 1.50 | 99.0 | 1.0 |

Table S3. SiELC Primesep 100 HPLC Method for LC/MS purification of immediate-polarity products.

Buffer A: 0.05% (v/v) FA in H_2_O.

Buffer B: 0.1% (v/v) FA in MeCN.

Products with relatively high polarity (*e.g.* as produced when using fluoroproline, hydroxyproline substrates, or where dihydroxylated products are produced) were purified as in Table S4. Fractions containing products with anticipated *m*/*z* values were collected in 50 mL Falcon tubes and freeze-dried.

| **Time/min** | **Flow/mL min**^-1^ | **% Buffer A** | **% Buffer B** |
| --- | --- | --- | --- |
| 0.00 | 1.50 | 99.9 | 0.1 |
| 10.00 | 1.50 | 99.9 | 0.1 |
| 15.00 | 1.50 | 90.0 | 10.0 |
| 20.00 | 1.50 | 0.0 | 100.0 |
| 29.00 | 1.50 | 99.9 | 0.1 |
| 30.00 | 1.50 | 99.9 | 0.1 |

Table S4. SiELC Primesep 100 HPLC Method for LC/MS purification of very polar products.

Buffer A: 0.05% (v/v) FA in H_2_O.

Buffer B: 0.1% (v/v) FA in ACN.

The resultant products were resuspended in D_2_O (600 μL), transferred to an Eppendorf vial, then freeze-dried. For NMR analyses, samples were resuspended in 16 μL of 99.9% (D atom) D_2_O [containing 0.05% (w/v) 3-(trimethylsilyl)propionic-2,2,3,3-*d*_4_ acid (TSP-*d*_4_)]; the resulting solution was then transferred into a 1 mm NMR tube (Bruker) using a 10 μL syringe and a hand centrifuge, then analyzed by NMR.

### End-point NMR Assays

Analytical incubations were performed by sequential addition of the reagents in Table S5 to a 1.5 mL Eppendorf tube (500 μL total volume):

| **Reaction Component** | **Stock Concentrations** | **Final Concentration** | **Volume/μL** |
| --- | --- | --- | --- |
| 50 mM Tris-DCl, pD 7.5 | 50.0 mM | - | (380/414.5) / (375/409.5) / (378/412.5) μL |
| Sodium Ascorbate | 100 mM | 2.0/1.0 mM | 10.0/5.0 μL |
| (NH_4_)_2_Fe(SO_4_)_2_ | 5 mM*^a^* | 0.5/0.25 mM | 50.0/25.0 μL |
| 2-Oxoglutarate (2- OG) | 100 mM | 10.0 mM | 5.0/0.5 μL |
| L-Proline/Substrate | 50 mM | 5.0 mM | 50.0 μL |
| *cis*P3H / *cis*P4H / *trans*P4H | 51/71/25 mg/mL | ~15 μM (0.5 mg/mL) | 5.0/10.0/7.0 μL |
|  |  | **Total** | **500 μL** |

Table S5. Proline hydroxylase end-point NMR assay conditions for proline analogue/product detection.

*^a^* (NH_4_)_2_Fe(SO_4_)_2_ powder was dissolved in 20 mM HCl_(aq)_ solution to give a 100 mM solution; a sample of the Fe^2+^ solution was then diluted 20-fold with pure D_2_O to give a 5 mM (NH_4_)_2_Fe(SO_4_)_2_ stock solution.

Reactions were incubated at 21 °C for 14 h (unless otherwise stated), then transferred to a 5 mm NMR tube for analysis using a 500 MHz (^1^H)/470.4 MHz (^19^F) Bruker AVII 500 NMR spectrometer [equipped with a 5 mm TFI-^1^H/^19^F(^13^C) probe]. Typically, ^1^H-NMR spectra (and proton-decoupled-^19^F spectra) were measured for each sample (usually ~128 scans); additional ^1^H-NMR spectra were also measured using an excitation-sculpting solvent suppression pulse sequence.

# Assignment of Reported Enzyme-catalysed Products

NMR spectra were recorded at 298 K using a Bruker AVIII 700 MHz spectrometer equipped with a ^1^H/^13^C/^15^N TCI-inverse cryoprobe, optimised for ^1^H observation (using TOPSPIN 3.1 software), unless otherwise stated. Experimentally observed chemical shifts were referenced to the D_2_O solvent shift (HDO: δ_H_ = 4.701 ppm); the deuterium signal was used as an internal lock signal, and the HDO signal was supressed by presaturation (where necessary). ^1^H-NMR spectra employed baseline optimisation (using the ‘zgpr’ pulse sequence) and selective 1D ge-TOCSY spectra were measured using an SPFGSE DIPSI2 with ZQS experiment (‘spfgsedipsi2zs’ pulse sequence). Where possible, products were analysed by 2D COSY and NOESY (mixing time 800 ms). Stereochemistries were assigned through combined analysis of ^3^*J*_HH_ coupling constants and NOE analyses.

Samples were prepared in 1 mm tubes containing (16 μL) D_2_O (unless otherwise stated); for most proline hydroxylase products of catalysis, trimethylsilane propionic acid sodium salt (TSP-*d*_4_) was used as an external standard [0.05% (w/v)] and as a chemical shift reference during final analysis (δ_H_ = ‘0.00 ppm’). In all cases, the LC/MS analyses (positive or negative ion electrospray ionization) supported the formation of the assigned products, as shown by observation of the molecular ion and/or the ion arising from decarboxylation of the product.

## *Substrate Turnover Information*

| Proline Hydroxylase Non-substrates | | | | | | |
| --- | --- | --- | --- | --- | --- | --- |
| Different Ring Sizes | | | | | | |
|  | |  | |  | |  |
| (2*S*)-L-aziridine-2-carboxylic acid (Azy) | |  |  |  |  |  |
| D-Stereochemistry | | | | | | |
|  |  | | |  | |  |
| (2*R*)-D-proline | (2*R*)- D-pipecolic acid | | | (2*R*)-D-pyroglutamic acid | | (2*R*,4*S*)-*N*-acetyl-*cis*-4-hydroxy-  D-proline |
| *N*-Acyl and *N*-Alkyl Substrate Analogues | | | | | | |
|  |  | | |  | |  |
| (2*S*)-*N*-acetyl-L-proline | (2*S*)-*N*-acetyl-*trans*-4-hydroxy‑  L-proline | | | Captopril | | L-pyroglutamic acid |
|  |  | | |  | |  |
| *N*-benzyl-L-proline | *N*-(*N*-Boc-piperidin-4-yl)-L-proline | | |  | |  |
| Carboxylic-Acid-Derivatised Substrate Analogues | | | | | | |
|  |  | | |  | |  |
| L-prolinamide |  | | |  | |  |
| Ring-Substituted Substrate Analogues | | | | | | |
|  |  | | |  | |  |
| (2*R*)-2-(4-methylbenzyl)-L-proline | (2*R*,5*S*)-L-pyrrolidine-2,5-dicarboxylic acid | | | (2*R*,5*S*)-*N*-methyl-L-pyrrolidine-2,5-dicarboxylic acid | | (2*R*,6*S*)-L-piperidine-2,6-  dicarboxylic acid |
|  | | |  | |  | |
| (2*R*,5*R*)-*cis*-5-(carboxymethyl)-L-proline (*c*CMP) | | | (2*R*,5*S*)-*trans*-5-(carboxymethyl)-L-proline (*t*CMP) | | (2*S*)-4-oxo-L-proline | |
| Bicyclic Compounds | | | | | | |
|  | |  | |  | |  |
| (1*R*,3*S*,4*S*)-2-azabicyclo[2.2.1]heptane-3-carboxylic acid | |  | |  | |  |

Table S6. Proline analogues found not to be substrates of the three proline hydroxylases by LC/MS and/or NMR.

| Substrate [and Product(s)] | | Product Yield(s) | | |
| --- | --- | --- | --- | --- |
| Natural Substrate | | *cis*P3H | *cis*P4H | *trans*P4H |
| (2*S*)-L-proline (**1**) | |  |  |  |
|  | (2*S*,3*S*)-*cis*-3-hydroxy**-**L-proline (**2**) | >95% | - | - |
|  | (2*S*,4*S*)-*cis*-4-hydroxy-L-proline (**3**) | - | >95% | - |
|  | (2*S*,4*R*)-*trans*-4-hydroxy-L-proline (**4**) | - | - | >95% |
| Different Ring Sizes | | | | |
| (2*S*)-L-azetidine-2-carboxylic acid (**5**) | |  |  |  |
|  | (2*S*,3*S*)-*cis*-3-hydroxy-L-azetidine-2-carboxylic acid (**6**) | >95% | 4% | 0% |
| (2*S*)-L-pipecolic acid (**7**) | |  |  |  |
|  | (2*S*,3*R*)-*cis*-3-hydroxy-L-pipecolic acid (**8**) | >95% | 38% | - |
|  | (2*S*,5*S*)-*cis*-5-hydroxy-L-pipecolic acid (**9**) | - | 45% | - |
|  | (2*S*,3*R*,5*S*)-3,5-dihydroxy-L-pipecolic acid (**10**) | - | 17% | - |
|  | (2*S*,5*R*)-*trans*-5-hydroxy-L-pipecolic acid (**11**) | - | - | >95% |
| (2*S*)-L-azepane-2-carboxylic acid (**12**) | |  |  |  |
|  | (2*S*,3*R*)-*cis*-3-hydroxy-L-azepane-2-carboxylic acid (**13**) | 31% | 35% | - |
|  | (2*S*,5*R*)-*trans*-5-hydroxy-L-azepane-2-carboxylic acid (**14**) | - | - | 8% |
|  | (2*S*)-hydroxy-L-azepane-2-carboxylic acid (**15**) | 5% | 8% | - |
|  | (2*S*)-hydroxy-L-azepane-2-carboxylic acid (**16**) | - | 3% | - |
|  | (2*S*)-dihydroxy-L-azepane-2-carboxylic acid (**17**) | 3% | - | - |
| *N*-Methylated Substrate Analogues | |  |  |  |
| (2*S*)-*N*-methyl-L-proline (**18**) | |  |  |  |
|  | (2*S*,3*R*)-*cis*-3-hydroxy**-***N*-methyl-L-proline (**19**) | >95% | - | - |
|  | (2*S*,4*S*)-*cis*-4-hydroxy**-***N*-methyl-L-proline (**20**) | - | >95% | - |
|  | (2*S*,4*S*)-*cis*-4-hydroxy**-***N*-methyl-L-proline (**21**) | - | 1% | 5% |
| (2*S*)-*N*-methyl-L-pipecolic acid (**22**) | |  |  |  |
|  | (2*S*,3*R*)-*cis*-3-hydroxy**-***N*-methyl-L-pipecolic acid (**23**) | 24% | 11% | - |
|  | (2*S*,5*S*)-*cis*-5-hydroxy**-***N*-methyl-L-pipecolic acid (**24**) | - | 5% | - |
|  | (2*S*,5*R*)-*trans*-5-hydroxy**-***N*-methyl-L-pipecolic acid (**25**) | - | - | >95% |
|  | (2*S*,3*R*,5*S*)-3,5-dihydroxy**-***N*-methyl-L-pipecolic acid (**26**) | 5% | - | - |
| Ring-Substituted Substrate Analogues | |  |  |  |
| (2*S*)-2-methyl-L-proline (**27**) | |  |  |  |
|  | (2*S*,3*R*)-*cis*-3-hydroxy-2-methyl-L-proline (**28**) | 1% | 1% | - |
|  | (2*S*,4*S*)-*cis*-4-hydroxy-2-methyl-L-proline (**29**) | - | 4% | - |
|  | (2*S*,4*R*)-*trans*-4-hydroxy-2-methyl-L-proline (**30**) | - | - | >95% |
| (2*S*,4*R*)-*trans*-4-(prop-2-yn-1-yl)-L-proline (**31**) | |  |  |  |
|  | (2*S*,3*R*,4*S*)-3-hydroxy-4-(prop-2-yn-1-yl)-L-proline (**32**) | 26% | 21% | - |
| (2*S*,5*S*)- *trans*-5-(2-methoxy-2-oxoethyl)-L-proline (**33**) | |  |  |  |
|  | (2*S*,3*R*,5*S*)-3-hydroxy-5-(2-methoxy-2-oxoethyl)-L-proline (**34**) | 63% | - | - |
| (2*S*,4*S*)-*cis*-4-fluoro-L-proline (**35**) | |  |  |  |
|  | (2*S*,3*S*,4*R*)-4-fluoro-3-hydroxy-L-proline (**36**) | 35% | 30% | - |
|  | 4-oxo-L-proline (**37**) | - | - | ~6% |
| (2*S*,4*R*)-*trans*-4-fluoro-L-proline (**38**) | |  |  |  |
|  | (2*S*,3*S*,4*S*)-4-fluoro-3-hydroxy-L-proline (**39**) | 29% | 26% | - |
|  | 4-oxo-L-proline | - | 3% | - |
| (2*S*,3*S*)-*trans*-3-hydroxy-L-proline (**40**) | |  |  |  |
|  | (2*S*,3*R*,4*S*)-3,4-dihydroxy-L-proline (**41**) | - | - | 16% |
| (2*S*,4*S*)-*cis*-4-hydroxy-L-proline (**3**) | |  |  |  |
|  | (2*S*,3*S*,4*R*)-3,4-dihydroxy-L-proline (**42**) | 22% | - | - |
| (2*S*,4*S*)-*cis*-4-hydroxy-*N*-methyl-L-proline (**20**) | |  |  |  |
|  | (2*S*,3*S*,4*R*)-3,4-dihydroxy-*N*-methyl-L-proline (**43**) | 1% | - | - |
| (2*S* ,4*R*)-*trans*-4-hydroxy-L-proline (**4**) | |  |  |  |
|  | (2*S*)-3,4,4‘-trihydroxy-L-proline (**44**) | 7% | 15% | - |
|  | (2*S*)-3-hydroxy-4-oxo-L-proline (**45**) | 4% | 7% | - |
|  | (2*S*)-3-hydroxy-4-oxo-L-proline / (2*S*)-4-hydroxy-3-oxo-L-proline (**46**) | 4% | 3% | - |
| Bicyclic Compounds | |  |  |  |
| (2*S*,3a*S*,6a*S*)-octahydropenta[*b*]pyrrole-2-carboxylic acid (**47**) | |  |  |  |
|  | (2*S*,3a*S*,4*R*,6a*S*)-4-hydroxy-L-octahydropenta[*b*]pyrrole-2-carboxylic acid (**48**) | 17% | 4% | - |
|  | (2*S*,3*R*,3a*R*,6a*S*)-3-hydroxy-L-octahydropenta[*b*]pyrrole-2-carboxylic acid (**49**) | 5% | - | - |
|  | (2*S*,3a*S*,4*S*,6a*S*)-4-hydroxy-L-octahydropenta[*b*]pyrrole-2-carboxylic acid (**50**) | - | - | ~1% |
| (2*S*,3a*R*,6a*R*)-octahydropenta[*b*]pyrrole-2-carboxylic acid (**51**) | |  |  |  |
|  | (2*S*,3*R*,3a*S*,6a*R*)-3-hydroxy-L-octahydropenta[*b*]pyrrole-2-carboxylic acid (**52**) | 25% | - | - |
|  | (2*S*,3a*R*,4*R*,6a*R*)-4-hydroxy-L-octahydropenta[*b*]pyrrole-2-carboxylic acid (**53**) | 16% | - | - |
|  | (2*S*,3a*R*,4S,6a*R*)-4-hydroxy-L-octahydropenta[*b*]pyrrole-2-carboxylic acid (**54**) | - | - | ~1% |
| (2*S*,3a*S*,7a*S*)-octahydro-1H-indole-2-carboxylic acid (**55**) | |  |  |  |
|  | (2*S*,3a*S*,4*R*,7a*S*)-4-hydroxyoctahydro-1*H*-indole-2-carboxylic acid (**56**) | 4% | - | - |
| decanhydroisoquinoline-3-carboxylic acid (**57**) | |  |  |  |
|  | Hydroxyl-decanhydroisoquinoline-3-carboxylic acid (**58**) | 5% | - | - |
|  | Hydroxyl-decanhydroisoquinoline-3-carboxylic acid (**59**) | - | - | 8% |
| Unsaturated Compounds | |  |  |  |
| (2*S*)-3,4-dehydro-L-proline (60) | |  |  |  |
|  | (2*S*,3*S*,4*R*)-*cis*-3,4-Epoxy-L-proline (61) | 1% | <0.5% | 1 |
|  | (2*S*,3*R*,4*S*)-*trans*-3,4-Epoxy-L-proline (62) | - | - | <0.5% |
| (2*S*)-4,5-dehydro-L-pipecolic acid (63) | |  |  |  |
|  | (2*S*,4*S*,5*R*)-*cis*-4,5-epoxy-L-proline (64) /  (2*S*,3*S*)-*cis*-3-hydroxyy-4,5-epoxy-L-proline (**65**) | <0.5% | - | - |
| **Table S7** Summary of proline hydroxylase transformation products and approximate yields, where possible to determine. Note the observed yields shall be regarded as approximate/provisional and likely can be optimised. (-) = not observed. | | | | |

## *Substrate Analogues with Different Ring Sizes*

1. *(2S)-L-Azetidine-2-carboxylic Acid (****5****)*

Scheme S1. *cis*P3H and *cis*P4H catalyse production of *cis*-3-hydroxy-L-azetidine-2-carboxylic acid (6) from L-azetidine-2-carboxylic acid (5).


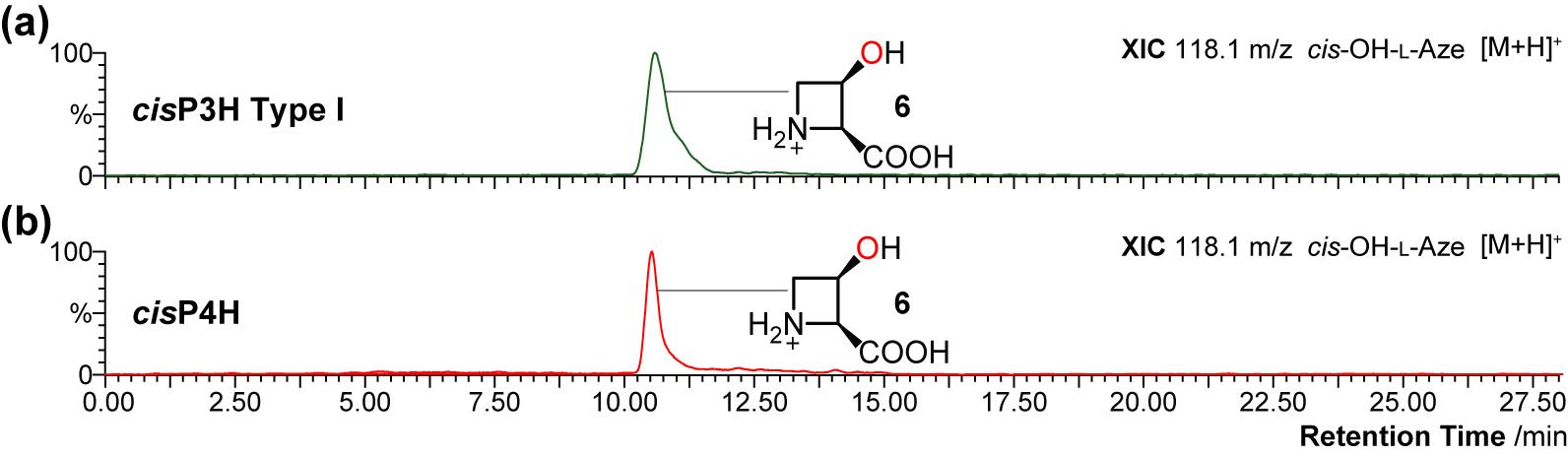


Fig. S5. Extracted-ion count (XIC) LC/MS chromatograms for the proline hydroxylase reactions using (2*S*)-L-azetidine-2-carboxylic acid (5): (a) *cis*P3H and (b) *cis*P4H reactions yield *cis*-3-hydroxy-L-azetidine-2-carboxylic acid (6).


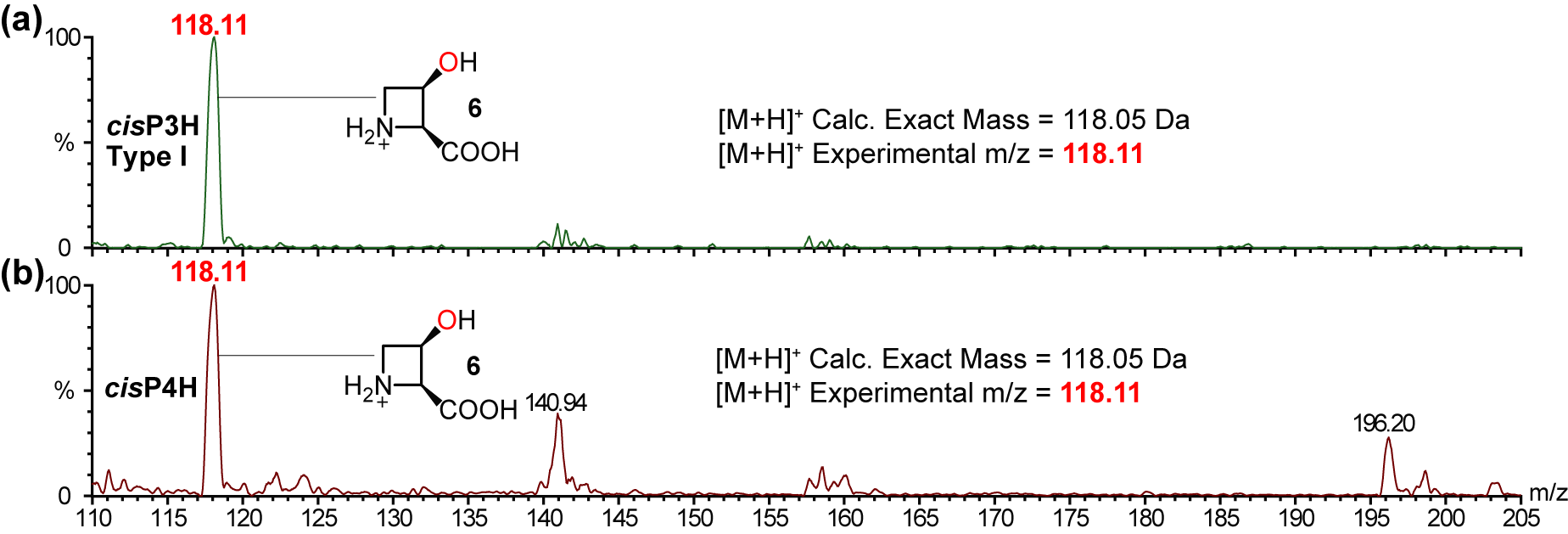


Fig. S6. LC/MS spectra of proline hydroxylase reactions with (2*S*)-L-azetidine-2-carboxylic acid (Aze) (5): (a) *cis*P3H and (b) *cis*P4H reactions yield *cis*-3-hydroxy-L-azetidine-2-carboxylic acid (6).


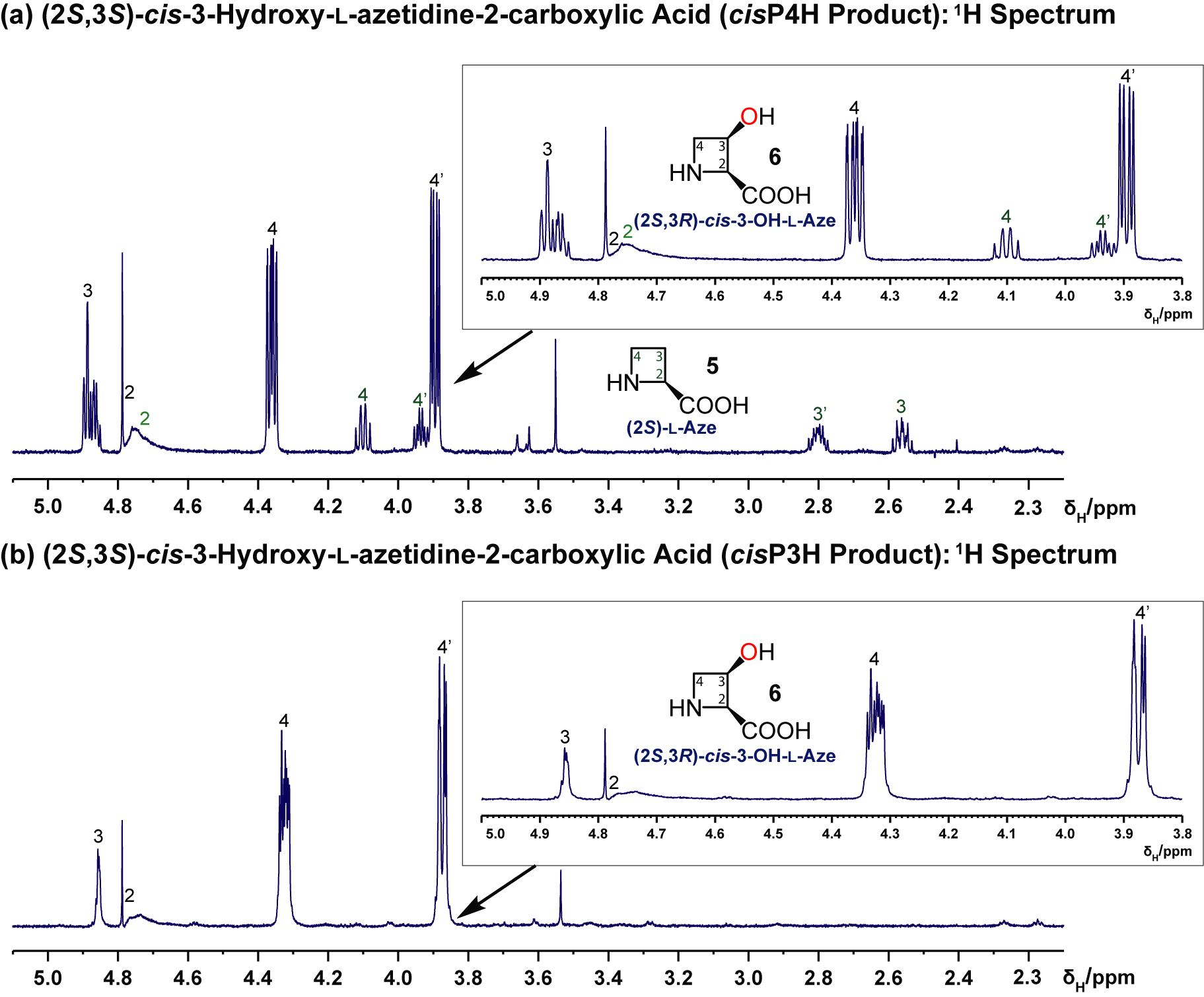


Fig. S7. ^1^H-NMR of the hydroxylation products from (a) *cis*P4H and (b) *cis*P3H reactions with (2*S*)-L-azetidine-2-carboxylic acid (5): both *cis*P3H and *cis*P4H yield (2*S*,3*R*)-*cis*-3-hydroxy-L-azetidine-2-carboxylic acid (6). Chemical shift values are referenced to TSP-*d*_4_ (‘0.0 ppm’).

**Assignments**

^1^H NMR (700 MHz, D_2_O) δ = 4.88 (dddd, *J =* 6.7, 4.8, 1.6, 0.8 Hz, 1H), 4.36 (ddd, *J* = 11.5, 6.7, 1.6 Hz, 1H), 3.90 (ddd, *J* = 11.5, 4.8, 0.8 Hz, 1H).

*Note:*  the H-2 peaks (and in (b) the H-3 peak) are obscured by the residual HDO solvent peak (which was suppressed).


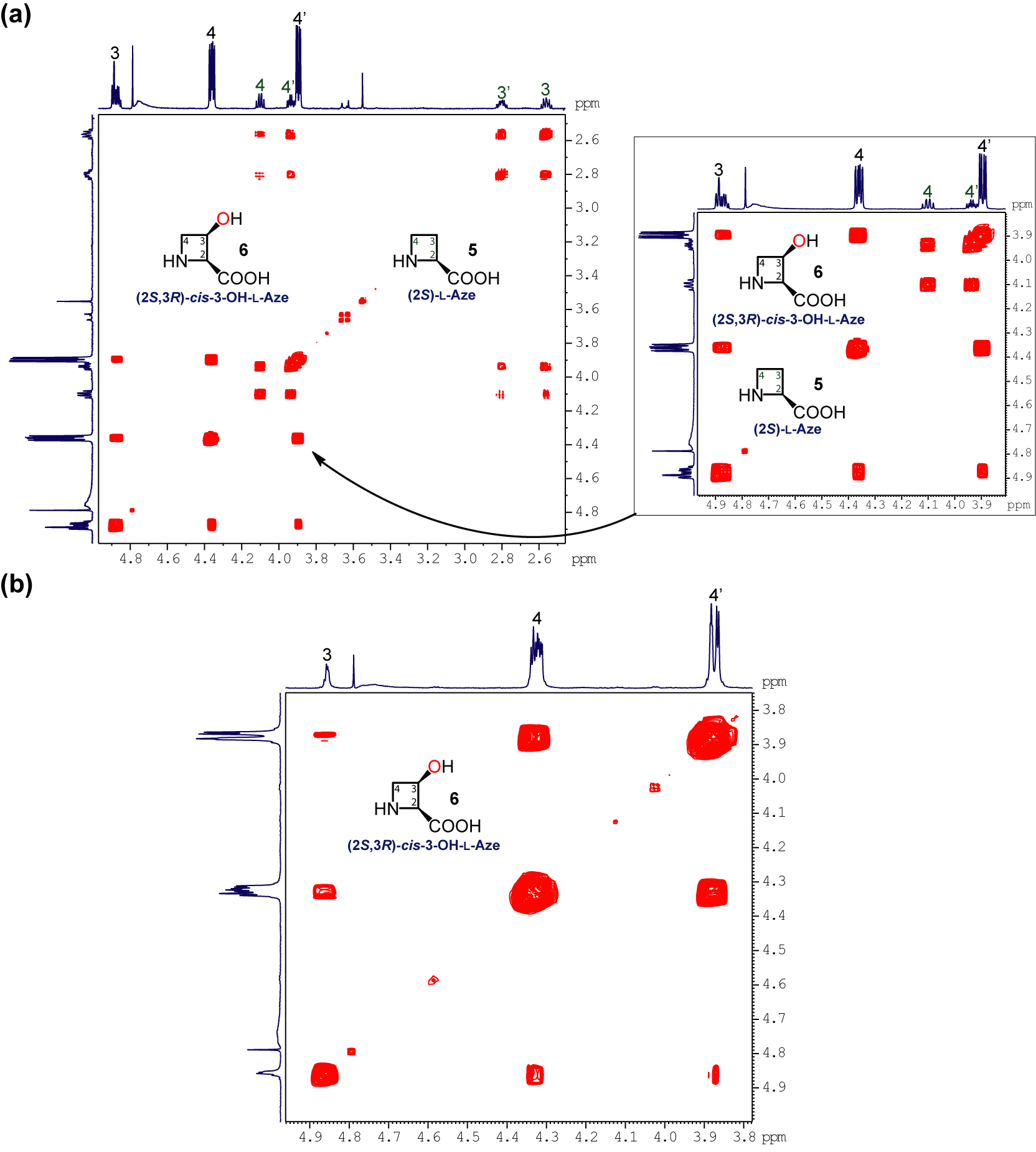


Fig. S8. ^1^H-^1^H-COSY-NMR analysis of the hydroxylation products from (a) *cis*P4H and (b) *cis*P3H reactions with (2*S*)-L-azetidine-2-carboxylic acid (5): NMR analysis assigns the products as (2*S*,3*R*)-*cis*-3-hydroxy-L-azetidine-2-carboxylic acid (6). Chemical shift values are referenced to TSP-*d*_4_ (‘0.0 ppm’).

1. *(2S)-L-Pipecolic Acid (Pip) (****7****)*

Scheme S2. Proline hydroxylase reactions using (2*S*)-L-pipecolic acid (Pip) (7):

(a) *cis*P3H catalyses production of (2S,3S)-*cis*-3-hydroxy-L-pipecolic acid (8);

(b) *cis*P4H catalyses production of (2S,3S)-*cis*-3-hydroxy-L-pipecolic acid (8), (2S,5S)-*cis*-5-hydroxy-L-pipecolic acid (9), and (2*S*,3*R*,5*S*)-3,5-dihydroxy-L-pipecolic acid (10);

(c) *trans*P4H catalyses production of (2*S*,5*S*)-*trans*-5-hydroxy-L-pipecolic acid (**11**).

Stereochemical assignments were made by NMR.


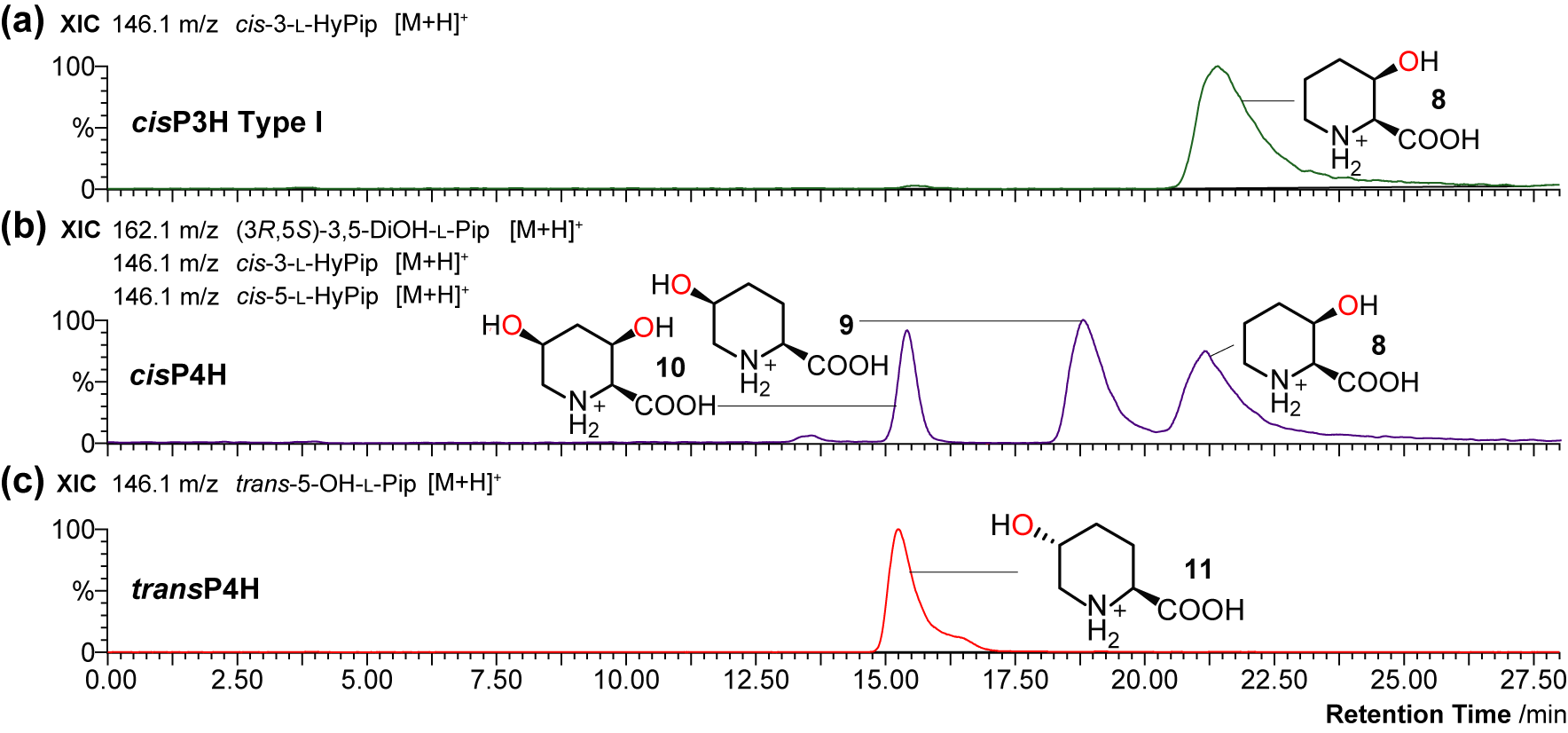


Fig. S9. Extracted-ion count (XIC) LC/MS chromatograms for proline hydroxylase reactions using (2*S*)-L-pipecolic acid (Pip) (7):

(a) *cis*P3H yields (2S,3R)-*cis*-3-hydroxy-L-pipecolic acid (8);

(b) *cis*P4H yields (2S,3R)-*cis*-3-hydroxy-L-pipecolic acid (8), (2S,5S)-*cis*-5-hydroxy-L-pipecolic acid (9), and (2*S*,3*R*,5*S*)-3,5-dihydroxy-L-pipecolic acid (10);

(c) *trans*P4H yields (2*S*,5*S*)-*trans*-5-hydroxy-L-pipecolic acid (**11**).

Stereochemical assignments were made by NMR.


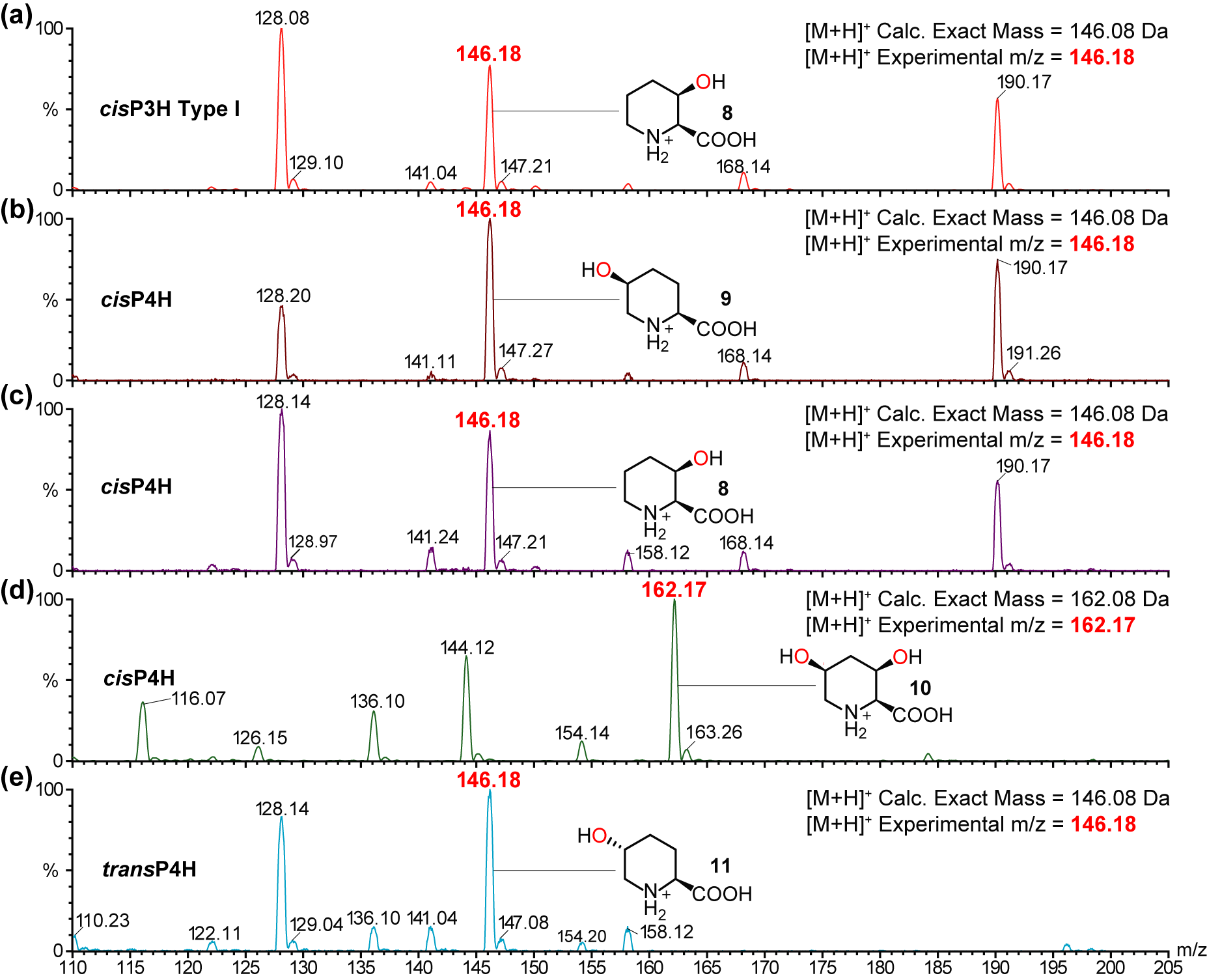


Fig. S10. LC/MS spectra of proline hydroxylase reactions using (2*S*)-L-pipecolic acid (Pip) (7):

(a) *cis*P3H reactions yield (2S,3R)-*cis*-3-hydroxy-L-pipecolic acid (8);

(b) *cis*P4H reactions yield (2S,5S)-*cis*-5-hydroxy-L-pipecolic acid (9);

(c) *cis*P4H reactions yield (2S,3R)-*cis*-3-hydroxy-L-pipecolic acid (8);

(d) *cis*P4H reactions yield (2*S*,3*R*,5*S*)-3,5-dihydroxy-L-pipecolic acid (**10**);

(e) *trans*P4H reactions yield (2*S*,5*S*)-*trans*-5-hydroxy-L-pipecolic acid (**11**).

Stereochemical assignments were made by NMR.


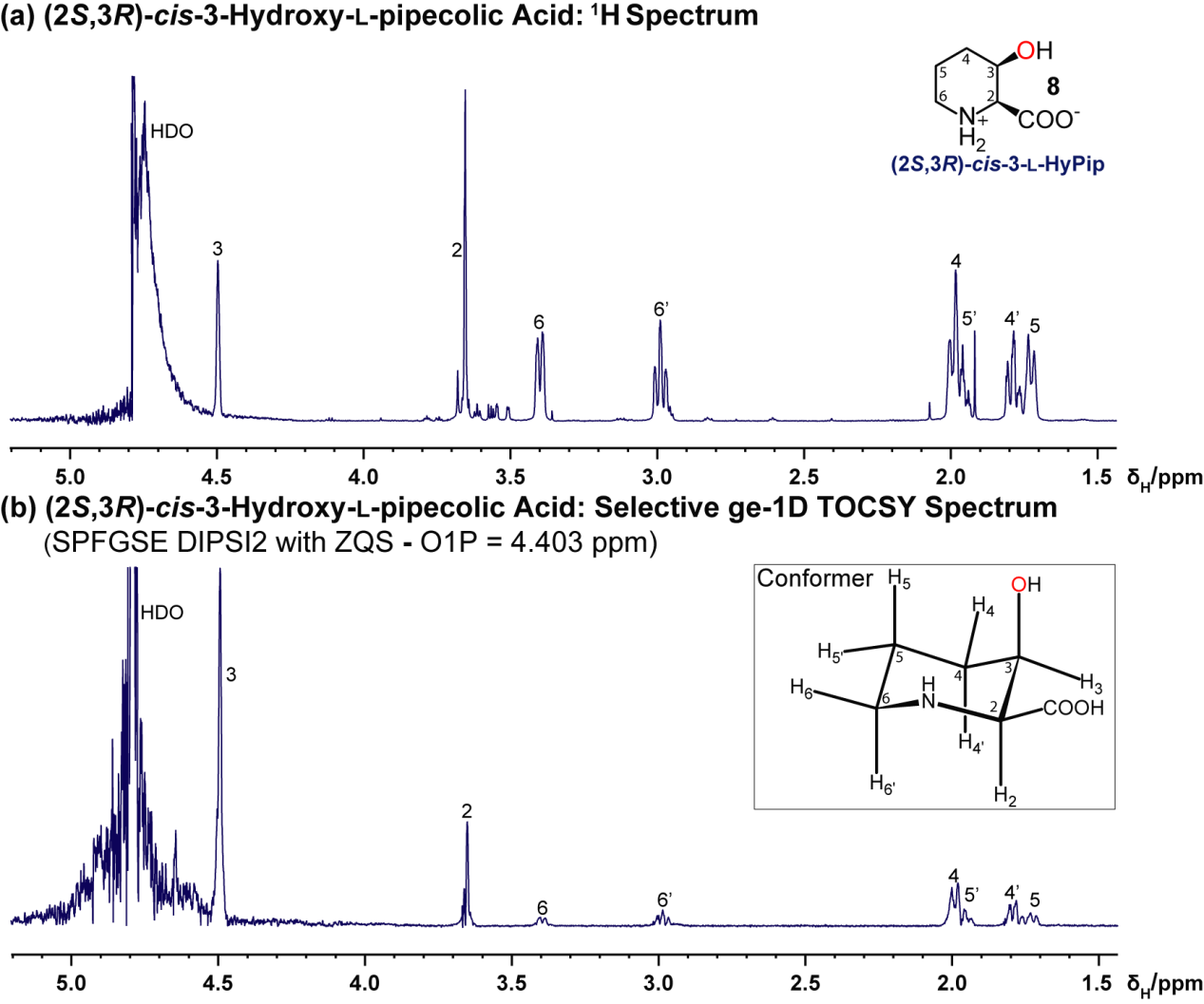


Fig. S11. NMR of the (2S,3R)-*cis*-3-hydroxy-L-pipecolic acid (8) hydroxylation product from *cis*P3H and *cis*P4H reactions using (2*S*)-L-pipecolic acid (Pip) (7): (a) ^1^H-NMR spectrum (‘zgpr’ pulse sequence) and (b) selective 1D ge-TOCSY spectrum (‘spfgsedipsi2zs’ pulse sequence). Chemical shift values are referenced to TSP-*d*_4_ (‘0.0 ppm’).

**Assignments**

^1^H NMR (700 MHz, D_2_O) δ = 4.50 (ddd, *J* = 2.3, 1.8 Hz, 1H), 3.65 (d, *J* = 1.9 Hz, 1H), 3.40
(ddd, *J* = 13.1, 4.4, 2.3 Hz, 1H), 2.99 (ddd, *J* = 13.1, 3.4, 1.8 Hz, 1H), 2.02 – 1.97 (m, 2H), 1.97 – 1.91 (m, 1H), 1.79 (ddd, *J* = 14.6, 4.4, 2.3 Hz, 1H), 1.73 (dddd, *J* = 14.2 Hz, 1H).


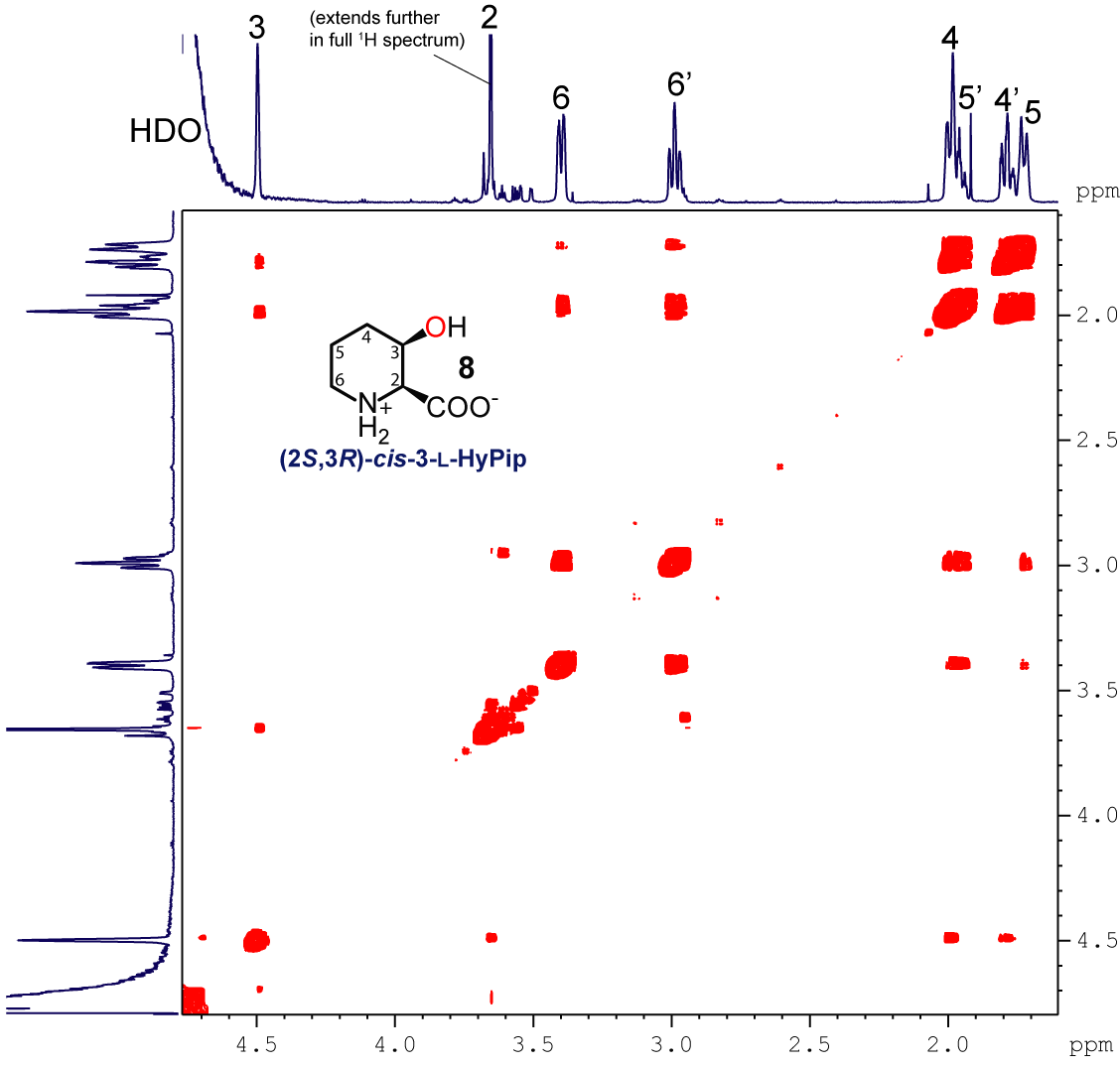


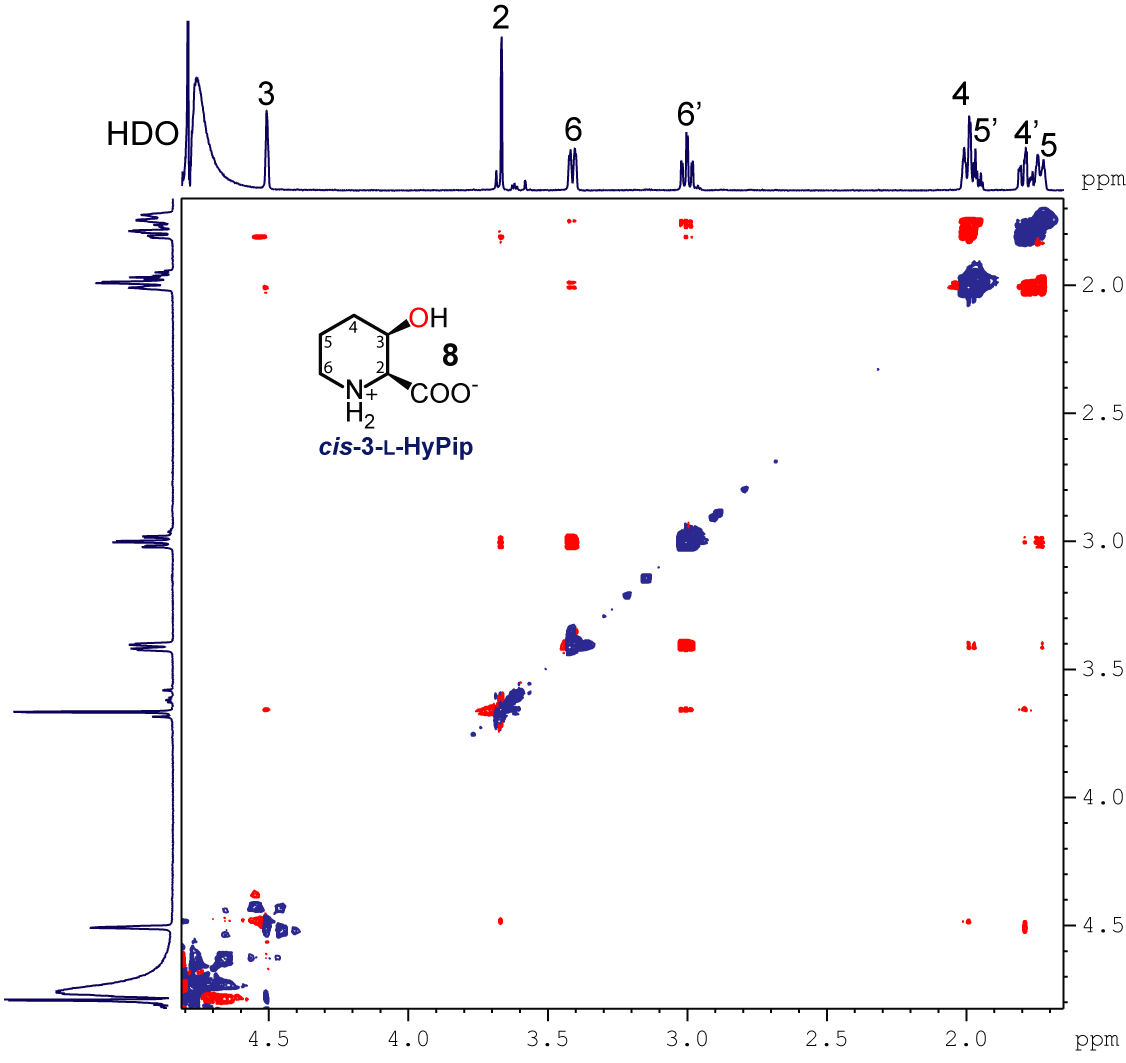


Fig. S12. 2D-NMR of the (2S,3R)-*cis*-3-hydroxy-L-pipecolic acid (8) hydroxylation product from *cis*P3H and *cis*P4H reactions using (2*S*)-L-pipecolic acid (Pip) (7):

(a) ^1^H-^1^H COSY spectrum (‘cosygpprf2qf’ pulse sequence);

(b) ^1^H-^1^H NOESY spectrum (‘noesyphprf2’ pulse sequence).

Chemical shift values are referenced to TSP-*d*_4_ (‘0.0 ppm’).

**(a)**

**(b)**


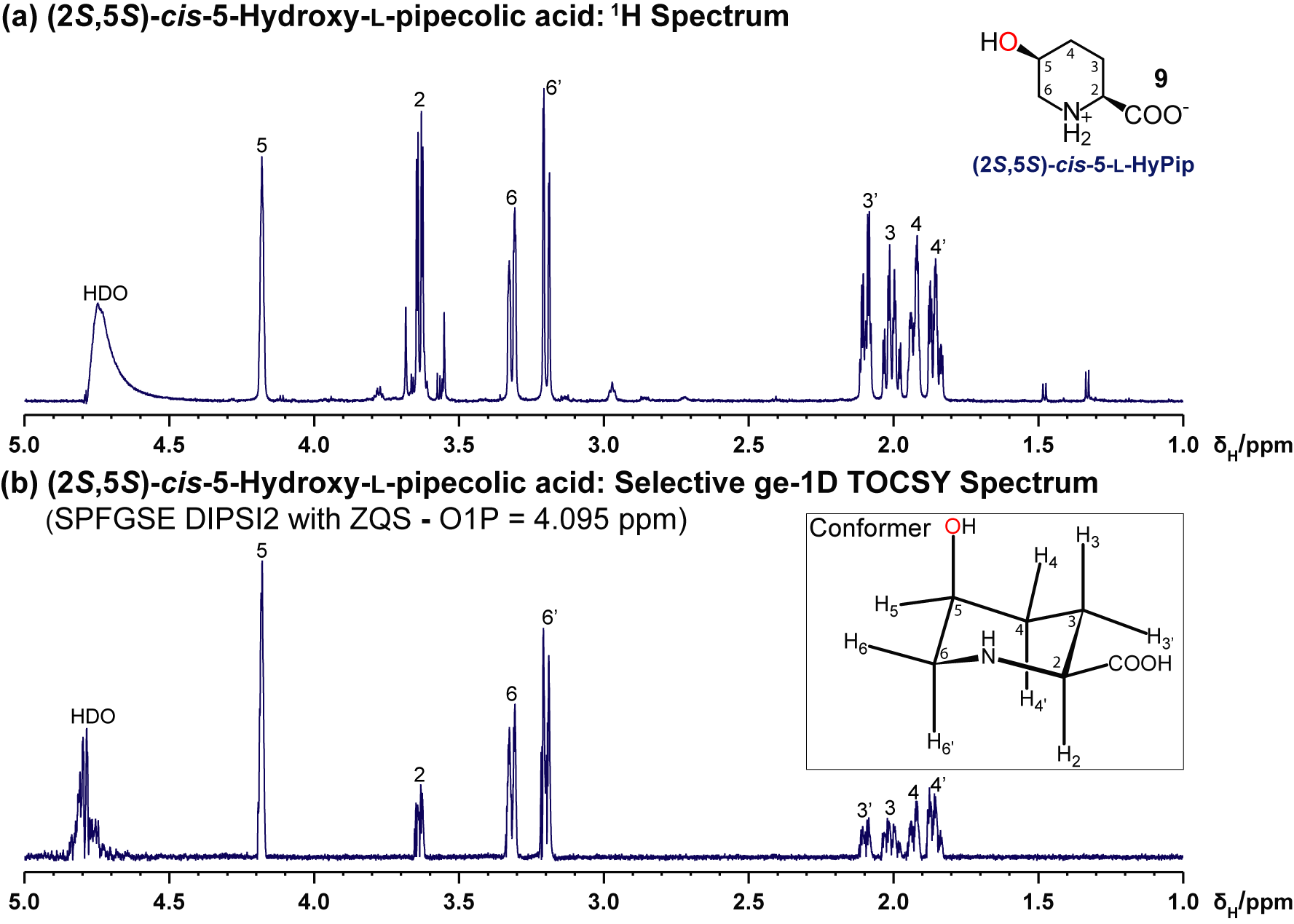


Fig. S13. NMR of the (2S,5S)-*cis*-5-hydroxy-L-pipecolic acid (9) hydroxylation product from *cis*P4H reactions using (2*S*)-L-pipecolic acid (Pip) (7): (a) ^1^H-NMR spectrum (‘zgpr’ pulse sequence) and (b) selective 1D ge-TOCSY (‘spfgsedipsi2zs’ pulse sequence). Chemical shift values are referenced to TSP-*d*_4_ (‘0.0 ppm’).

**Assignments**

^1^H NMR (700 MHz, D_2_O) δ = 4.18 (dddd, *J* = 4.4, 4.0 , 2.2 Hz, 1H), 3.64 (dd, *J* = 13.2, 3.7 Hz, 1H), 3.32 (dd, *J* = 13.2, 2.2 Hz, 1H), 3.20 (dd, *J* = 13.2, 2.2 Hz, 1H), 2.10 (dddd, *J* = 15.0, 14.3, 9.1, 4.4, 4.0 Hz, 1H), 2.01 (dddd, *J* = 15.0, 13.2, 11.8, 4.0 Hz, 1H), 1.95 – 1.90 (m, 1H), 1.85 (dddd, *J* = 14.7, 12.2, 4.4, 2.8 Hz, 1H).


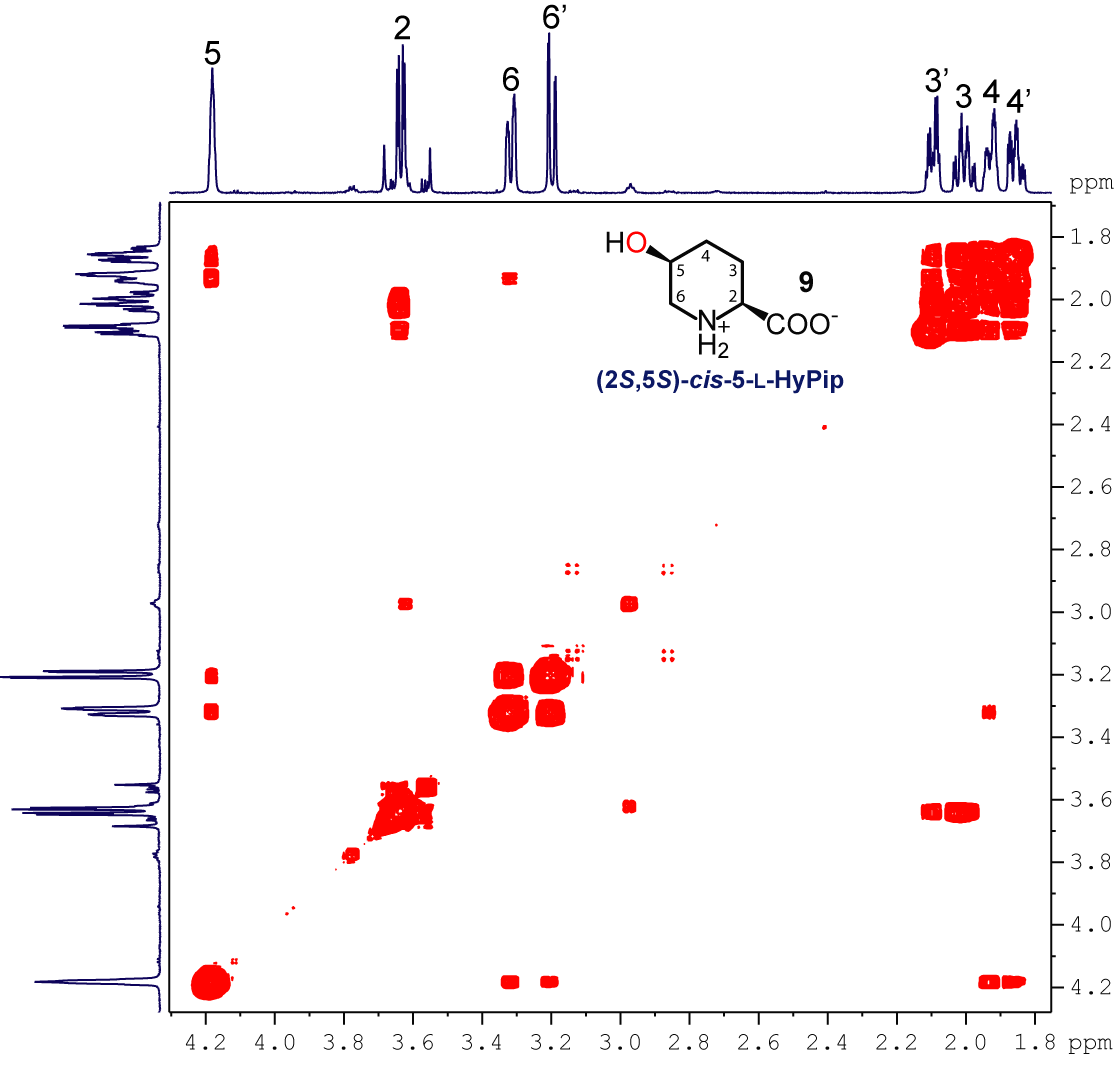


Fig. S14. 2D-NMR of the (2S,5S)-*cis*-5-hydroxy-L-pipecolic acid (9) hydroxylation product from *cis*P3H and *cis*P4H reactions using (2*S*)-L-pipecolic acid (Pip) (7): ^1^H-^1^H COSY spectrum (‘cosygpprf2qf’ pulse sequence). Chemical shift values are referenced to TSP-*d*_4_ (‘0.0 ppm’).


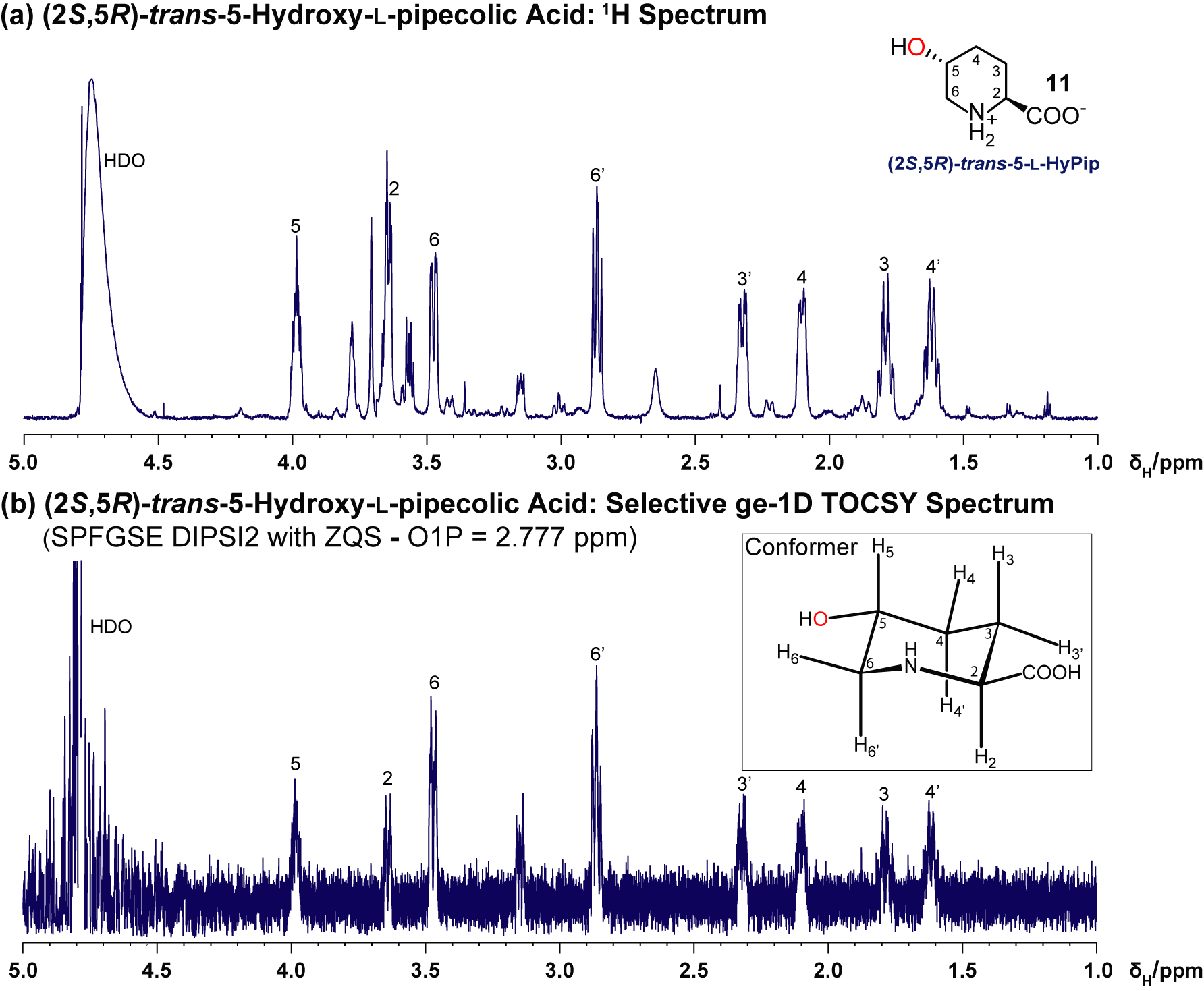


Fig. S15. NMR of the (2*S*,5*R*)-*trans*-5-hydroxy-L-pipecolic acid (11) hydroxylation product from *trans*P4H reactions using (2*S*)-L-pipecolic acid (Pip) (7): (a) ^1^H-NMR spectrum (‘zgpr’ pulse sequence) and (b) selective 1D ge-TOCSY (‘spfgsedipsi2zs’ pulse sequence). Chemical shift values are referenced to TSP-*d*_4_ (‘0.0 ppm’).

Assignments

^1^H NMR (700 MHz, D_2_O) δ = 3.99 (dddd, *J* = 14.7, 5.3, 4.7, 0.8 Hz, 1H), 3.64 (dd, *J* = 11.1, 10.9, 5.9, 3.7, 3.7, 3.3, 2.7 Hz, 1H), 3.47 (dd, *J* = 12.4, 3.2 Hz, 1H), 2.86 (dd, *J* = 12.4, 9.6 Hz, 1H), 2.32 (dddd, *J* = 15.0, 5.0 Hz, 1H), 2.11 (dddd, *J* = 13.7, 5.1, 4.1 Hz, 1H), 1.79 (dddd, *J* = 24.1, 10.7, 3.4 Hz, 1H), 1.62 (dddd, *J* = 18.1, 11.7, 4.6 Hz, 1H).


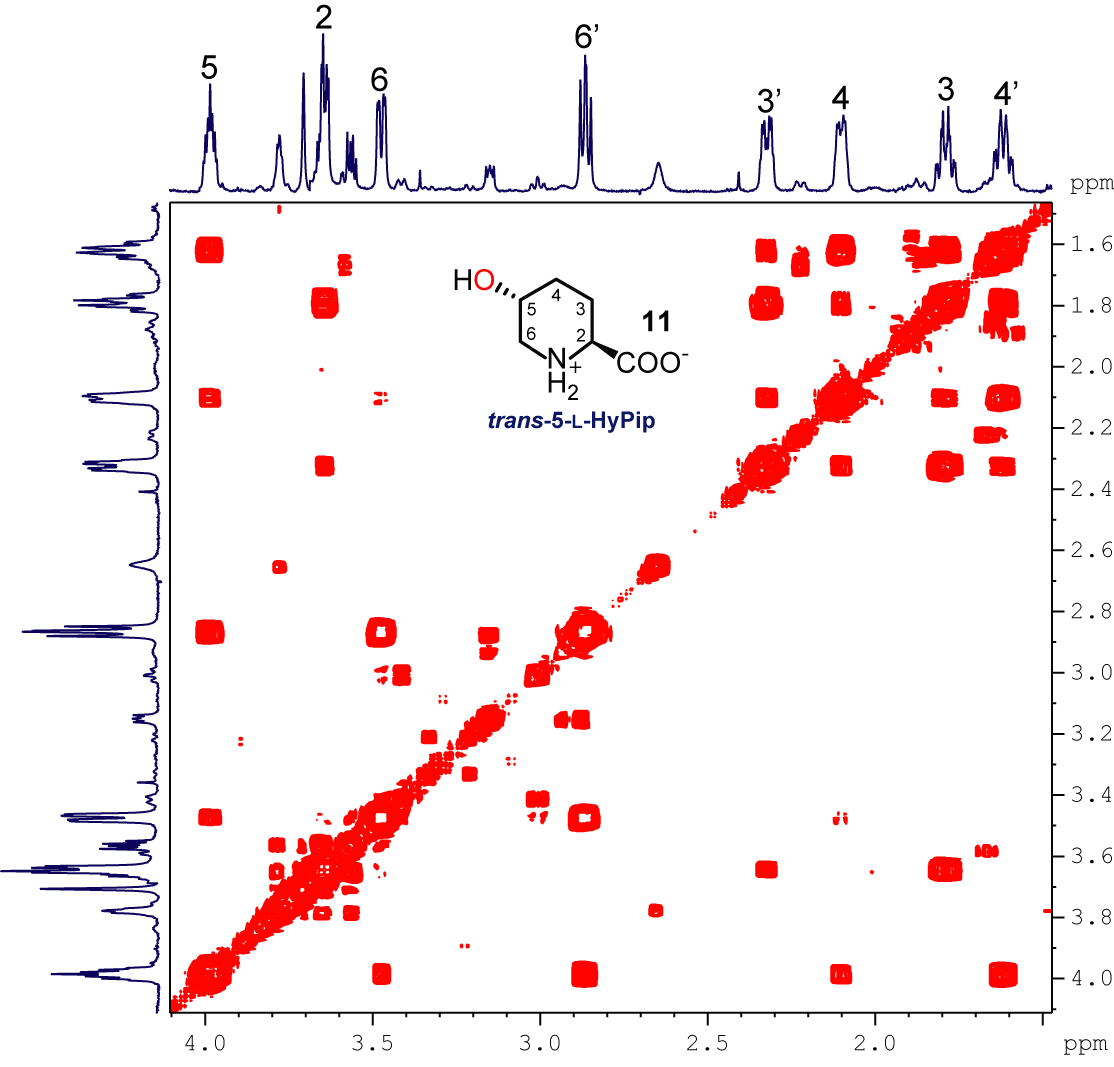

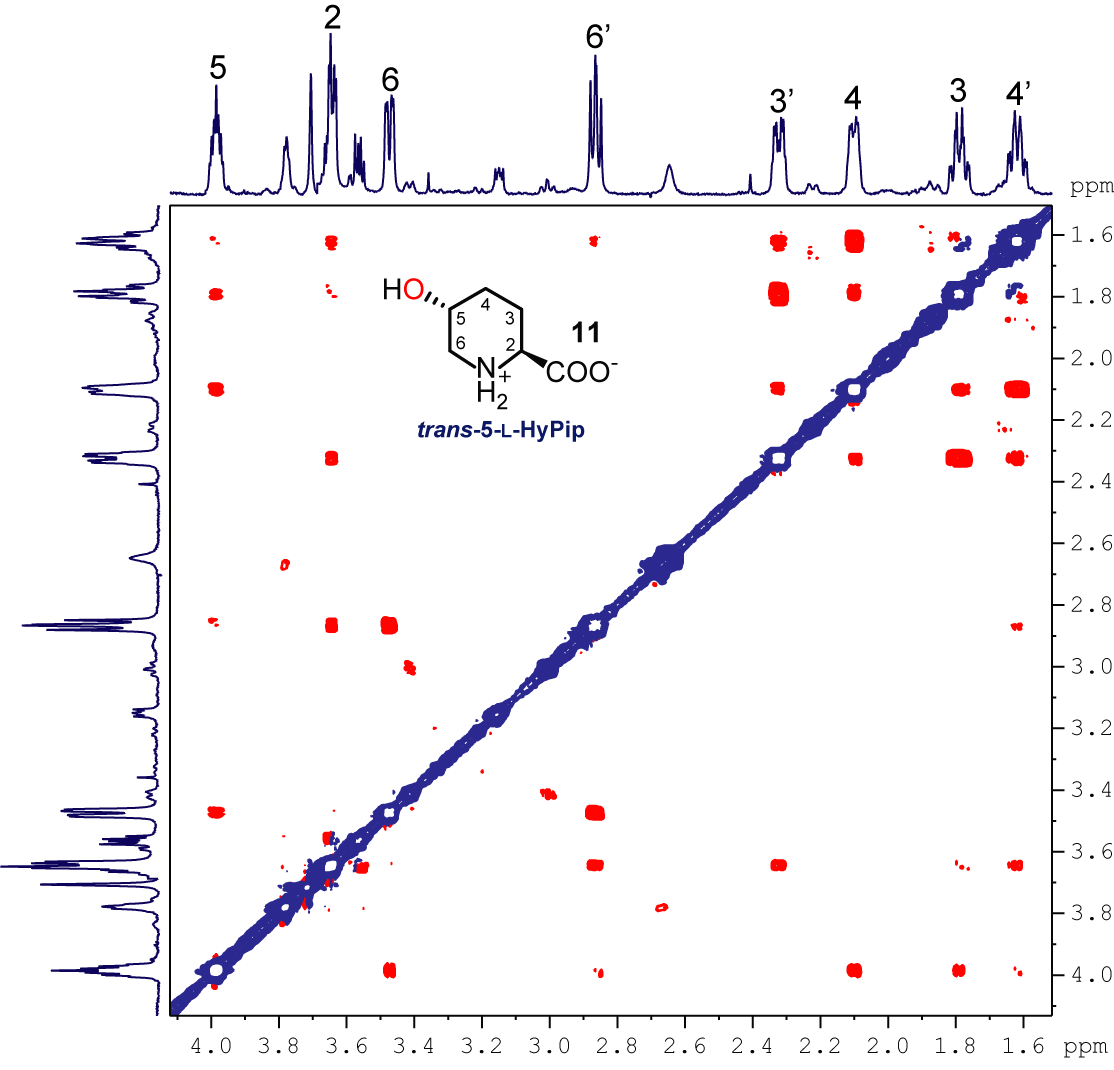


Fig. S16. 2D-NMR of the (2S,5R)-*trans*-5-hydroxy-L-pipecolic acid (11) hydroxylation product from *cis*P3H and *cis*P4H reactions using (2*S*)-L-pipecolic acid (Pip) (7):

(a) ^1^H-^1^H COSY spectrum (‘cosygpprf2qf’ pulse sequence);

(b) ^1^H-^1^H NOESY spectrum (‘noesyphprf2’ pulse sequence).

Chemical shift values are referenced to TSP-*d*_4_ (‘0.0 ppm’).

**(a)**

**(b)**


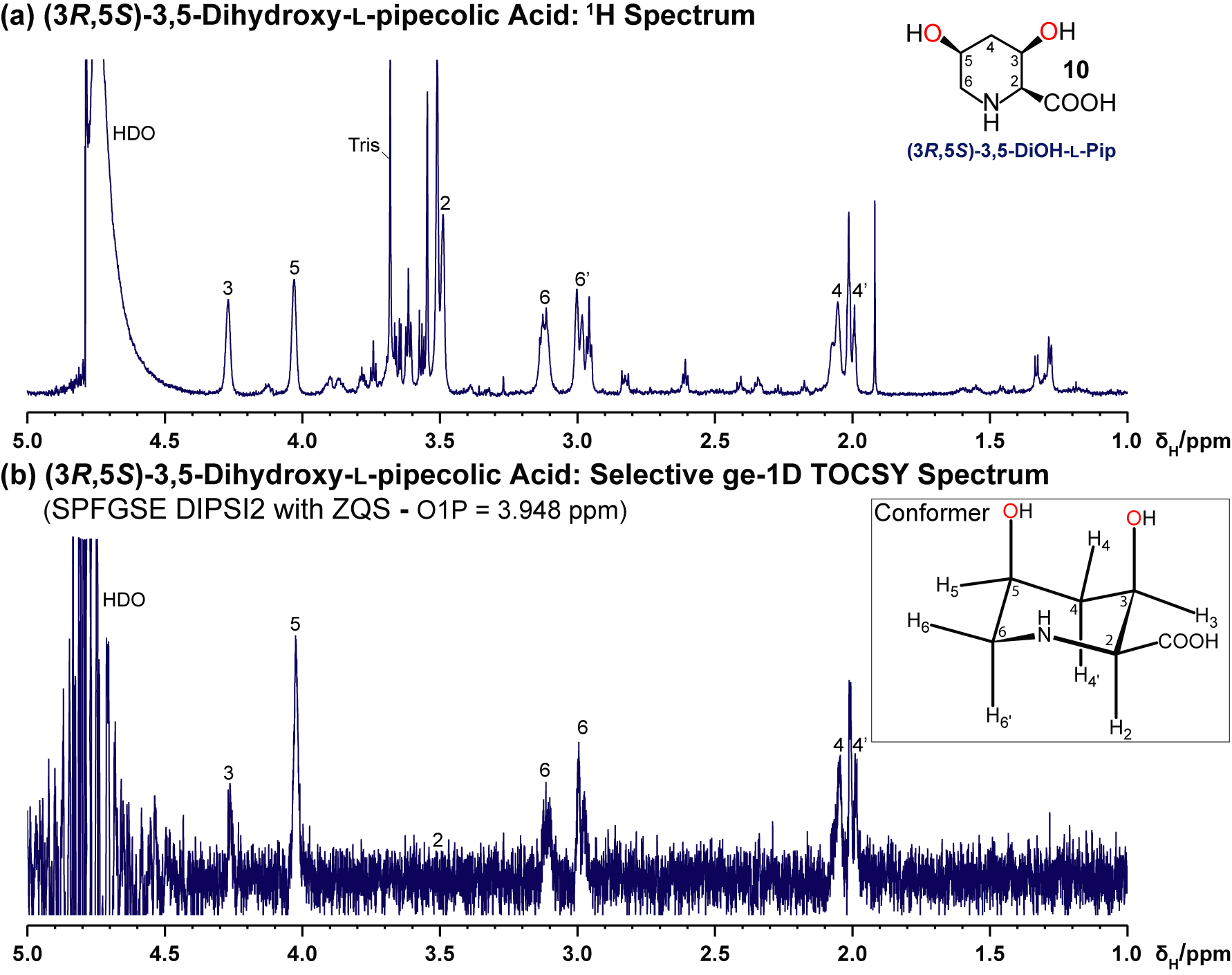


Fig. S17. NMR of the (2*S*,3*R*,5*S*)-3,5-dihydroxy-L-pipecolic acid (10) hydroxylation product from *trans*P4H reactions using (2*S*)-L-pipecolic acid (Pip) (7): (a) ^1^H-NMR spectrum (‘zgpr’ pulse sequence) and (b) selective 1D ge-TOCSY (‘spfgsedipsi2zs’ pulse sequence). Chemical shift values are referenced to TSP-*d*_4_ (‘0.0 ppm’).

Assignments

^1^H NMR (700 MHz, D_2_O) δ = 4.30 – 4.24 (m, 1H), 4.07 – 4.00 (m, 1H), 3.50 (d, *J* = 14.5 Hz, 1H), 3.13 (dd, *J* = 11.0, 5.3 Hz, 1H), 2.99 (dd, *J* = 13.8, 2.5 Hz, 1H), 2.05 (ddd, *J* = 14.2, 4.7, 2.0 Hz, 1H), 2.01 (ddd, *J* = 14.2, 3.3 Hz, 1H).


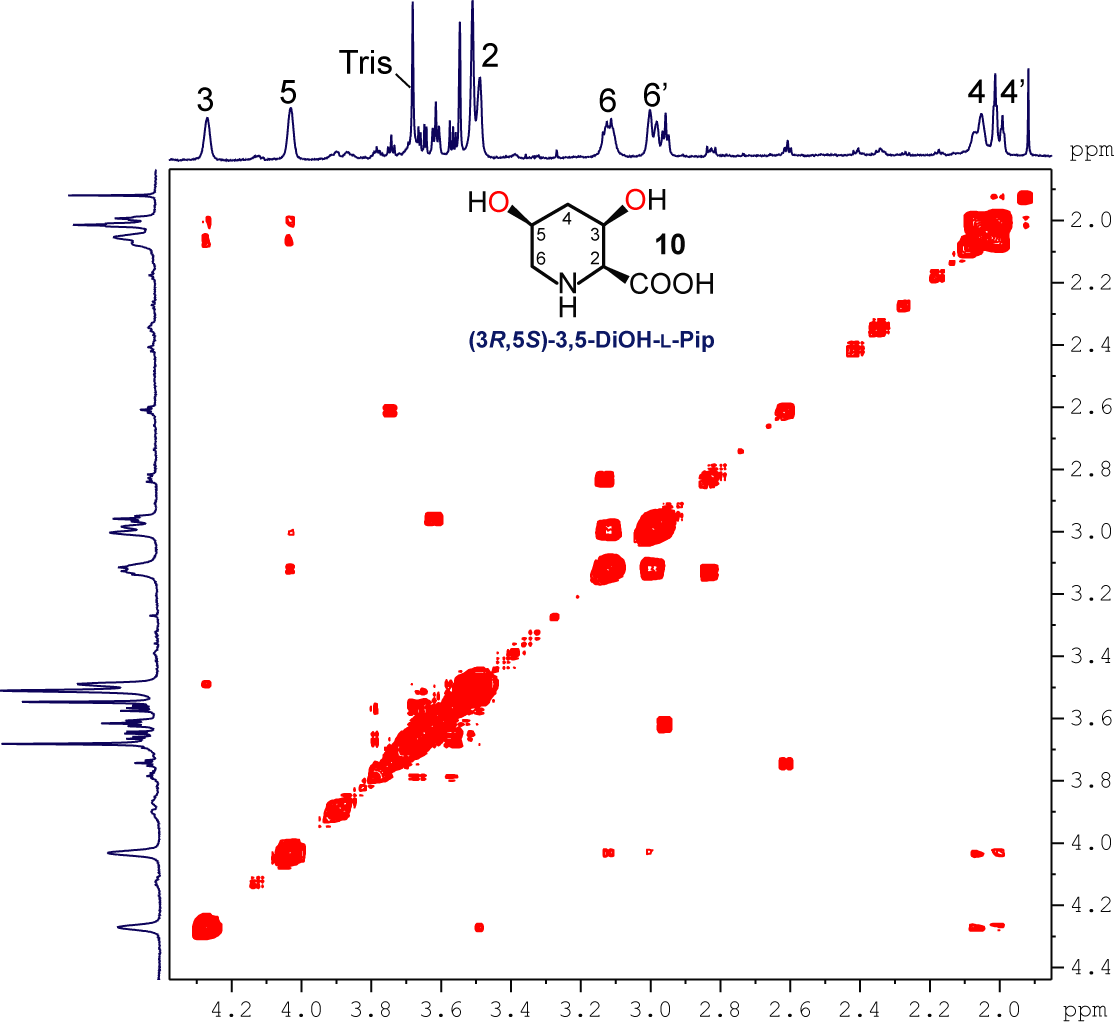


**Fig. S18.** ^1^H-^1^H**-**COSY-NMR of the dihydroxylated product from the *cis*P4H reaction with L-pipecolic acid (Pip) (**7**): NMR assigned the product as (2*S*,3*R*,5*S*)-3,5-dihydroxy-L-pipecolic acid (**10**). Chemical shift values are referenced to TSP-*d*_4_ (‘0.0 ppm’).

1. *(2S)-L-Azepane-2-carboxylic Acid (Azp) (****12****)*


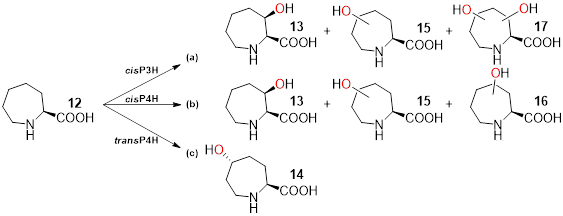


Scheme S3. Proline hydroxylase reactions using (2*S*)-L-azepane-2-carboxylic acid (Azp) (12)

(a) *cis*P3H catalyses the production of (2*S*,3*R*)-*cis*-3-hydroxy-L-azepane-2-carboxylic acid (13), hydroxyl-L-azepane-2-carboxylic acid (15), and dihydroxyl-L-azepane-2-carboxylic acid (17);

(b) *cis*P4H catalyses production of (2*S*,3*R*)-*cis*-3-hydroxy-L-azepane-2-carboxylic acid (13) and two other hydroxylation products (probably C5- and C4-hydroxy products) (16 and 15);

(c) *trans*P4H catalyses production of (2*S*,5*R*)-*trans*-5-hydroxy-L-azepane-2-carboxylic acid (**14**).

Stereochemical assignments were made by NMR.

1. *(2S)-L-Azepane-2-carboxylic acid (Azp) (Error! Reference source not found.)*


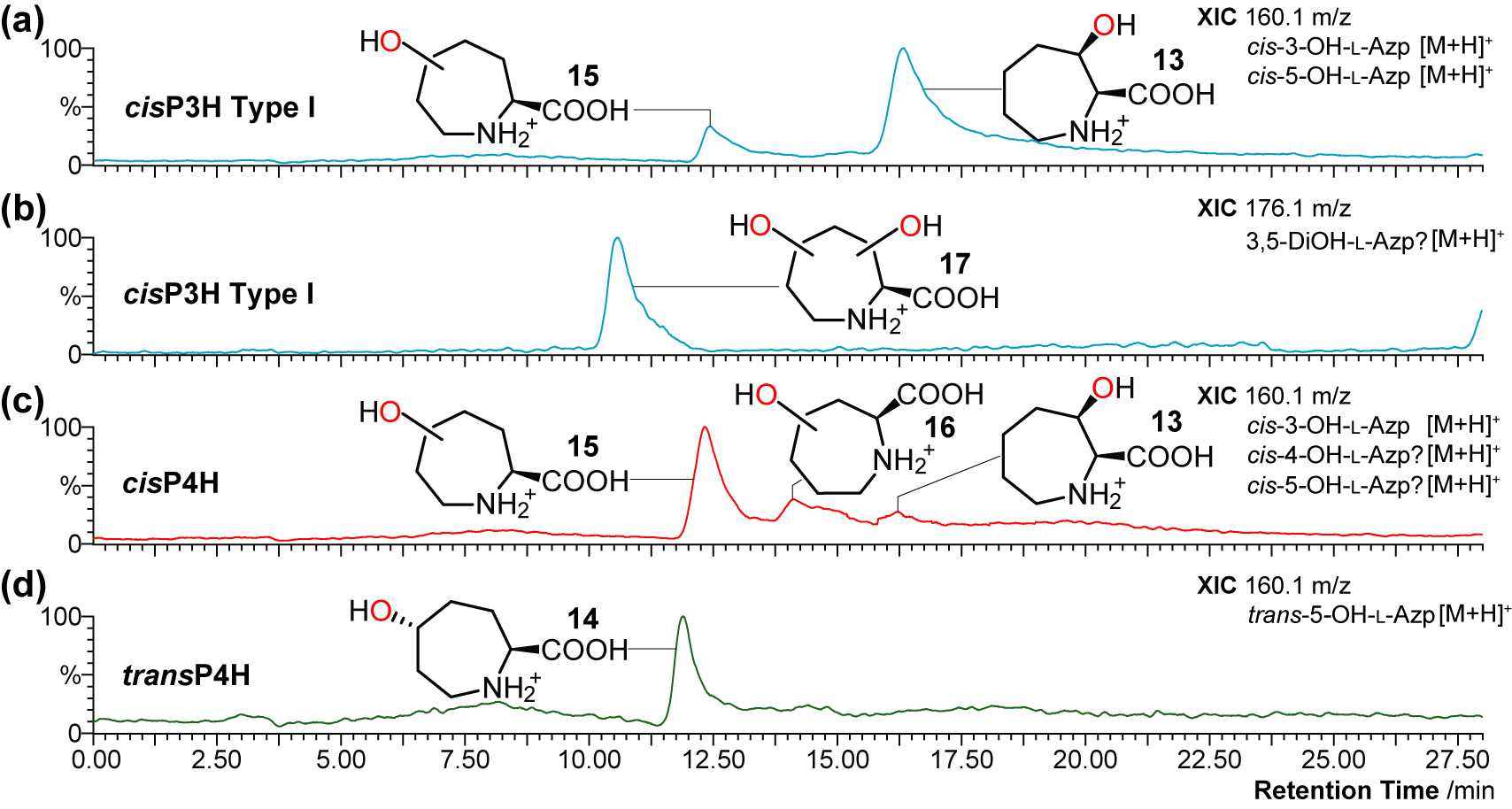


Fig. S19. Extracted-ion count (XIC) LC/MS chromatograms for the proline hydroxylase reactions using (2*S*)-L-azepane-2-carboxylic acid (Azp) (12):

(a) *cis*P3H reactions yield (2*S*,3*R*)-*cis*-3-hydroxy-L-azepane-2-carboxylic acid (13) and another hydroxylation product (15);

(b) *cis*P3H reactions yield a dihydroxylation product (**17**);

(c) *cis*P4H reactions yield (2*S*,3*R*)-*cis*-3-hydroxy-L-azepane-2-carboxylic acid (13) and two other hydroxylation products (probably C5- and C4-hydroxy products) (16 and 15);

(d) *trans*P4H reactions yield (2*S*,5*R*)-*trans*-5-hydroxy-L-azepane-2-carboxylic acid (**14**).

Stereochemical assignments were made by NMR.


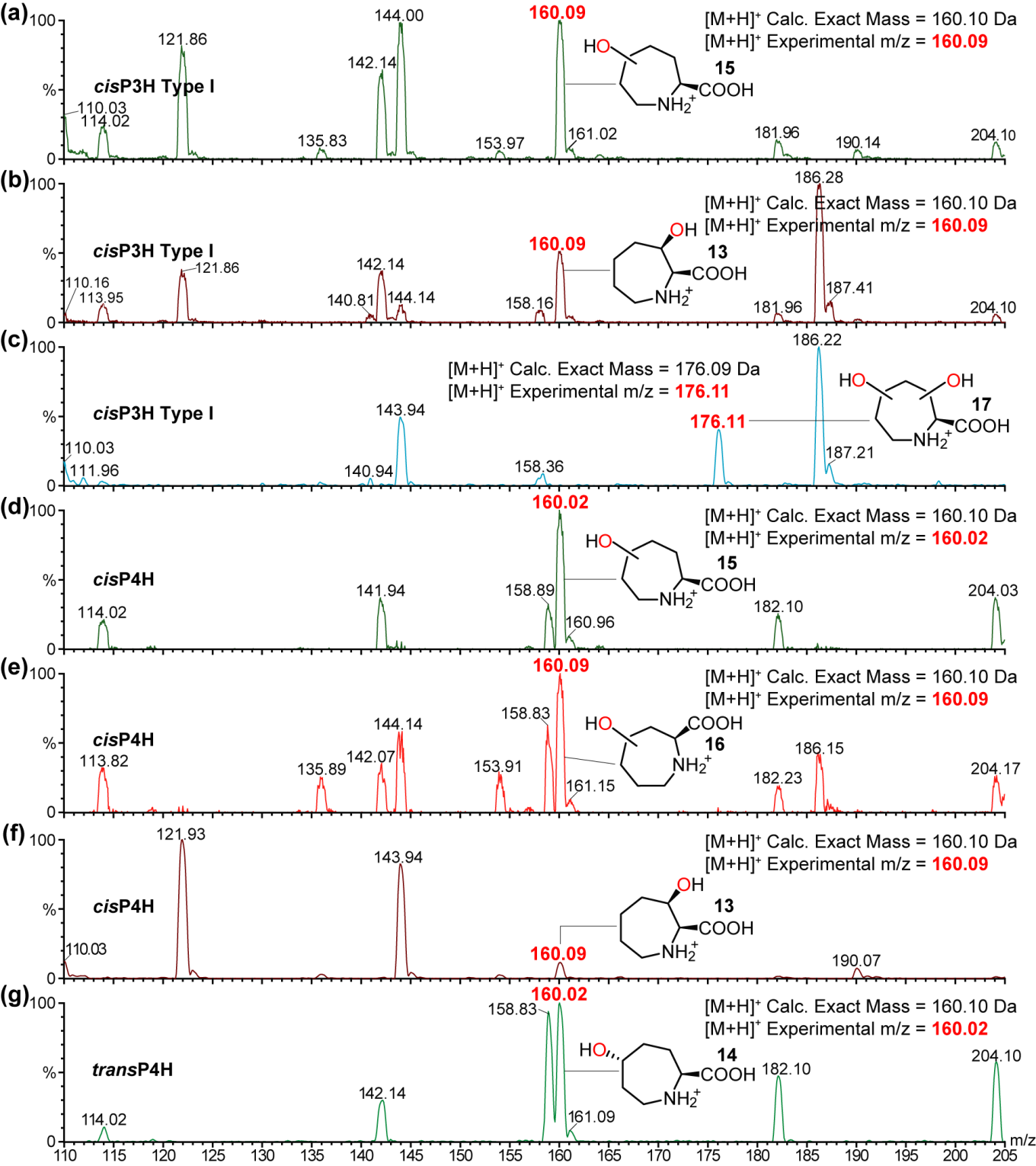


Fig. S20. LC/MS spectra of proline hydroxylase reactions using (2*S*)-L-azepane-2-carboxylic acid (Azp) (12):

(a) *cis*P3H reactions yield (2*S*,3*R*)-*cis*-3-hydroxy-L-azepane-2-carboxylic acid (13) and another hydroxylation product (15);

(b) *cis*P3H reactions yield a dihydroxylation product (**17**);

(c), (d), (e), (f) *cis*P4H reactions yield (2*S*,3*R*)-*cis*-3-hydroxy-L-azepane-2-carboxylic acid (13) and two other hydroxylation products (probably C5- and C4-hydroxy products) ) (16 and 15);

(g) *trans*P4H reactions yield (2*S*,5*R*)-*trans*-5-hydroxy-L-azepane-2-carboxylic acid (**14**).

Stereochemical assignments were made by NMR.

1. *(2S)-L-Azepane-2-carboxylic Acid (Azp) (Error! Reference source not found.)*


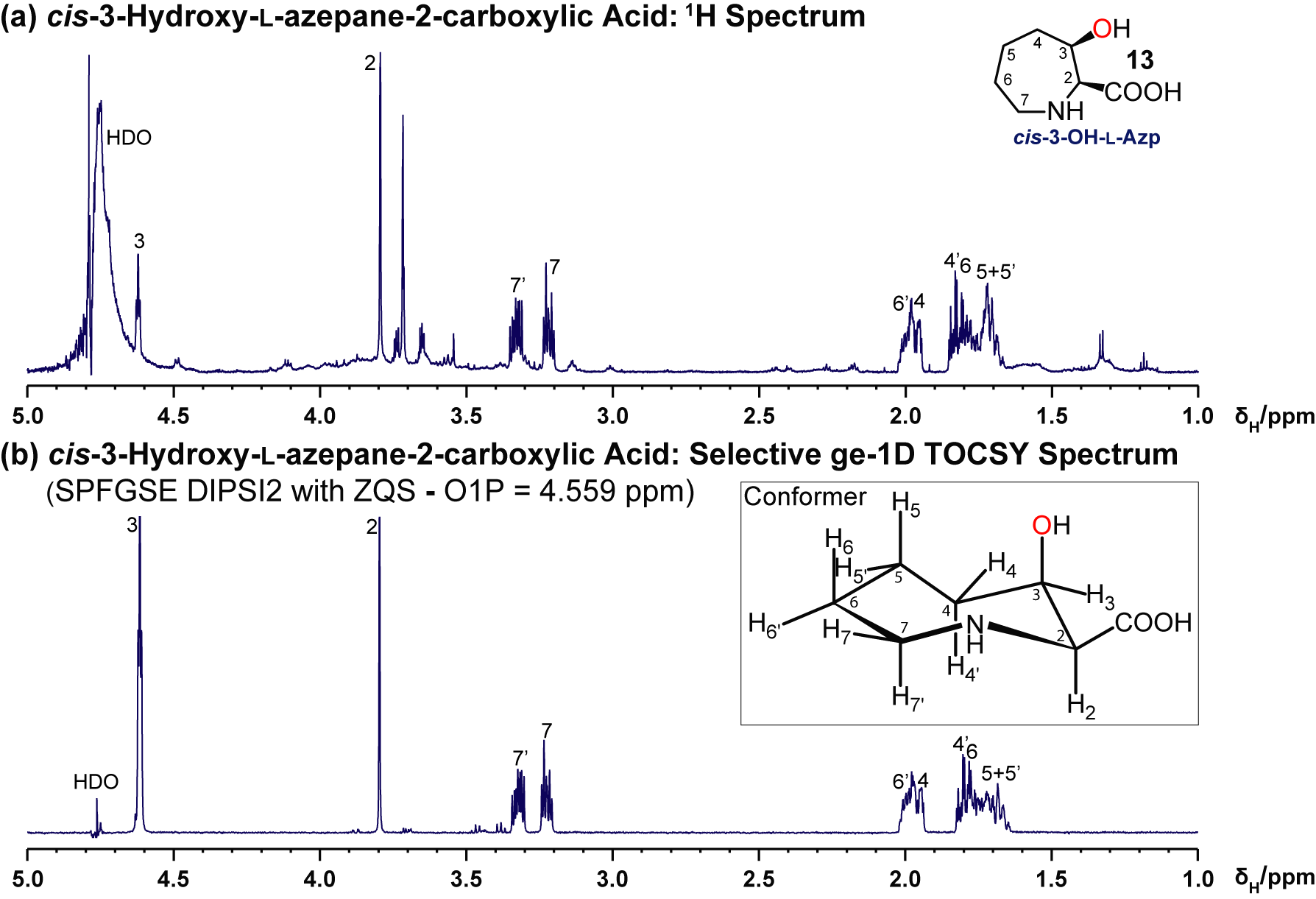


Fig. S21. NMR of the (2*S*,3*R*)-*cis*-3-hydroxy-L-azepane-2-carboxylic acid (13) hydroxylation product from *cis*P3H and *cis*P4H reactions using a (2*S*)-L-azepane-2-carboxylic acid (Azp) (12) substrate: (a) ^1^H-NMR spectrum (‘zgpr’ pulse sequence) and (b) selective 1D ge-TOCSY (‘spfgsedipsi2zs’ pulse sequence). Chemical shift values are referenced to TSP-*d*_4_ (‘0.0 ppm’).

Assignments

^1^H NMR (700 MHz, D_2_O) δ = 4.62 (ddd, *J* = 4.2, 3.9, 1.3 Hz, 1H), 3.80 (d, *J* = 1.3 Hz, 1H), 3.33 (ddd, *J* = 13.3, 9.3, 5.6 Hz, 1H), 3.22 (ddd, *J* = 13.3, 5.5, 1.2 Hz, 1H), 2.00 (ddddd, *J* = 13.6, 7.7, 5.6, 2.4, 1.2 Hz, 1H), 1.96 (dddd, *J* = 14.0, 4.7, 4.2, 2.3, 1.6 Hz, 1H), 1.83 (dddd, *J* = 14.0, 11.0, 3.9, 3.0 Hz, 1H), 1.77 (ddddd, *J* = 13.6, 9.3, 5.5, 3.1, 2.3 Hz, 1H), 1.74 – 1.66 (m, 2H).


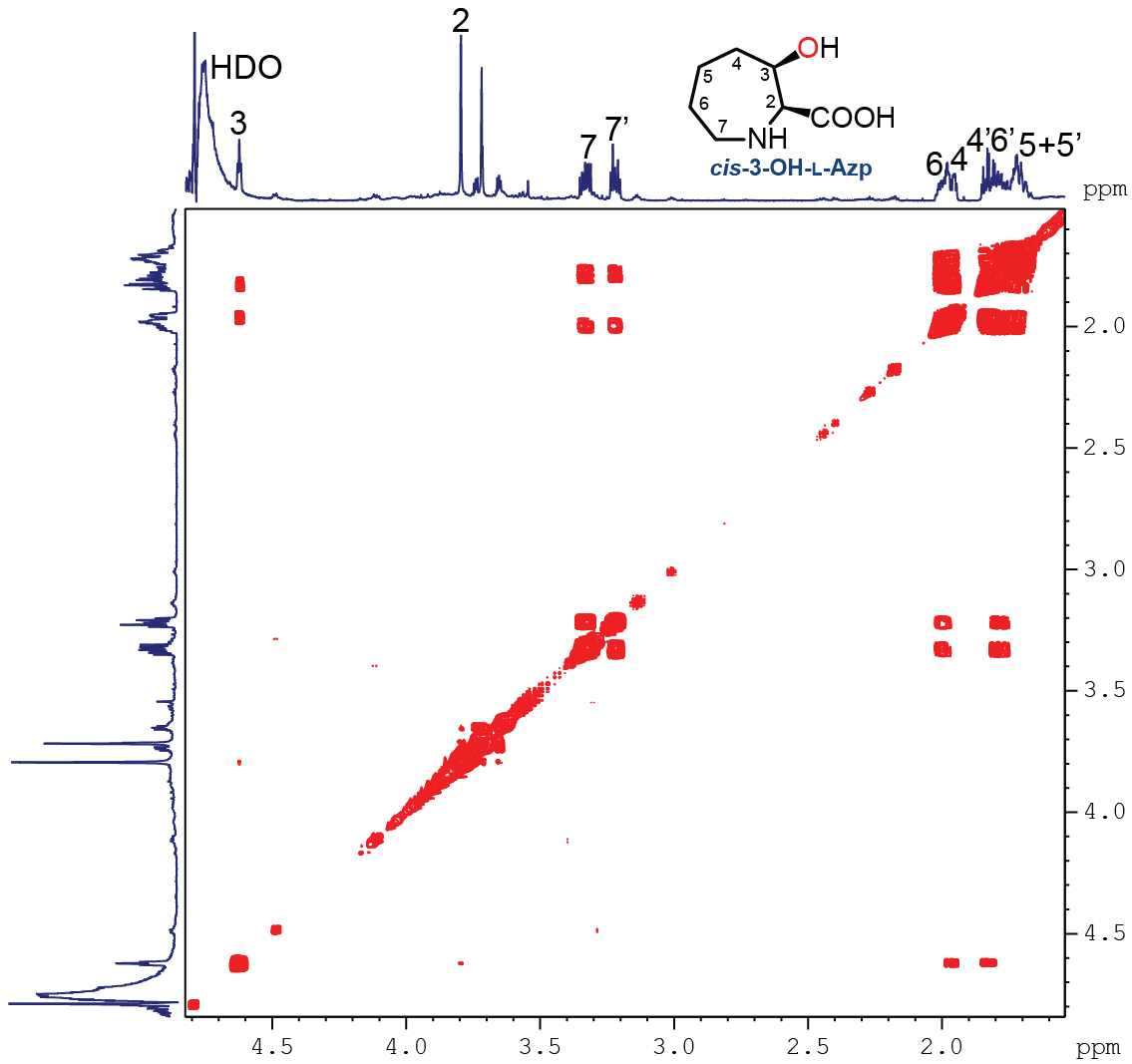


**Fig. S22** ^1^H-^1^H-COSY-NMR analysis of the hydroxylation product from the *cis*P3H reaction with (2*S*)-L-azepane-2-carboxylic acid (Azp) (**Error! Reference source not found.Error! Reference source not found.**): NMR assignments confirmed that the product is (2*S*,3*R*,5*S*)-3,5-dihydroxy-L-pipecolic acid (**Error! Reference source not found.**).

Spectra were measured at 298 K using on a 700 MHz Bruker AVIII 700 spectrometer (with a ^1^H/^13^C/^15^N TCI CryoProbe); presaturation (with O1P = 4.701 ppm) was used to suppress the broad, HDO-solvent peak. Samples were prepared in 1 mm tubes containing (16 μL) D_2_O containing 0.05% (w/v) TSP-*d*_4_ [NMR chemical shift reference (‘0.0 ppm’)/internal standard].


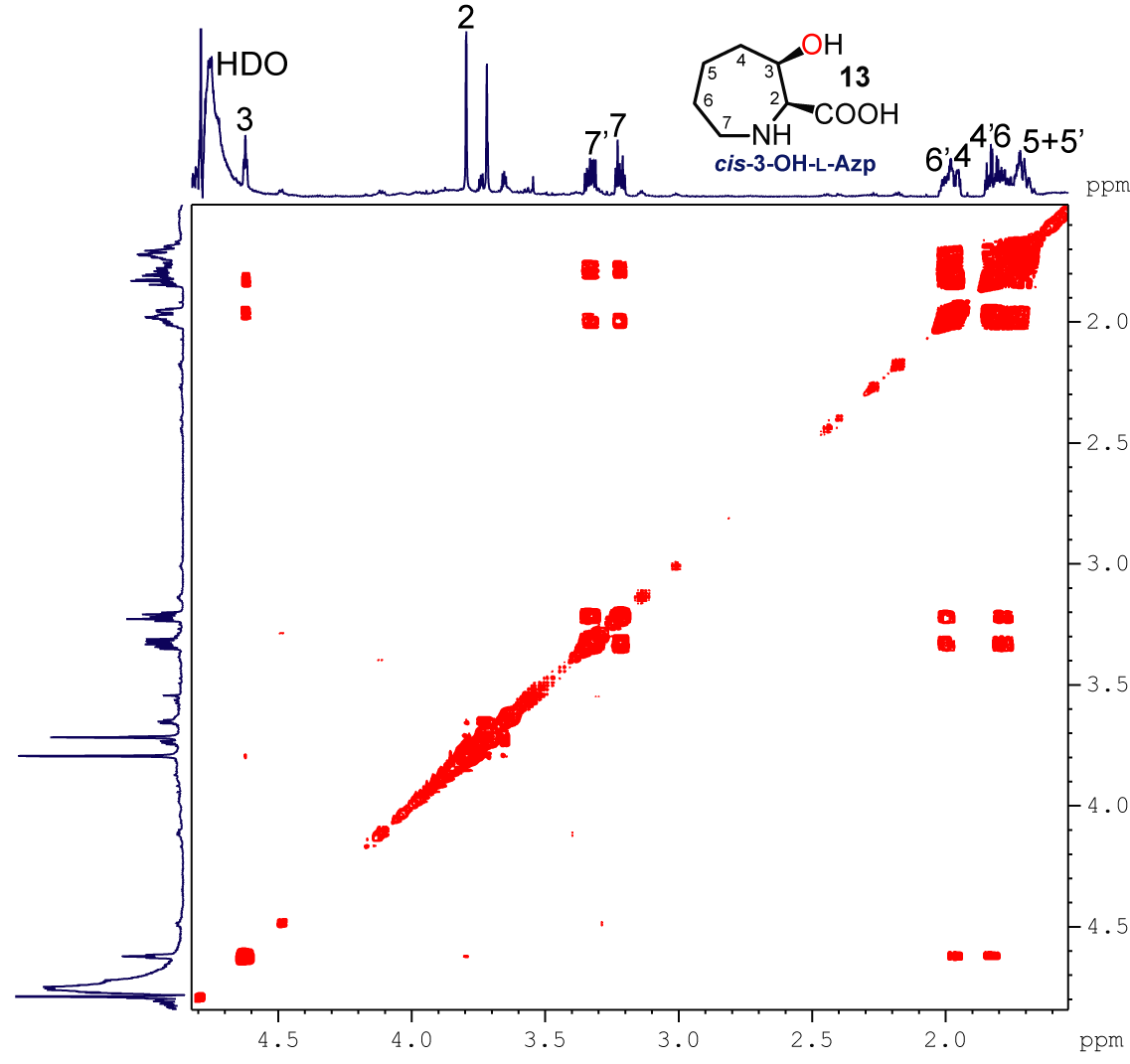


Fig. S22. ^1^H-^1^H-COSY-NMR of the hydroxylation product from the *cis*P3H reaction with (2*S*)-L-azepane-2-carboxylic acid (Azp) (12): NMR assigned product as (2*S*,3*R*)-*cis*-3-hydroxy-L-azepane-2-carboxylic acid (13). Chemical shift values are referenced to TSP-*d*_4_ (‘0.0 ppm’).


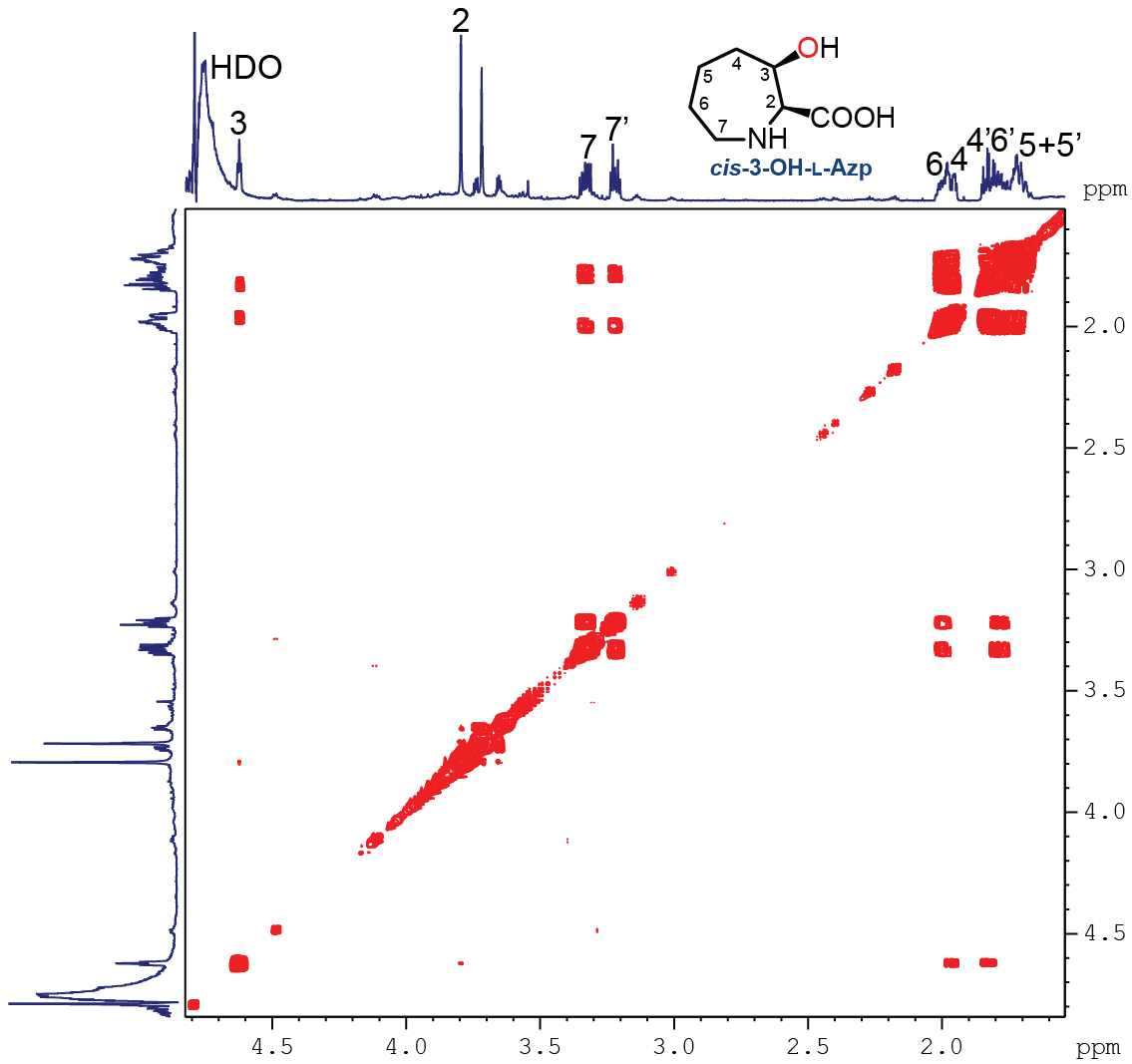


**Fig. S23** ^1^H-^1^H-COSY-NMR analysis of the hydroxylation product from the *cis*P3H reaction with (2*S*)-L-azepane-2-carboxylic acid (Azp) (**Error! Reference source not found.Error! Reference source not found.**): NMR assignments confirmed that the product is (2*S*,3*R*,5*S*)-3,5-dihydroxy-L-pipecolic acid (**Error! Reference source not found.**).

Spectra were measured at 298 K using on a 700 MHz Bruker AVIII 700 spectrometer (with a ^1^H/^13^C/^15^N TCI CryoProbe); presaturation (with O1P = 4.701 ppm) was used to suppress the broad, HDO-solvent peak. Samples were prepared in 1 mm tubes containing (16 μL) D_2_O containing 0.05% (w/v) TSP-*d*_4_ [NMR chemical shift reference (‘0.0 ppm’)/internal standard].


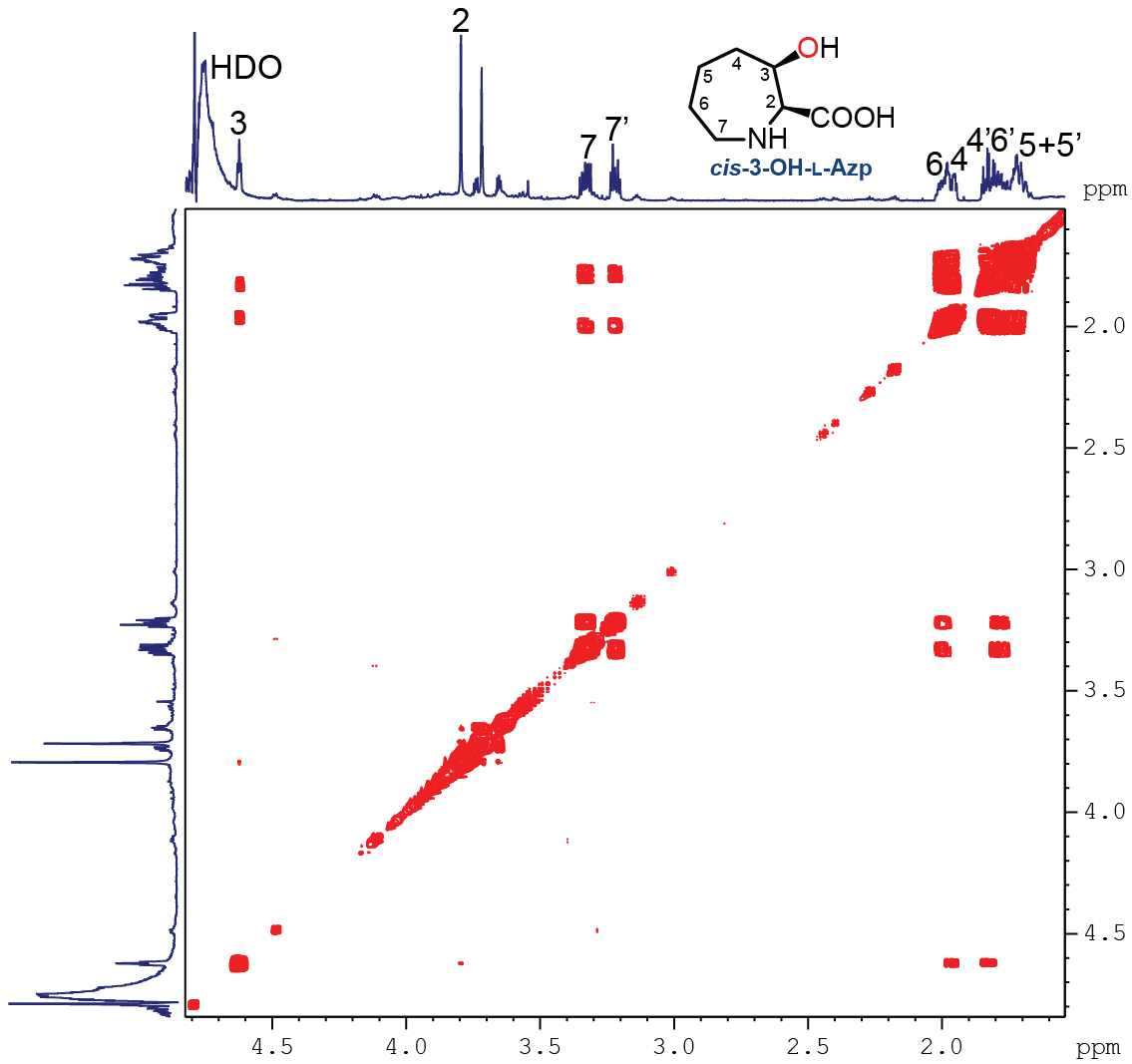


**Fig. S24** ^1^H-^1^H-COSY-NMR analysis of the hydroxylation product from the *cis*P3H reaction with (2*S*)-L-azepane-2-carboxylic acid (Azp) (**Error! Reference source not found.Error! Reference source not found.**): NMR assignments confirmed that the product is (2*S*,3*R*,5*S*)-3,5-dihydroxy-L-pipecolic acid (**Error! Reference source not found.**).

Spectra were measured at 298 K using on a 700 MHz Bruker AVIII 700 spectrometer (with a ^1^H/^13^C/^15^N TCI CryoProbe); presaturation (with O1P = 4.701 ppm) was used to suppress the broad, HDO-solvent peak. Samples were prepared in 1 mm tubes containing (16 μL) D_2_O containing 0.05% (w/v) TSP-*d*_4_ [NMR chemical shift reference (‘0.0 ppm’)/internal standard].


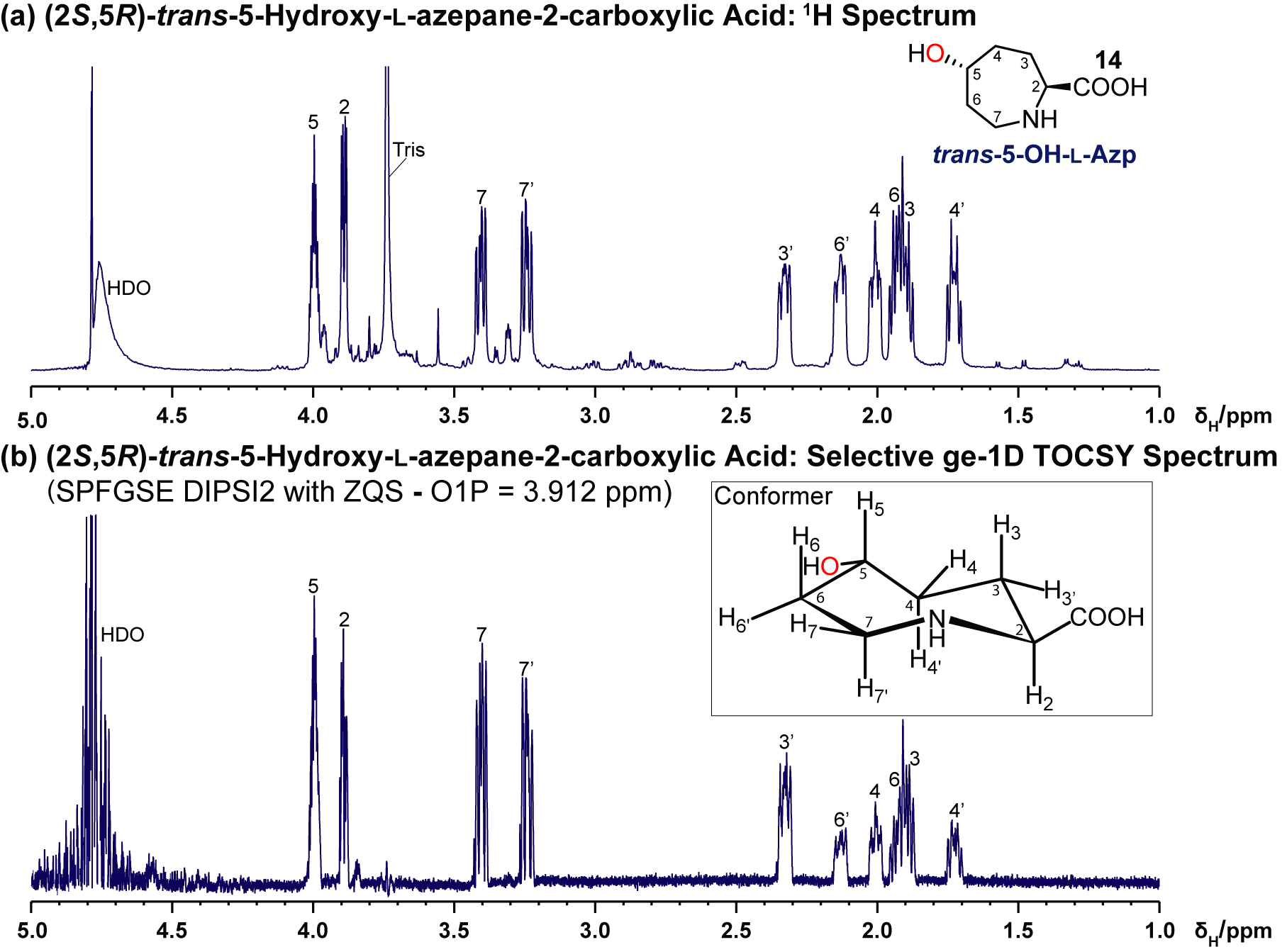


**Fig. S23.** NMR of the (2*S*,5*R*)-*trans*-5-hydroxy-L-azepane-2-carboxylic acid (**14**) hydroxylation product from *trans*P4H reactions using a (2*S*)-L-azepane-2-carboxylic acid (Azp) (**12**) substrate: (a) ^1^H-NMR spectrum (‘zgpr’ pulse sequence) and (b) selective 1D ge-TOCSY (‘spfgsedipsi2zs’ pulse sequence). Chemical shift values are referenced to TSP-*d*_4_ (‘0.0 ppm’).

Assignments

^1^H NMR (700 MHz, D_2_O) δ = 4.00 (dddd, *J* = 8.1, 7.8, 3.9, 3.9 Hz, 1H), 3.89 (dd, *J* = 8.8, 3.5 Hz, 1H), 3.41 (ddd, *J* = 14.3, 8.8, 1.7 Hz, 1H), 3.25 (ddd, *J* = 14.3, 9.3, 1.6 Hz, 1H), 2.33 (dddd, *J* = 16.0, 3.9, 3.5, 1.9 Hz, 1H), 2.13 (dddd, *J* = 15.5, 8.8, 3.9, 1.7 Hz, 1H), 2.01 (dddd, *J* = 14.3, 3.9, 3.0, 1.9 Hz, 1H), 1.94 (dddd, *J* = 15.5, 9.3, 7.8, 1.6 Hz, 1H), 1.89 (dddd, *J* = 16.0, 8.8, 8.4, 3.0 Hz, 1H), 1.73 (dddd, *J* = 14.3, 8.4, 8.1, 3.9 Hz, 1H).


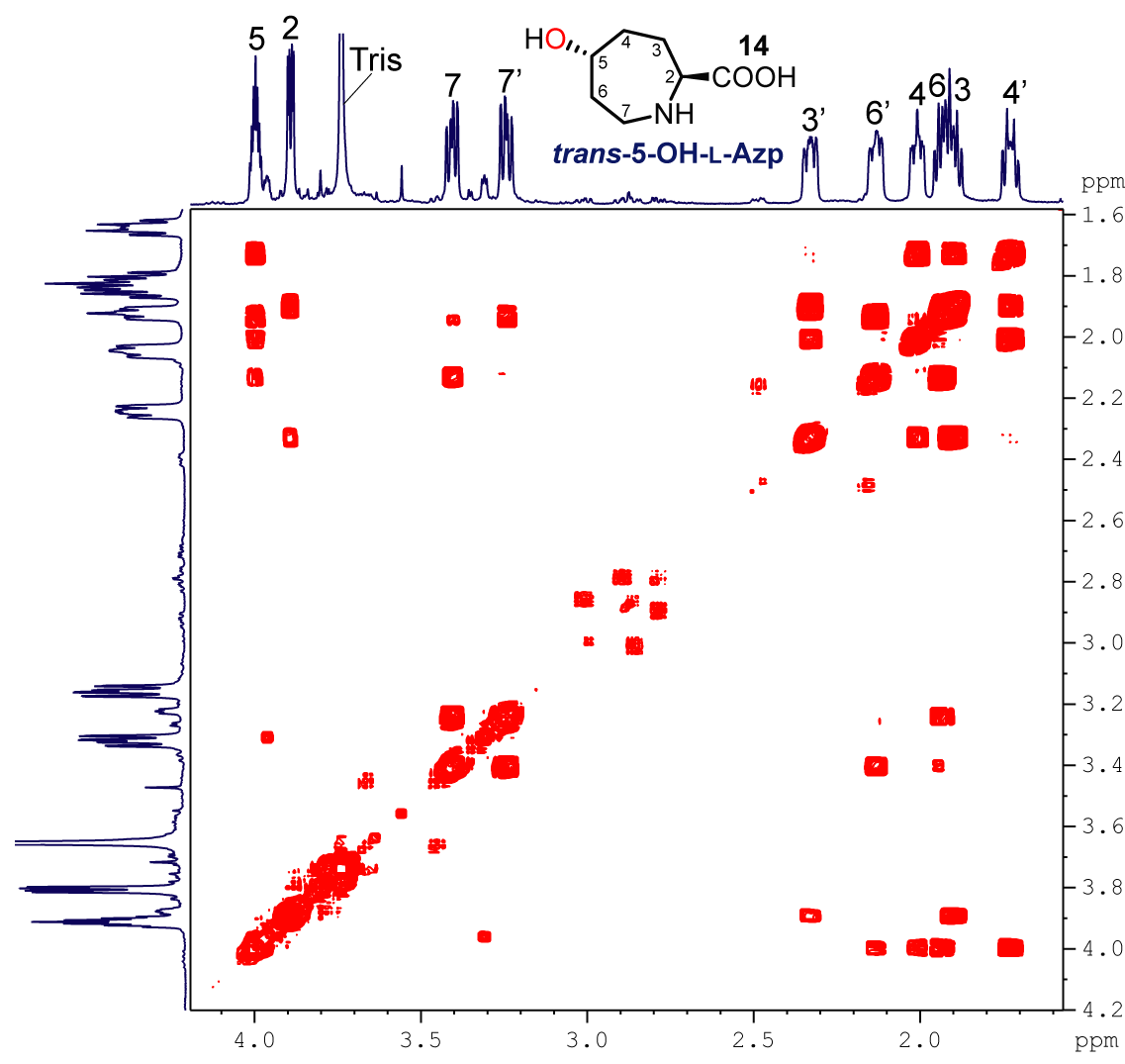


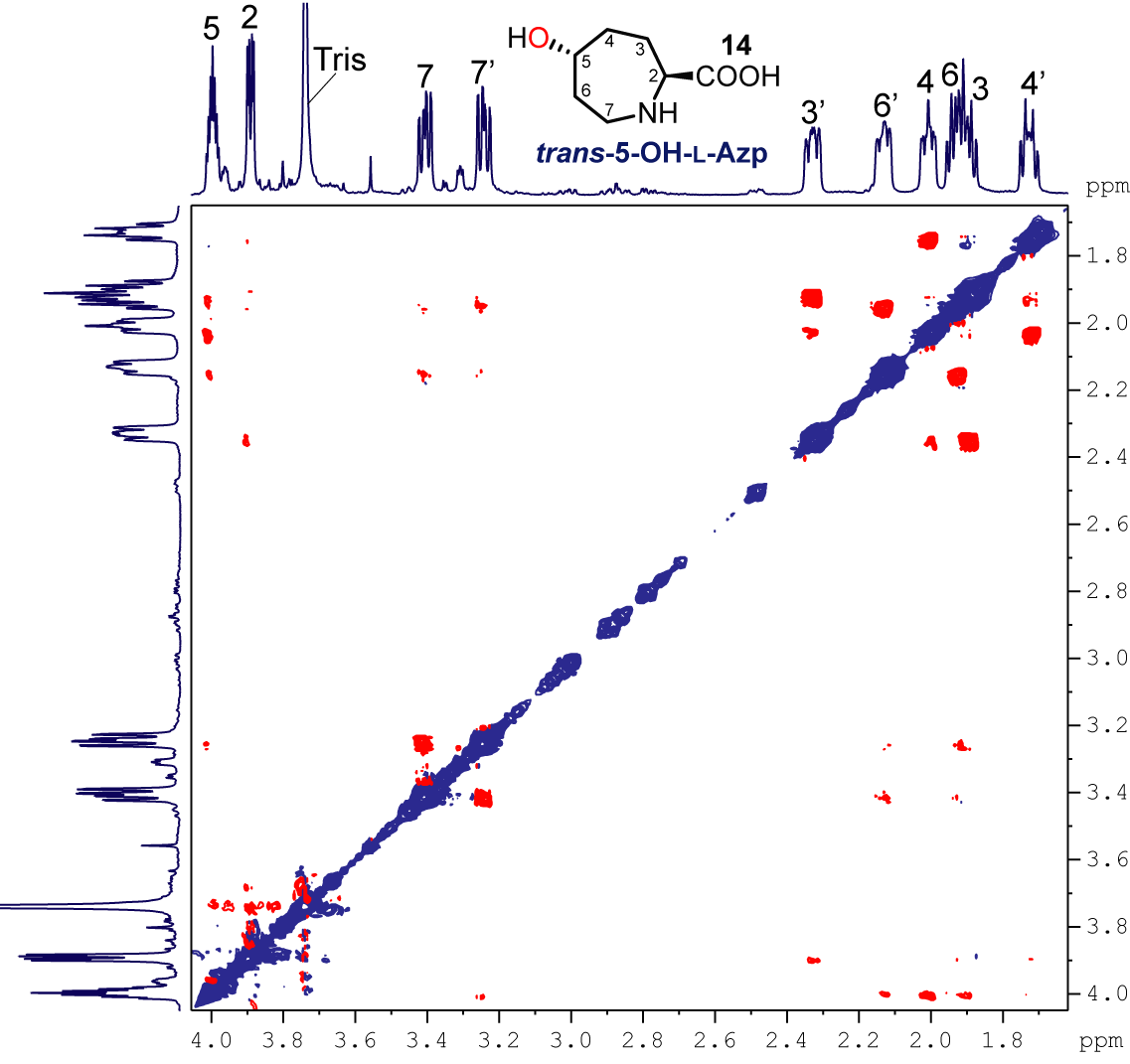


**Fig. S24.** 2D-NMR of the (2*S*,5*R*)-*trans*-5-hydroxy-L-azepane-2-carboxylic acid (**14**) hydroxylation product from *cis*P3H and *cis*P4H reactions using (2*S*)-L-azepane-2-carboxylic acid (Azp) (**12**):

(a) ^1^H-^1^H COSY spectrum (‘cosygpprf2qf’ pulse sequence);

(b) ^1^H-^1^H NOESY spectrum (‘noesyphprf2’ pulse sequence).

Chemical shift values are referenced to TSP-*d*_4_ (‘0.0 ppm’).

**(b)**

**(a)**

## N-Methylated Substrate Analogues

1. *(2S)-N-Methyl-L-proline (N-Me-Pro) (****18****)*

Scheme S4. Proline hydroxylase reactions using (2*S*)-*N*-methyl-L-proline (*N*-Me-Pro) (18):

(a) *cis*P3H catalyses production of (2*S*,3*R*)-*cis*-3-hydroxy-*N*-methyl-L-proline (19);

(b) *cis*P4H catalyses production of (2*S*,4*S*)-*cis*-4-hydroxy-*N*-methyl-L-proline (20);

(c) *trans*P4H catalyses production of a hydroxyl-*N*-methyl-L-proline [proposed to be (2*S*,4*R*)-*trans*-4-hydroxy-*N*-methyl-L-proline (**21**)] and (2*S*,4*S*)-*cis*-4-hydroxy-*N*-methyl-L-proline (**20**).


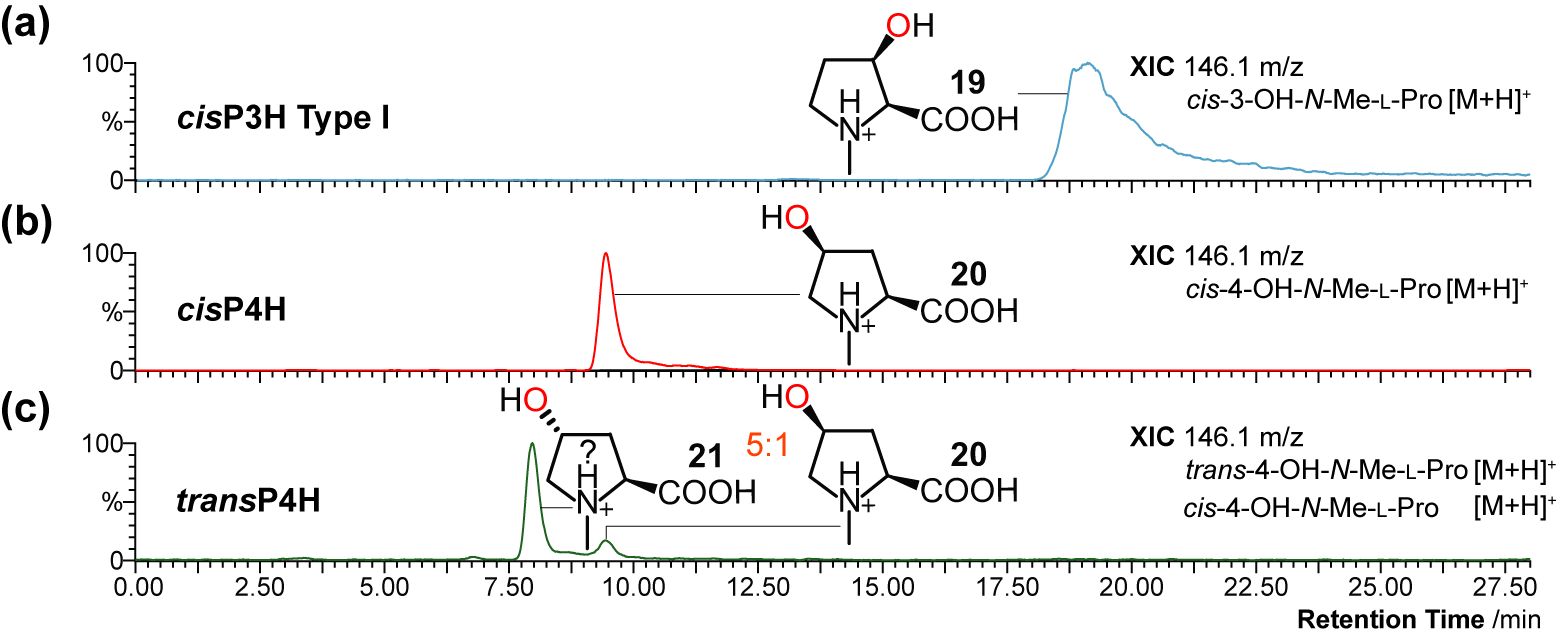


Fig. S25. Extracted-ion count (XIC) LC/MS chromatograms for the proline hydroxylase reactions using (2*S*)-*N*-methyl-L-proline (*N*-Me-Pro) (18):

(a) *cis*P3H reactions yield (2*S*,3*R*)-*cis*-3-hydroxy-*N*-methyl-L-proline (19);

(b) *cis*P4H reactions yield (2*S*,4*S*)-*cis*-4-hydroxy-*N*-methyl-L-proline (20);

(c) *trans*P4H reactions yield a hydroxyl-*N*-methyl-L-proline [proposed to be (2*S*,4*R*)-*trans*-4-hydroxy-*N*-methyl-L-proline (21)] and (2*S*,4*S*)-*cis*-4-hydroxy-*N*-methyl-L-proline (20).

Stereochemical assignments were made by NMR.


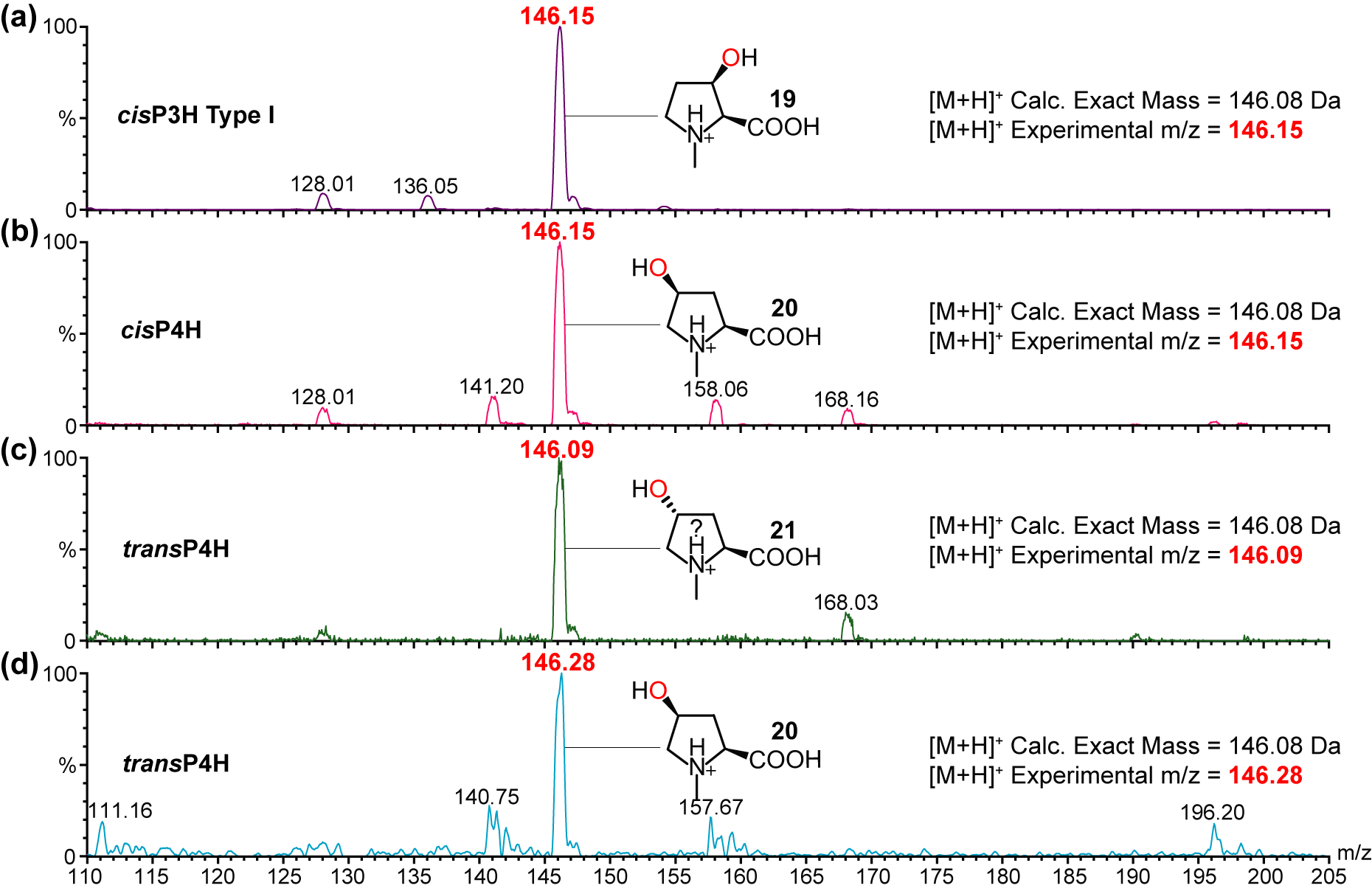


Fig. S26. LC/MS spectra of proline hydroxylase reactions using a (2*S*)-*N*-methyl-L-proline (*N*-Me-Pro) (18) substrate:

(a) *cis*P3H reactions yield (2*S*,3*R*)-*cis*-3-hydroxy-*N*-methyl-L-proline (19);

(b) *cis*P4H reactions yield (2*S*,4*S*)-*cis*-4-hydroxy-*N*-methyl-L-proline (20);

(c) *trans*P4H reactions yield a hydroxyl-*N*-methyl-L-proline [proposed to be (2*S*,4*R*)-*trans*-4-hydroxy-*N*-methyl-L-proline (21)];

(d) *trans*P4H reactions yield (2*S*,4*S*)-*cis*-4-hydroxy-*N*-methyl-L-proline (20).

Stereochemical assignments were made by NMR.


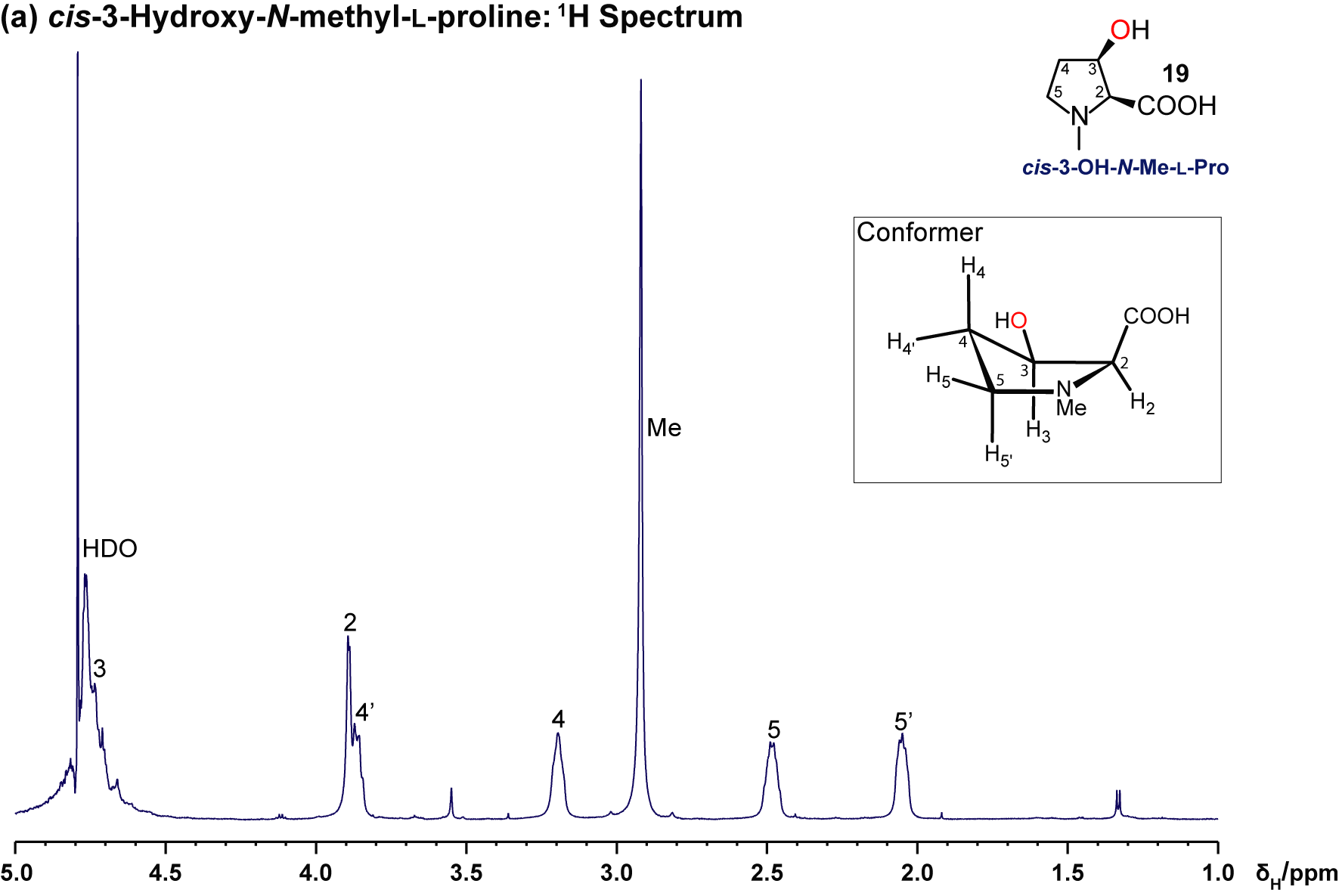


Fig. S27. NMR of the (2*S*,3*R*)-*cis*-3-hydroxy-*N*-methyl-L-proline (19) hydroxylation product from *cis*P3H reactions using (2*S*)-*N*-methyl-L-proline (*N*-Me-Pro) (18): ^1^H-NMR spectrum (‘zgpr’ pulse sequence). Chemical shift values are referenced to TSP-*d*_4_ (‘0.0 ppm’).

Assignments

^1^H NMR (700 MHz, D_2_O) δ = 4.74 – 4.73 (m, 1H), 3.89 (d, *J* = 4.5 Hz, 1H), 3.88 – 3.83 (m, 1H), 3.23 – 3.16 (m, 1H), 2.92 (s, 3H), 2.52 – 2.44 (m, 1H), 2.09 – 2.01 (m, 1H).


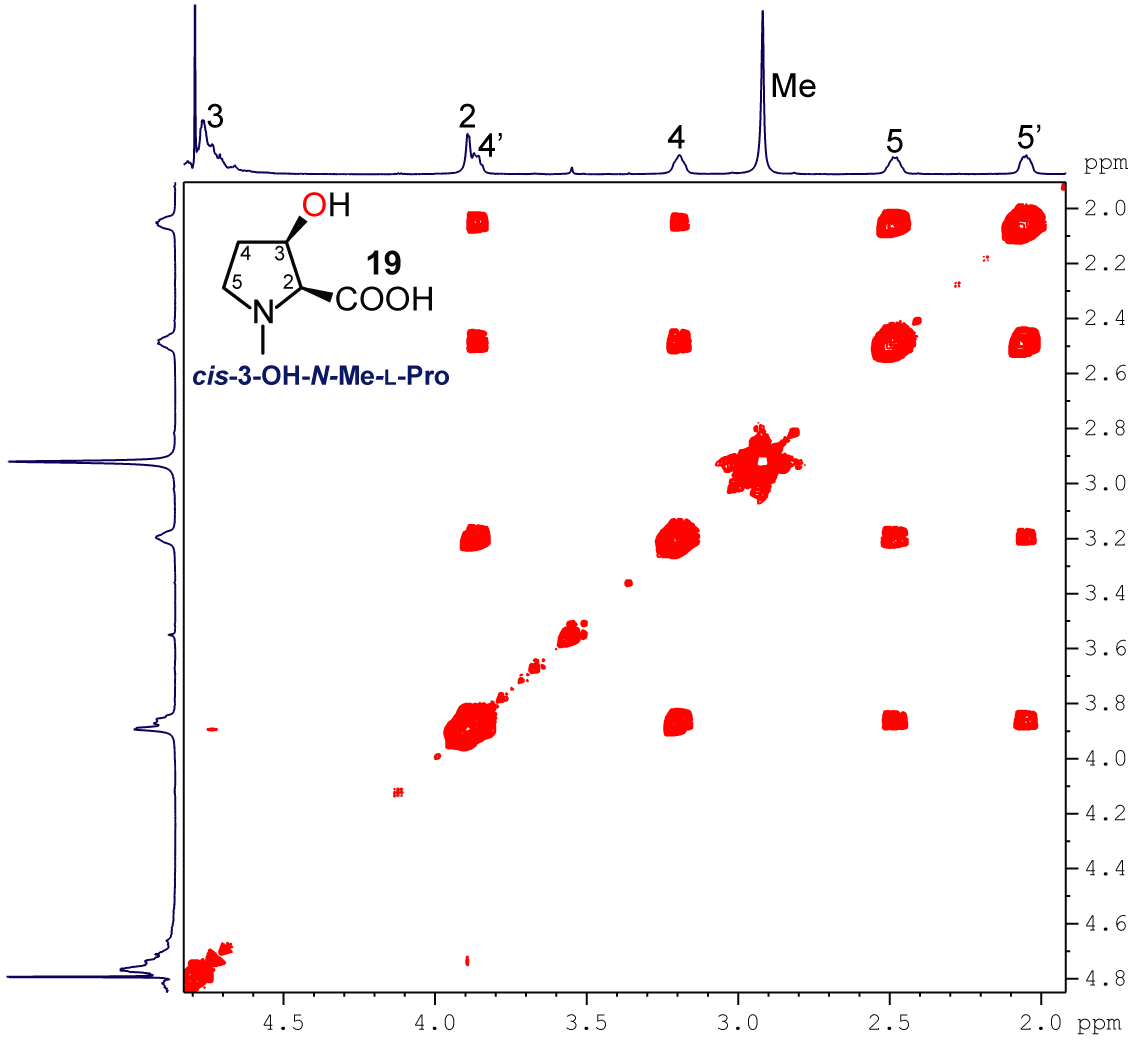


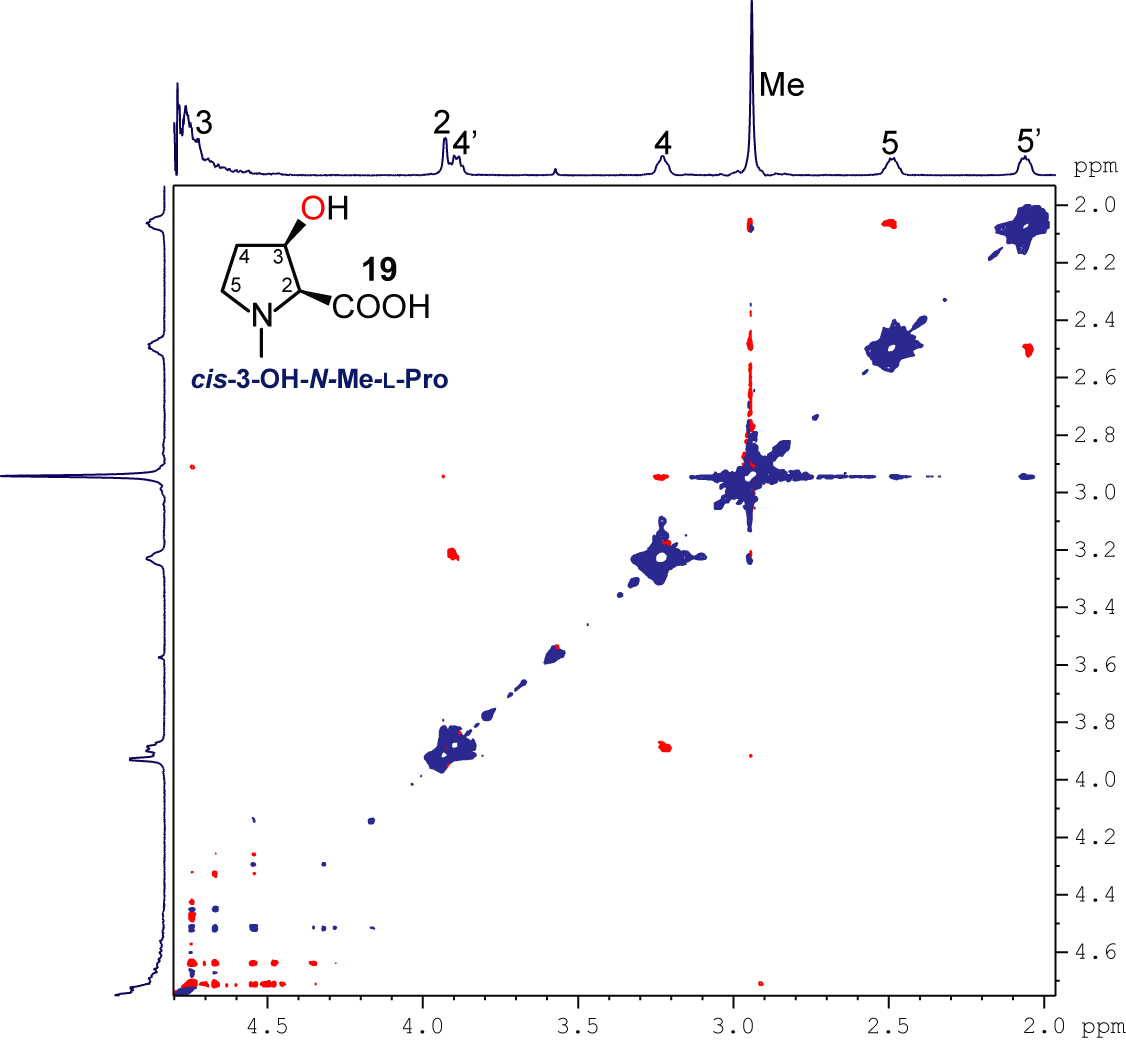


Fig. S28. 2D-NMR of the (2*S*,3*R*)-*cis*-3-hydroxy-*N*-methyl-L-proline (19) hydroxylation product from *cis*P3H reactions using (2*S*)-*N*-methyl-L-proline (*N*-Me-Pro) (18):

(a) ^1^H-^1^H COSY spectrum (‘cosygpprf2qf’ pulse sequence);

(b) ^1^H-^1^H NOESY spectrum (‘noesyphprf2’ pulse sequence).

Chemical shift values are referenced to TSP-*d*_4_ (‘0.0 ppm’).

**(a)**

**(b)**


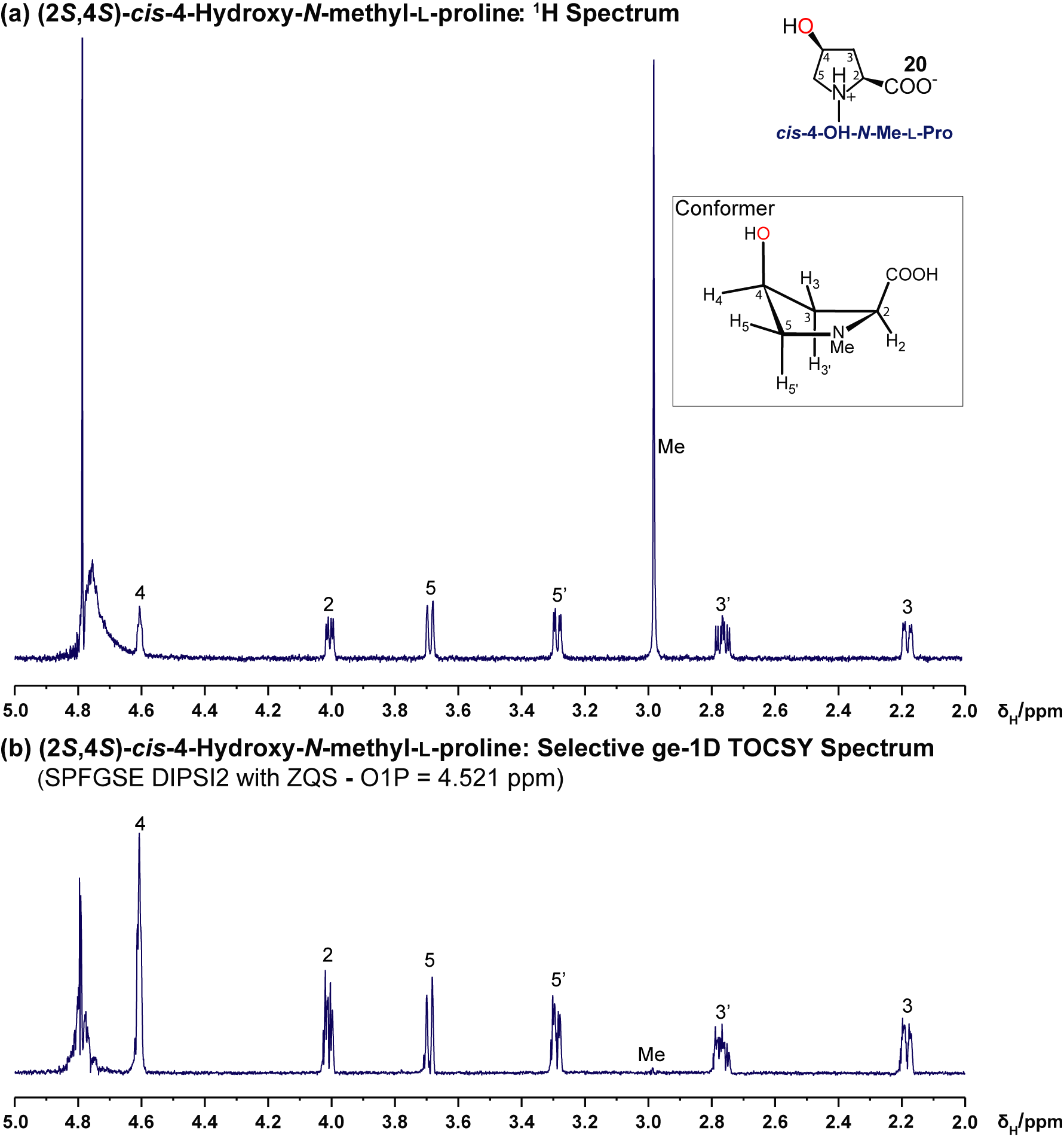


Fig. S29. NMR of the (2*S*,4*S*)-*cis*-4-hydroxy-*N*-methyl-L-proline (20) hydroxylation product from *cis*P4H reactions using (2*S*)-*N*-methyl-L-proline (*N*-Me-Pro) (18): (a) ^1^H-NMR spectrum (‘zgpr’ pulse sequence) and (b) selective 1D ge-TOCSY (‘spfgsedipsi2zs’ pulse sequence). Chemical shift values are referenced to TSP-*d*_4_ (‘0.0 ppm’).

Assignments

^1^H NMR (700 MHz, D_2_O) δ = 4.61 (dddd, *J* = 4.9, 3.7, 1.9, 1.1 Hz, 1H), 4.01 (dd, *J* = 11.2, 4.7 Hz, 1H), 3.69 (dd, *J* = 12.2, 1.1 Hz, 1H), 3.29 (dd, *J* = 12.2, 3.7 Hz, 1H), 2.98 (s, 3H), 2.77 (ddd, *J* = 14.3, 11.2, 4.9 Hz, 1H), 2.18 (ddd, *J* = 14.3, 6.6, 4.7, 1.9 Hz, 1H).


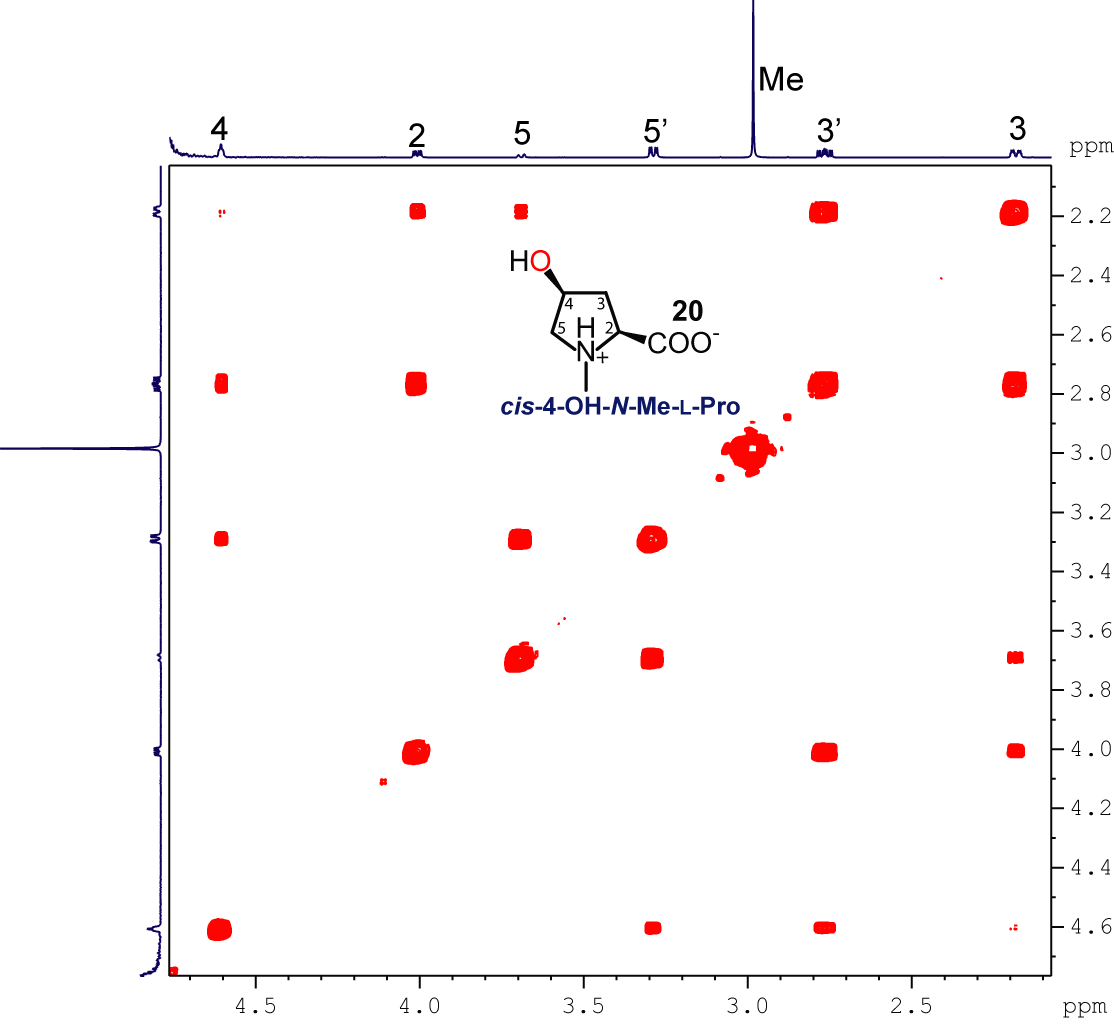


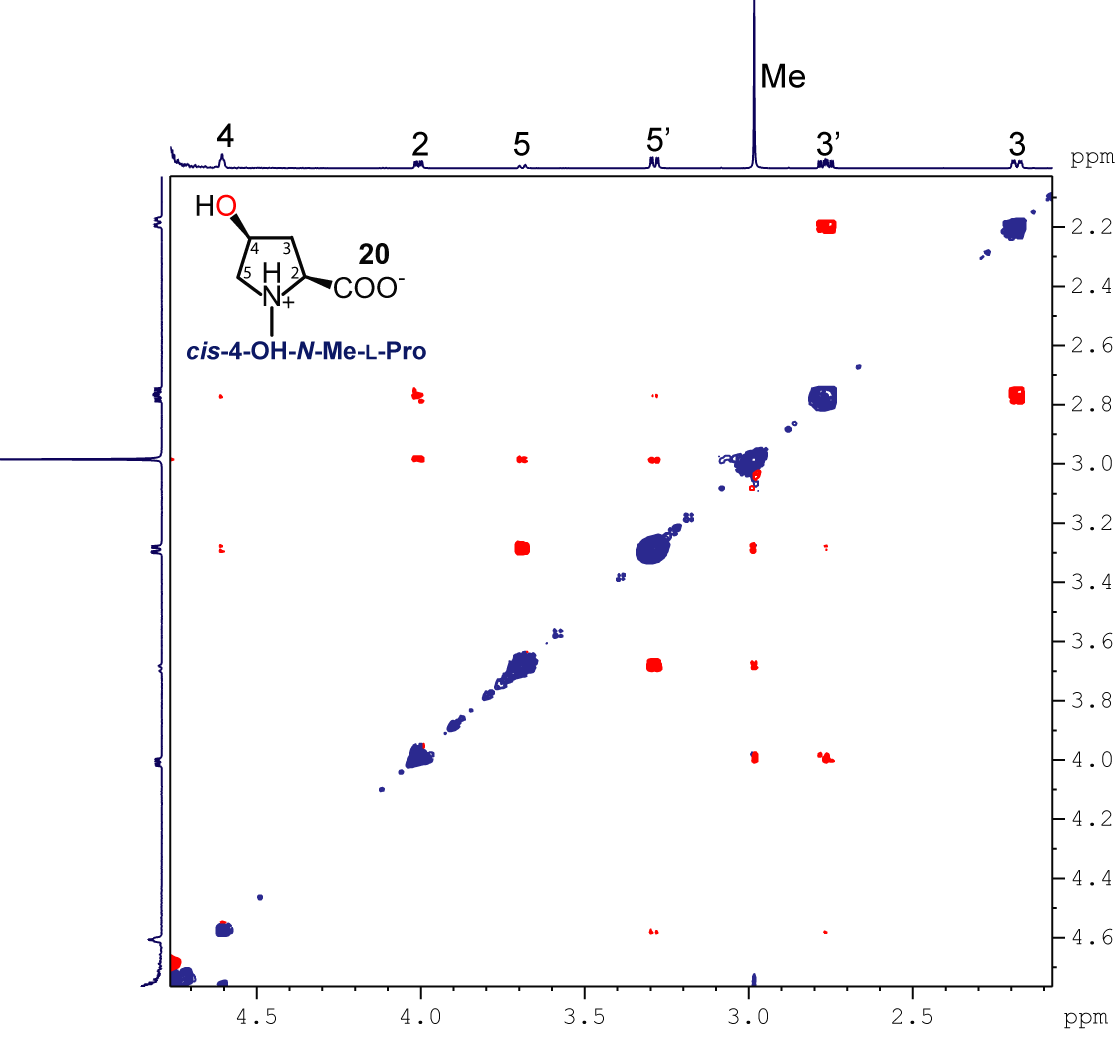


Fig. S30. 2D-NMR of the (2*S*,4*S*)-*cis*-4-hydroxy-*N*-methyl-L-proline (20) hydroxylation product from *cis*P4H reactions using (2*S*)-*N*-methyl-L-proline (*N*-Me-Pro) (18):

(a) ^1^H-^1^H COSY spectrum (‘cosygpprf2qf’ pulse sequence);

(b) ^1^H-^1^H NOESY spectrum (‘noesyphprf2’ pulse sequence).

Chemical shift values are referenced to TSP-*d*_4_ (‘0.0 ppm’).

***Repurify transP4H major product spectra? – 1H, TOCSY, COSY + NOESY ideally

**(a)**

**(b)**

1. *(2S)-N-Methyl-L-pipecolic Acid (N-Me-Pip) (****22****)*

Scheme S5. Proline hydroxylase reactions using (2*S*)-*N*-methyl-L-pipecolic acid (*N*-Me-Pip) (22):

(a) *cis*P3H catalyses production of (2*S*,3*R*)-*cis*-3-hydroxy-*N*-methyl-L-pipecolic acid (23) and a dihydroxy-*N*-methyl-L-pipecolic acid [proposed to be (2*S*,3*R*,5*S*)-dihydroxy-L-pipecolic acid (26)];

(b) *cis*P4H catalyses production of (23) and (2*S*,5*S*)-*cis*-5-hydroxy-*N*-methyl-L-pipecolic acid (24);

(c) *trans*P4H catalyses production of (2*S*,5*R*)-*trans*-5-hydroxy-*N*-methyl-L-pipecolic acid (**25**).


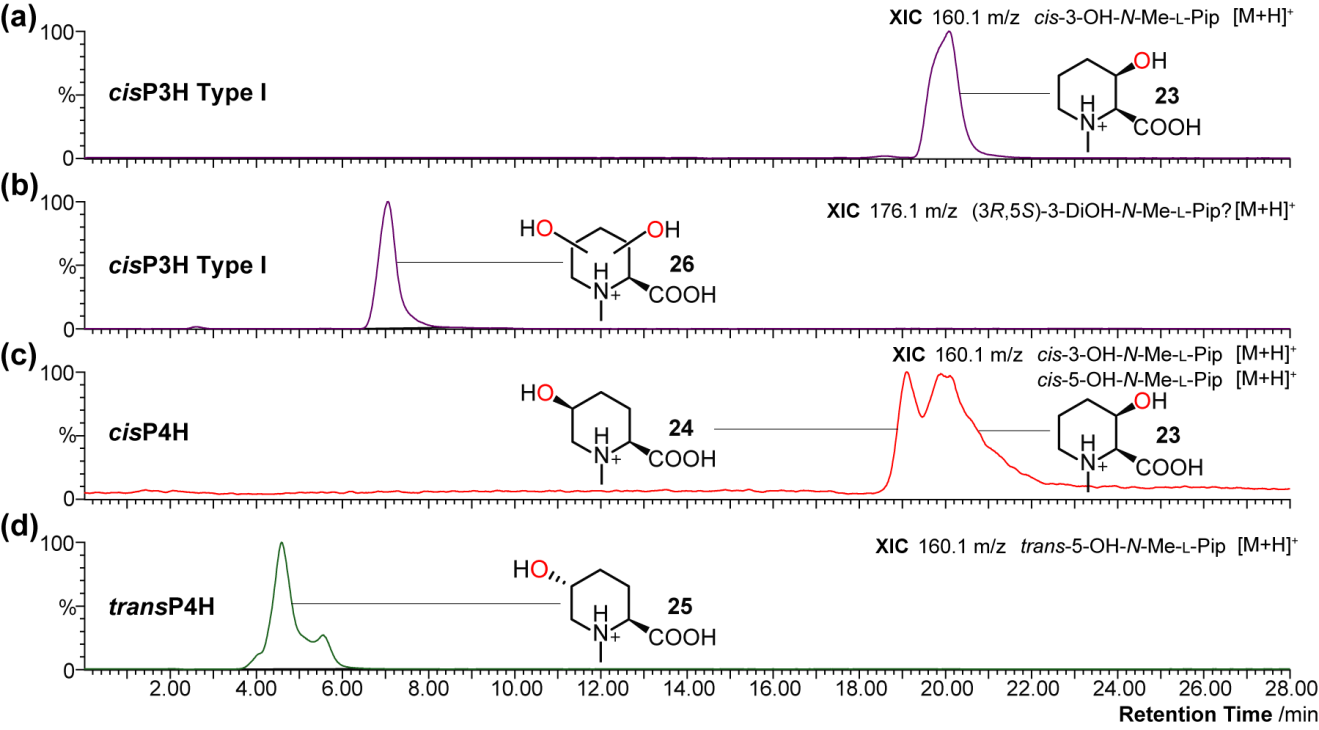


Fig. S31. Extracted-ion count (XIC) LC/MS chromatograms for the proline hydroxylase reactions using (2*S*)-*N*-methyl-L-pipecolic acid (*N*-Me-Pip) (22):

(a) *cis*P3H yields (2*S*,3*R*)-*cis*-3-hydroxy-*N*-methyl-L-pipecolic acid (23) and (b) a dihydroxy-*N*-methyl-L-pipecolic acid [proposed to be (2*S*,3*R*,5*S*)-dihydroxy-L-pipecolic acid (26)];

(c) *cis*P4H yield (2*S*,5*S*)-*cis*-5-hydroxy-*N*-methyl-L-pipecolic acid (24) and (2*S*,3*R*)-*cis*-3-hydroxy-*N*-methyl-L-pipecolic acid (23);

(d) *trans*P4H yields (2*S*,5*R*)-*trans*-5-hydroxy-*N*-methyl-L-pipecolic acid (**25**).

Stereochemical assignments were made by NMR.


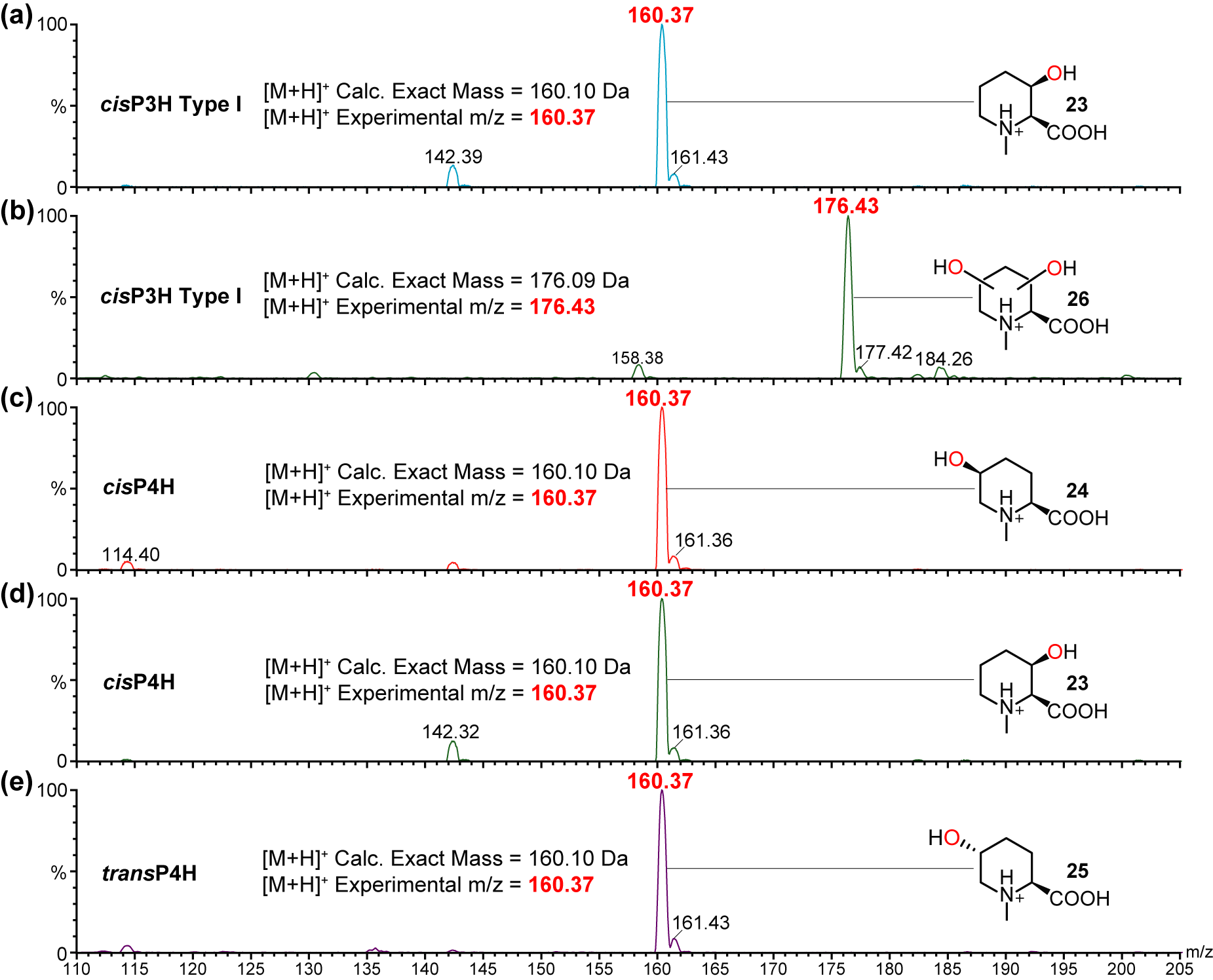


Fig. S32. Mass spectral analyses (LC/MS) of proline hydroxylase reactions using (2*S*)-*N*-methyl-L-pipecolic acid (*N*-Me-Pip) (22):

(a) *cis*P3H yields (2*S*,3*R*)-*cis*-3-hydroxy-*N*-methyl-L-pipecolic acid (23);

(b) *cis*P3H reactions yield a dihydroxy-*N*-methyl-L-pipecolic acid [proposed to be (2*S*,3*R*,5*S*)-dihydroxy-L-pipecolic acid (26)];

(c) *cis*P4H yields (2*S*,5*S*)-*cis*-5-hydroxy-*N*-methyl-L-pipecolic acid (24);

(d) *cis*P4H yields (2*S*,3*R*)-*cis*-3-hydroxy-*N*-methyl-L-pipecolic acid (**23**)**;**

(e) *trans*P4H yields (2*S*,5*R*)-*trans*-5-hydroxy-*N*-methyl-L-pipecolic acid (**25**).

Stereochemical assignments were made by NMR.

.


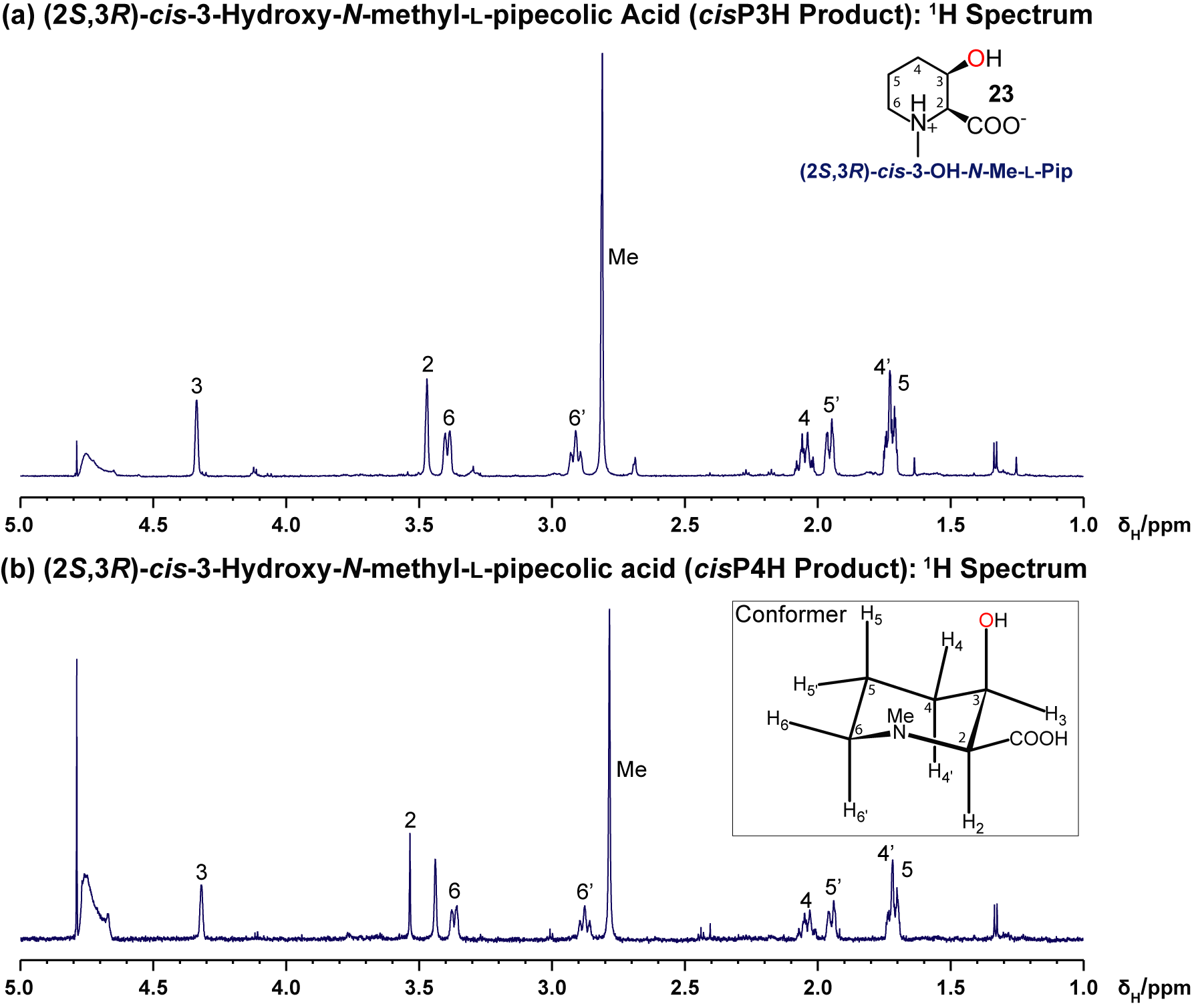


Fig. S33. NMR of the (2*S*,3*R*)-*cis*-3-hydroxy-*N*-methyl-L-pipecolic acid (23) hydroxylation product from (a) *cis*P3H and (b) *cis*P4H reactions using (2*S*)-*N*-methyl-L-pipecolic acid (*N*-Me-Pip) (22): ^1^H-NMR spectra (‘zgpr’ pulse sequence). Chemical shift values are referenced to TSP-*d*_4_ (‘0.0 ppm’).

Assignments

^1^H NMR (700 MHz, D_2_O) δ = 4.36 – 4.31 (m, 1H), 3.47 (d, *J* = 2.2 Hz, 1H), 3.39 (ddd, *J* = 12.9, 1.9, 0.7 Hz, 1H), 2.91 (ddd, *J* = 12.9, 3.2, 0.7 Hz, 1H), 2.81 (s, 3H), 2.05 (dddd, *J* = 14.9, 4.4, 3.7, 0.7 Hz, 1H), 1.96 (ddd, *J* = 14.5, 3.7, 1.9 Hz, 1H), 1.76 – 1.70 (m + m, *J* = 4.4 Hz, *J* = 3.6 Hz, 2H).


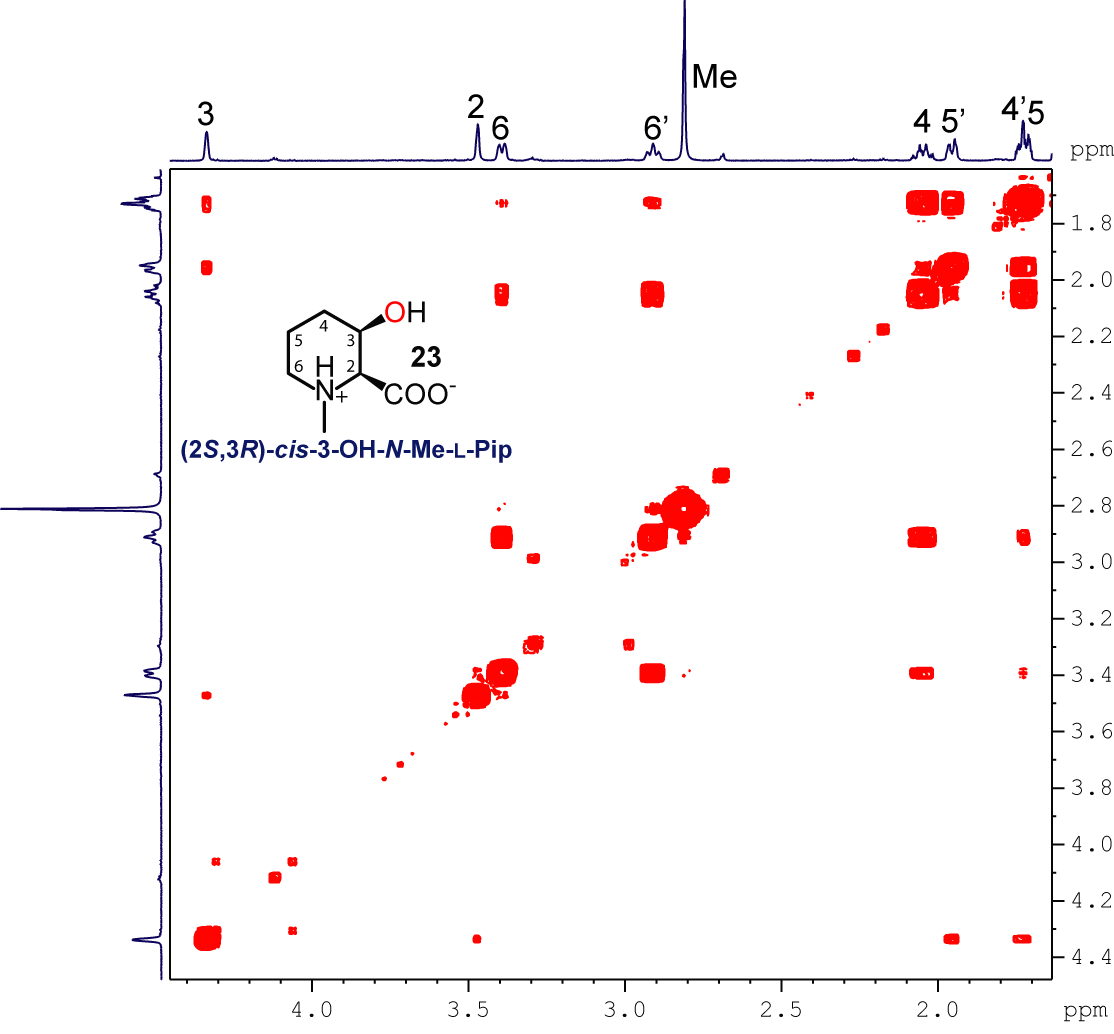


Fig. S34. 2D-NMR of the (2*S*,3*R*)-*cis*-3-hydroxy-*N*-methyl-L-pipecolic acid (23) hydroxylation product from *cis*P3H and *cis*P4H reactions using (2*S*)-*N*-methyl-L-pipecolic acid (*N*-Me-Pip) (22): ^1^H-^1^H COSY spectrum (‘cosygpprf2qf’ pulse sequence). Chemical shift values are referenced to TSP-*d*_4_ (‘0.0 ppm’).


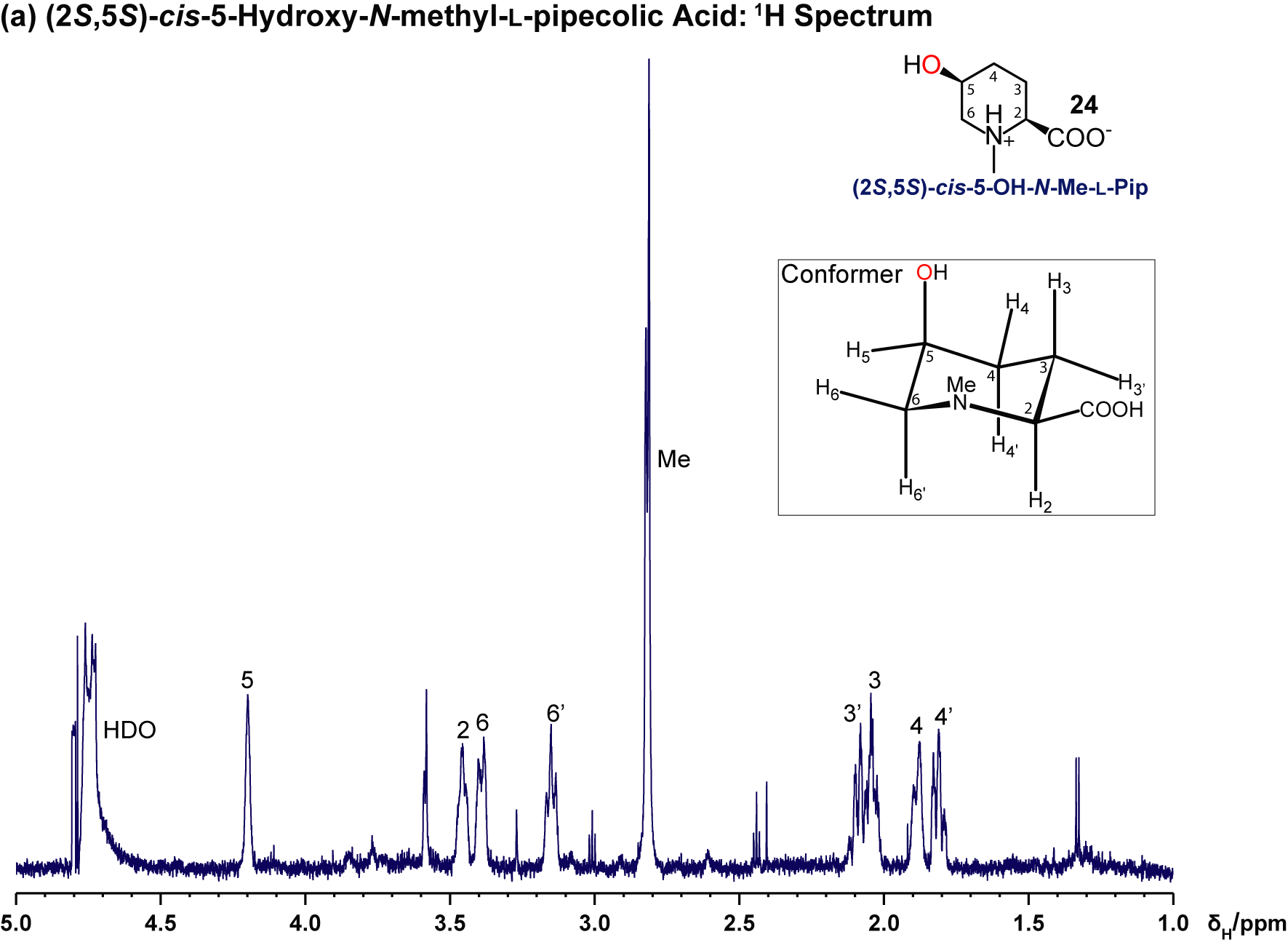


Fig. S35. NMR of the (2*S*,5*S*)-*cis*-5-hydroxy-*N*-methyl-L-pipecolic acid (24) hydroxylation product from *cis*P4H reactions using (2*S*)-*N*-methyl-L-pipecolic acid (*N*-Me-Pip) (22): (a) ^1^H-NMR spectrum (‘zgpr’ pulse sequence). Chemical shift values are referenced to TSP-*d*_4_ (‘0.0 ppm’).

Assignments

^1^H NMR (700 MHz, D_2_O) δ = 4.22 – 4.18 (m, 1H), 3.46 (dd, *J* = 10.4, 3.3 Hz, 1H), 3.39 (dd, *J* = 11.6, 0.9 Hz, 1H), 3.15 (dd, *J* = 11.6, 1.2 Hz, 1H), 2.82 (s, 3H), 2.09 (dddd, *J* = 12.1, 3.3, 2.6, 2.2 Hz, 1H), 2.04 (dddd, *J* = 12.1, 10.4, 4.0, 2.2 Hz, 1H), 1.88 (dddd, *J* = 13.4, 3.9, 2.2, 1.8 Hz, 1H), 1.81 (dddd, *J* = 13.4, 4.0, 3.1, 2.6 Hz, 1H).


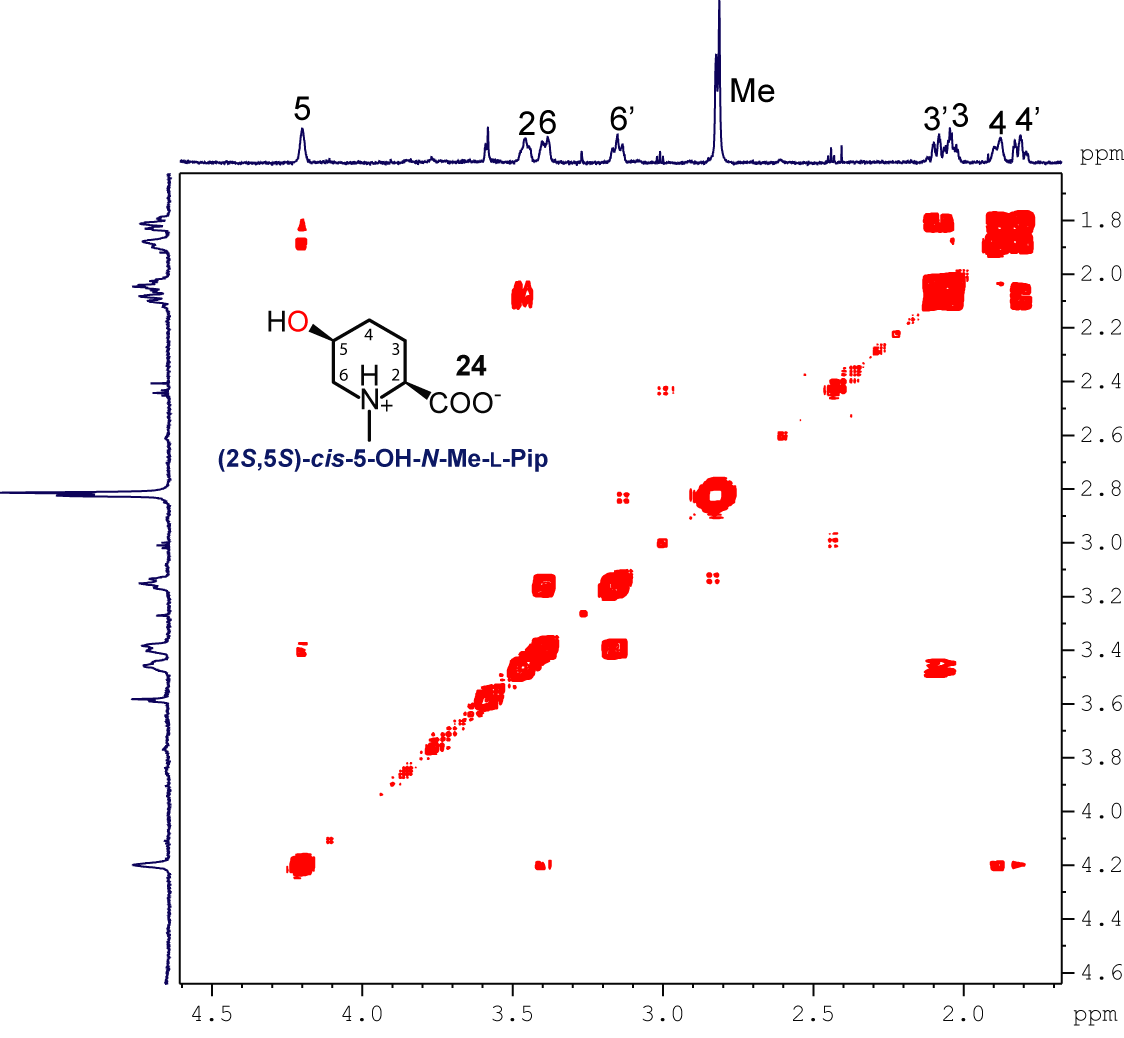


Fig. S36. 2D-NMR of the (2*S*,5*S*)-*cis*-5-hydroxy-*N*-methyl-L-pipecolic acid (24) hydroxylation product from *cis*P4H reactions using (2*S*)-*N*-methyl-L-pipecolic acid (*N*-Me-Pip) (22): ^1^H-^1^H COSY spectrum (‘cosygpprf2qf’ pulse sequence). Chemical shift values are referenced to TSP-*d*_4_ (‘0.0 ppm’).


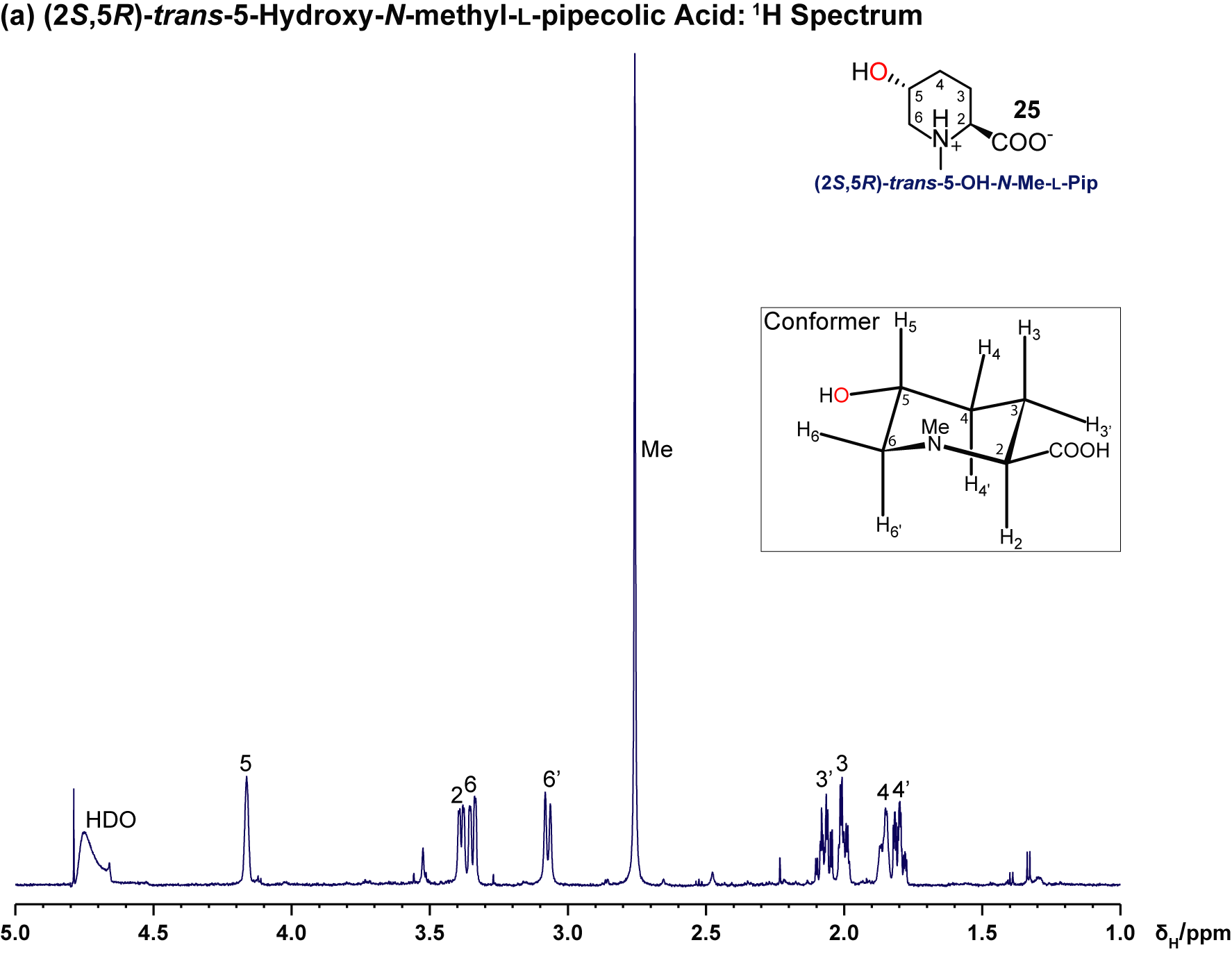


Fig. S37 NMR of the (2*S*,5*R*)-*trans*-5-hydroxy-*N*-methyl-L-pipecolic acid (25) hydroxylation product from *trans*P4H reactions using (2*S*)-*N*-methyl-L-pipecolic acid (*N*-Me-Pip) (22): (a) ^1^H-NMR spectrum (‘zgpr’ pulse sequence). Chemical shift values are referenced to TSP-*d*_4_ (‘0.0 ppm’).

Assignments

^1^H NMR (700 MHz, D_2_O) δ = 4.19 – 4.14 (m, 1H), 3.38 (dd, *J* = 10.9, 3.1 Hz, 1H), 3.35 (dd, *J* = 13.0, 3.1 Hz, 1H), 3.07 (dd, *J* = 13.0, 0.7 Hz, 1H), 2.76 (s, 3H), 2.07 (dddd, *J* = 14.3, 3.9, 3.3, 3.1 Hz, 1H), 2.00 (dddd, *J* = 14.3, 10.9, 8.1, 4.3 Hz, 1H), 1.85 (dddd, *J* = 14.4, 4.3, 3.9, 3.4, Hz, 1H), 1.80 (dddd, *J* = 14.4, 8.1, 7.5, 3.3 Hz, 1H).


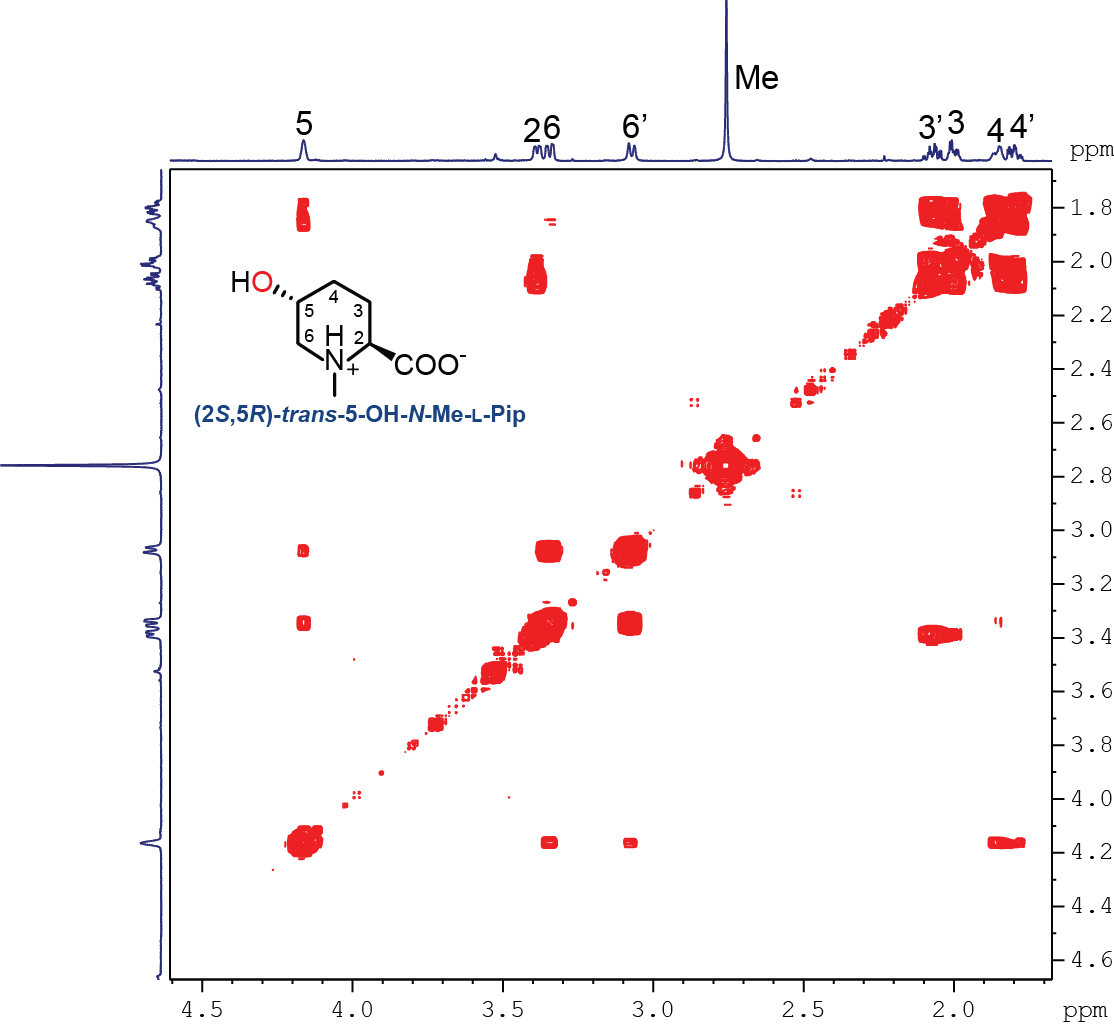


Fig. S38. 2D-NMR of the (2*S*,5*R*)-*trans*-5-hydroxy-*N*-methyl-L-pipecolic acid (25) hydroxylation product from *trans*P4H reactions using (2*S*)-*N*-methyl-L-pipecolic acid (*N*-Me-Pip) (22): ^1^H-^1^H COSY spectrum (‘cosygpprf2qf’ pulse sequence).

Spectra were measured at 298 K using on a 700 MHz Bruker AVIII 700 spectrometer (with a ^1^H/^13^C/^15^N TCI CryoProbe); presaturation (with O1P = 4.701 ppm) was used to suppress the HDO peak (which is set to 4.701 ppm). Samples were prepared in 1 mm tubes containing (16 μL) D_2_O containing 0.05% (w/v) TSP-*d*_4_; after spectra were measured, chemical shift values are referenced to TSP-*d*_4_ (‘0.0 ppm’).

## *Ring-substituted Substrate Analogues*

1. *(2S)-2-Methyl-L-proline (****27****)*

Scheme S6. Proline hydroxylase reactions using (2*S*)-2-methyl-L-proline (2-Me-Pro) (27):

(a) *cis*P3H catalyses the production of a hydroxy-2-methyl-L-proline [proposed to be (2*S*,3*R*)-*cis*-3-hydroxy-2-methyl-L-proline (28)];

(b) *cis*P4H catalyses the production of (2*S*,4*S*)-*cis*-4-hydroxy-2-methyl-L-proline (29) and a hydroxy-2-methyl-L-proline [proposed to be (2*S*,3*R*)-*cis*-3-hydroxy-2-methyl-L-proline (28)];

**(c)** *trans*P4H catalyses the production of a hydroxy-2-methyl-L-proline product [proposed to be (2*S*,4*R*)-*trans*-4-hydroxy-2-methyl-L-proline (**30**)].


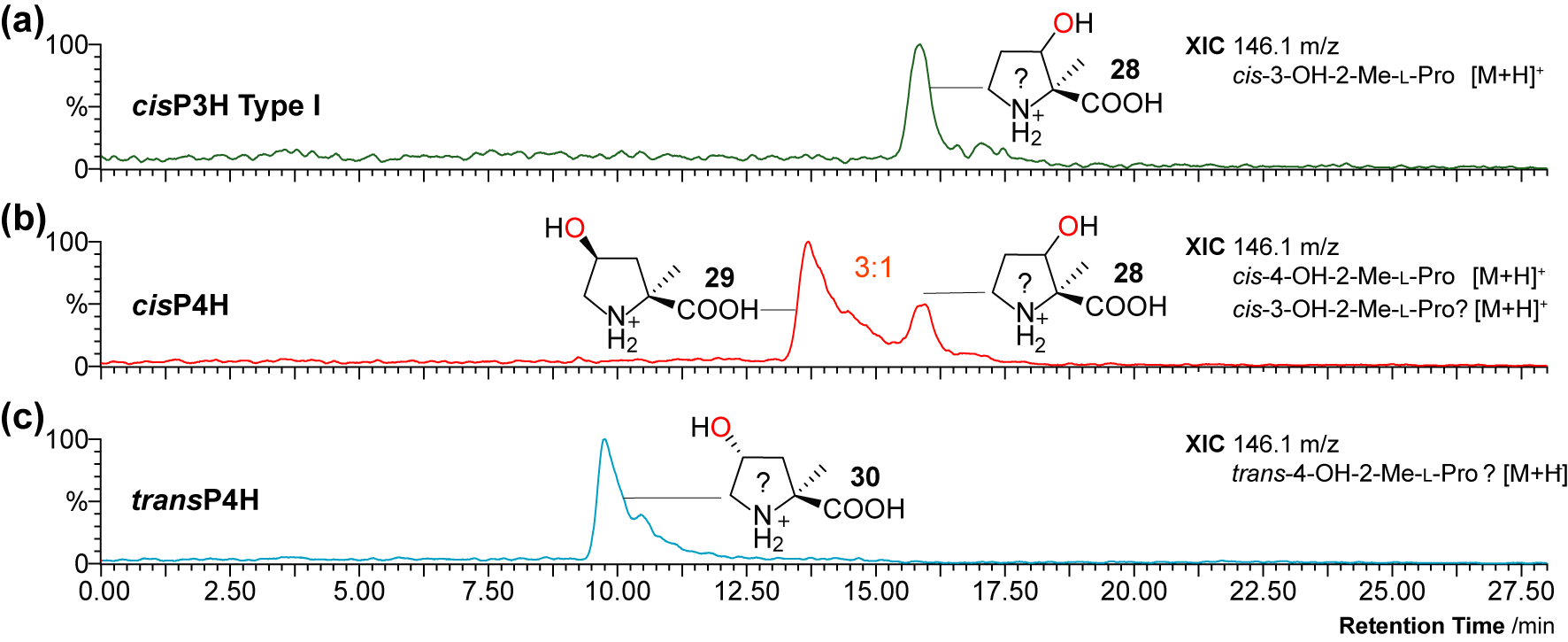


Fig. S39. Extracted-ion count (XIC) LC/MS chromatograms for the proline hydroxylase reactions using (2*S*)-2-methyl-L-proline (2-Me-Pro) (27):

(a) *cis*P3H reactions yield a hydroxy-2-methyl-L-proline [proposed to be (2*S*,3*R*)-*cis*-3-hydroxy-2-methyl-L-proline (28)];

(b) *cis*P4H reactions yield (2*S*,4*S*)-*cis*-4-hydroxy-2-methyl-L-proline (29) and a hydroxy-2-methyl-L-proline [propsed to be (2*S*,3*R*)-*cis*-3-hydroxy-2-methyl-L-proline (28)];

**(c)** *trans*P4H reactions yield a hydroxy-2-methyl-L-proline product [proposed to be (2*S*,4*R*)-*trans*-4-hydroxy-2-methyl-L-proline (**30**)]**.**

Stereochemical assignments were made by NMR.


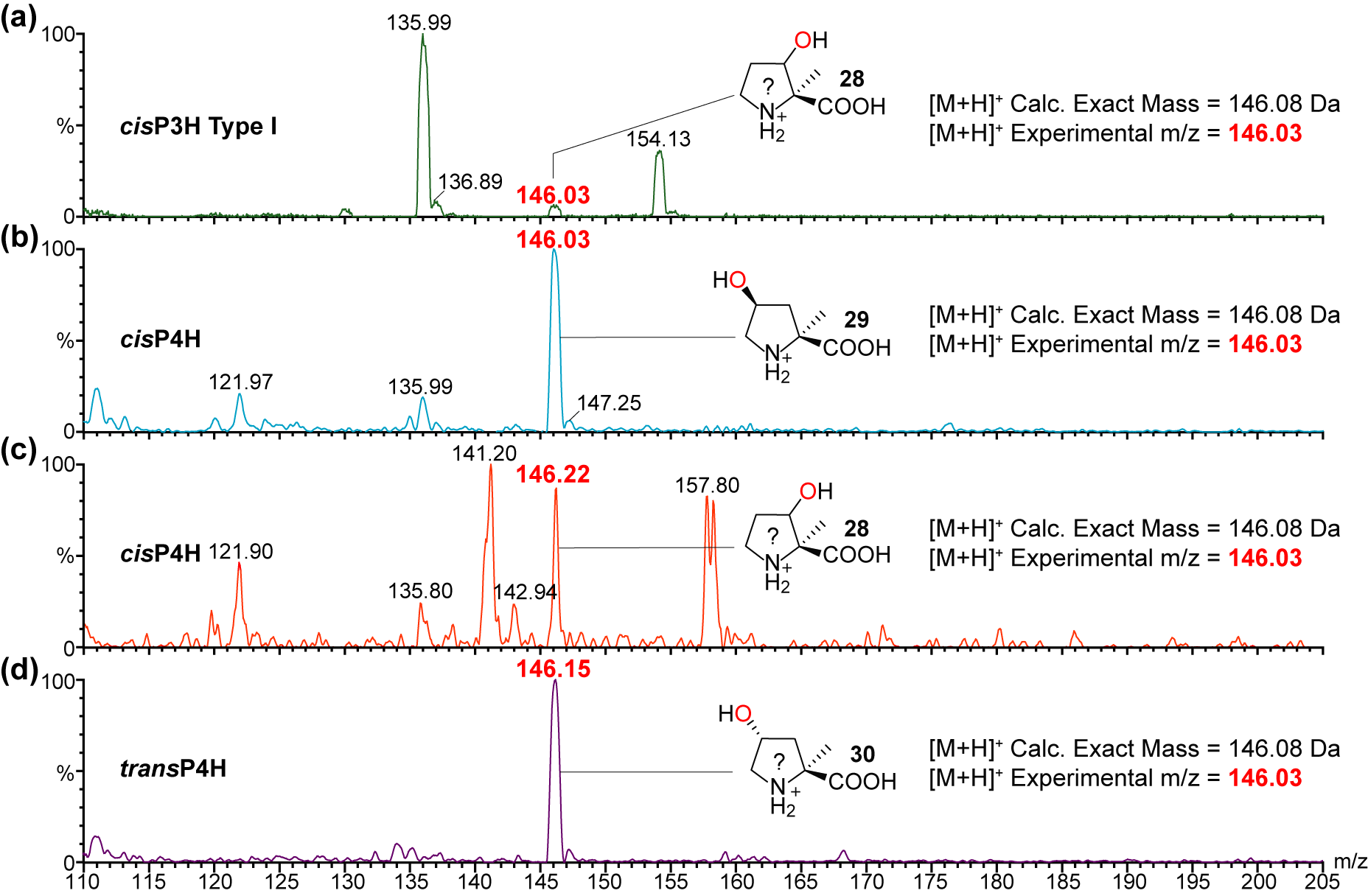


Fig. S40. LC/MS spectra of proline hydroxylase reactions using (2*S*)-2-methyl-L-proline (2-Me-Pro) (27):

(a) *cis*P3H reactions yield a hydroxy-2-methyl-L-proline [proposed to be (2*S*,3*R*)-*cis*-3-hydroxy-2-methyl-L-proline (28)];

(b) *cis*P4H reactions yield (2*S*,4*S*)-*cis*-4-hydroxy-2-methyl-L-proline (29);

(c) *cis*P4H reactions yield a hydroxy-2-methyl-L-proline product [proposed to be (2*S*,3*R*)-*cis*-3-hydroxy-2-methyl-L-proline (28)];

**(d)** *trans*P4H reactions yield a hydroxy-2-methyl-L-proline product [proposed to be (2*S*,4*R*)-*trans*-4-hydroxy-2-methyl-L-proline (**30**)].

Stereochemical assignments were made by NMR.


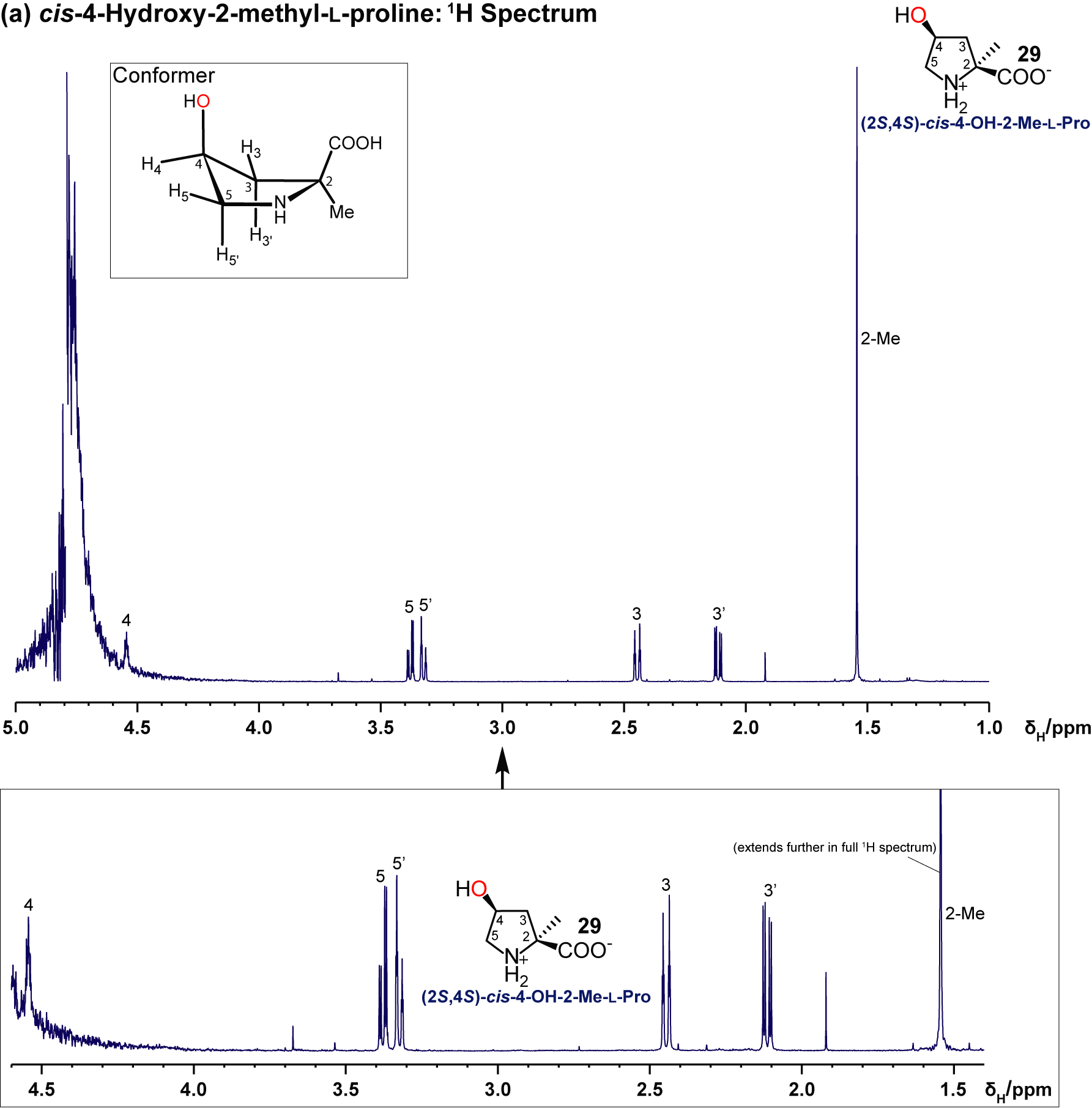


Fig. S41. NMR analyses of the (2*S*,4*S*)-*cis*-4-hydroxy-2-methyl-L-proline (29) hydroxylation product from *cis*P4H reactions using (2*S*)-2-methyl-L-proline (2-Me-Pro) (27): ^1^H-NMR spectrum (‘zgpr’ pulse sequence). Chemical shift values are referenced to TSP-*d*_4_ (‘0.0 ppm’).

Assignments

^1^H NMR (700 MHz, D_2_O) δ = 4.54 (dddd, *J* = 4.8, 4.1, 1.9, 1.8 Hz, 1H), 3.38 (dd, *J* = 12.4, 4.1 Hz, 1H), 3.32 (dd, *J* = 12.4, 1.8 Hz, 1H), 2.45 (dd, *J* = 14.3, 1.9 Hz, 1H), 2.11 (dd, *J* = 14.2, 4.8 Hz, 1H), 1.54 (s, 3H).


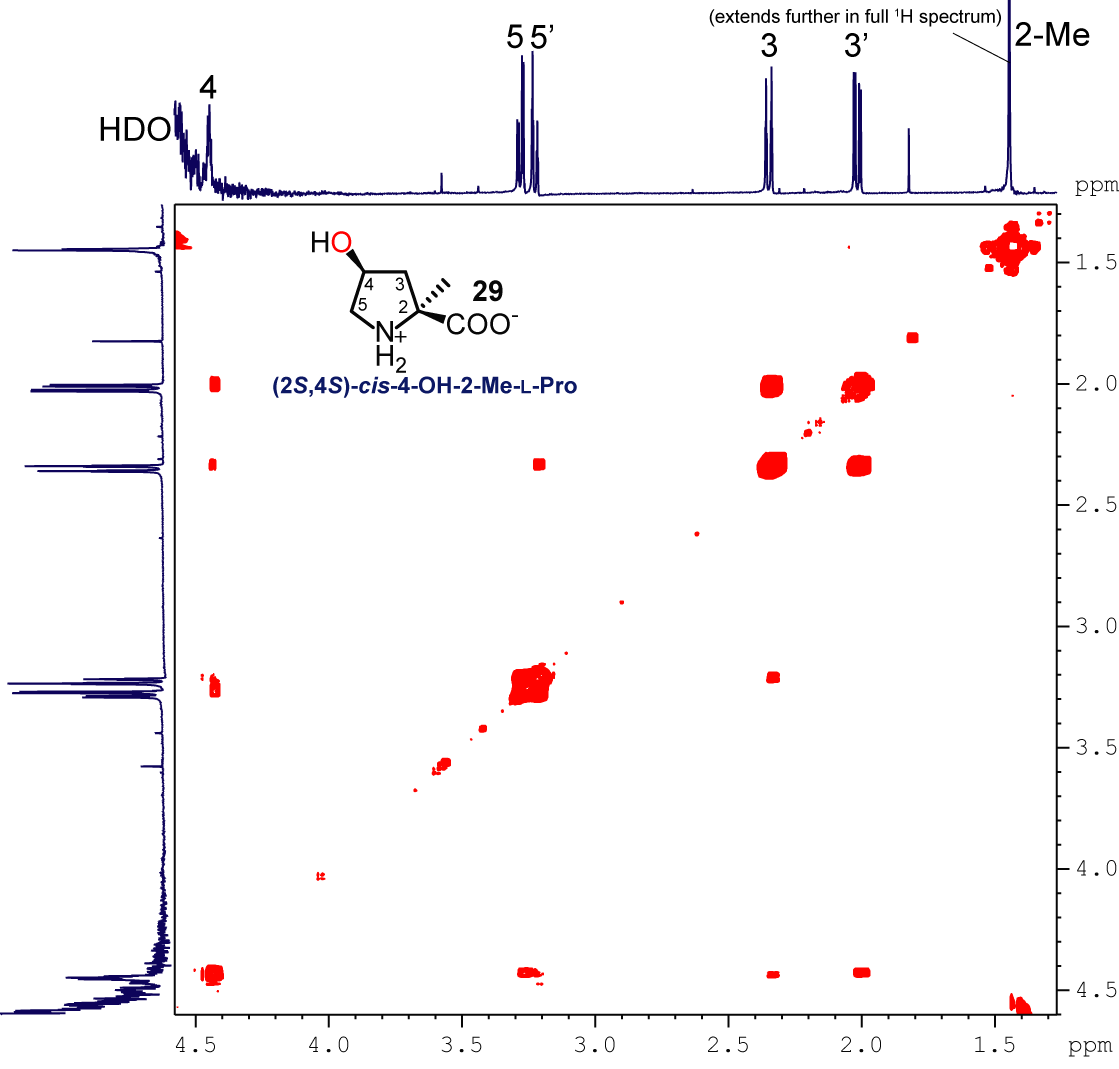


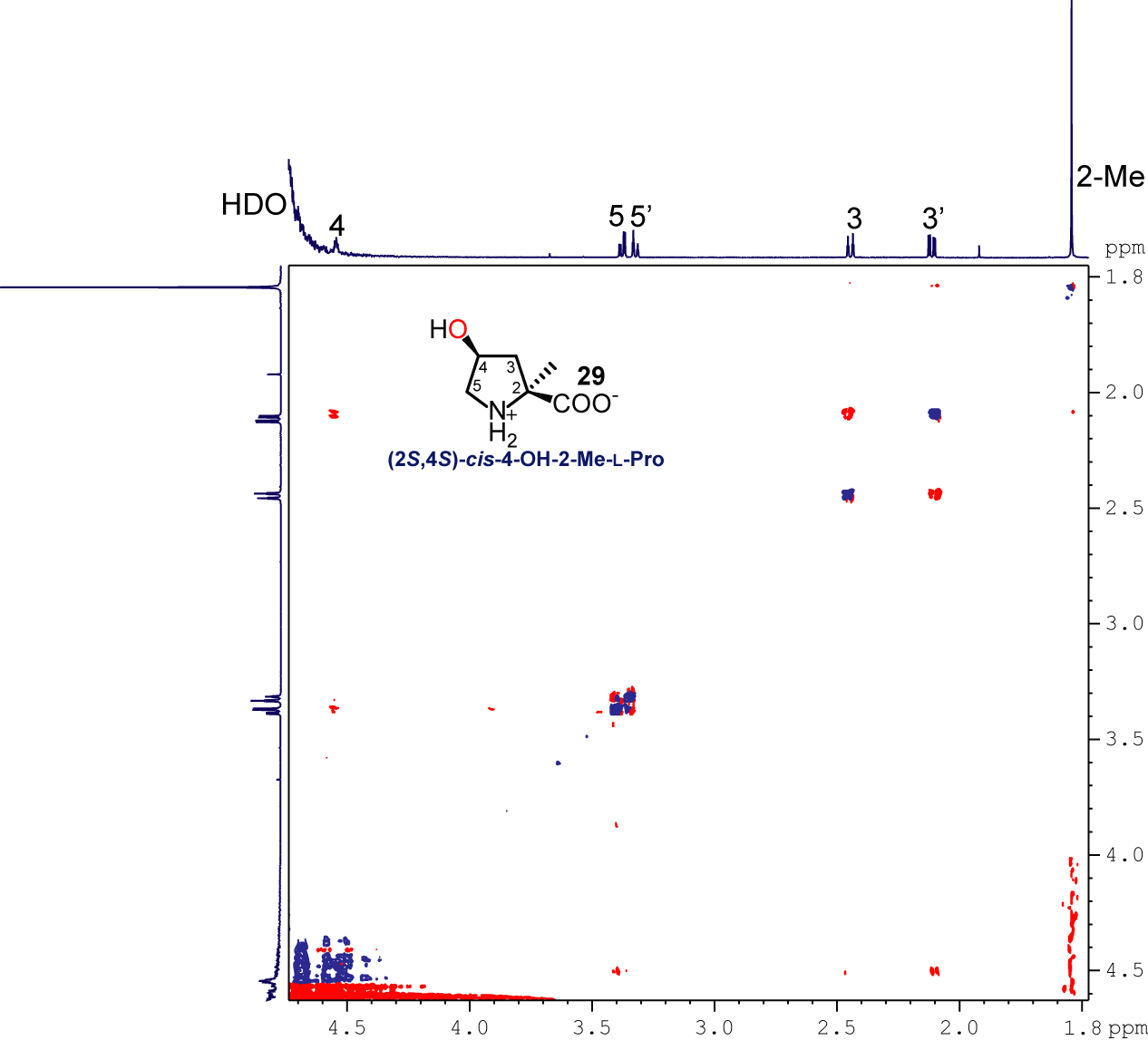


Fig. S42. 2D-NMR analyses of the (2*S*,4*S*)-*cis*-4-hydroxy-2-methyl-L-proline (29) hydroxylation product from *cis*P4H reactions using 2*-*methyl-L-proline (2-Me-Pro) (27):

(a) ^1^H-^1^H COSY spectrum (‘cosygpprf2qf’ pulse sequence);

**(b)** ^1^H-^1^H NOESY spectrum (‘noesyphprf2’ pulse sequence). Chemical shift values are referenced to TSP-*d*_4_ (‘0.0 ppm’).

**(a)**

**(b)**

1. *(2S,4R)-trans-4-(prop-2-yn-1-yl)-L-proline (****31****)*

Scheme S7. Proline hydroxylase reactions using (2*S*,4*R*)-*trans*-4-(prop-2-yn-1-yl)-L-proline (31): *cis*P3H and *cis*P4H catalyze the production of (2*S*,3*R*,4*S*)-3-hydroxy-4-(prop-2-yn-1-yl)-L-proline (32).


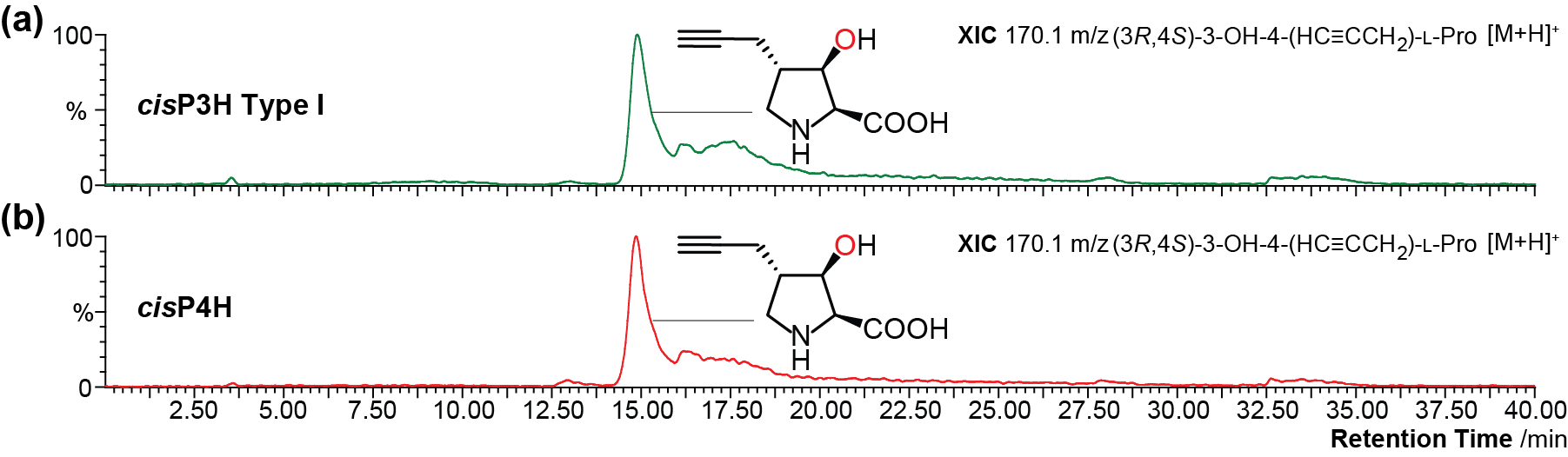


Fig. S43. Extracted-ion count (XIC) LC/MS chromatograms for the proline hydroxylase reactions using (2*S*,4*R*)-*trans*-4-(prop-2-yn-1-yl)-L-proline (31): (a) *cis*P3H and (b) *cis*P4H catalyse the production of (2*S*,3*R*,4*S*)-3-hydroxy-4-(prop-2-yn-1-yl)-L-proline (32).

Stereo chemical assignments were made by NMR.


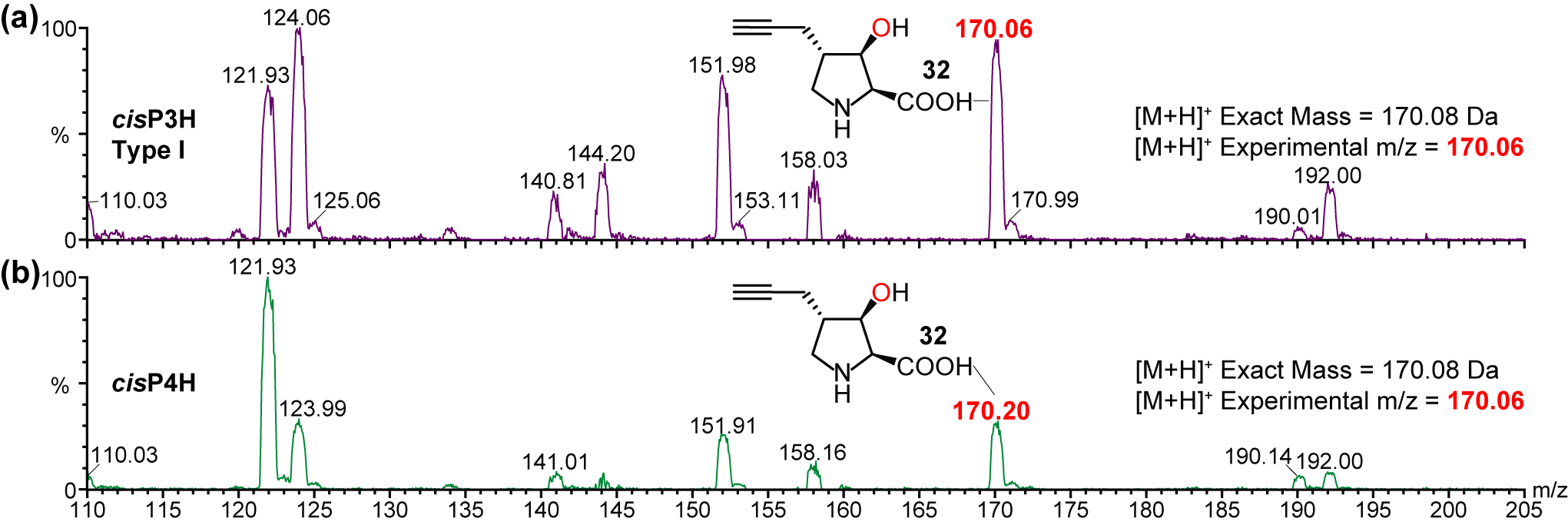


Fig. S44. LC/MS spectra of proline hydroxylase reactions using (2*S*,4*R*)-*trans*-4-(prop-2-yn-1-yl)-L-proline (31):

(a) *cis*P3H and (b) *cis*P4H catalyse the production of (2*S*,3*R*,4*S*)-3-hydroxy-4-(prop-2-yn-1-yl)-L-proline (32)

Stereo chemical assignments were made by NMR.


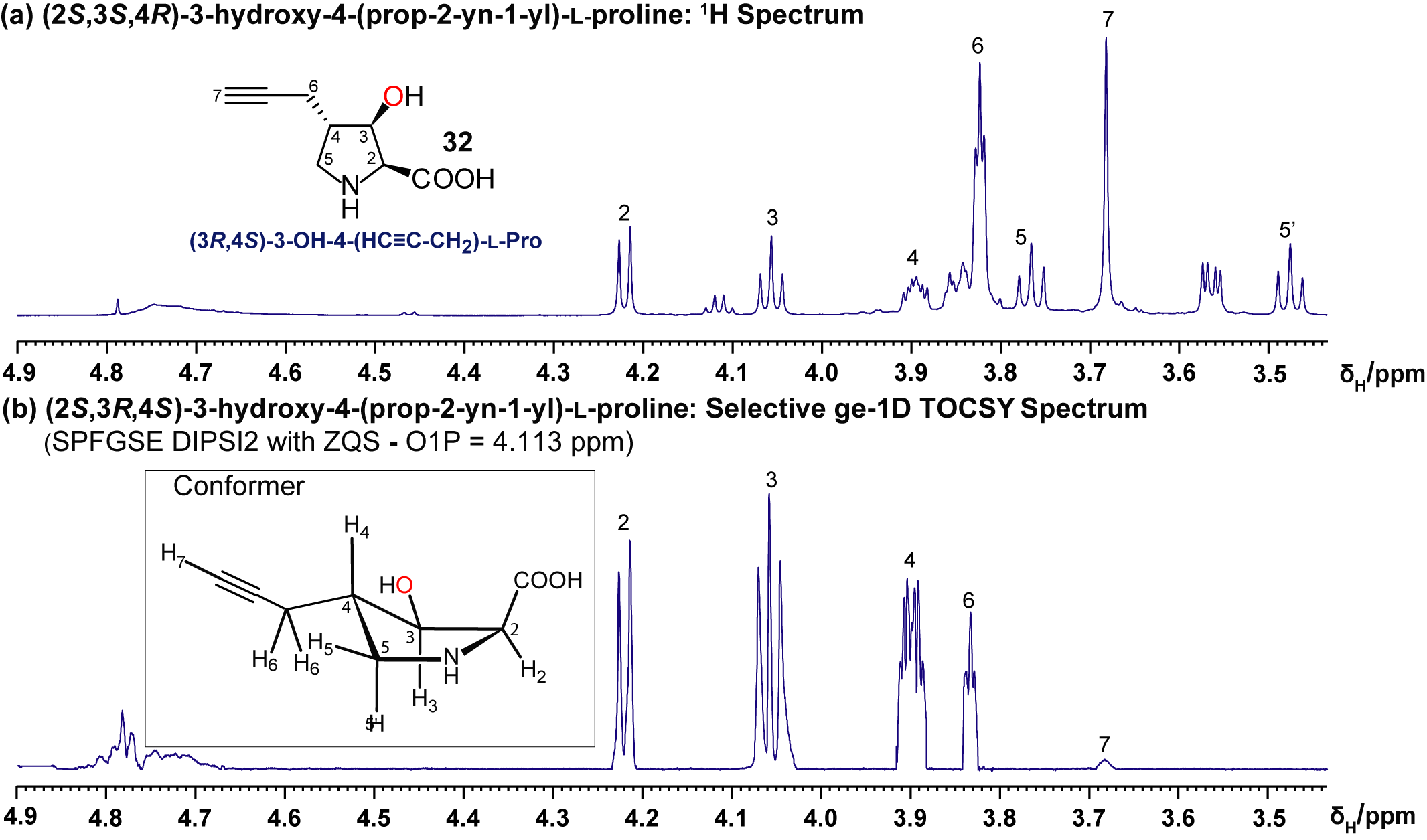


Fig. S45. NMR analyses of the (2*S*,3*R*,4*S*)-3-hydroxy-4-(prop-2-yn-1-yl)-L-proline (32) hydroxylation product from *cis*P4H reactions using (2*S*,4*R*)-*trans*-4-(prop-2-yn-1-yl)-L-proline (31): (a) ^1^H-NMR spectrum (‘zgpr’ pulse sequence) and (b) selective 1D ge-TOCSY (‘spfgsedipsi2zs’ pulse sequence).

^1^H NMR (700 MHz, D_2_O) δ = 4.22 (d, *J* = 6.9 Hz, 1H), 4.15 (dd, *J* = 6.9, 4.2 Hz, 1H), 3.90 (ddtd, *J* = 7.7, 6.9, 4.5, 4.2 Hz, 1H), 3.83 (d, *J* = 7.7, 6.9, 4.5, 4.2 Hz, 1H), 3.78 (dd, *J* = 9.4, 6.9 Hz, 1H), 3.68 (s, 1H), 3.48 (dd, *J* = 9.4, 7.7, 1H).


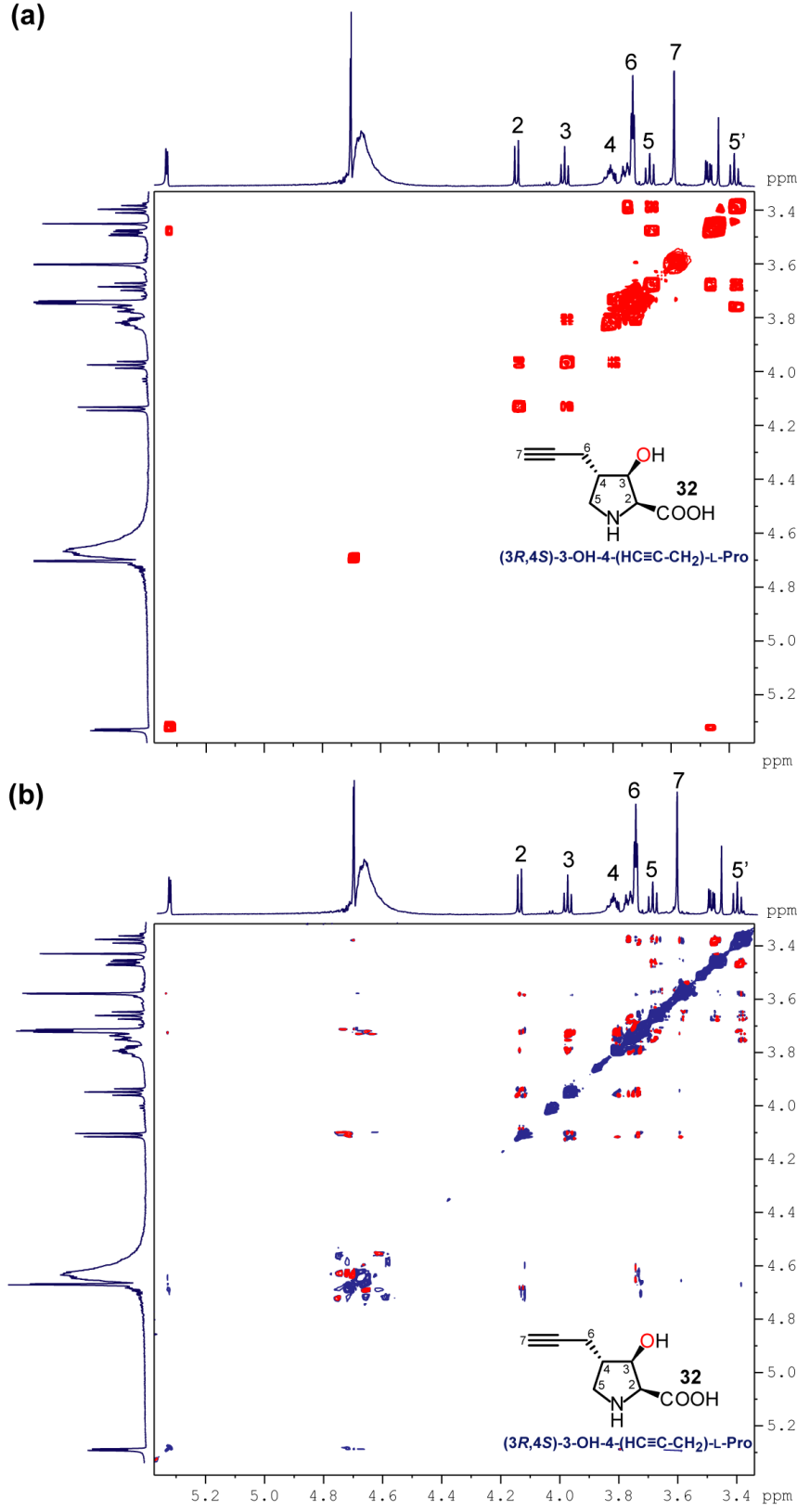


Fig. S4644. 2D-NMR analyses of the (2*S*,3*R*,4*S*)-3-hydroxy-4-(prop-2-yn-1-yl)-L-proline (32) hydroxylation product from *cis*P4H reactions using (2*S*,4*R*)-*trans*-4-(prop-2-yn-1-yl)-L-proline (31):

(a) ^1^H-^1^H COSY spectrum (‘cosygpprf2qf’ pulse sequence);

**(b)** ^1^H-^1^H NOESY spectrum (‘noesyphprf2’ pulse sequence).

Chemical shift values are referenced to TSP-*d*_4_ (‘0.0 ppm’).

1. *(2S,4R)-trans-5-(2-methoxy-2-oxoethyl)-L-proline (‘Me-tCMP’)* *(****33****)*

**Scheme S8**. Proline hydroxylase reactions using trans-5-(2-methoxy-2-oxoethyl)-L-proline (‘Me-tCMP’) (**33**): cisP3H catalyzes the production of (2S,3R,5S)-3-hydroxy-5-(2-methoxy-2-oxoethyl)-L-proline **(34**)

*
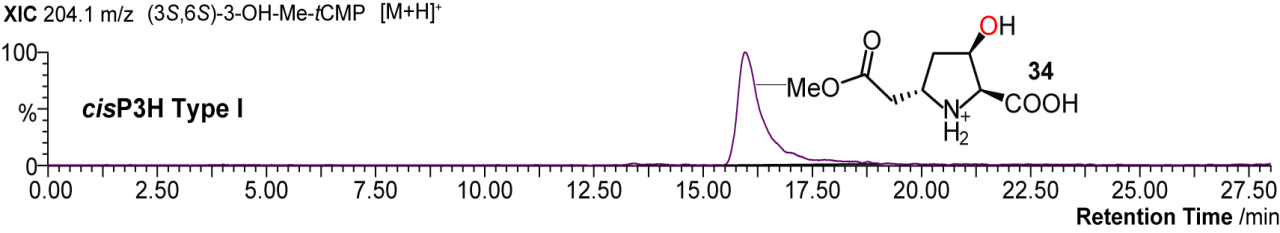
*

Fig. S47. Extracted-ion count (XIC) LC/MS chromatograms for the proline hydroxylase reactions using *trans*-5-(2-methoxy-2-oxoethyl)-L-proline (‘Me-*t*CMP’) (33): *cis*P3H catalyses the production of (2*S*,3*R*,5*S*)-3-hydroxy-5-(2-methoxy-2-oxoethyl)-L-proline (34).

Stereo chemical assignments were made by NMR


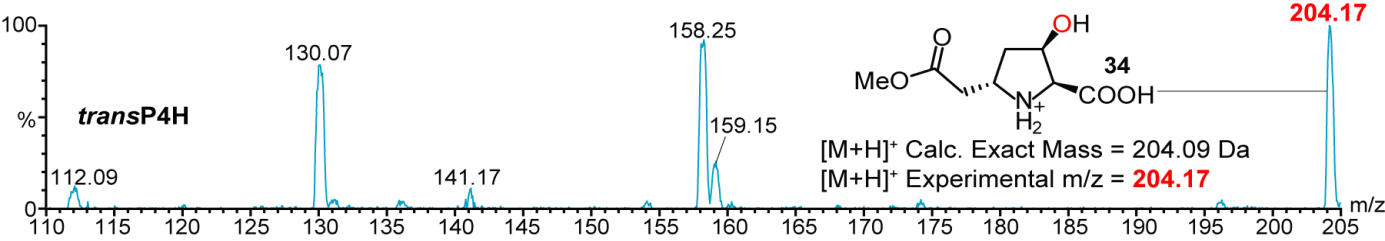


Fig. S48. LC/MS spectrum of proline hydroxylase reactions using (2*S*,5*S*)-*trans*-5-(2-methoxy-2-oxoethyl)-L-proline (‘Me-*t*CMP’) (33): *cis*P3H catalyzes the production of (2*S*,3*R*,5*S*)-3-hydroxy-5-(2-methoxy-2-oxoethyl)-L-proline (34). Stereo chemical assignments were made by NMR.

Fig. S49. NMR analyses of the (2*S*,3*R*,5*S*)-3-hydroxy-5-(2-methoxy-2-oxoethyl)-L-proline (34) hydroxylation product from *cis*P3H reactions using (2*S*,5*S*)-5-(2-methoxy-2-oxoethyl)-L-proline (33): (a) ^1^H-NMR spectrum (‘zgpr’ pulse sequence) and (b) selective 1D ge-TOCSY (‘spfgsedipsi2zs’ pulse sequence). Spectrometer chemical shifts are referenced to solvent (HDO) (4.701 ppm).

Assignments

^1^H NMR (700 MHz, D_2_O) δ = 4.65 (ddd, J = 3.4, 3.2, 1.0 Hz, 1H), 4.29 (dddd, J = 11.7, 11.3, 6.4, 5.2 Hz, 1H), 4.20 (d, J = 3.2 Hz, 1H), 3.69 (s, 3H), 2.89 (dd, J = 11.7, 5.2 Hz, 2H), 2.28 (ddd, J = 14.0, 6.4, 1.0 Hz, 1H), 1.97 (ddd, J = 14.0, 11.3, 3.4 Hz, 1H).

*N.B.* H6 protons show as superimposed dd peaks, rather than d, suggesting that they are anisotropic (perhaps due to intramolecular/intermolecular [dimer] H-bonding).


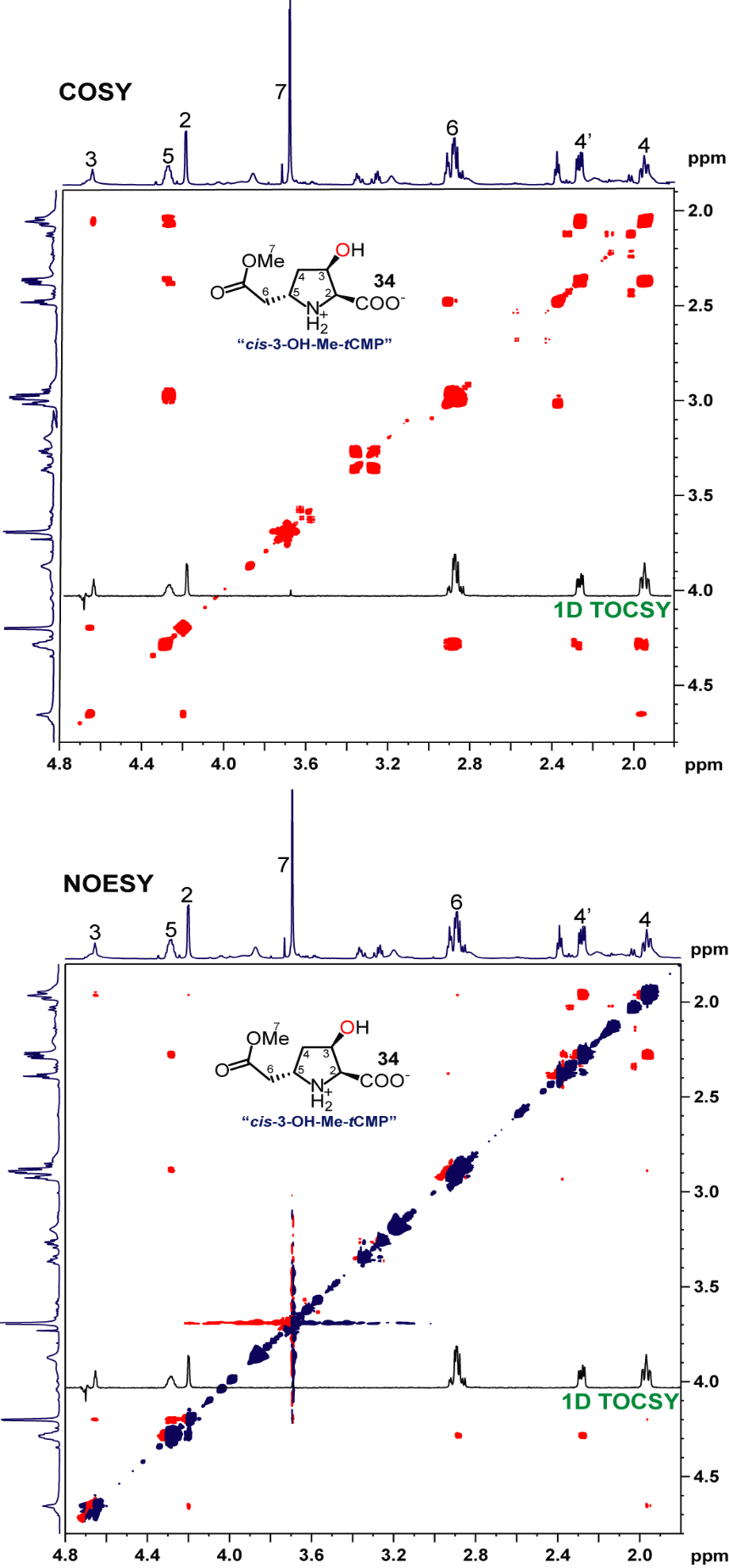


**(b)**

**(a)**

Fig. S50. 2D-NMR analyses of the *trans*-5-(2-methoxy-2-oxoethyl)-L-proline (‘Me-*t*CMP’) (33): *cis*P3H catalyses the production of (2*S*,3*R*,5*S*)-3-hydroxy-5-(2-methoxy-2-oxoethyl)-L-proline (34):

(a) ^1^H-^1^H COSY spectrum (‘cosygpprf2qf’ pulse sequence);

**(b)** ^1^H-^1^H NOESY spectrum (‘noesyphprf2’ pulse sequence).

Spectrometer chemical shifts are referenced to the solvent (HDO) peak (4.701 ppm).

1. *(2S,4S)-cis-4-Fluoro-L-proline (cis-4-F-Pro) (****35****)*

Scheme S9. Proline hydroxylase reactions using (2*S*,4*S*)-*cis*-4-fluoro-L-proline (*cis*-4-F-Pro) (35):

(a) *cis*P3H catalyses the production of (2*S*,3*S*,4*R*)-4-fluoro-3-hydroxy-L-proline (36);

**(b)** *trans*P4H catalyses the production of (2*S*)-4-oxo-L-proline (**37**) (and fluoride).


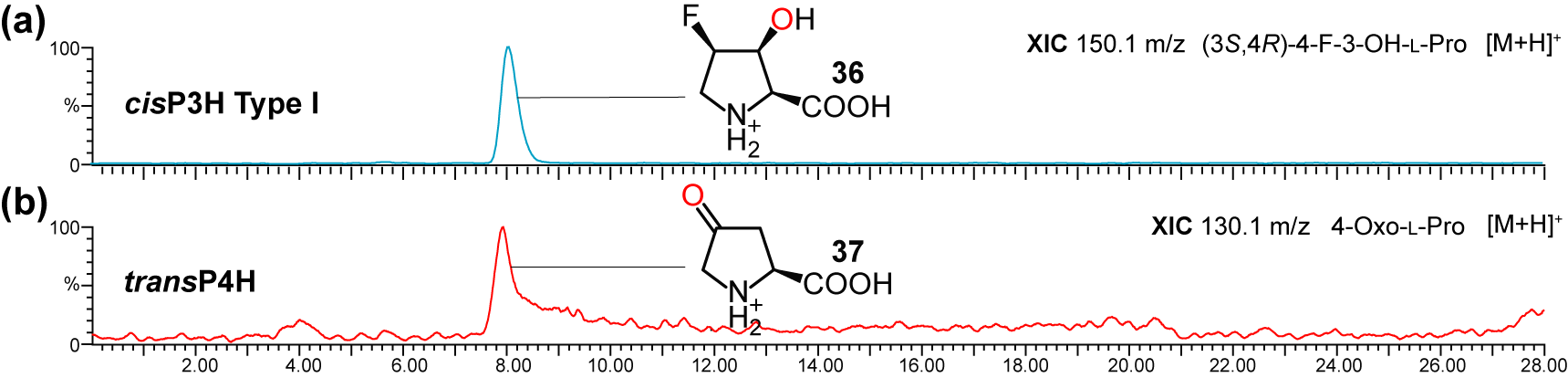


Fig. S51. Extracted-ion count (XIC) LC/MS chromatograms for the proline hydroxylase reactions using (2*S*,4*S*)-*cis*-4-fluoro-L-proline (35):

(a) *cis*P3H reactions yield (2*S*,3*S*,4*R*)-4-fluoro-3-hydroxy-L-proline (36);

**(b)** *trans*P4H reactions yield (2*S*)-4-oxo-L-proline (**37**).


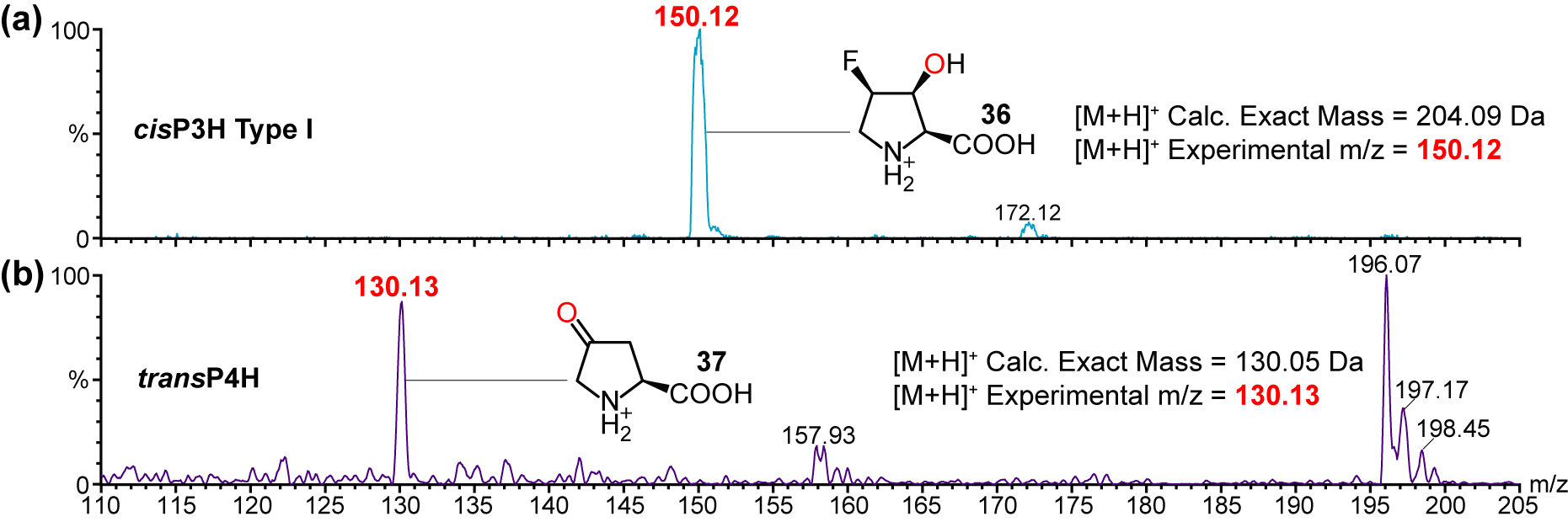


Fig. S52. LC/MS spectra of proline hydroxylase reactions using (2*S*,4*S*)-*cis*-4-fluoro-L-proline (35):

(a) *cis*P3H reactions yield (2*S*,3*S*,4*R*)-4-fluoro-3-hydroxy-L-proline (36);

**(b)** *trans*P4H reactions yield (2*S*)-4-oxo-L-proline (**37**).


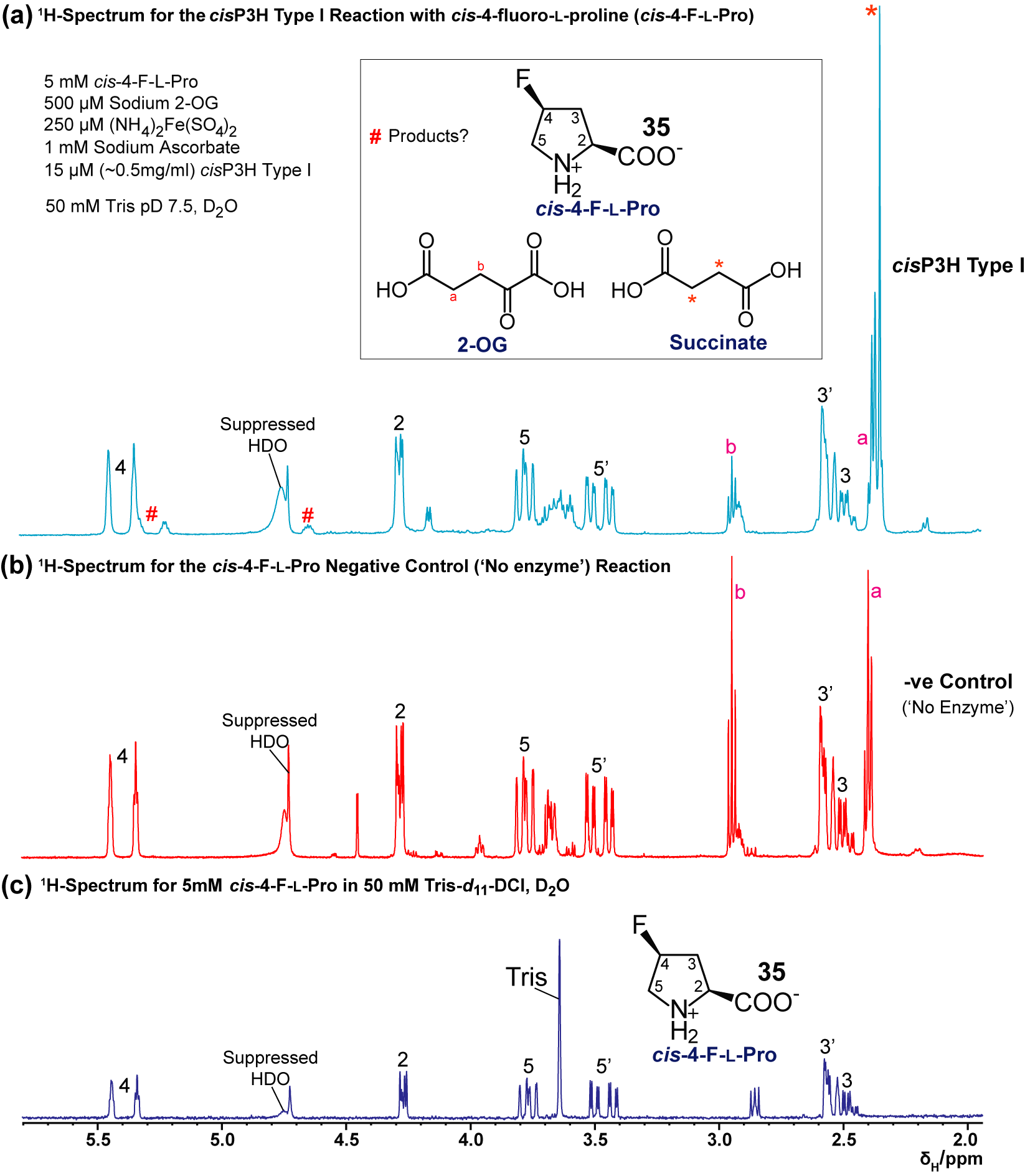


**Fig. S53.** ^1^H-NMR analyses of proline hydroxylase reactions using (2*S*,4*S*)-*cis*-4-fluoro-L-proline (*cis*-4-F-L-Pro) (**35**) [**(a)** *cis*P3H].

Reactions were carried at room temperature (25 °C) for 14 h. Proton-decoupled ^19^F-spectra were measured at 298 K for samples in 5 mm tubes using a 500 MHz (^1^H) Bruker AVII 500 NMR spectrometer [equipped with a 5 mm TFI-^1^H/^19^F(^13^C) probe]. Chemical shift values are referenced to those of CFCl_3_ (where δ_F, CFCl3_ = ‘0.0 ppm’).


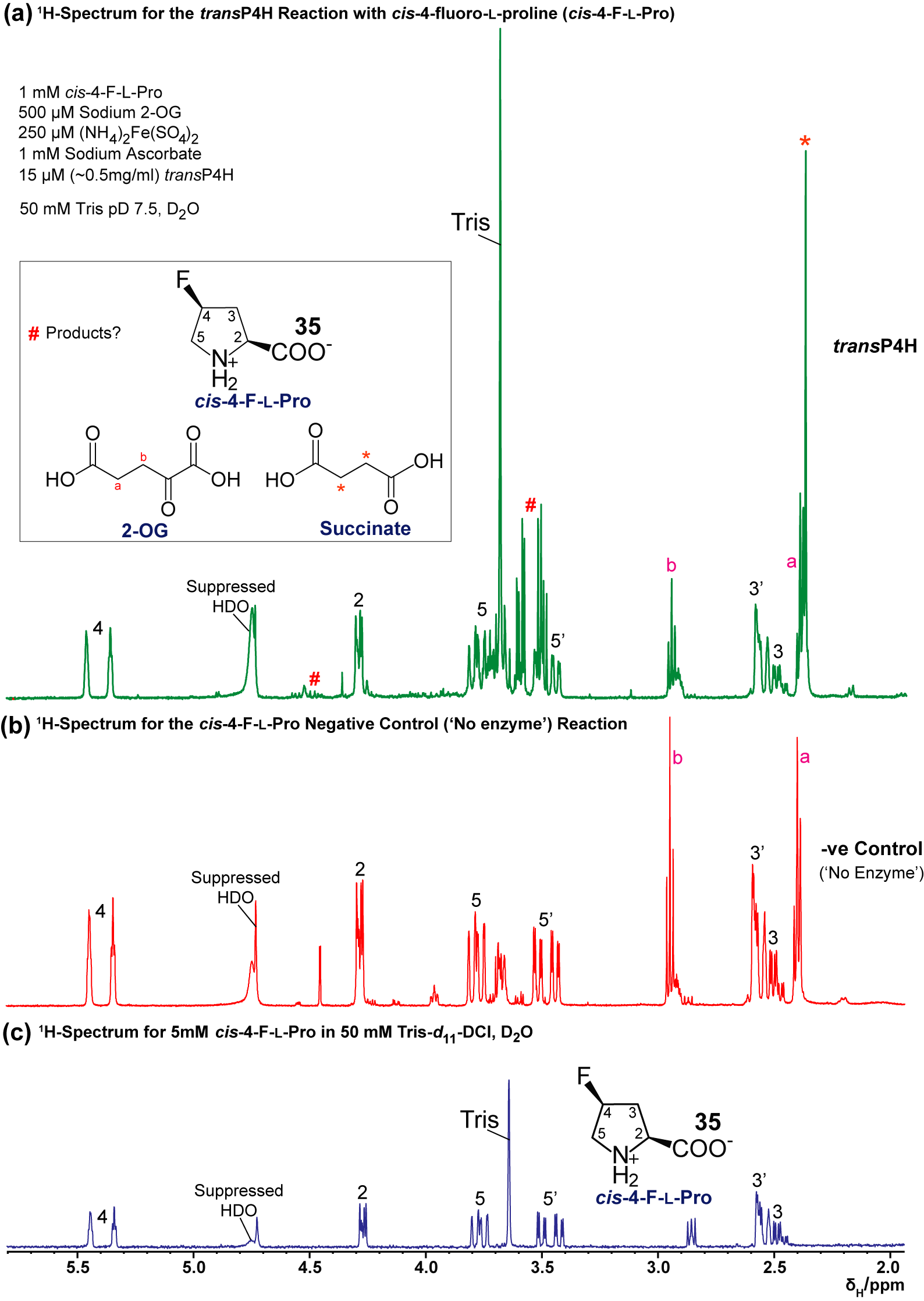


**Fig. S54.** ^1^H-NMR analyses of proline hydroxylase reactions using (2*S*,4*S*)-*cis*-4-fluoro-L-proline (*cis*-4-F-L-Pro) (**35**) [**(a)** *trans*P4H].

Reactions were carried at room temperature (25 °C) for 14 h. Proton-decoupled ^19^F-spectra were measured at 298 K for samples in 5 mm tubes using a 500 MHz (^1^H) Bruker AVII 500 NMR spectrometer [equipped with a 5 mm TFI-^1^H/^19^F(^13^C) probe]. Chemical shift values are referenced to those of CFCl_3_ (where δ_F, CFCl3_ = ‘0.0 ppm’).

.


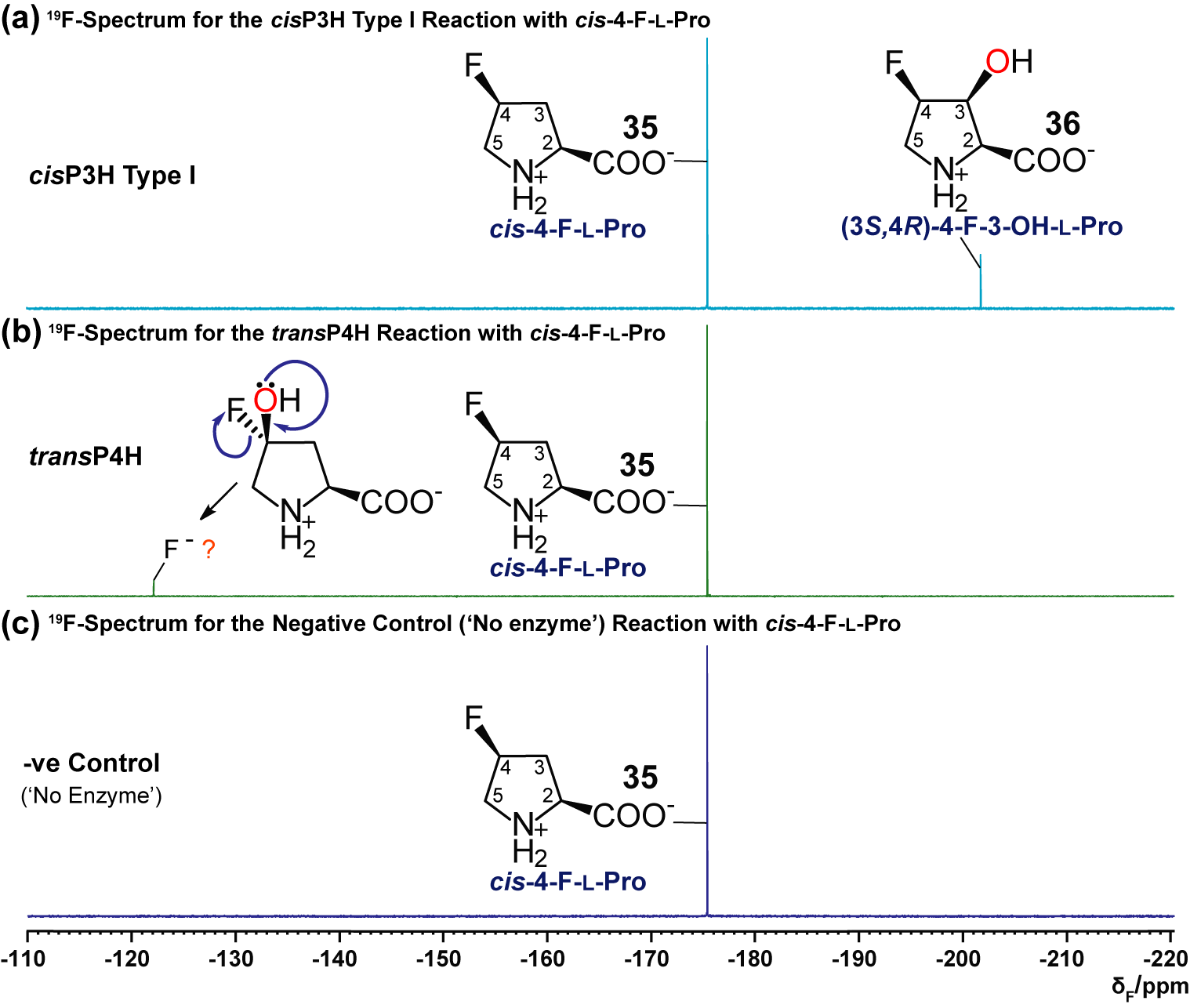


**Fig. S55.** Proton-decoupled ^19^F-NMR analyses of proline hydroxylase reactions using (2*S*,4*S*)-*cis*-4-fluoro-L-proline (*cis*-4-F-L-Pro) (**35**) [**(a)** *cis*P3H Type I and **(b)** *trans*P4H].

Proton-decoupled ^19^F-spectra were measured at 298 K for samples in 5 mm tubes using a 470.4 MHz (^19^F) Bruker AVII 500 NMR spectrometer [equipped with a 5 mm TFI-^1^H/^19^F(^13^C) probe]. Chemical shift values are referenced to those of CFCl_3_ (where δ_F, CFCl3_ = ‘0.0 ppm’).

Starting Material - ^19^F NMR (470 MHz, D_2_O) δ = -175.42 (s, 1F).

Hydroxylation Product - ^19^F NMR (470 MHz, D_2_O) δ = -201.74 (s, 1F).


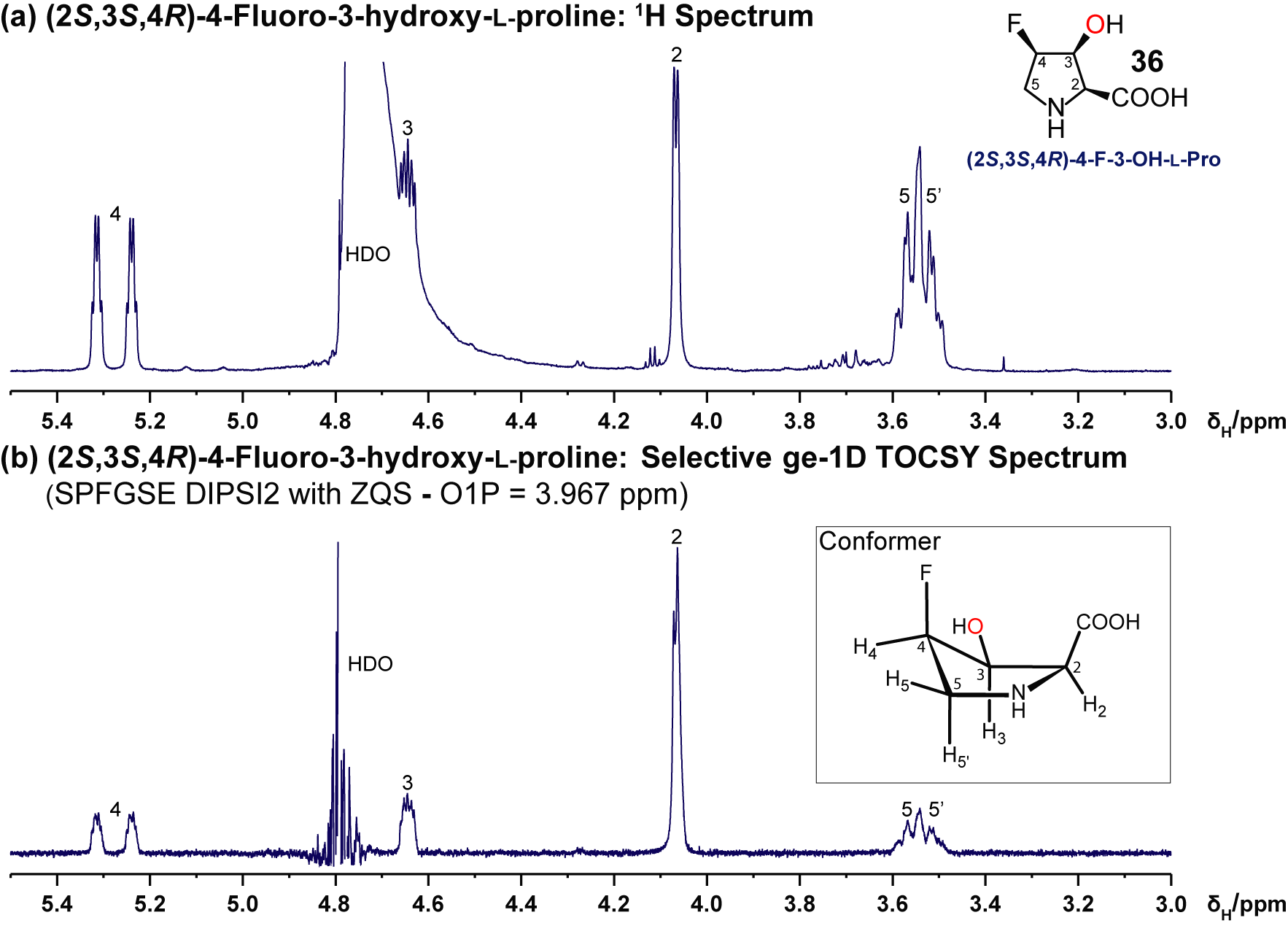


Fig. S56. NMR analyses of the (2*S*,3*S*,4*R*)-4-fluoro-3-hydroxy-L-proline (36) hydroxylation product from *cis*P3H reactions using (2*S*,4*S*)-*cis*-4-fluoro-L-proline (*cis*-4-F-L-Pro) (35): (a) ^1^H-NMR spectrum (‘zgpr’ pulse sequence) and (b) selective 1D ge-TOCSY (‘spfgsedipsi2zs’ pulse sequence). Chemical shift values are referenced to TSP-*d*_4_ (‘0.0 ppm’).

Assignments

^1^H NMR (700 MHz, D_2_O) δ = 5.28 (dddd, *J* = 52.4, 9.4, 4.8, 0.6 Hz, 1H), 4.64 (ddd, *J* = 5.2, 4.8, 0.6 Hz, 1H), 4.07 (d, *J* = 5.2 Hz, 1H), 3.60 – 3.48 (m, 2H).

**(b)**

**(a)**


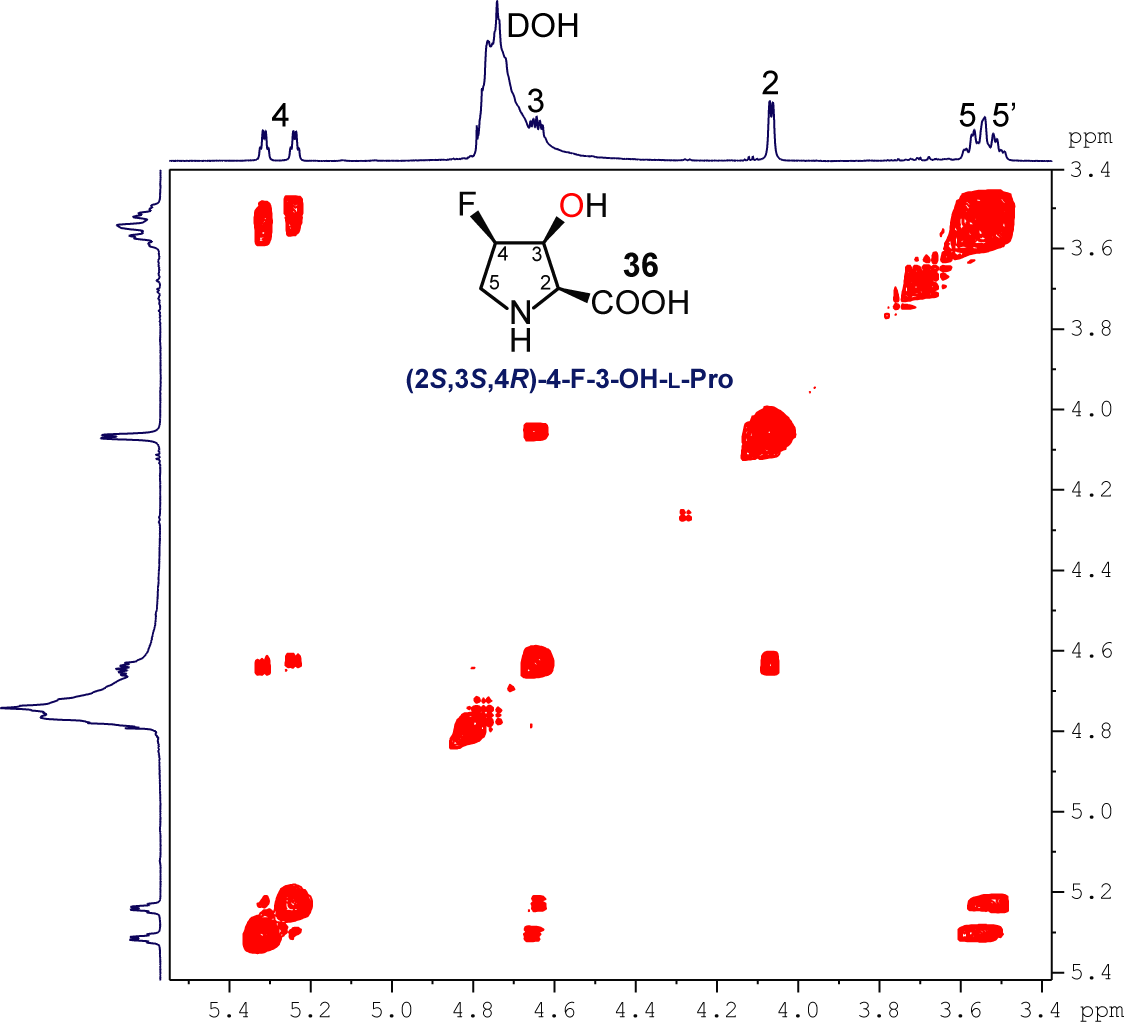

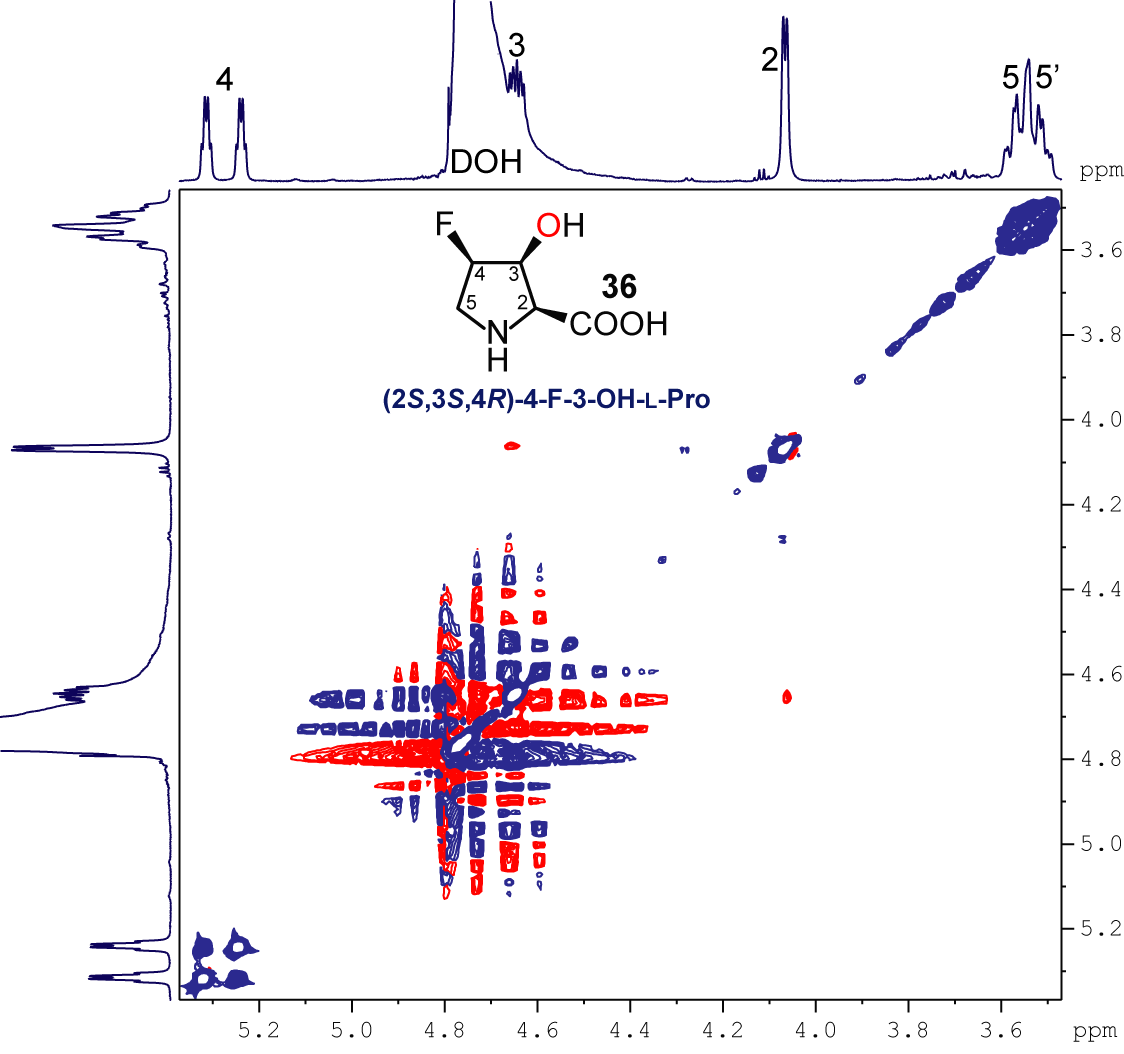


Fig. S57. 2D-NMR analyses of the (2*S*,3*S*,4*R*)-4-fluoro-3-hydroxy-L-proline (36) hydroxylation product from *cis*P4H reactions using (2*S*,4*S*)-*cis*-4-fluoro-L-proline (*cis*-4-F-L-Pro) (35):

(a) ^1^H-^1^H COSY spectrum (‘cosygpprf2qf’ pulse sequence);

**(b)** ^1^H-^1^H NOESY spectrum (‘noesyphprf2’ pulse sequence).

Chemical shift values are referenced to TSP-*d*_4_ (‘0.0 ppm’).

1. *(2S,4R)-trans-4-Fluoro-L-proline (trans-4-F-Pro) (****38****)*

Scheme S10 Proline hydroxylase reactions using (2*S*,4*R*)-*trans*-4-fluoro-L-proline (*trans*-4-F-Pro) (38):

(a) *cis*P3H catalyses the production of (2*S*,3*S*,4*S*)-4-fluoro-3-hydroxy-L-proline (39) and 4-oxo-L-proline (37);

(b) *cis*P4H catalyses the production of (2*S*,3*S*,4*S*)-4-fluoro-3-hydroxy-L-proline (39).


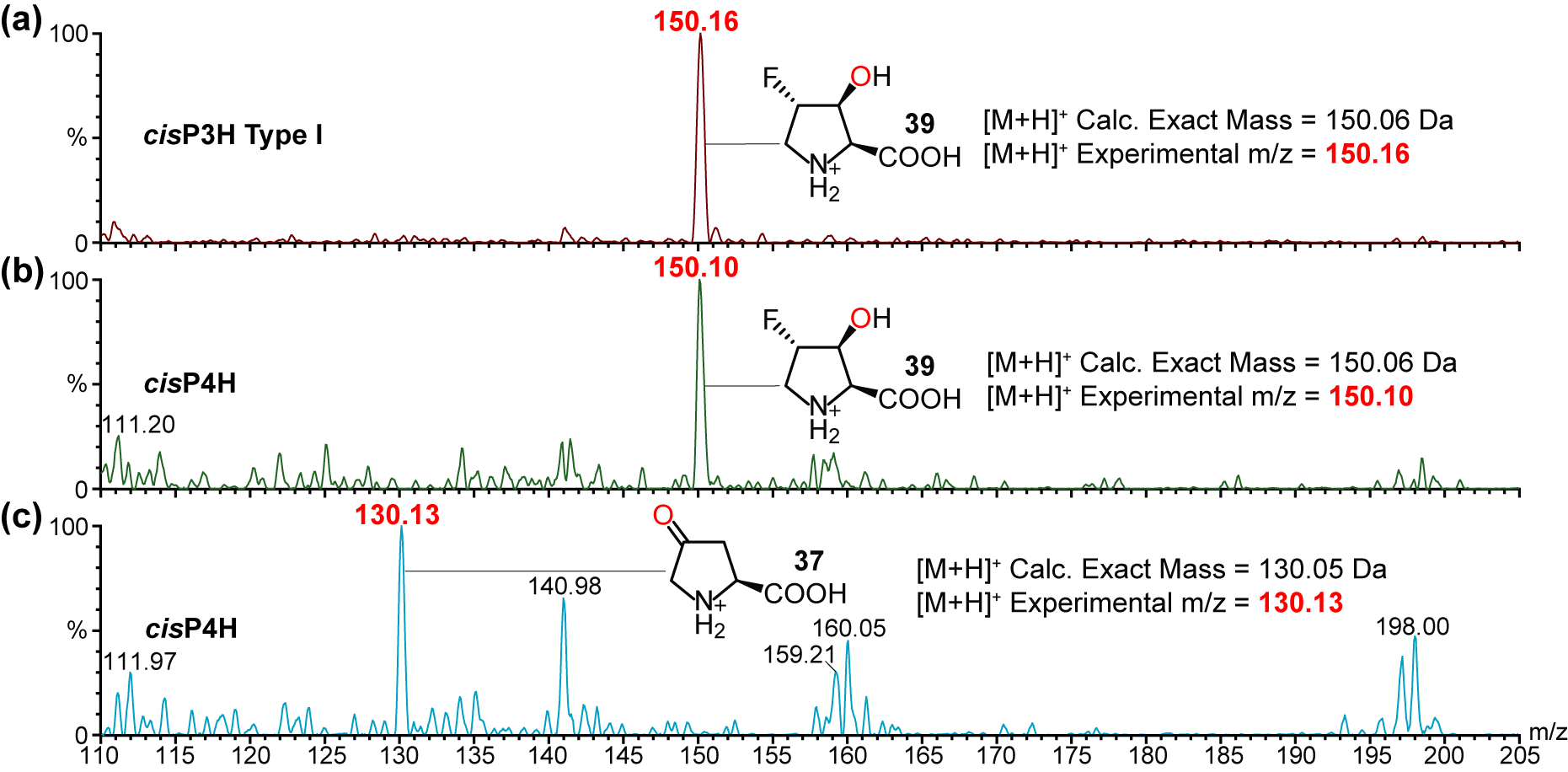


Fig. S59. LC/MS spectra of proline hydroxylase reactions using (2*S*,4*R*)-*trans*-4-fluoro-L-proline (*trans*-4-F-L-Pro) (38):

(a) *cis*P3H reactions yield (2*S*,3*S*,4*S*)-4-fluoro-3-hydroxy-L-proline (39);

(b) *cis*P4H reactions yield (2*S*,3*S*,4*S*)-4-fluoro-3-hydroxy-L-proline (39);

**(c)** *cis*P4H reactions yield (2*S*)-4-oxo-L-proline (**37**).


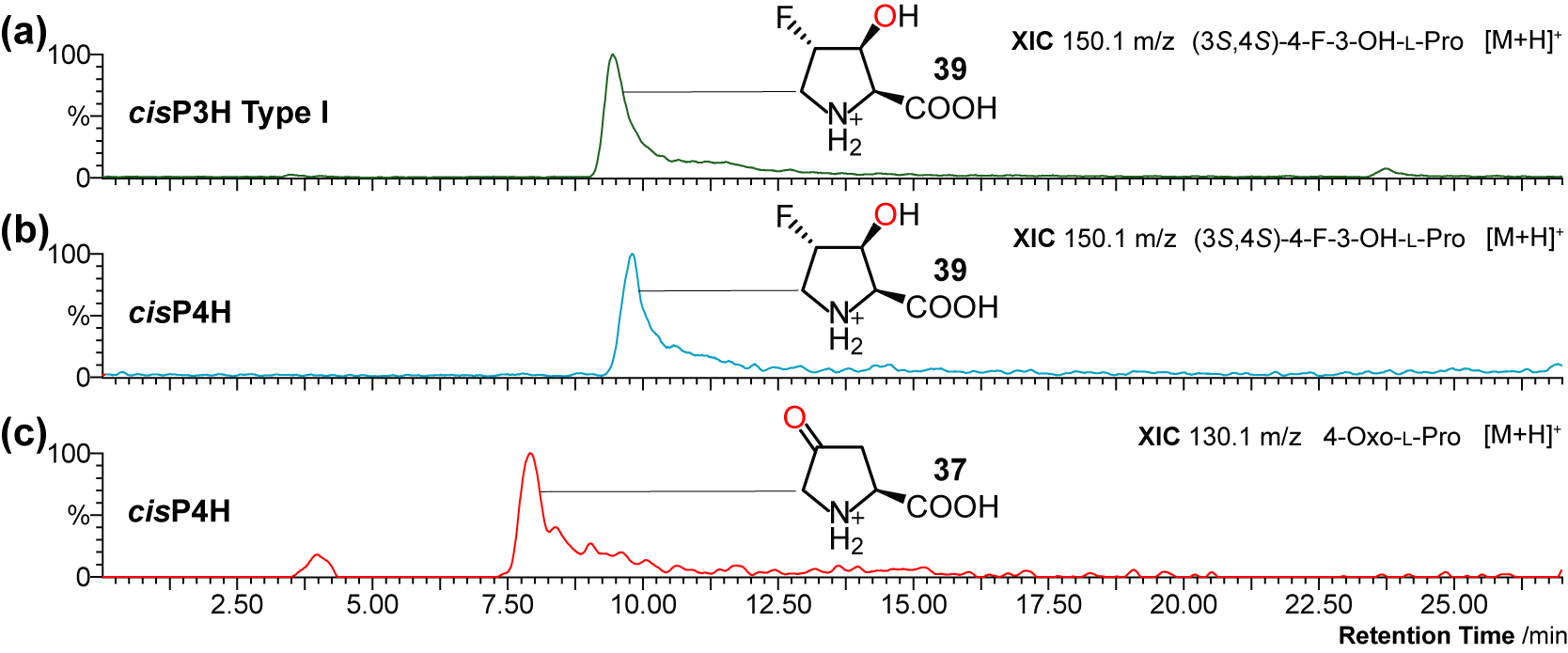


Fig. S58. Extracted-ion count (XIC) LC/MS chromatograms for the proline hydroxylase reactions using (2*S*,4*R*)-*trans*-4-fluoro-L-proline (*trans*-4-F-L-Pro) (38):

(a) *cis*P3H reactions yield (2*S*,3*S*,4*S*)-4-fluoro-3-hydroxy-L-proline (39);

(b) *cis*P4H reactions yield (2*S*,3*S*,4*S*)-4-fluoro-3-hydroxy-L-proline (39);

**(c)** *cis*P4H reactions yield (2*S*)-4-oxo-L-proline (**37**)**.**


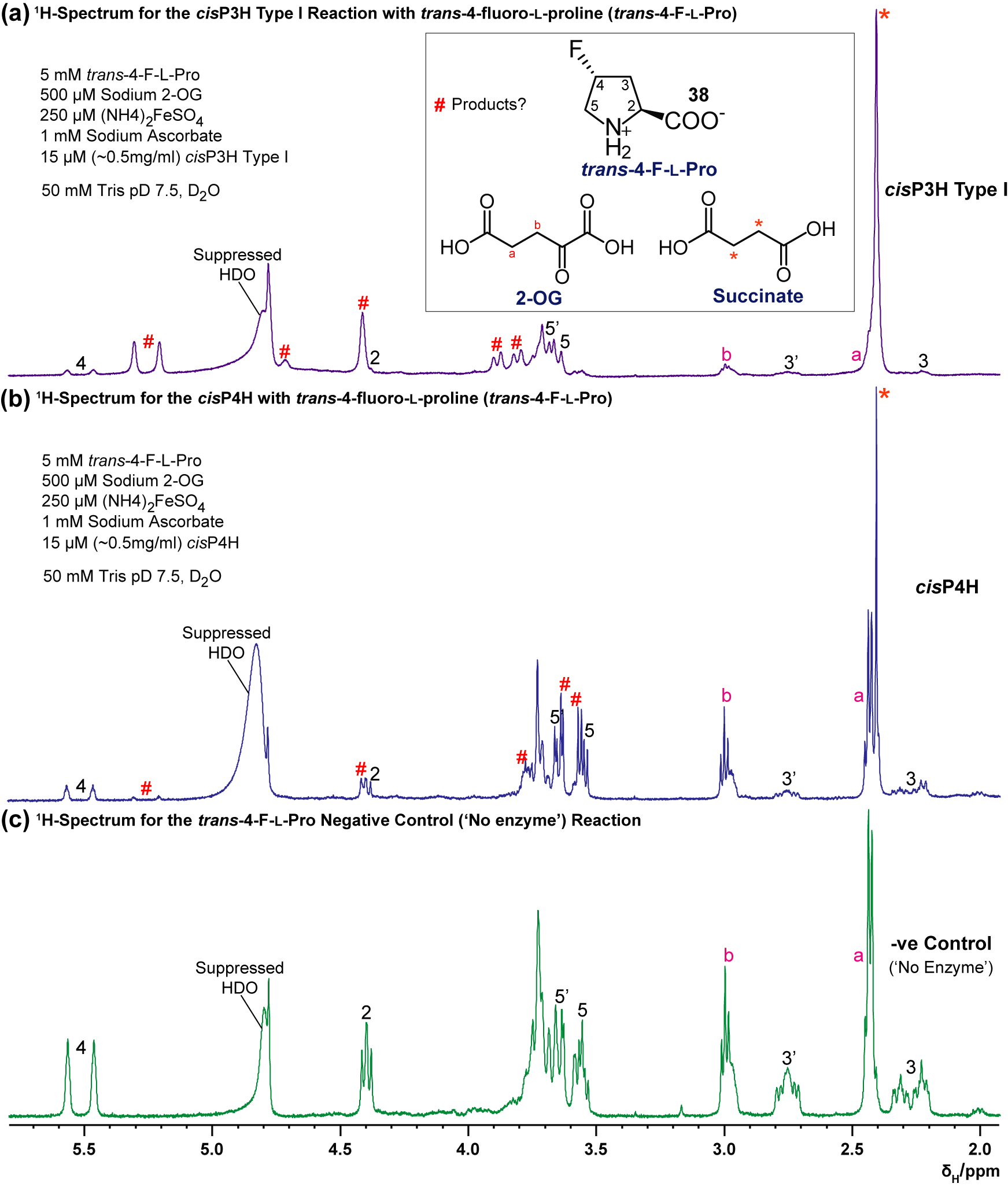


**Fig. S60.** ^1^H-NMR analyses of proline hydroxylase reactions using (2*S*,4*R*)-*trans*-4-fluoro-L-proline (*trans*-4-F-L-Pro) (**38**) [**(a)** *cis*P3H and **(b)** *cis*P4H].

Reactions were carried at room temperature (25 °C) for 14 h. Proton-decoupled ^19^F-spectra were measured at 298 K for samples in 5 mm tubes using a 500 MHz (^1^H) Bruker AVII 500 NMR spectrometer [equipped with a 5 mm TFI-^1^H/^19^F(^13^C) probe]. Chemical shift values are referenced to those of CFCl_3_ (where δ_F, CFCl3_ = ‘0.0 ppm’).


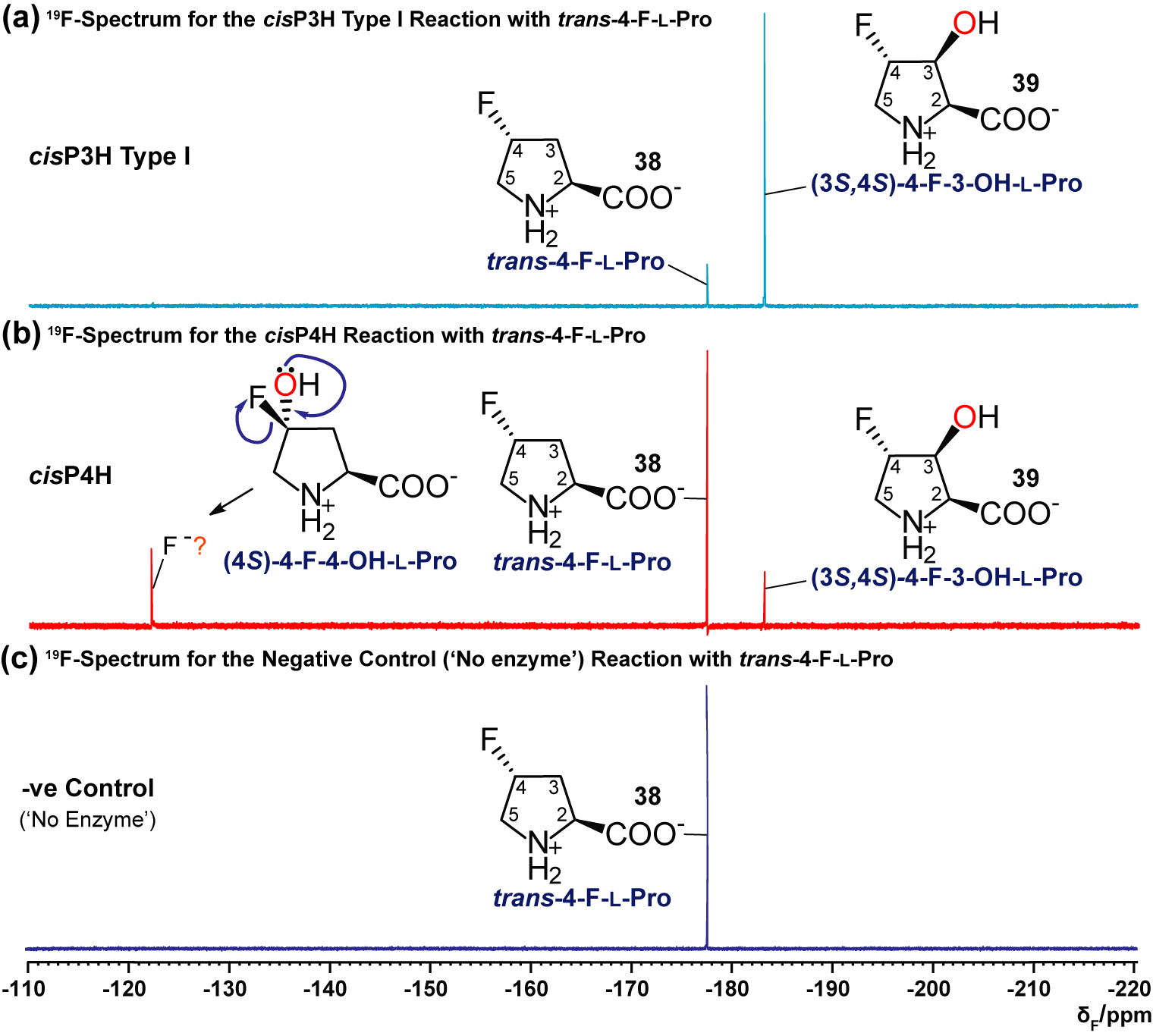


**Fig. S61.** Proton-decoupled ^19^F-NMR analyses of proline hydroxylase reactions using (2*S*,4*R*)-*trans*-4-fluoro-L-proline (*trans*-4-F-L-Pro) (**38**) [**(a)** *cis*P3H Type I and **(b)** *cis*P4H].

Proton-decoupled ^19^F-spectra were measured at 298 K for samples in 5 mm tubes using a 470.4 MHz (^19^F) Bruker AVII 500 NMR spectrometer [equipped with a 5 mm TFI-^1^H/^19^F(^13^C) probe]. Chemical shift values are referenced to those of CFCl_3_ (where δ_F, CFCl3_ = ‘0.0 ppm’).

Starting Material - ^19^F NMR (470 MHz, D_2_O) δ = -177.55 (s, 1F).

Hydroxylation Product - ^19^F NMR (470 MHz, D_2_O) δ = -183.27 (s, 1F).

Fluoride Released - ^19^F NMR (470 MHz, D_2_O) δ = -122.1 (s, 1F).


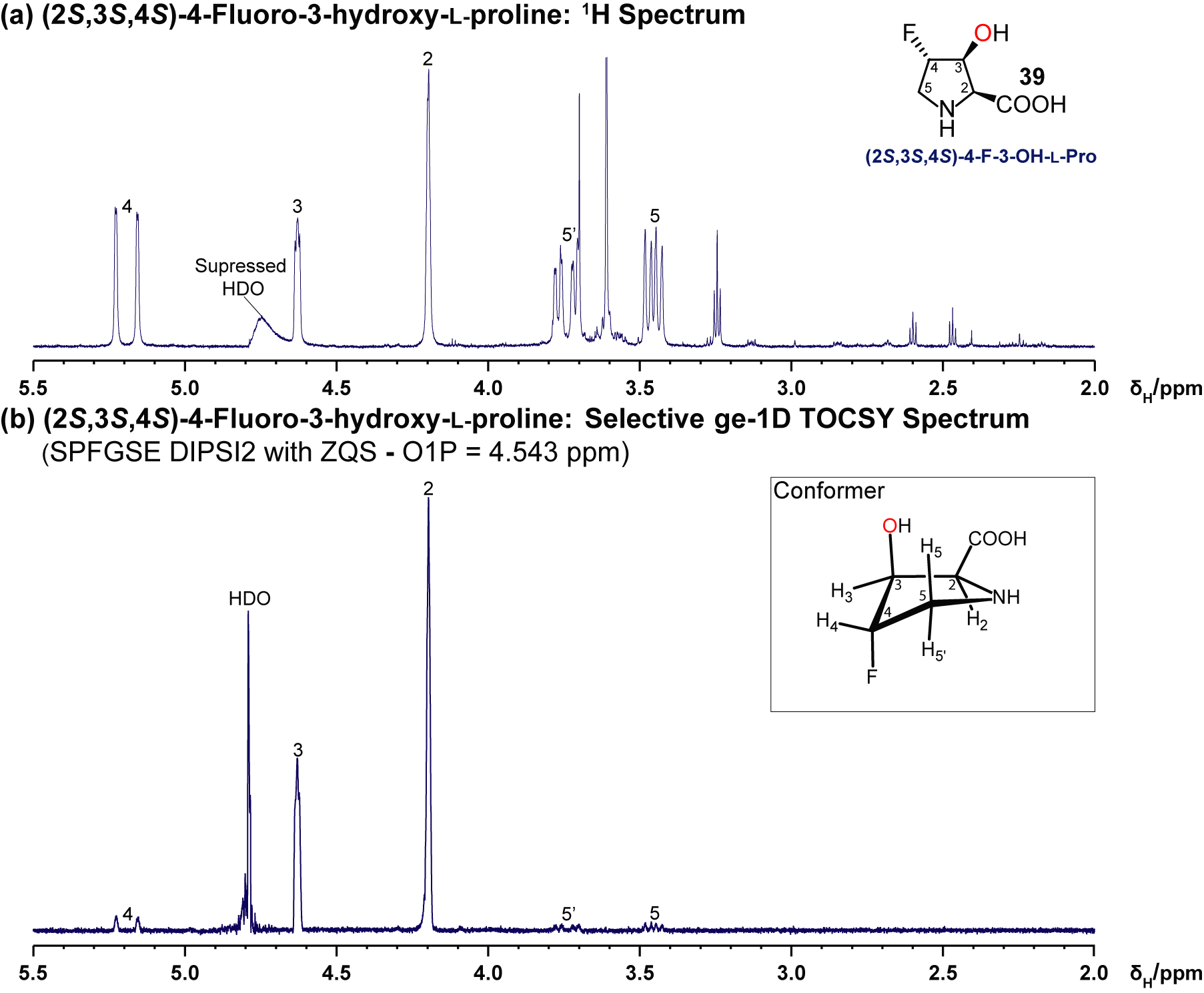


Fig. S62. NMR analyses of the (2*S*,3*S*,4*S*)-4-fluoro-3-hydroxy-L-proline (39) hydroxylation product from *cis*P3H reactions using (2*S*,4*R*)-*trans*-4-fluoro-L-proline (*trans*-4-F-L-Pro) (38): (a) ^1^H-NMR spectrum (‘zgpr’ pulse sequence) and (b) selective 1D ge-TOCSY (‘spfgsedipsi2zs’ pulse sequence). Chemical shift values are referenced to TSP-*d*_4_ (‘0.0 ppm’).

Assignments

^1^H NMR (700 MHz, D_2_O) δ = 5.19 (dddd, *J* = 49.7, 3.3, 0.8 Hz, 1H), 4.63 (ddd, *J* = 6.3, 2.8, 0.8 Hz, 1H), 4.20 (d, *J* = 2.8 Hz, 1H), 3.74 (ddd, *J* = 39.5, 13.9, 3.3 Hz, 1H), 3.46 (ddd, *J* = 25.0, 13.9, 0.8 Hz, 1H).


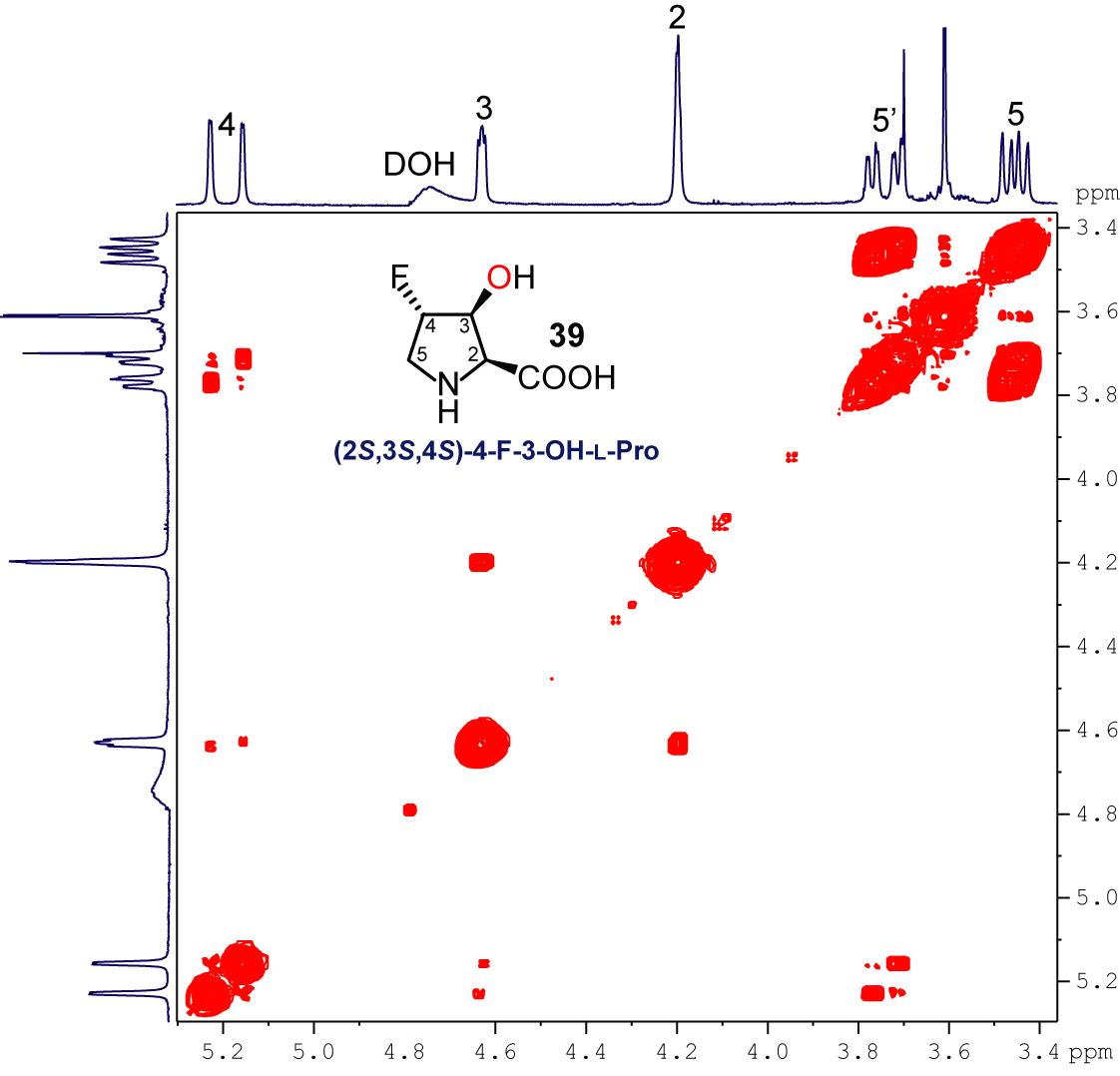

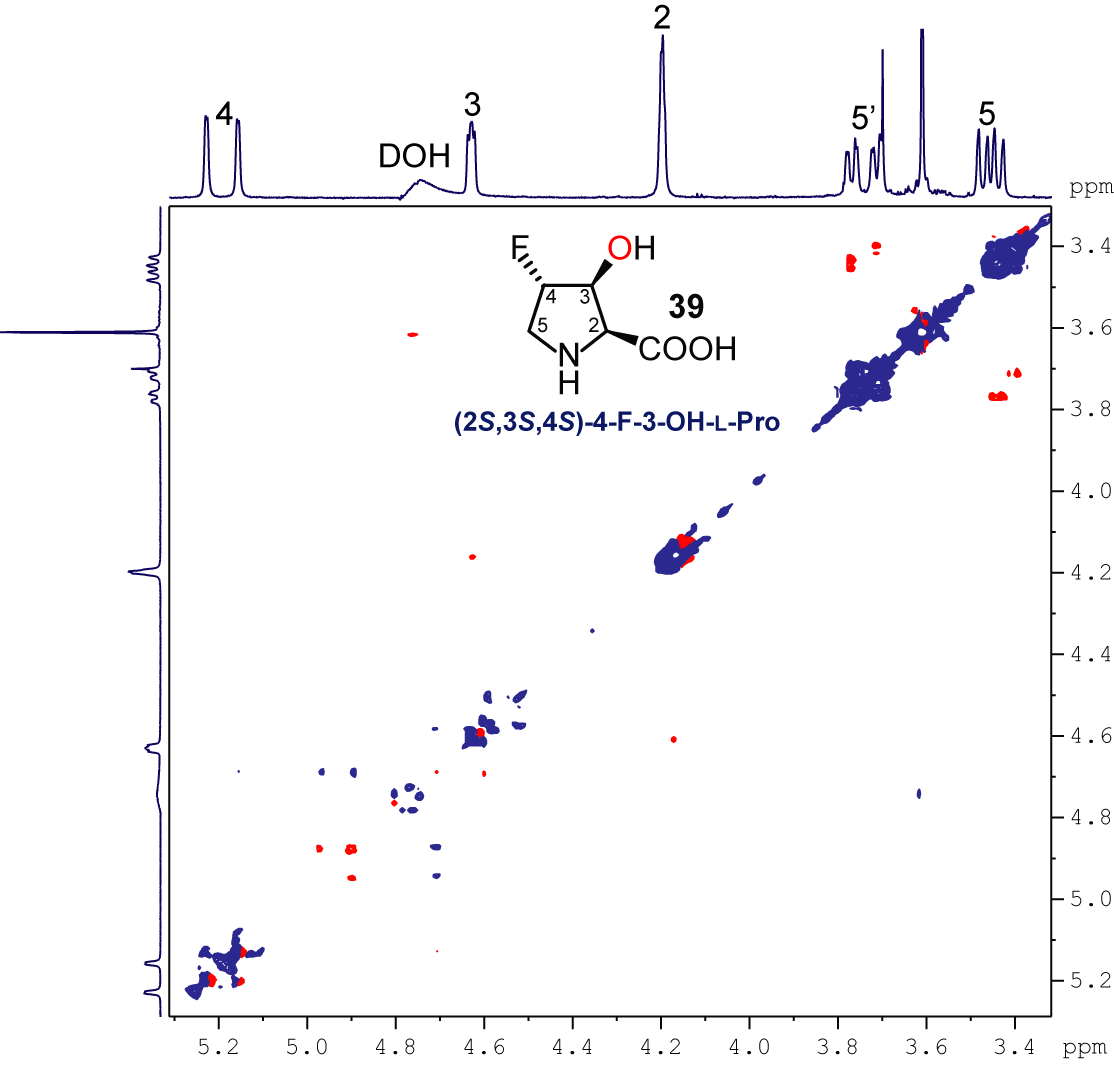


**Fig.** **S63.** 2D-NMR analyses of the (2*S*,3*S*,4*S*)-4-fluoro-3-hydroxy-L-proline (**39**) hydroxylation product from *cis*P4H reactions using (2*S*,4*R*)-*trans*-4-fluoro-L-proline (*trans*-4-F-L-Pro) (**38**):

(a) ^1^H-^1^H COSY spectrum (‘cosygpprf2qf’ pulse sequence);

**(b)** ^1^H-^1^H NOESY spectrum (‘noesyphprf2’ pulse sequence).

Chemical shift values are referenced to TSP-*d*_4_ (‘0.0 ppm’).

**(a)**

**(b)**

1. *(2S,3S)-trans-3-Hydroxy-L-proline (trans-3-Hyp) (****40****)*

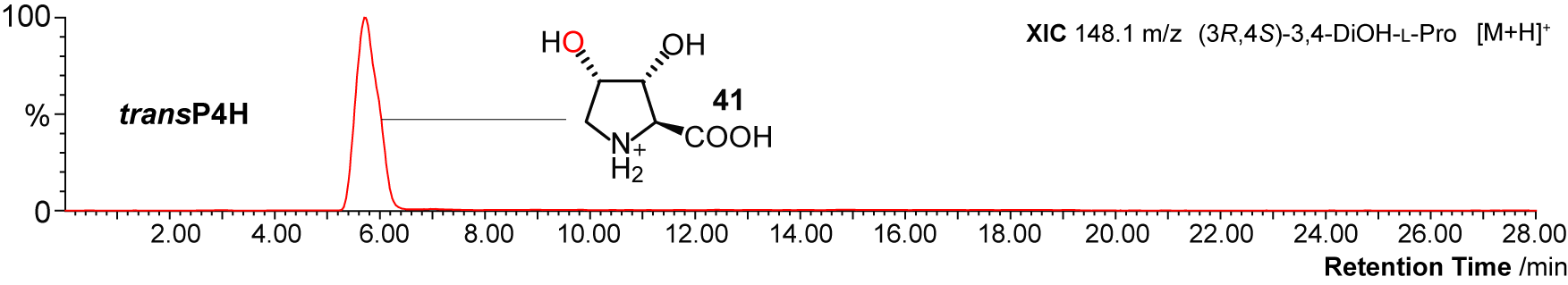


Fig. S64. Extracted-ion count (XIC) LC/MS chromatograms for the proline hydroxylase reactions using (2*S*,3*S*)-*trans*-3-hydroxy-L-proline (*trans*-3-Hyp) (40): *trans*P4H catalyses the production of (2*S*,3*R*,4*S*)-3,4-dihydroxy-L-proline (41).

Scheme S11. Proline hydroxylase reactions using (2*S*,3*S*)-*trans*-3-hydroxy-L-proline (*trans*-3-Hyp) (40): *trans*P4H catalyses the production of (2*S*,3*R*,4*S*)-3,4-dihydroxy-L-proline (41).


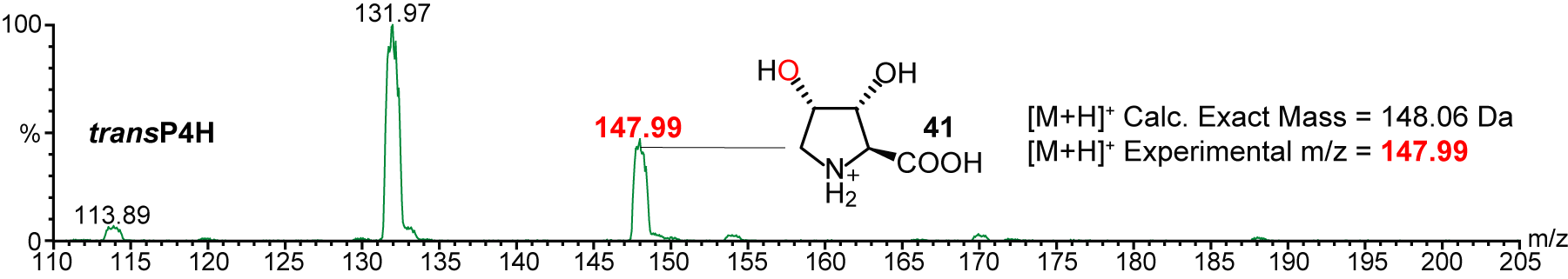


Fig. S65. LC/MS spectra of proline hydroxylase reactions using (2*S*,3*S*)-*trans*-3-hydroxy-L-proline (*trans*-3-Hyp) (40): *trans*P4H catalyses the production of (2*S*,3*R*,4*S*)-3,4-dihydroxy-L-proline (41).

Stereochemical assignments were made by NMR.


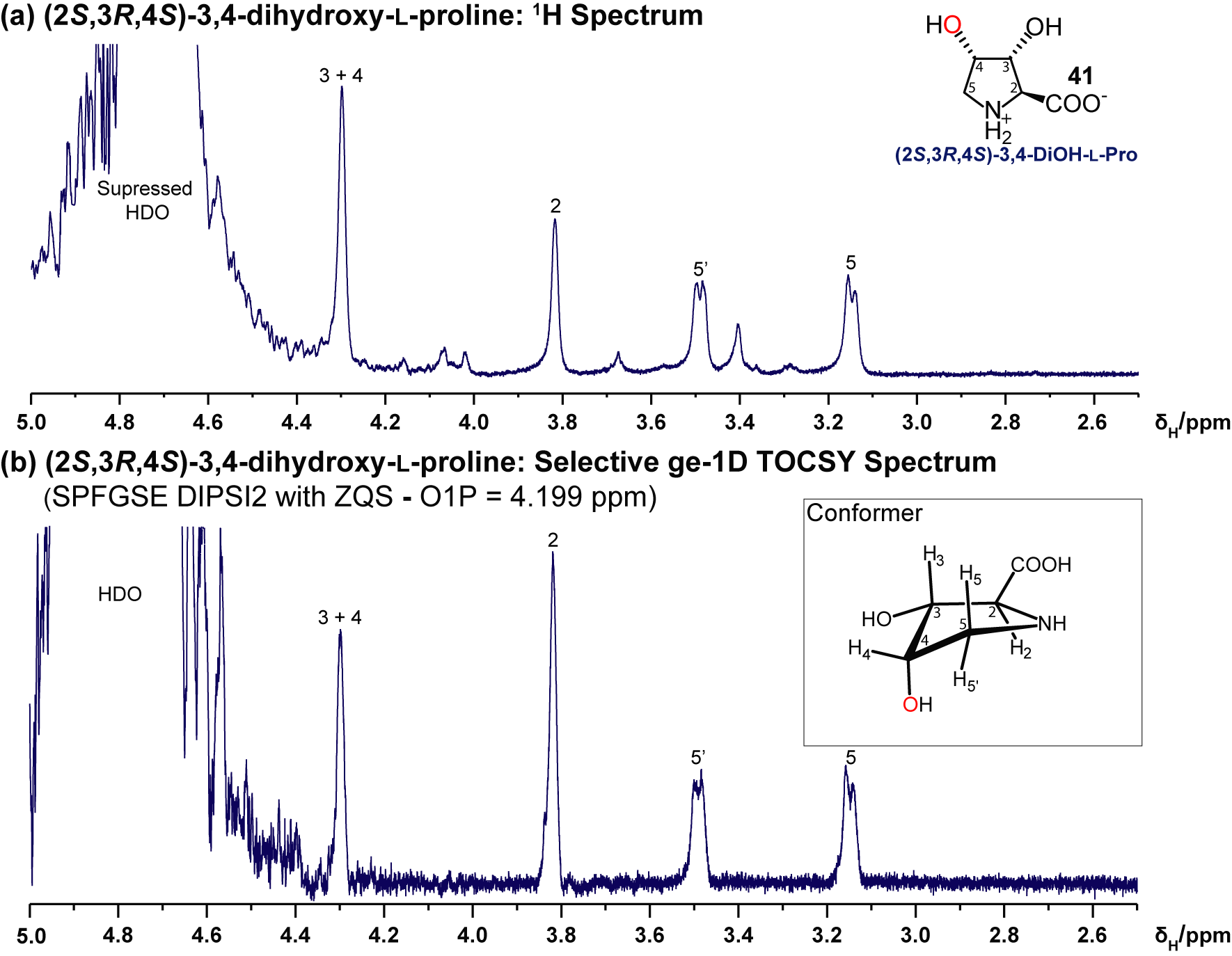


Fig. S66. NMR analyses of the (2*S*,3*R*,4*S*)-3,4-dihydroxy-L-proline (41) hydroxylation product from *cis*P3H reactions using a (2*S*,3*S*)-*trans*-3-hydroxy-L-proline (*trans*-3-Hyp) (40): (a) ^1^H-NMR spectrum (‘zgpr’ pulse sequence) and (b) selective 1D ge-TOCSY (‘spfgsedipsi2zs’ pulse sequence). Chemical shift values are referenced to TSP-*d*_4_ (‘0.0 ppm’).

Assignments

^1^H NMR (700 MHz, D_2_O) δ = 4.33 – 4.27 (m, 2H), 3.82 (d, *J* = 3.6 Hz, 1H), 3.49 (dd, *J* =
12.1, 4.5 Hz, 1H), 3.15 (dd, *J* = 12.1, 3.5 Hz, 1H).


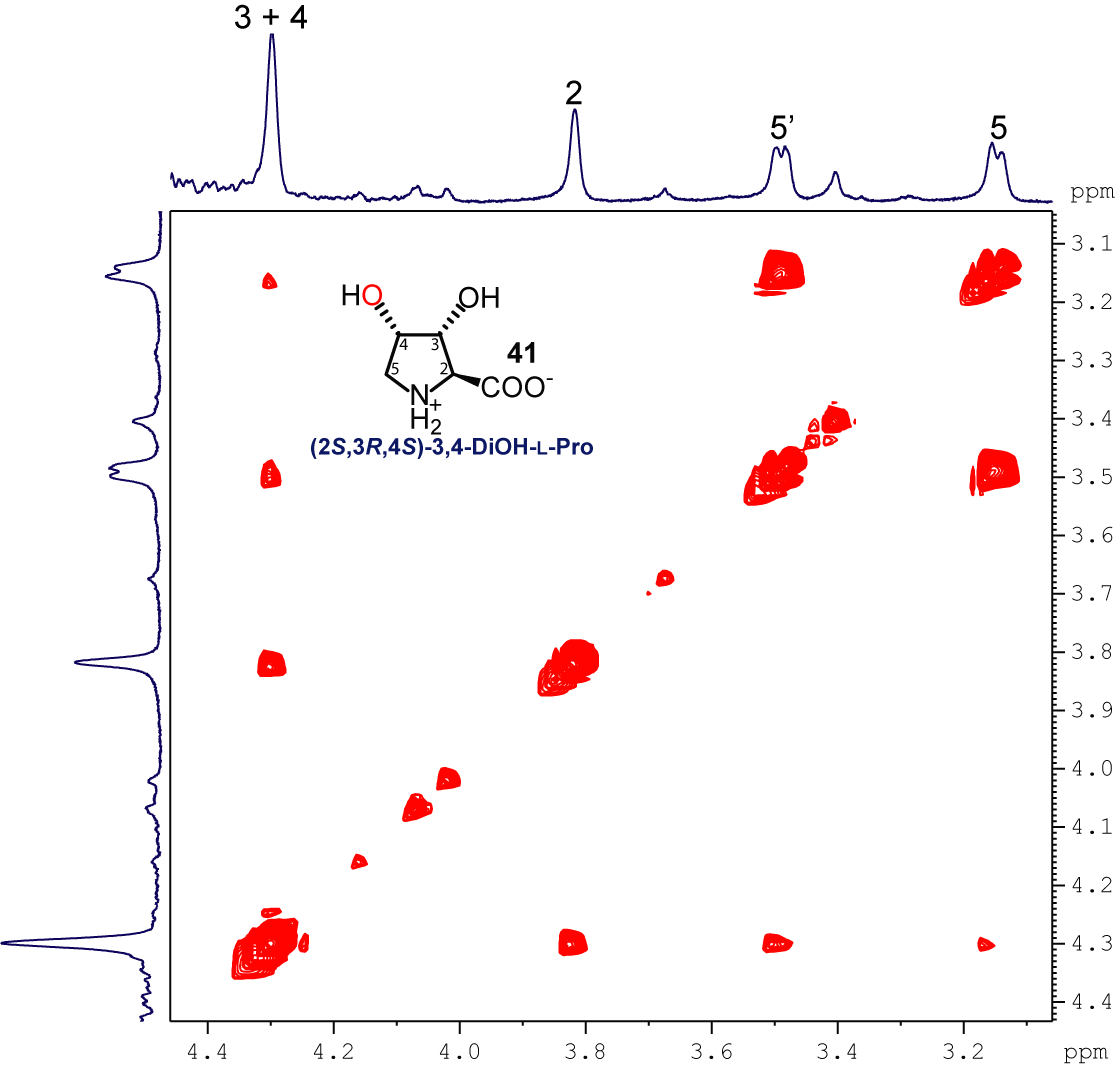

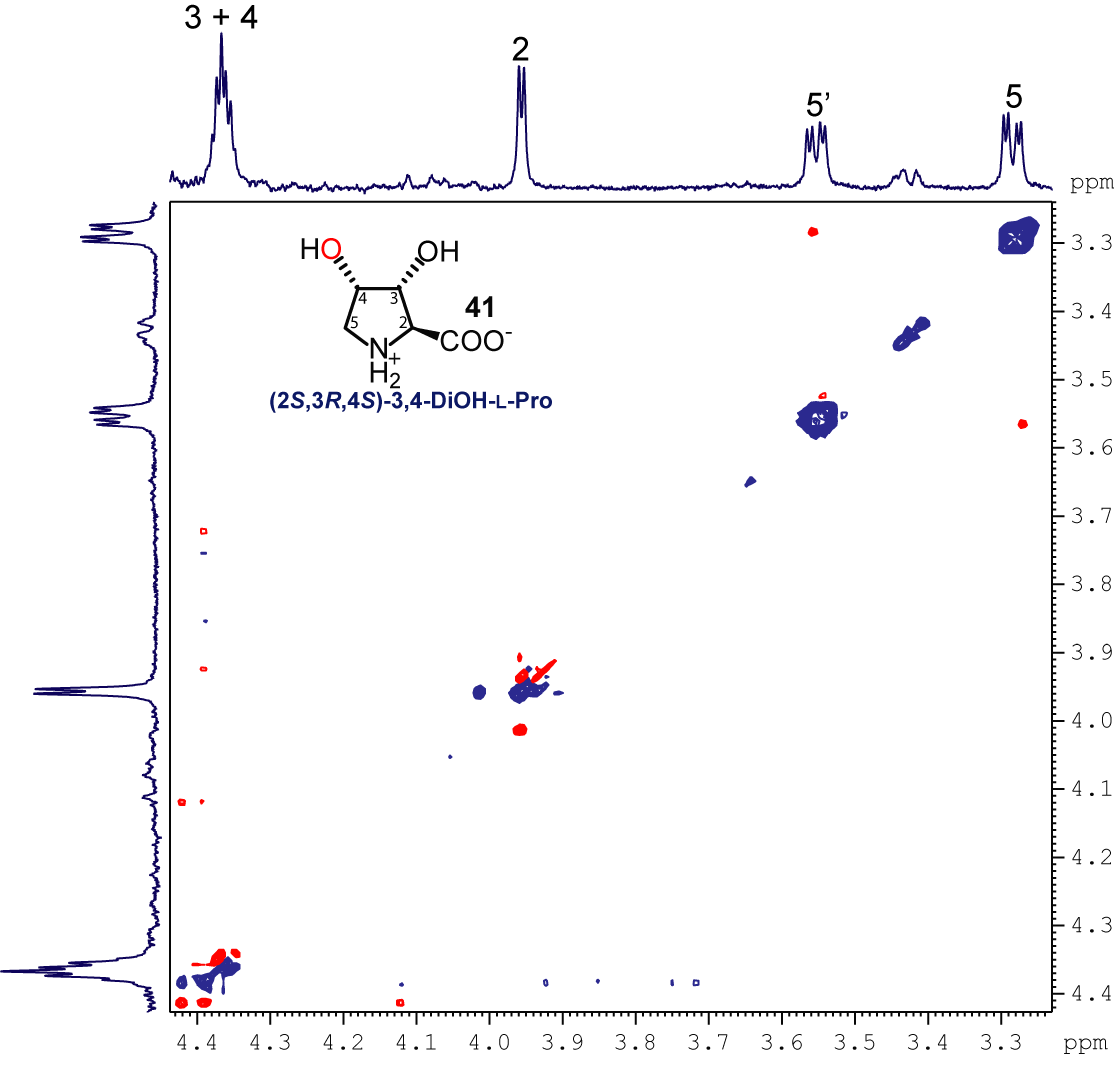


**Fig. S67.** 2D-NMR analyses of the (2*S*,3*R*,4*S*)-3,4-dihydroxy-L-proline (**41**) hydroxylation product from *cis*P4H reactions using (2*S*,3*S*)-*trans*-3-hydroxy-L-proline (*trans*-3-Hyp) (**40**):

(a) ^1^H-^1^H COSY spectrum (‘cosygpprf2qf’ pulse sequence);

**(b)** ^1^H-^1^H NOESY spectrum (‘noesyphprf2’ pulse sequence).

Chemical shift values are referenced to TSP-*d*_4_ (‘0.0 ppm’).

**(a)**

**(b)**

1. *(2S,4S)-cis-4-Hydroxy-L-proline (cis-4-Hyp) (****3****)*

Scheme S12. Proline hydroxylase reactions using (2*S*,4*S*)-*cis*-4-hydroxy-L-proline (*cis*-4-Hyp) (3): *cis*P3H catalyses production of (2*S*,3*S*,4*R*)-3,4-dihydroxy-L-proline (42).

**
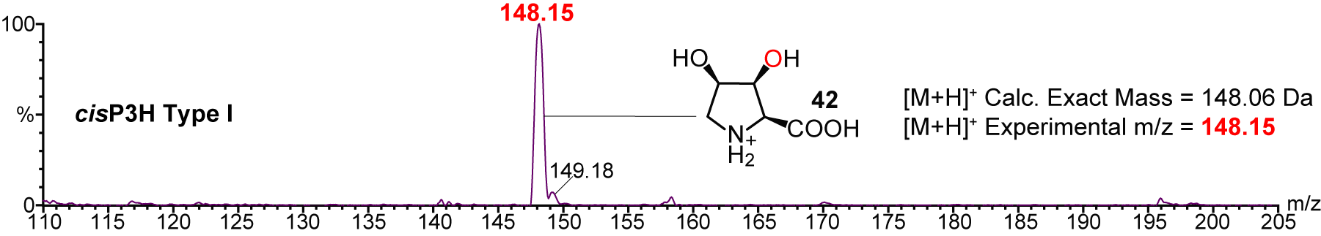
**

Fig. S69. LC/MS spectra of proline hydroxylase reactions using (2*S*,4*S*)-*cis*-4-hydroxy-L-proline (*cis*-4-Hyp) (3): *trans*P4H reactions produce (2*S*,3*S*,4*R*)-3,4-dihydroxy-L-proline (42).

Stereochemical assignments were made by NM**R.**


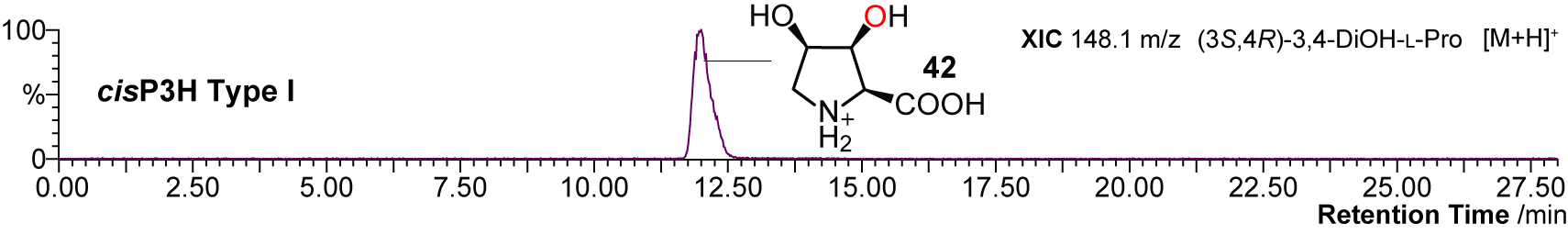


Fig. S68. Extracted-ion count (XIC) LC/MS chromatograms for the proline hydroxylase reactions using (2*S*,4*S*)-*cis*-4-hydroxy-L-proline (*cis*-4-Hyp) (3): *trans*P4H reactions produce (2*S*,3*S*,4*R*)-3,4-dihydroxy-L-proline (42).


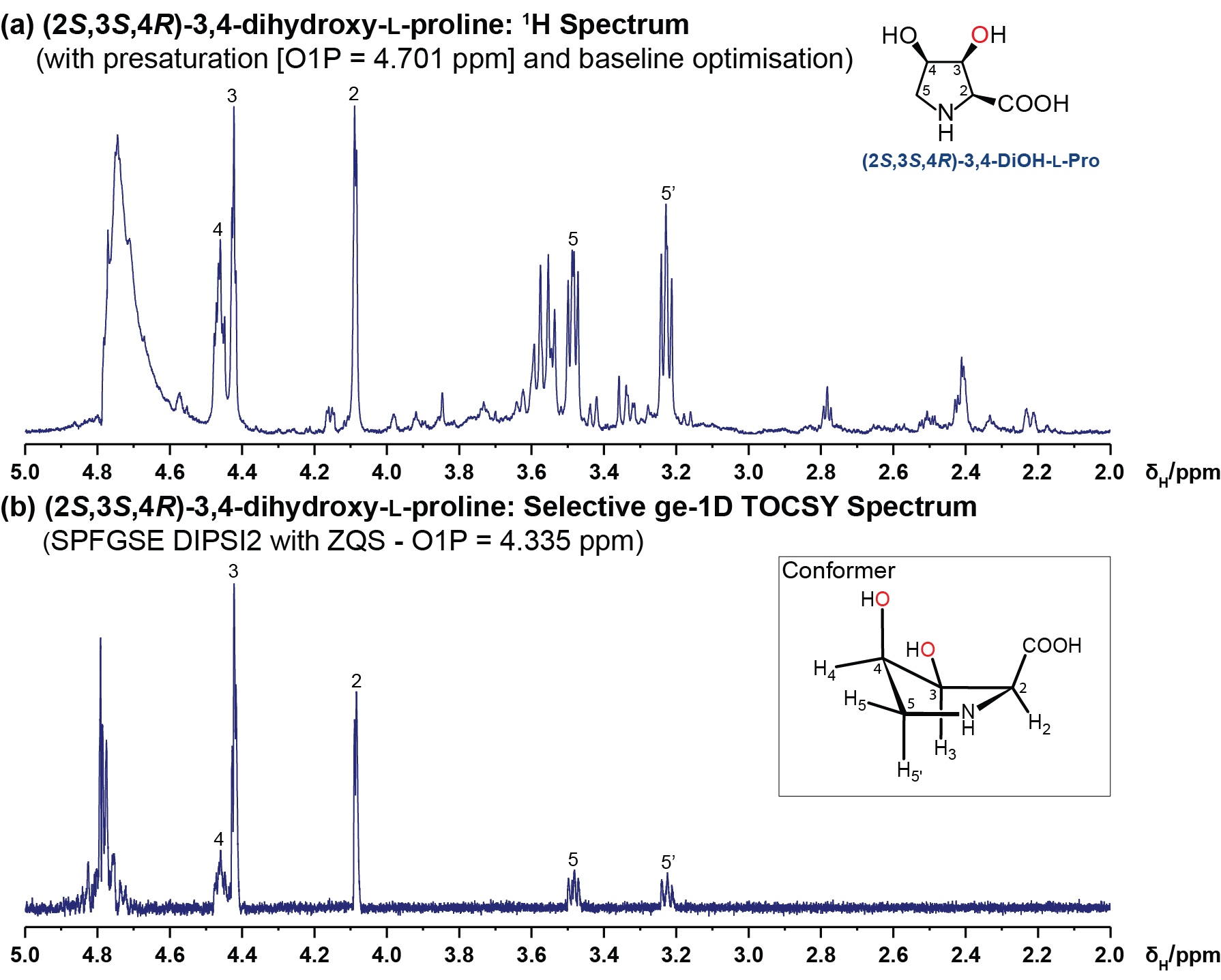


Fig. S70. NMR analyses of the (2*S*,3*S*,4*R*)-3,4-dihydroxy-L-proline (42) hydroxylation product from *cis*P3H reactions using (2*S*,4*S*)-*cis*-4-hydroxy-L-proline (*cis*-4-Hyp) (3): (a) ^1^H-NMR spectrum (‘zgpr’ pulse sequence) and (b) selective 1D ge-TOCSY (‘spfgsedipsi2zs’ pulse sequence). Chemical shift values are referenced to TSP-*d*_4_ (‘0.0 ppm’).

Assignments

^1^H NMR (700 MHz, D_2_O) δ = 4.46 (ddd, *J* = 8.7, 7.6, 3.9 Hz, 1H), 4.42 (dd, *J* = 5.3, 3.9, 3.8 Hz, 1H), 4.09 (d, *J* = 3.8 Hz, 1H), 3.49 (dd, *J* = 11.5, 7.6 Hz, 1H), 3.23 (dd, *J* = 11.5, 8.7 Hz, 1H).

(a)

**
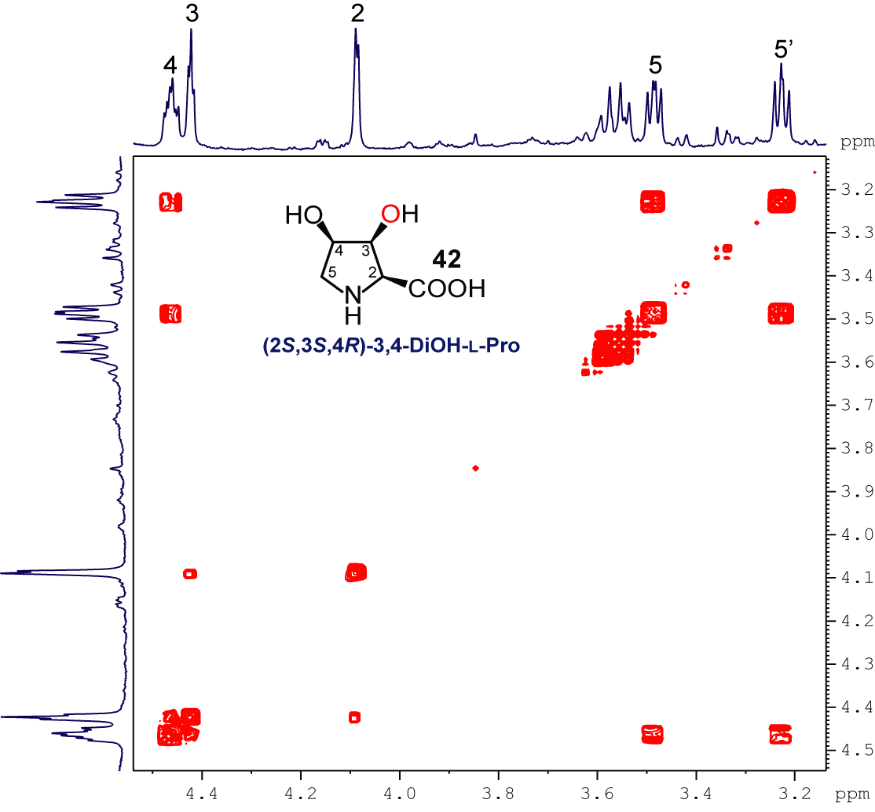
**

(b)

**
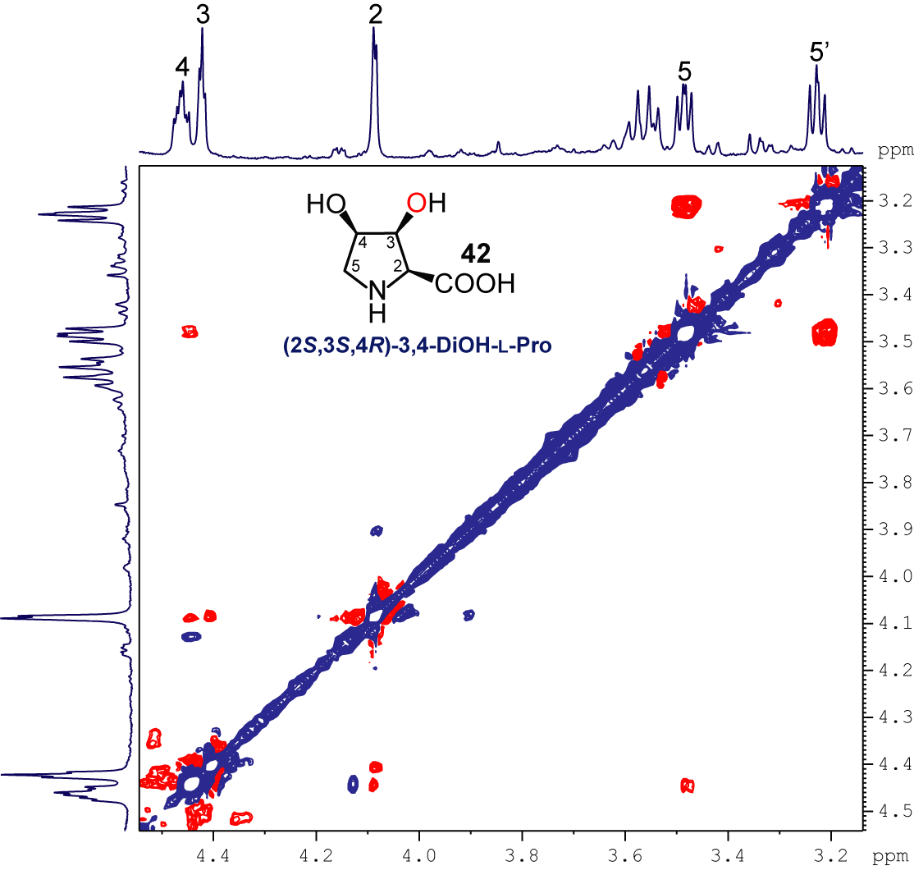
**

**Fig.** **S71.** 2D-NMR analyses of the (2*S*,3*S*,4*R*)-3,4-dihydroxy-L-proline (**42**) hydroxylation product from *cis*P4H reactions using (2*S*,4*S*)-*cis*-4-hydroxy-L-proline (*cis*-4-Hyp) (**3**):

(a) ^1^H-^1^H COSY spectrum (‘cosygpprf2qf’ pulse sequence);

**(b)** ^1^H-^1^H NOESY spectrum (‘noesyphprf2’ pulse sequence).

Chemical shift values are referenced to TSP-*d*_4_ (‘0.0 ppm’).

1. *(2S,4S)-cis-4-Hydroxy-N-methyl-L-proline (cis-4-OH-N-Me-Pro) (****20****)*

Scheme S13. Proline hydroxylase reactions using a (2*S*,4*S*)-*cis*-4-hydroxy-*N*-methyl-L-proline (*cis*-4-OH-*N*-Me-Pro) (20): *cis*P3H catalyses the production of (2*S*,3*S*,4*R*)-3,4-dihydroxy-*N*-methyl-L-proline (43).


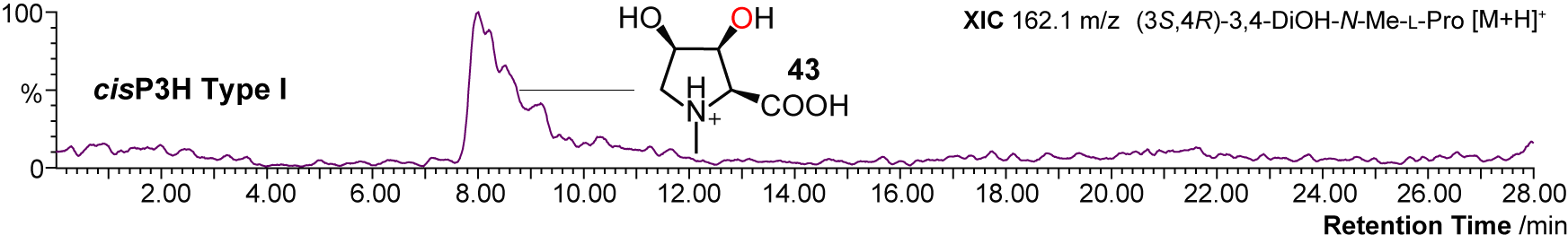


Fig. S72. Extracted-ion count (XIC) LC/MS chromatograms for the proline hydroxylase reactions using (2*S*,4*S*)-*cis*-4-hydroxy-*N*-methyl-L-proline (*cis*-4-OH-*N*-Me-Pro) (20): *trans*P4H reactions produce (2*S*,3*S*,4*R*)-3,4-dihydroxy-*N*-methyl-L-proline (43).


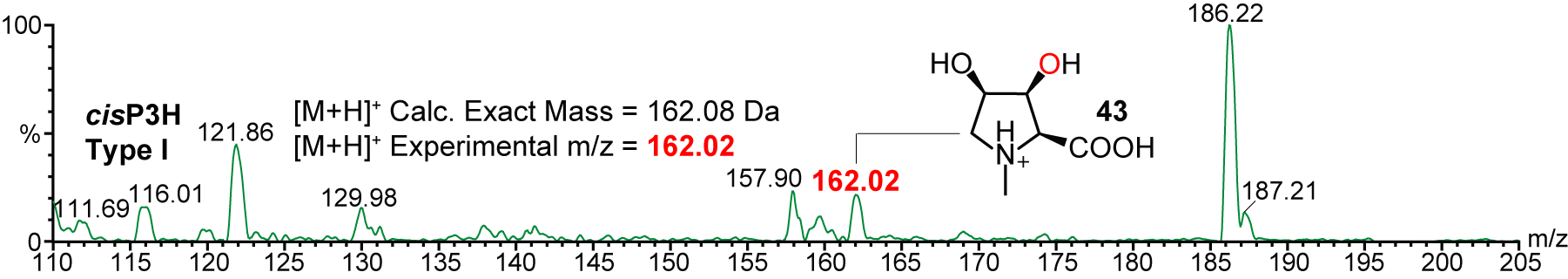


Fig. S73. LC/MS spectra of proline hydroxylase reactions using (2*S*,4*S*)-*cis*-4-hydroxy-*N*-methyl-L-proline (*cis*-4-OH-*N*-Me-Pro) (20): *trans*P4H reactions produce (2*S*,3*S*,4*R*)-3,4-dihydroxy-*N*-methyl-L-proline (43).

Stereochemical assignments were made by NMR.

1. *(2S,4R)-trans-4-Hydroxy-L-proline (trans-4-Hyp) (****4****)*

Scheme S14. Proline hydroxylase reactions using a (2*S*,4*R*)-*trans*-4-hydroxy-L-proline (*trans*-4-OH-Pro) (4):

1. *cis*P3H and (b) *cis*P4H are proposed to produce the dihydroxylation product, (2*S*)-3,4,4‘-trihydroxy-L-proline (44), which can tautomerise to hydroxyketoproline species, i.e (2*S*,3*S*)-*cis*-3-hydroxy-4-oxo-L-proline (45) and (2*S*,3*R*)-*trans*-3-hydroxy-4-oxo-L-proline (46).


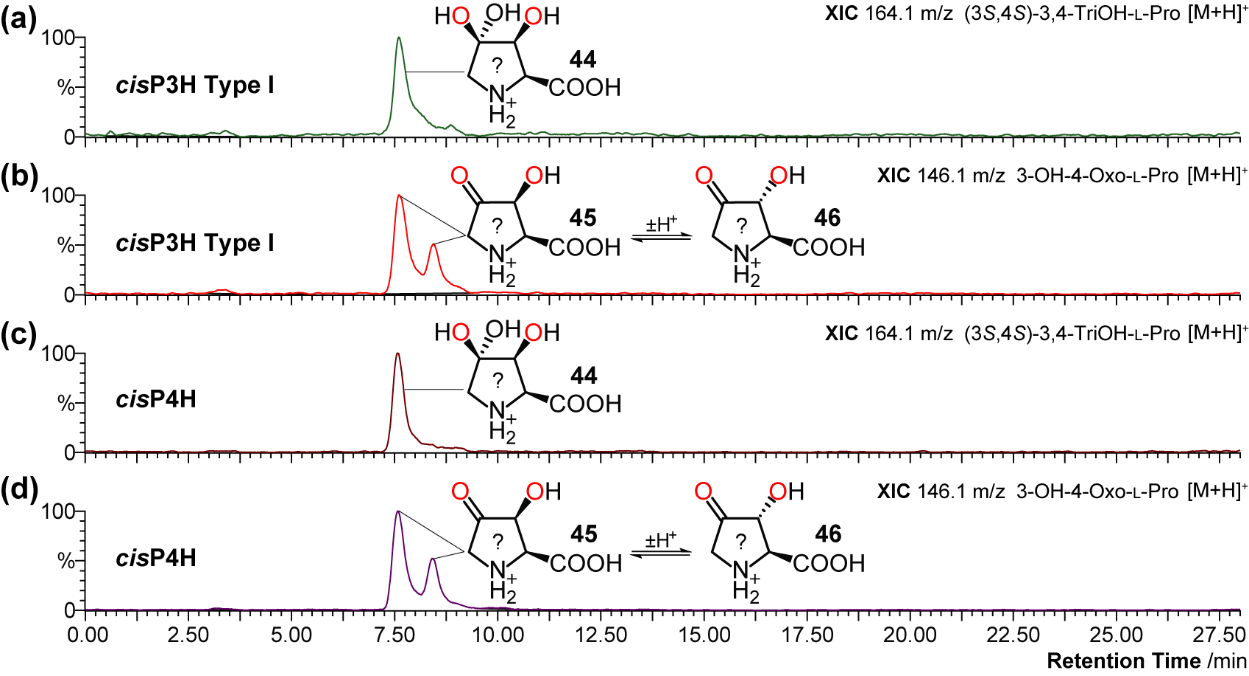


Fig. S74. Extracted-ion count (XIC) LC/MS chromatograms for the proline hydroxylase reactions using (2*S*,4*R*)-*trans*-4-hydroxy-L-proline (4):

(a) *cis*P3H reactions are proposed to yield a dihydroxylation product, (2*S*)-3,4,4‘-trihydroxy-L-proline (44), which can tautomerise to (b) hydroxyketoproline species, i.e. (2*S*,3*S*)-*cis*-3-hydroxy-4-oxo-L-proline (45) and (2*S*,3*R*)-*trans*-3-hydroxy-4-oxo-L-proline (46);

(c) *cis*P4H reactions are proposed to yield the dihydroxylation product, (2*S*)-3,4,4‘-trihydroxy-L-proline (44), which can tautomerise to (d) hydroxyketoproline species, i.e. (2*S*,3*S*)-*cis*-3-hydroxy-4-oxo-L-proline (45) and (2*S*,3*R*)-*trans*-3-hydroxy-4-oxo-L-proline (46).

Stereo chemical assignments were made by NMR.


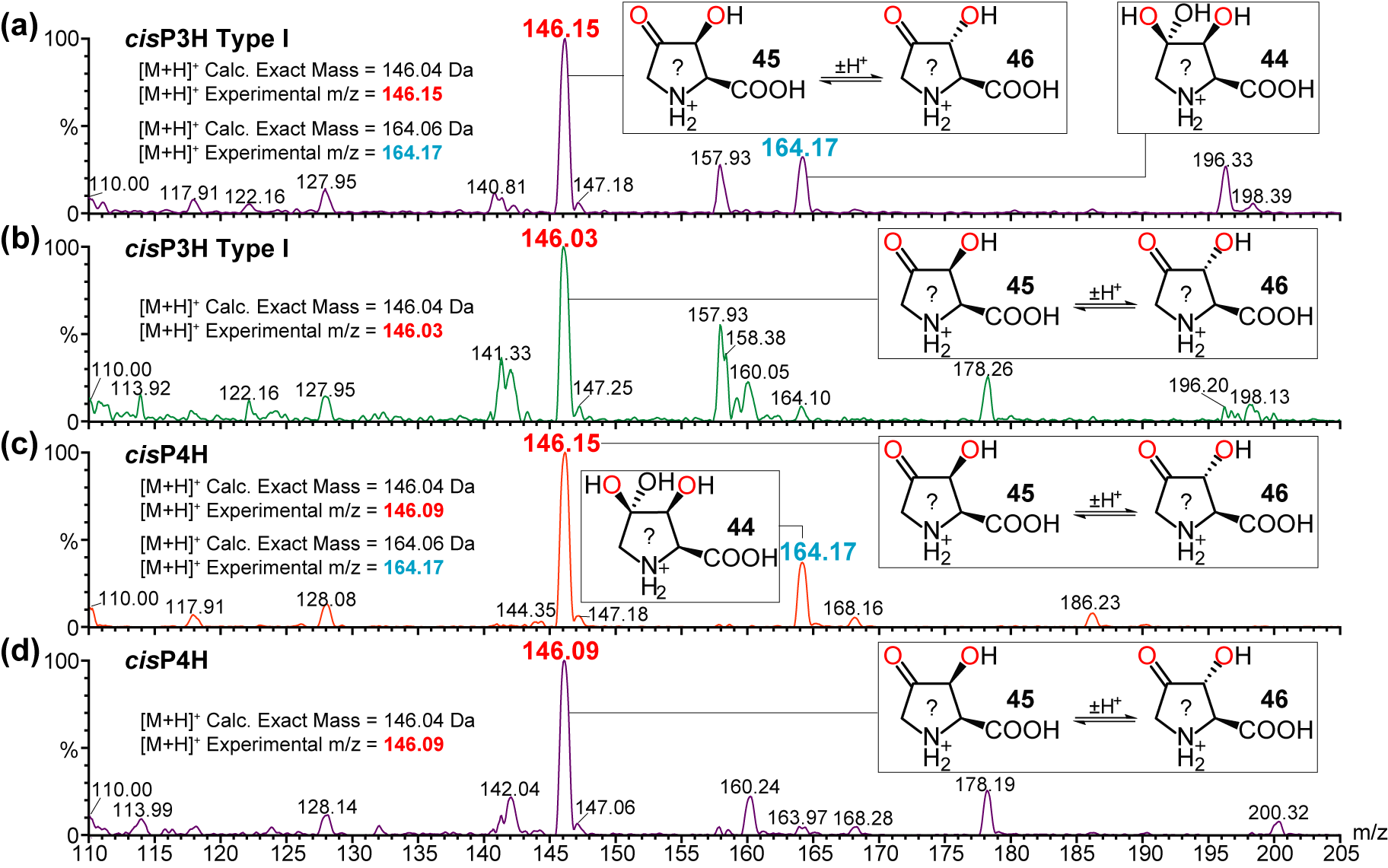


Fig. S75. LC/MS spectra of proline hydroxylase reactions using (2*S*,4*R*)-*trans*-4-hydroxy-L-proline (4):

(a) *cis*P3H reactions yield a (2*S*)-3-hydroxy-4-oxo-L-proline (45) [proposed to be (2*S*,3*S*)-*cis*-3-hydroxy-4-oxo-L-proline] and a (2*S*)-3,4,4‘-trihydroxy-L-proline (44);

**(b)** *cis*P3H reactions yield another hydroxyketoproline species (**46**) [proposed to be (2*S*,3*R*)-*trans*-3-hydroxy-4-oxo-L-proline];

(c) *cis*P4H reactions yield a (2*S*)-3-hydroxy-4-oxo-L-proline (45) [proposed to be (2*S*,3*S*)-*cis*-3-hydroxy-4-oxo-L-proline] and a (2*S*)-3,4,4‘-trihydroxy-L-proline (44);

**(d)** *cis*P4H reactions yield another hydroxyketoproline species (**46**) [proposed to be (2*S*,3*R*)-*trans*-3-hydroxy-4-oxo-L-proline].

Stereochemical assignments were made by NMR.

## *Bicyclic Substrate Analogues*

1. *(2S,3aS,6aS)-Octahydrocyclopenta[b]pyrrole-2-carboxylic Acid (****47****)*

Scheme S15. Proline hydroxylase reactions using (2*S*,3a*S*,6a*S*)-octahydrocyclopenta[*b*]pyrrole-2-carboxylic acid (47):

(a) *cis*P3H catalyses the production of (2*S*,3a*S*,4*R*,6a*S*)-*cis*-4-hydroxy-octahydrocyclopenta[*b*]pyrrole-2-carboxylic acid (48) and (2*S*,3*R*,3a*R*,6a*S*)-*cis*-3-hydroxy-octahydrocyclopenta[*b*]pyrrole-2-carboxylic acid (49);

(b) *cis*P4H catalyses the production of (2*S*,3a*S*,4*R*,6a*S*)-*cis*-4-hydroxy-octahydrocyclopenta[*b*]pyrrole-2-carboxylic acid (48);

**(c)** *trans*P4H catalyses the production of a single hydroxylation product, which could be (2*S*,3a*S*,4*S*,6a*S*)-4-hydroxy-octahydropenta[*b*]pyrrole-2-carboxylic acid (**50**).


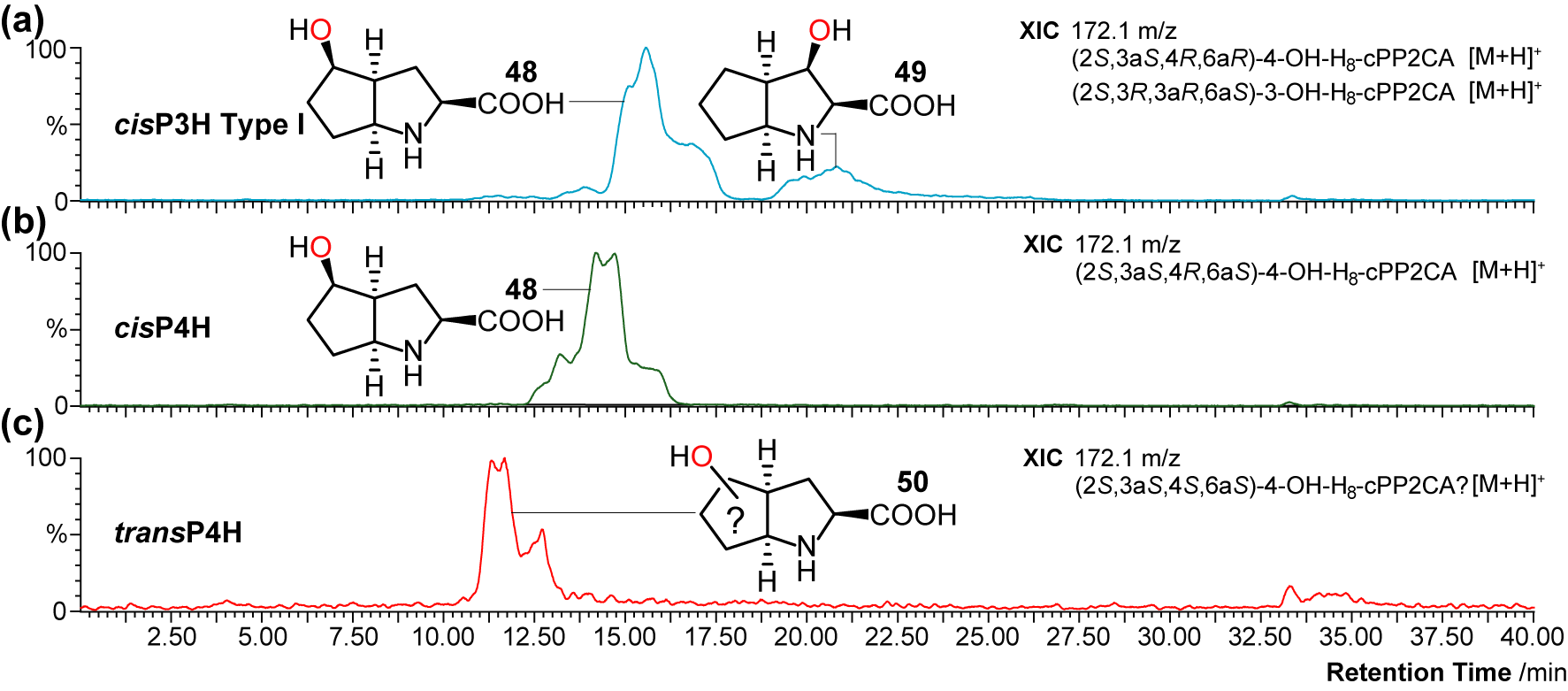


Fig. S76. Extracted-ion count (XIC) LC/MS chromatograms for the proline hydroxylase reactions using (2*S*,3a*S*,6a*S*)-octahydrocyclopenta[*b*]pyrrole-2-carboxylic acid (47):

(a) *cis*P3H reactions yield (2*S*,3a*S*,4*R*,6a*S*)-*cis*-4-hydroxy-octahydrocyclopenta[*b*]pyrrole-2-carboxylic acid (48) and (2*S*,3*R*,3a*R*,6a*S*)-*cis*-3-hydroxy-octahydrocyclopenta[*b*]pyrrole-2-carboxylic acid (49);

(b) *cis*P4H reactions yield (2*S*,3a*S*,4*R*,6a*S*)-*cis*-4-hydroxy-octahydrocyclopenta[*b*]pyrrole-2-carboxylic acid (48);

**(c)** *trans*P4H reactions yield a single hydroxylation product (**50**) [proposed to be (2*S*,3a*S*,4*S*,6a*S*)-4-hydroxy-octahydropenta[*b*]pyrrole-2-carboxylic acid].

Stereochemical assignments were made by NMR.


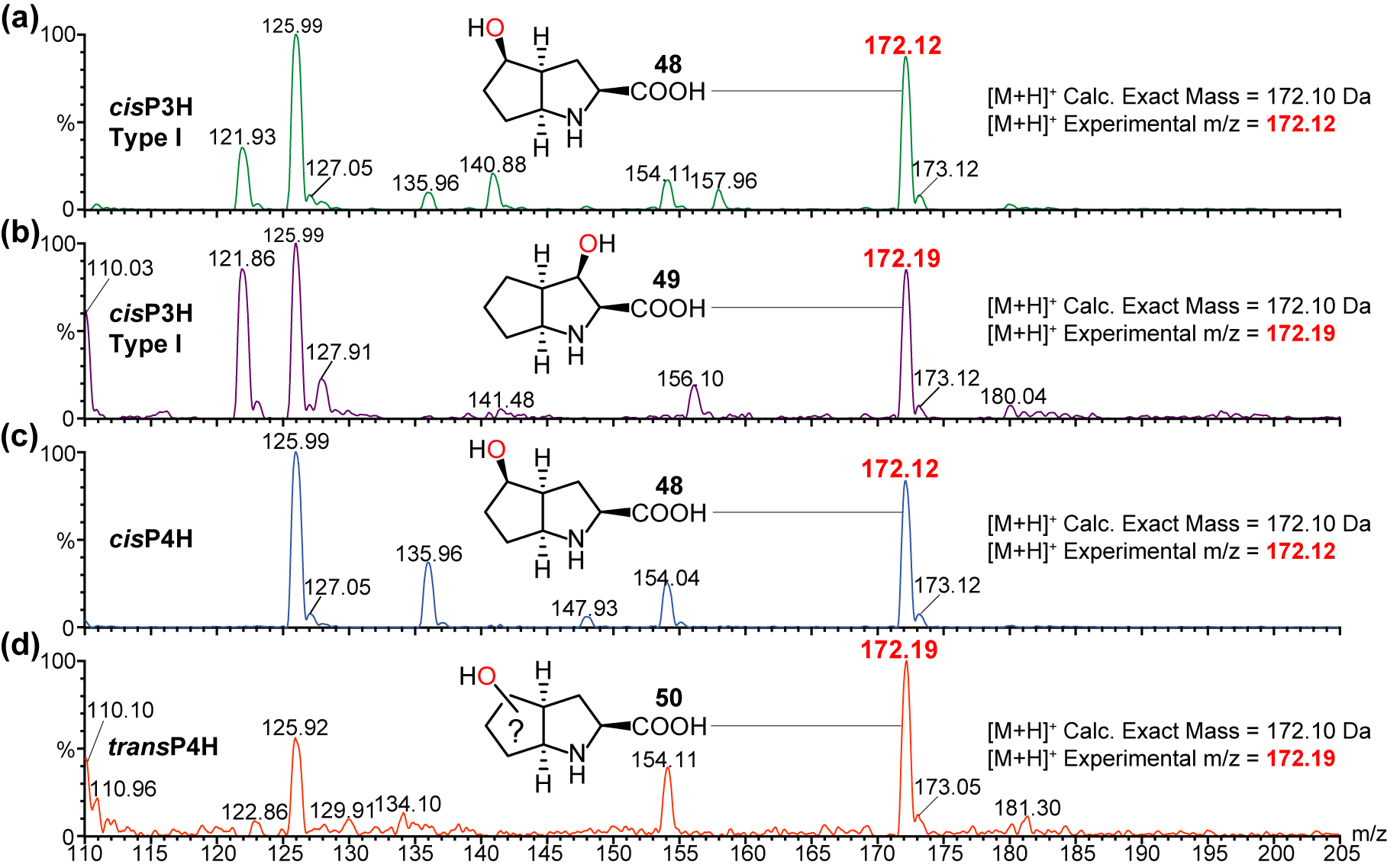


Fig. S77. LC/MS spectra of proline hydroxylase reactions using (2*S*,3a*S*,6a*S*)-octahydrocyclopenta[*b*]pyrrole-2-carboxylic acid (47):

(a) *cis*P3H reactions yield (2*S*,3a*S*,4*R*,6a*S*)-*cis*-4-hydroxy-octahydrocyclopenta[*b*]pyrrole-2-carboxylic acid (48) and (2*S*,3*R*,3a*R*,6a*S*)-*cis*-3-hydroxy-octahydrocyclopenta[*b*]pyrrole-2-carboxylic acid (49);

(b) *cis*P4H reactions yield (2*S*,3a*S*,4*R*,6a*S*)-*cis*-4-hydroxy-octahydrocyclopenta[*b*]pyrrole-2-carboxylic acid (48);

**(c)** *trans*P4H reactions yield a single hydroxylation product (**50**) [proposed to be (2*S*,3a*S*,4*S*,6a*S*)-4-hydroxy-octahydropenta[*b*]pyrrole-2-carboxylic acid].

.


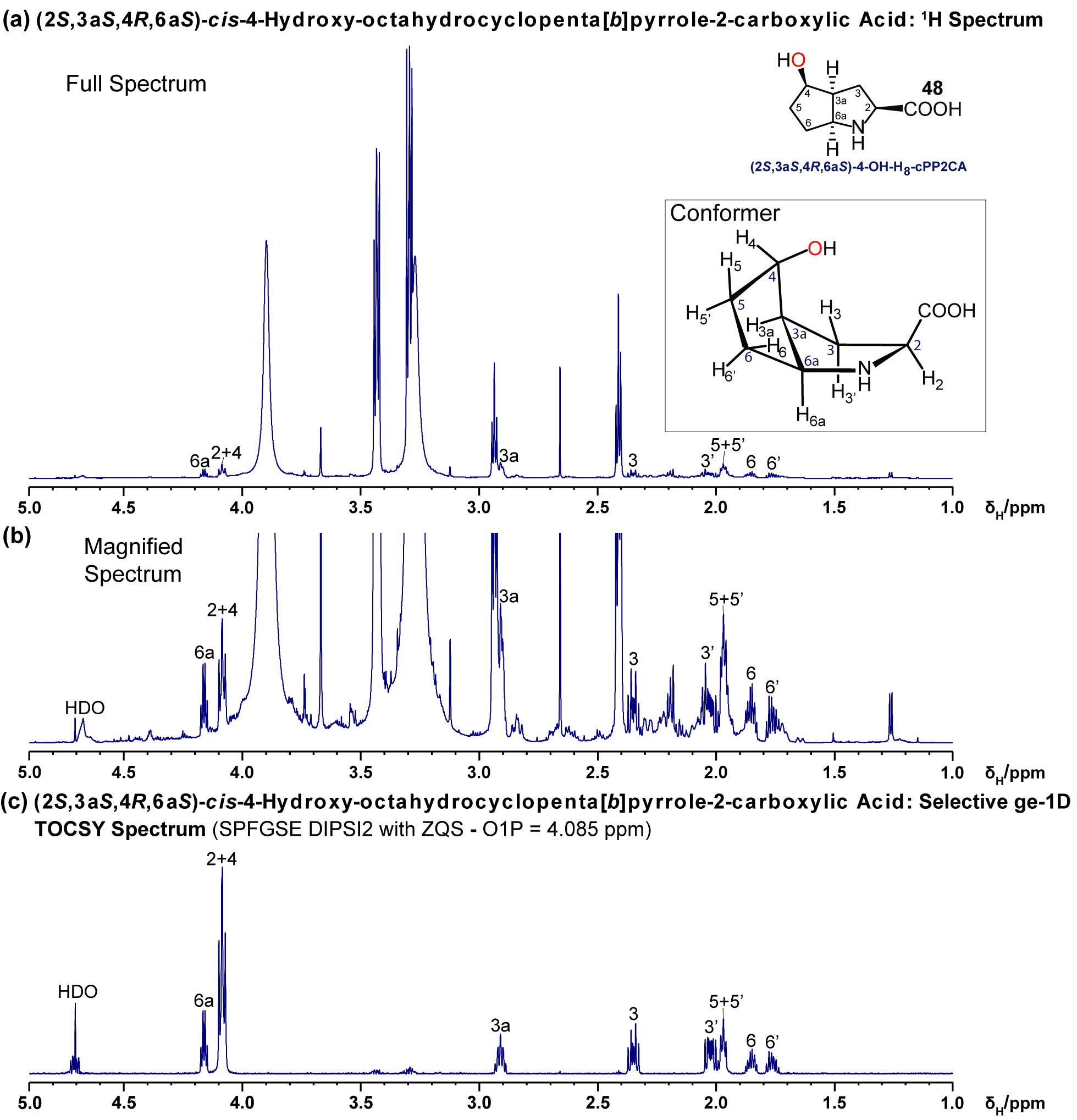


Fig. S78. NMR analyses of the (2*S*,3a*S*,4*R*,6a*S*)-*cis*-4-hydroxy-octahydrocyclopenta[*b*]pyrrole-2-carboxylic acid (48) hydroxylation product from *cis*P3H reactions using (2*S*,3a*S*,6a*S*)-octahydrocyclopenta[*b*]pyrrole-2-carboxylic acid (47): (a) and (b) ^1^H-NMR spectrum (‘zgpr’ pulse sequence) and (c) selective 1D ge-TOCSY (‘spfgsedipsi2zs’ pulse sequence). Chemical shifts are referenced to solvent (HDO) (4.701 ppm).

Assignments

^1^H NMR (700 MHz, D_2_O) δ = 4.16 (ddd, *J* = 6.2, 5.8, 5.7 Hz, 1H), 4.10 (dd, *J* = 10.3, 8.7, 8.5 Hz, 1H), 4.08 (ddd, *J* = 8.3, 3.7, 3.4 Hz, 1H), 2.91 (dddd, *J* = 8.3, 7.8, 7.3, 5.8 Hz, 1H), 2.35 (ddd, *J* = 13.8, 8.7, 7.8 Hz, 1H), 2.02 (ddd, *J* = 13.8, 10.3, 7.3 Hz, 1H), 1.98 (dddd, *J* = 15.5, 7.7, 5.6, 3.4 Hz, 1H), 1.97 (dddd, *J* =15.5, 7.6, 3.7, Hz, 1H), 1.85 (dddd, *J* = 13.3, 7.7, 5.7, 5.6 Hz, 1H), 1.76 (dddd, *J* = 15.0, 13.3, 7.7, 7.6, 6.2 Hz, 1H).


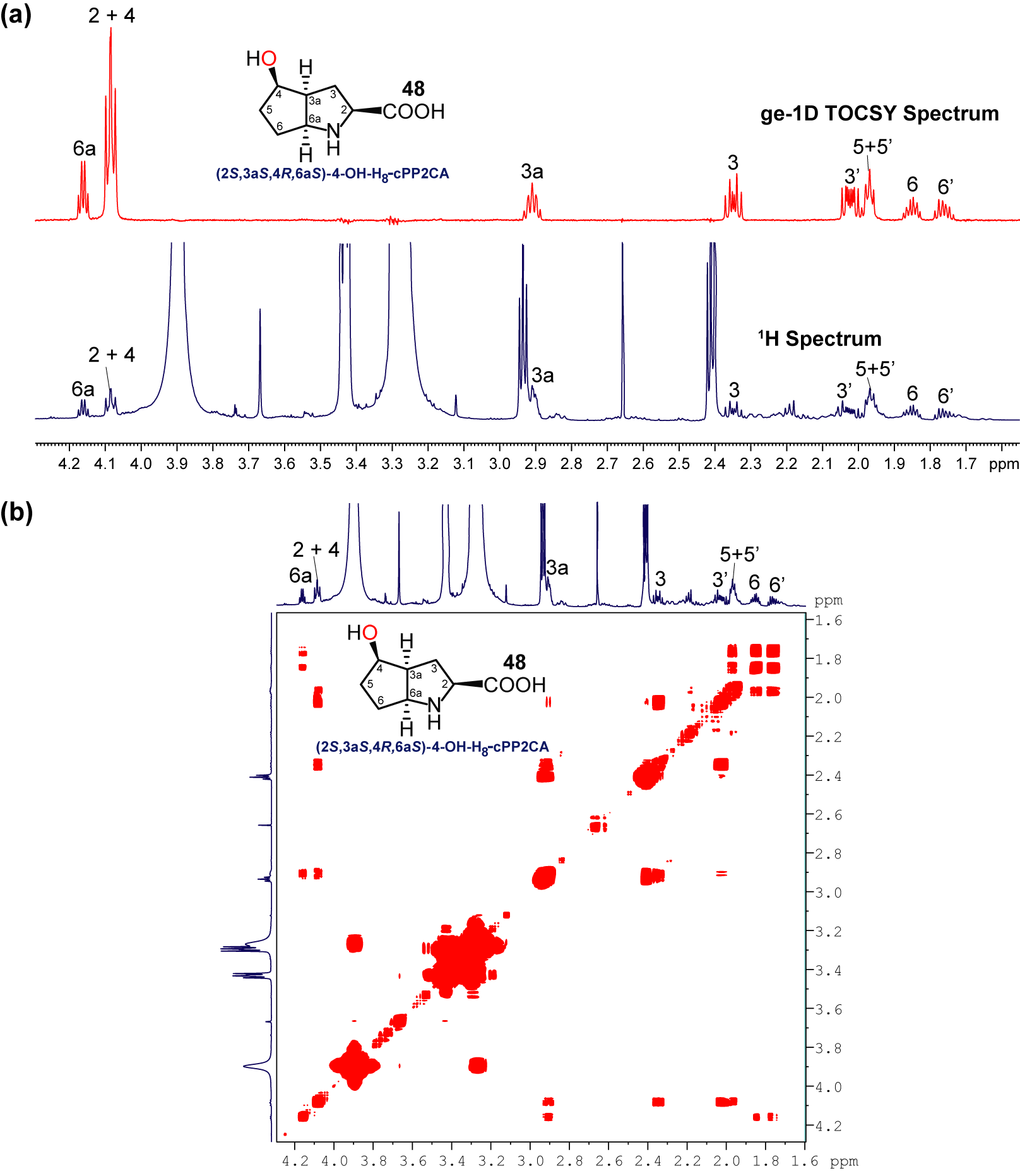


**Fig. S79.** **(a)** ge-TOCSY-NMR and ^1^H-NMR analyses and **(b)** COSY-NMR analysis of the hydroxylation product from the *cis*P3H reaction with (2*S*,3a*S*,6a*S*)-octahydrocyclopenta[*b*]pyrrole-2-carboxylic acid (**47**)**:**  NMR assignments confirmed that the product is (2*S*,3a*S*,4*R*,6a*S*)-*cis*-4-hydroxy-octahydrocyclopenta[*b*]pyrrole-2-carboxylic acid (**48**). Chemical shifts are referenced to solvent (HDO) (4.701 ppm).


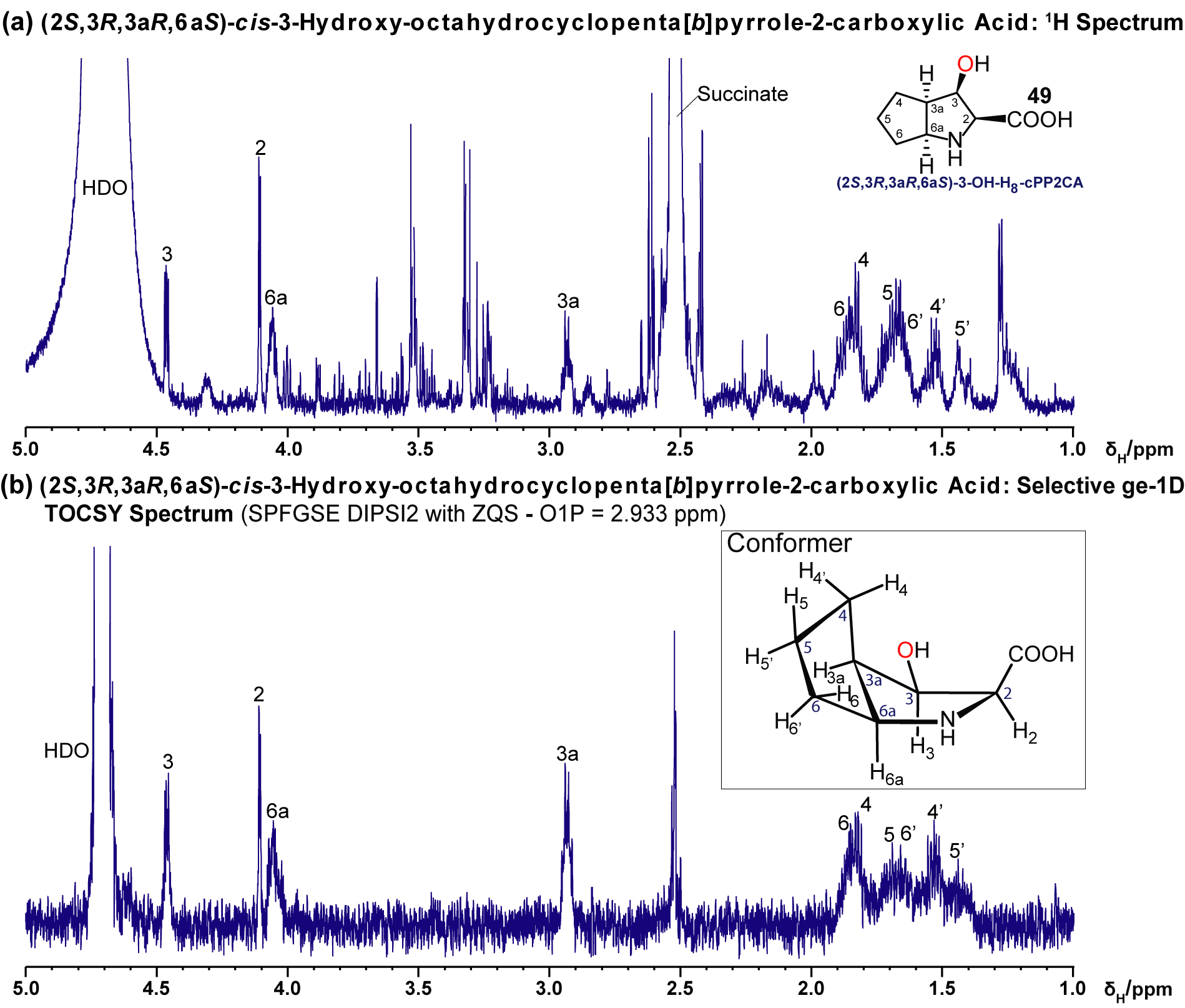


Fig. S80. NMR analyses of the (2*S*,3*R*,3a*R*,6a*S*)-*cis*-3-hydroxy-octahydrocyclopenta[*b*]pyrrole-2-carboxylic acid (49) hydroxylation product from *cis*P3H reactions using (2*S*,3a*S*,6a*S*)-octahydrocyclopenta[*b*]pyrrole-2-carboxylic acid (47): (a) ^1^H-NMR spectrum (‘zgpr’ pulse sequence) and (b) selective 1D ge-TOCSY (‘spfgsedipsi2zs’ pulse sequence). Chemical shifts are referenced to solvent (HDO) (4.701 ppm).

**Assignments**

^1^H NMR (700 MHz, D_2_O) δ = 4.46 (dd, *J* = 5.9, 3.8 Hz, 1H), 4.11 (d, *J* = 3.8 Hz, 1H), 4.06 (dddd, *J* = 7.0, 5.7, 3.8, 1.5 Hz, 1H), 2.93 (dddd, *J* = 5.9, 3.8, 3.3, 0.9 Hz, 1H), 1.86 [1.92-1.82] (dddd, *J* = 14.5, 7.0, 3.8, 1.3 Hz, 1H), 1.83 [1.78-1.87](dddd, *J* = 14.2, 7.8, 3.3, 1.5 Hz, 1H), 1.70 [1.75 – 1.64] (ddddd, *J* = 14.8, 7.8, 7.4, 1.3 Hz, 1H), 1.66 [1.61-1.71] (dddd, *J* = 14.5, 6.1, 5.7, 1.5 Hz, 1H), 1.53 [1.58 – 1.48] (dddd, *J* = 14.2, 7.4, 5.6, 1.5, 0.9 Hz, 1H), 1.43 [1.47-1.38] (ddddd, *J* = 14.8, 6.1, 5.6, 3.8, 1.5 Hz, 1H) .

**
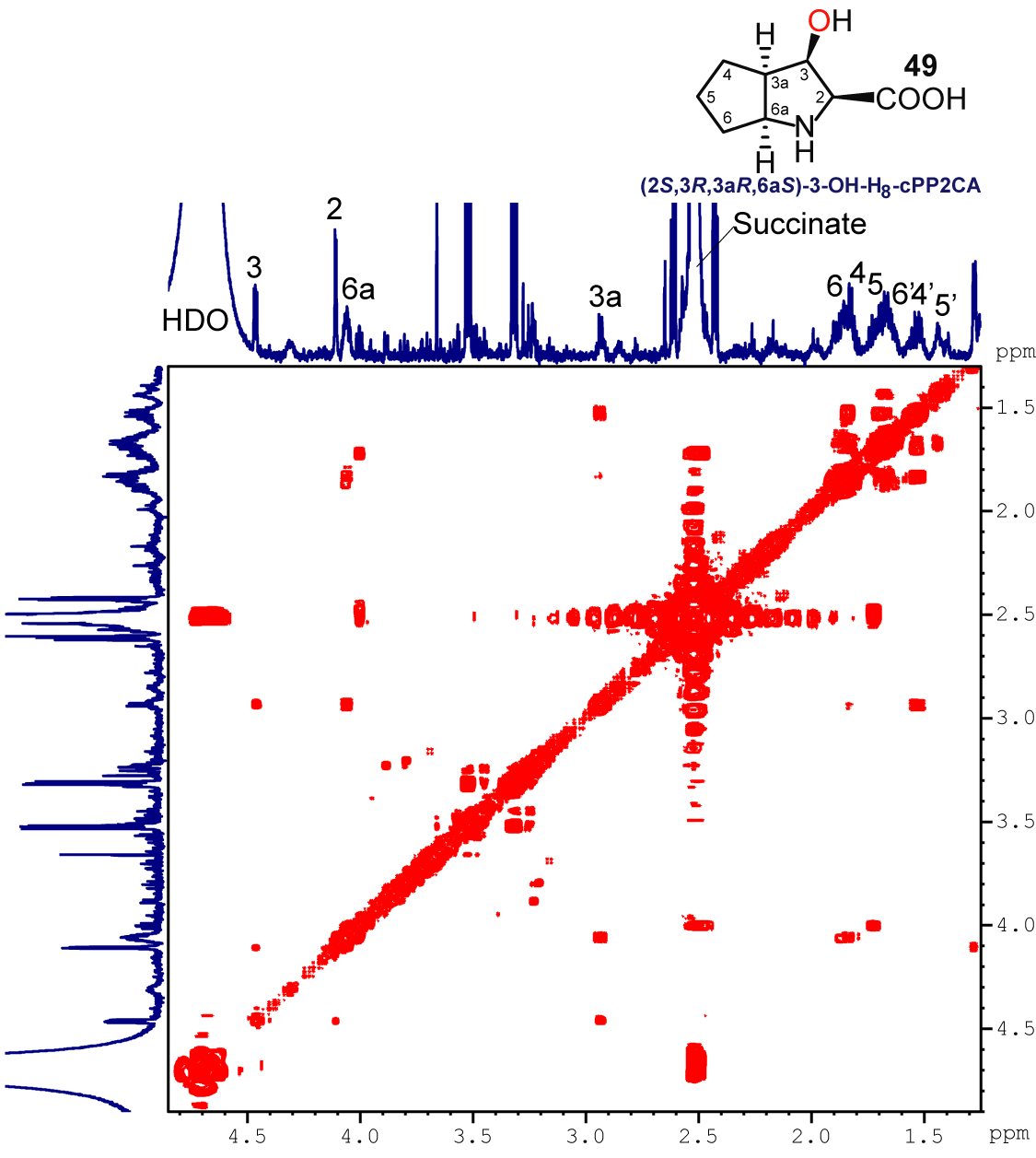
**

**Fig. S81.** 2D-NMR analyses of the (2*S*,3*R*,3a*R*,6a*S*)-*cis*-3-hydroxy-octahydrocyclopenta[*b*]pyrrole-2-carboxylic acid (**49**) hydroxylation product from *cis*P3H reactions using (2*S*,3a*S*,6a*S*)-octahydrocyclopenta[*b*]pyrrole-2-carboxylic acid (**47**): ^1^H-^1^H COSY spectrum (‘cosygpprf2qf’ pulse sequence). Chemical shifts are referenced to solvent (HDO) (4.701 ppm).

1. *(2S,3aR,6aR)-Octahydrocyclopenta[b]pyrrole-2-carboxylic Acid (****51****)*

Scheme S16. Proline hydroxylase reactions using (2*S*,3a*R*,6a*R*)-octahydrocyclopenta[*b*]pyrrole-2-carboxylic acid (51):

(a) *cis*P3H catalyses production of (2*S*,3*R*,3a*S*,6a*R*)-3-hydroxy-octahydropenta[*b*]pyrrole-2-carboxylic acid (53) and another hydroxylation product, which is (2*S*,3a*R*,4*R*,6a*R*)-4-hydroxy-octahydropenta[*b*]pyrrole-2-carboxylic acid (52);

(b) *trans*P4H catalyses production of a single hydroxylation product, which could be (2*S*,3a*R*,4S,6a*R*)-4-hydroxy-octahydropenta[*b*]pyrrole-2-carboxylic acid (54).


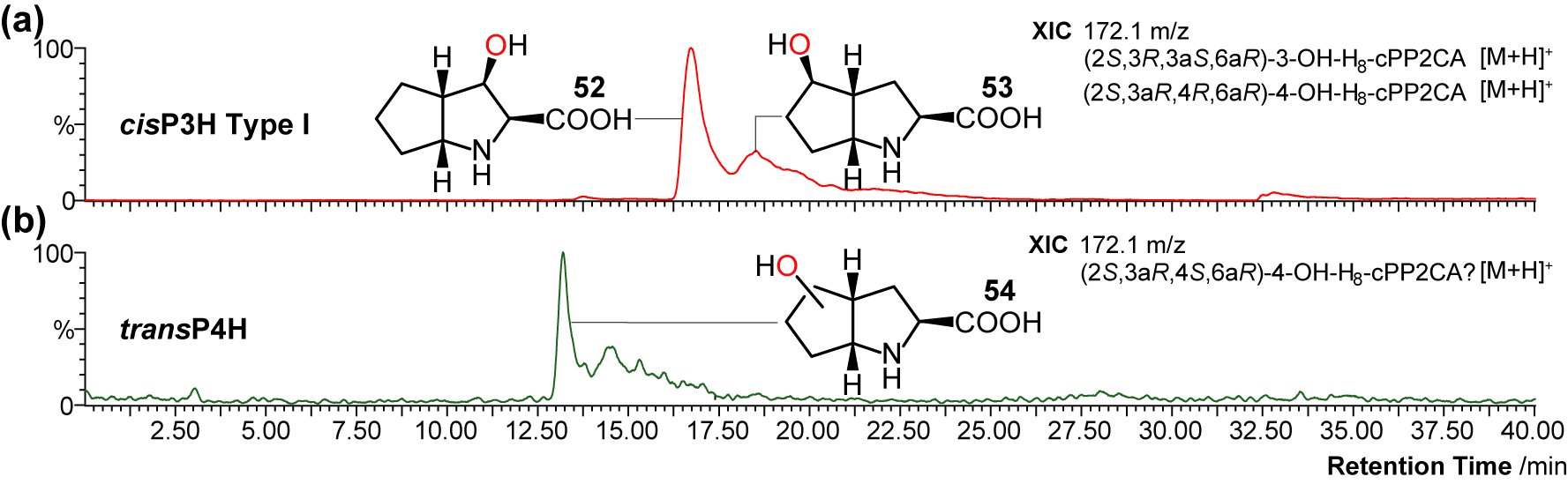


Fig. S82. Extracted-ion count (XIC) LC/MS chromatograms for the proline hydroxylase reactions using a (2*S*,3a*R*,6a*R*)-octahydrocyclopenta[*b*]pyrrole-2-carboxylic acid (51) substrate:

(a) *cis*P3H reactions yield (2*S*,3*R*,3a*S*,6a*R*)-3-hydroxy-octahydropenta[*b*]pyrrole-2-carboxylic acid (53) and another hydroxylation product, which is (2*S*,3a*R*,4*R*,6a*R*)-4-hydroxy-octahydropenta[*b*]pyrrole-2-carboxylic acid (52);

(b) *trans*P4H reactions yield a single hydroxylation product (54) [proposed to be (2*S*,3a*R*,4S,6a*R*)-4-hydroxy-octahydropenta[*b*]pyrrole-2-carboxylic acid].

Stereochemical assignments were made by NMR.


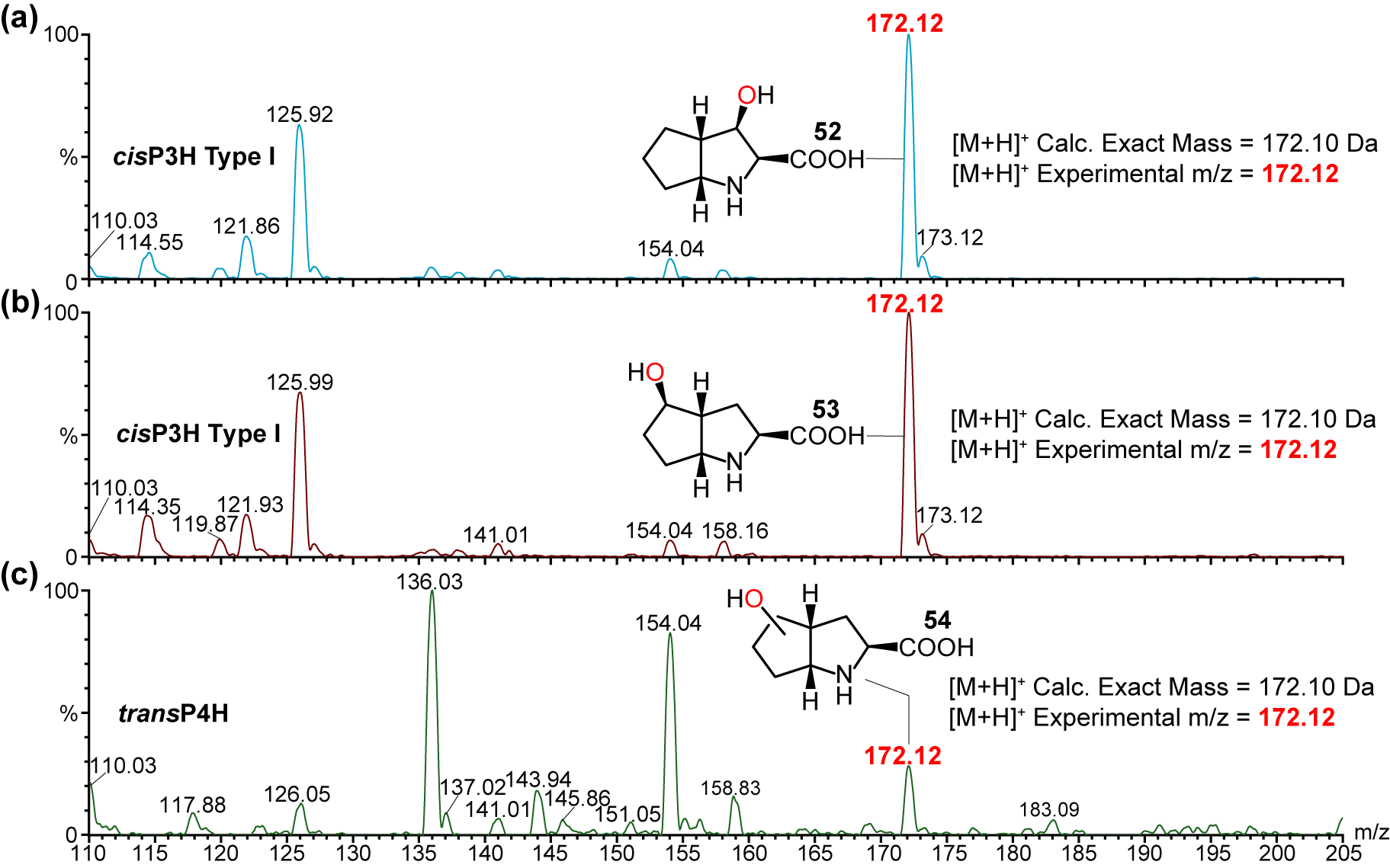


Fig. S83. LC/MS spectra of proline hydroxylase reactions using (2*S*,3a*R*,6a*R*)-octahydrocyclopenta[*b*]pyrrole-2-carboxylic acid (51):

(a) and (b) *cis*P3H reactions contain (2*S*,3*R*,3a*S*,6a*R*)-3-hydroxy-octahydropenta[*b*]pyrrole-2-carboxylic acid (52) and (b) another hydroxylation product, which may be (2*S*,3a*R*,4*R*,6a*R*)-4-hydroxy-octahydropenta[*b*]pyrrole-2-carboxylic acid (53);

(c) *trans*P4H reactions contain a single hydroxylation product (54) [proposed to be (2*S*,3a*R*,4S,6a*R*)-4-hydroxy-octahydropenta[*b*]pyrrole-2-carboxylic acid].

Stereochemical assignments were made by NMR.


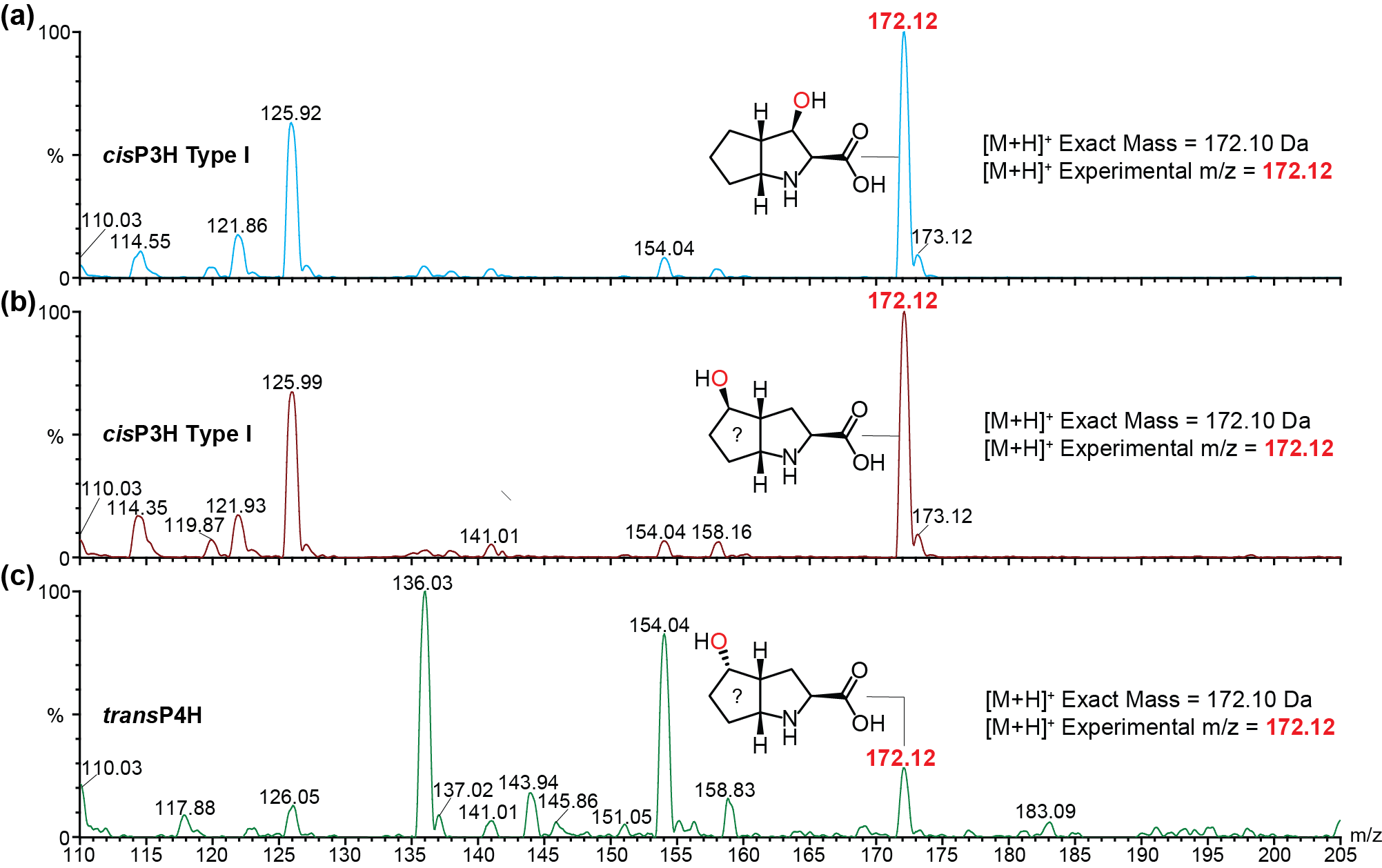


Fig. S45 Mass spectral analyses (LC/MS) of proline hydroxylase reactions using a (2*S*,3a*R*,6a*R*)-octahydrocyclopenta[*b*]pyrrole-2-carboxylic acid (66) substrate:

(a) *cis*P3H reactions contain (2*S*,3*R*,3a*S*,6a*R*)-3-hydroxy-L-octahydropenta[*b*]pyrrole-2-carboxylic acid (67) and (b) another hydroxylation product, which may be (2*S*,3a*R*,4*R*,6a*R*)-4-hydroxy-L-octahydropenta[*b*]pyrrole-2-carboxylic acid (68);

(b) *trans*P4H reactions contain Error! Reference source not found.(2*S*,3a*S*,4*R*,6a*S*)-*cis*-4-hydroxy-L-octahydrocyclopenta[*b*]pyrrole-2-carboxylic acid (Error! Reference source not found.).


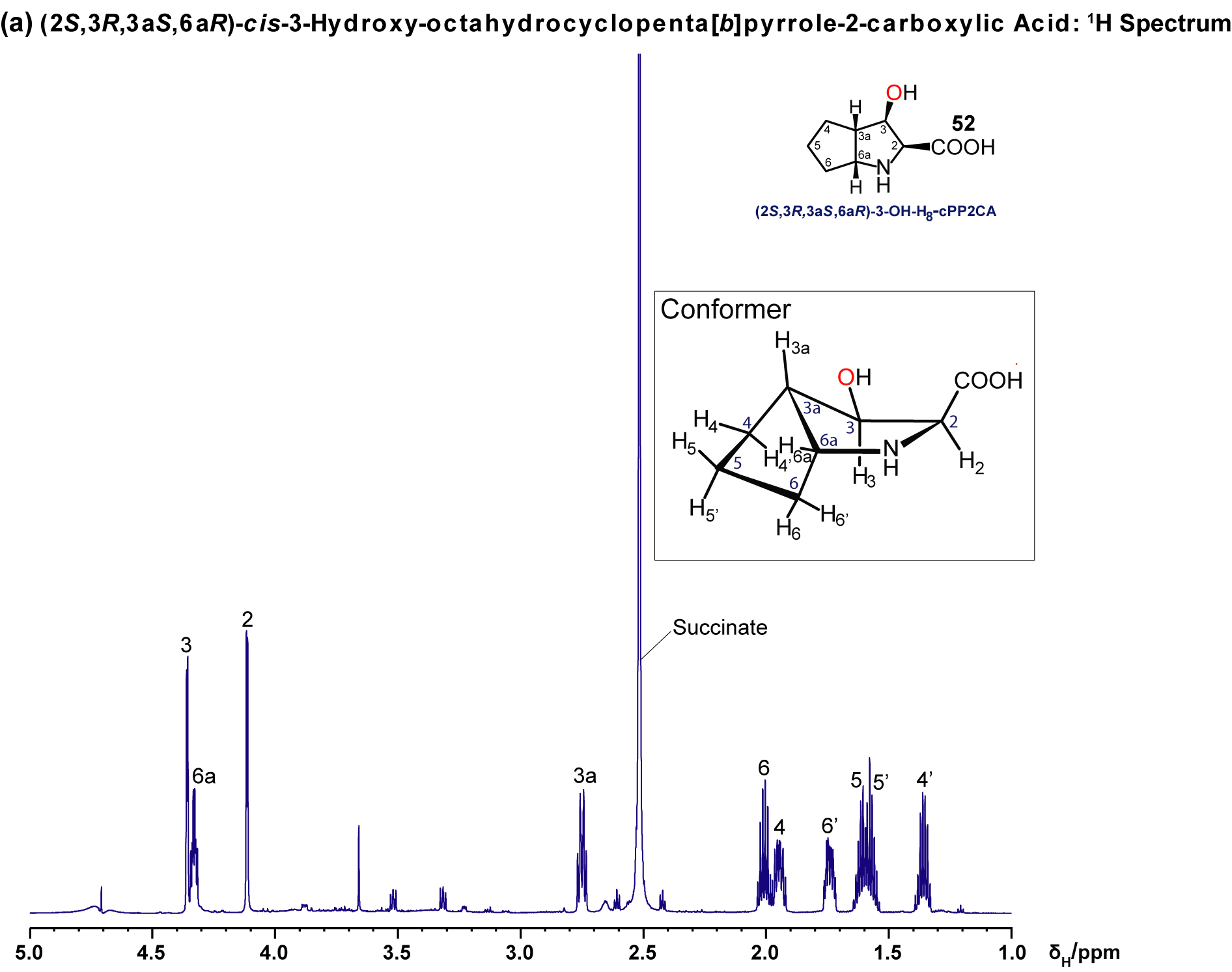


**Fig. S84.** NMR analyses of the (2*S*,3*R*,3a*S*,6a*R*)-3-hydroxy-octahydropenta[*b*]pyrrole-2-carboxylic acid (**52**) hydroxylation product from *cis*P3H reactions using (2*S*,3a*R*,6a*R*)-octahydrocyclopenta[*b*]pyrrole-2-carboxylic acid (**51**): **(a)** ^1^H-NMR spectrum (‘zgpr’ pulse sequence). Chemical shifts are referenced to solvent (HDO) (4.701 ppm).

**Assignments**

^1^H NMR (700 MHz, D2O) δ = 4.36 (dd, J = 3.5, 0.5 Hz, 1H), 4.33 (ddd, J = 7.3, 7.2, 4.2 Hz, 1H), 4.11 (d, J = 3.5 Hz, 1H), 2.75 (dddd, J = 7.3, 7.2, 2.9, 0.5 Hz, 1H), 2.01 (dddd, J = 14.3, 7.3, 7.1, 7.0 Hz, 1H), 1.95 (dddd, J = 13.7, 7.0, 6.9, 2.9 Hz, 1H), 1.74 (dddd, J = 14.3, 7.2, 6.7, 4.2 Hz, 1H), 1.60 [1.64 – 1.58] (ddddd, J = 13.4, 7.0, 6.9, 6.7, 6.7 Hz, 1H), 1.57 [1.61 – 1.53] (ddddd, J = 13.4, 7.2, 7.1, 7.0, 7.0 Hz, 1H), 1.36 [1.40 – 1.32] (dddd, J = 13.7, 7.3, 7.0, 6.7 Hz, 1H).


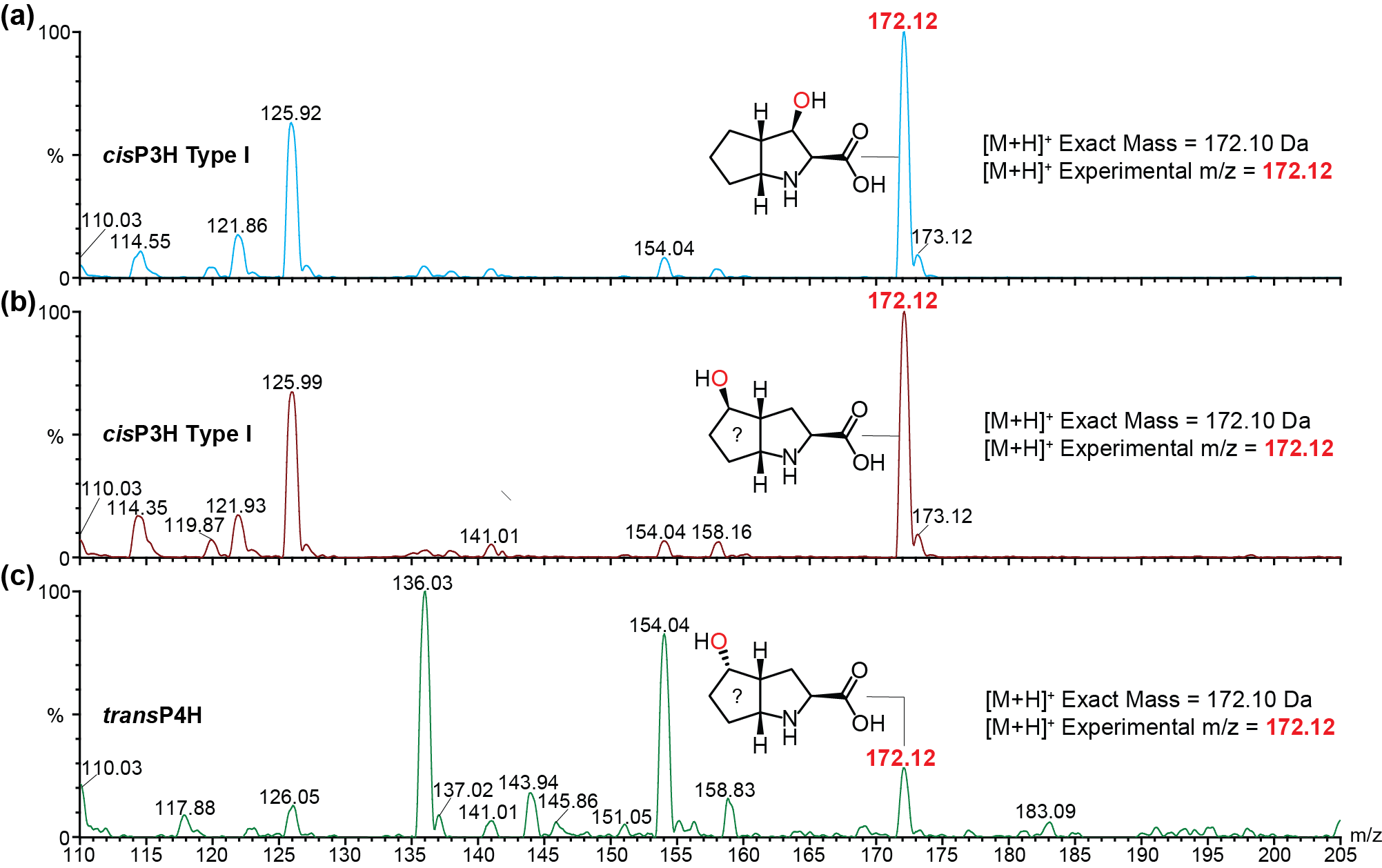


Figure S46 Mass spectral analyses (LC/MS) of proline hydroxylase reactions using a (2*S*,3a*R*,6a*R*)-octahydrocyclopenta[*b*]pyrrole-2-carboxylic acid (69) substrate:

(a) *cis*P3H reactions contain (2*S*,3*R*,3a*S*,6a*R*)-3-hydroxy-L-octahydropenta[*b*]pyrrole-2-carboxylic acid (70) and (b) another hydroxylation product, which may be (2*S*,3a*R*,4*R*,6a*R*)-4-hydroxy-L-octahydropenta[*b*]pyrrole-2-carboxylic acid (71);

(b) *trans*P4H reactions contain Error! Reference source not found.(2*S*,3a*S*,4*R*,6a*S*)-*cis*-4-hydroxy-L-octahydrocyclopenta[*b*]pyrrole-2-carboxylic acid (Error! Reference source not found.).


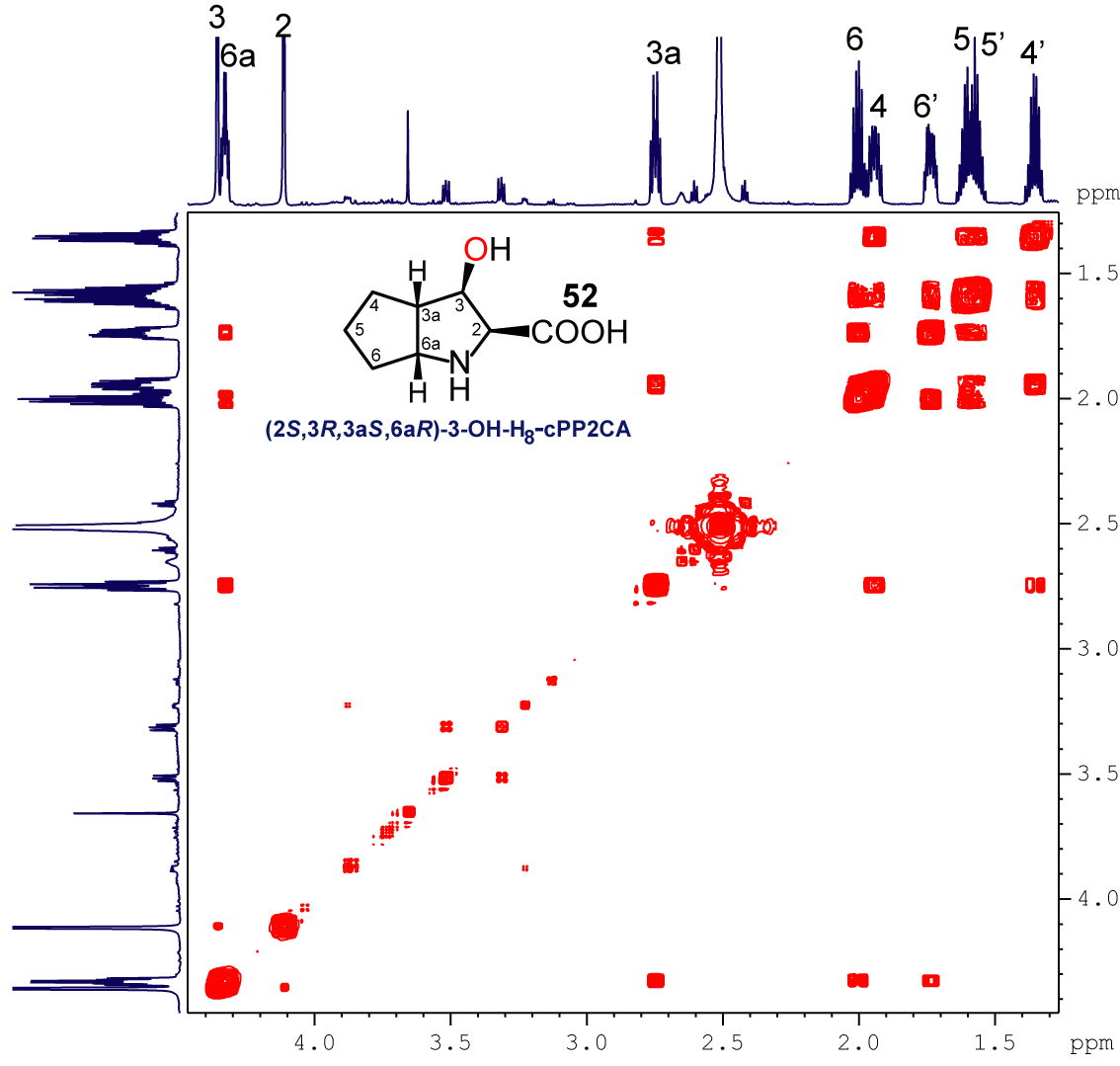


**Fig. S85.** **(a)** COSY-NMR analysis and **(b)** NOESY-NMR analysis of the hydroxylation product from the *cis*P3H reaction with (2*S*,3a*R*,6a*R*)-octahydrocyclopenta[*b*]pyrrole-2-carboxylic acid (**51**)**:**  NMR assignments confirmed that the product is (2*S*,3*R*,3a*S*,6a*R*)-3-hydroxy-octahydropenta[*b*]pyrrole-2-carboxylic acid (**52**). Chemical shifts are referenced to solvent (HDO) (4.701 ppm).

**(b)**

**(a)**

1. *(2S,3aS,7aS)-Octahydro-1H-indole-2-carboxylic Acid (****55****)*

Scheme S17 Proline hydroxylase reactions using (2*S*,3a*S*,7a*S*)-octahydro-1H-indole-2-carboxylic acid [(2*S*,3a*S*,7a*S*)-H_8_-L-I2CA] (55): *cis*P3H reactions yield (2*S*,3a*S*,4*R*,7a*S*)-4-hydroxy-octahydro-1*H*-indole-2-carboxylic acid (56).

Fig. S86. Extracted-ion count (XIC) LC/MS chromatograms for the proline hydroxylase reactions using (2*S*,3a*S*,7a*S*)-octahydro-1H-indole-2-carboxylic acid [(2*S*,3a*S*,7a*S*)-H_8_-L-I2CA] (55): *cis*P3H reactions produce (2*S*,3a*S*,4*R*,7a*S*)-4-hydroxy-octahydro-1*H*-indole-2-carboxylic acid (56).

Fig. S87. LC/MS spectra of proline hydroxylase reactions using (2*S*,3a*S*,7a*S*)-octahydro-1H-indole-2-carboxylic acid [(2*S*,3a*S*,7a*S*)-H_8_-L-I2CA] (55): *cis*P3H reactions yield (2*S*,3a*S*,4*R*,7a*S*)-4-hydroxy-octahydro-1*H*-indole-2-carboxylic acid (56).

Stereochemical assignments were made by NMR.

Fig. S88. NMR analyses of the (2*S*,3a*S*,7a*S*)-octahydro-1H-indole-2-carboxylic acid [(2*S*,3a*S*,4*R*,7a*S*)-4-OH-H_8_-L-I2CA] (56) hydroxylation product from *cis*P3H reactions using (2*S*,3a*S*,7a*S*)-octahydro-1*H*-indole-2-carboxylic acid (55): (a) ^1^H-NMR spectrum (‘zgpr’ pulse sequence) and (b) selective 1D ge-TOCSY (‘spfgsedipsi2zs’ pulse sequence). Chemical shifts are referenced to solvent (HDO) (4.701 ppm).

**Assignments**

^1^H NMR (700 MHz, D_2_O) δ = 4.16 (dd, *J* = 9.5, 9.3 Hz, 1H), 3.93 (ddd, *J* = 10.4, 5.1, 4.6 Hz, 1H), 3.76 (ddd, *J* = 10.6, 5.5, 3.9 Hz, 1H), 2.64 (dddd, *J* = 6.3, 5.5, 5.1, 3.5 Hz, 1H), 2.35 [2.38 – 2.32] (ddd, *J* = 13.1, 9.5, 6.3 Hz, 1H, 2.04 [2.07 – 2.01] (ddd, *J* = 13.1, 9.3, 3.5 Hz, 1H), 1.80 [1.83 – 1.77] (dddd, *J* = 13.5, 4.7, 4.6, 3.9 Hz, 1H), 1.74 [1.77 – 1.72] (ddddd, *J* = 14.0, 4.7, 4.2, 3.6, 3.3 Hz, 1H), 1.65 [1.67 – 1.62] (dddd, *J* = 13.2, 4.6, 4.2, 3.4 Hz, 1H), 1.41 [1.45 – 1.37] (dddd, *J* = 13.2, 10.4, 9.3, 3.3 Hz, 1H), 1.40 [1.41 – 1.34] (dddd, *J* = 13.5, 10.6, 7.6, 3.6 Hz, 1H), 1.24 [1.28 – 1.19] (ddddd, *J* = 14.0, 9.3, 7.6, 4.6, 3.4 Hz, 1H).

**Fig. S89.** **(a)** COSY-NMR analysis and **(b)** NOESY-NMR analysis of the hydroxylation product (**56**) from the *cis*P3H reaction with (2*S*,3a*S*,7a*S*)-octahydro-1H-indole-2-carboxylic acid [(2*S*,3a*S*,7a*S*)-H_8_-L-I2CA] (**55**) (aligned with ge-1D TOCSY spectra)**:** NMR assignments assigned the product as (2*S*,3a*S*,4*R*,7a*S*)-4-hydroxyoctahydro-1*H*-indole-2-carboxylic acid (**56**). Chemical shifts are referenced to solvent (HDO) (4.701 ppm).

**(b)**

**(a)**

1. *Decanhydroisoquinoline-3-carboxylic Acid (****57****)*

Scheme S18. Proline hydroxylase reactions using decanhydroisoquinoline-3-carboxylic acid (57):

(a) *cis*P3H catalyses production of a single hydroxylation product (58);

(b) *trans*P4H catalyses production of a single hydroxylation product (59).

Fig. S90. Extracted-ion count (XIC) LC/MS chromatograms for the proline hydroxylase reactions using a decanhydroisoquinoline-3-carboxylic acid (57) substrate:

(a) *cis*P3H reactions yield a single hydroxylation product (58);

(b) *trans*P4H reactions yield a single hydroxylation product (59).

Fig. S91 LC/MS spectra of proline hydroxylase reactions using decanhydroisoquinoline-3-carboxylic acid (57):

(a) *cis*P3H reactions yield a single hydroxylation product (58);

(b) *trans*P4H reactions yield a single hydroxylation product (59).

Stereochemical assignments were made by NMR.

## *Unsaturated Substrate Analogues*

1. *(2S)-3,4-Dehydro-L-proline (****60****)*

Scheme S19. Proline hydroxylase reactions using (2*S*)-3,4-dehydro-L-proline (60):

(a) *cis*P3H catalyses production of (2*S*,3*R*,4*R*)-*cis*-3,4-epoxy-L-proline (61);

(b) *cis*P4H catalyses production of (2*S*,3*R*,4*R*)-*cis*-3,4-epoxy-L-proline (61);

(c) *trans*P4H catalyses production of (2*S*,3*R*,4*S*)-*trans*-3,4-epoxy-L-proline (62).

Fig. S92. Extracted-ion count (XIC) LC/MS chromatograms for the proline hydroxylase reactions using (2*S*)-3,4-dehydro-L-proline (60):

(a) *cis*P3H reactions yield (2*S*,3*R*,4*R*)-*cis*-3,4-epoxy-L-proline (61);

(b) *cis*P4H reactions yield (2*S*,3*R*,4*R*)-*cis*-3,4-epoxy-L-proline (61);

(c) *trans*P4H reactions yield (2*S*,3*R*,4*S*)-*trans*-3,4-epoxy-L-proline (62).

Stereochemical assignments were made by NMR.

Fig. S93. LC/MS spectra of proline hydroxylase reactions using (2*S*)-3,4-dehydro-L-proline (60):

(a) *cis*P3H reactions yield (2*S*,3*R*,4*R*)-*cis*-3,4-epoxy-L-proline (61);

(b) *cis*P4H reactions yield (2*S*,3*R*,4*R*)-*cis*-3,4-epoxy-L-proline (61);

(c) *trans*P4H reactions yield (2*S*,3*R*,4*S*)-*trans*-3,4-epoxy-L-proline (62).

The stereochemical assignments of the (2*S*,3*R*,4*R*)-*cis*-3,4-epoxy-L-proline (**61**) and (2*S*,3*R*,4*S*)-*trans*-3,4-epoxy-L-proline (**62**) products have been determined using NMR in previous reports [[9](#_ENREF_9),[11-13](#_ENREF_11)].

1. *(2S)-4,5-Dehydro-L-pipecolic Acid (****63****)*

Scheme S20. Proline hydroxylase reactions using (2*S*)-4,5-dehydro-L-pipecolic acid (63): *cis*P3H catalyses the production of epoxy-L-pipecolic acid (2*S*,4*S*,5*R*)-*cis*-4,5-epoxy-L-pipecolic acid (64)] or a hydroxylation product (2*S*,3*R*)-*cis*-3-hydroxy- L-pipecolic acid (65)].

Fig. S94. Extracted-ion count (XIC) LC/MS chromatograms for the proline hydroxylase reactions using (2*S*)-4,5-dehydro-L-pipecolic acid (63): *cis*P3H reactions yield an epoxy-L-pipecolic acid [proposed to be (2*S*,4*S*,5*R*)-*cis*-4,5-epoxy-L-pipecolic acid (64)] or a hydroxylation product [proposed to be (2*S*,3*R*)-*cis*-3-hydroxy-L-pipecolic acid (65)].

Fig. S95. LC/MS spectra of proline hydroxylase reactions using (2*S*)-4,5-dehydro-L-pipecolic acid (63): *cis*P3H reactions yield an epoxy-L-pipecolic acid (2*S*,4*S*, 5*R*)-*cis*-4,5-epoxy-L-pipecolic acid (64)] or a hydroxylation product (2*S*,3*R*)-*cis*-3-hydroxy- L-pipecolic acid (65)].

# References

[1] J. Sambrook, E.F. Fritsch, T. Maniatis, Molecular cloning: A laboratory manual, 2nd edition, Cold Spring Harbor Laboratory Press, Cold Spring Harbor, New York, (1989).

[2] E.T. Batchelar, R.B. Hamed, C. Ducho, T.D. Claridge, M.J. Edelmann, B. Kessler, C.J. Schofield, Thioester hydrolysis and C-C bond formation by carboxymethylproline synthase from the crotonase superfamily, Angewandte Chemie Intl. Ed. 47(48) (2008) 9322-5. doi: 10.1002/anie.200803906

[3] R.B. Hamed, J. Mecinović, C. Ducho, T.D. Claridge, C.J. Schofield, Carboxymethylproline Synthase Catalysed Syntheses of Functionalised *N*-Heterocycles, Chem. Commun. 46(9) (2010) 1413-1415. doi: 10.1039/b924519g

[4] R.B. Hamed, J.R. Gomez-Castellanos, A. Thalhammer, D. Harding, C. Ducho, T.D. Claridge, C.J. Schofield, Stereoselective C-C Bond Formation Catalysed by Engineered Carboxymethylproline Synthases, Nat. Chem. 3(5) (2011) 365-371. doi: 10.1038/nchem.1011

[5] R.B. Hamed, L. Henry, J.R. Gomez-Castellanos, J. Mecinovic, C. Ducho, J.L. Sorensen, T.D. Claridge, C.J. Schofield, Crotonase Catalysis Enables Flexible Production of Functionalized Prolines and Carbapenams, J. Am. Chem. Soc. 134(1) (2012) 471-479. doi: 10.1021/ja208318d

[6] R.B. Hamed, L. Henry, J.R. Gomez-Castellanos, A. Asghar, J. Brem, T.D. Claridge, C.J. Schofield, Stereoselective Preparation of Lipidated Carboxymethyl-proline/pipecolic Acid Derivatives *via* Coupling of Engineered Crotonases with an Alkylmalonyl-CoA Synthetase, Org. Biomol. Chem. 11(47) (2013) 8191-8196. doi: 10.1039/c3ob41525b

[7] H. Mori, T. Shibasaki, Y. Uozaki, K. Ochiai, A. Ozaki, Detection of Novel Proline 3-Hydroxylase Activities in *Streptomyces* and *Bacillus spp.* by Regio- and Stereospecific Hydroxylation of L-Proline., Appl. Environ. Microbiol. 62(6) (1996) 1903-1907

[8] H. Mori, T. Shibasaki, K. Yano, A. Ozaki, Purification and Cloning of a Proline 3-Hydroxylase, a Novel Enzyme which Hydroxylates Free L-Proline to *cis*-3-Hydroxy-L-Proline., J. Bacteriol. 179(18) (1997) 5677-5683. doi: 10.1128/jb.179.18.5677-5683.1997

[9] T. Shibasaki, H. Mori, A. Ozaki, Cloning of an Isozyme of Proline 3-Hydroxylase and its Purification from Recombinant *Escherichia coli.*, Biotechnol. Lett. 22(24) (2000) 1967-1973. doi: 10.1023/A:1026792430742

[10] F.B. Küllchen, Studies on Proline 3-Hydroxylase., DPhil Thesis, The Dyson Perrins Laboratory, Department of Chemistry, University of Oxford, 2002.

[11] T. Shibasaki, W. Sakurai, A. Hasegawa, Y. Uosaki, H. Mori, M. Yoshida, A. Ozaki, Substrate Selectivities of Proline Hydroxylases, Tetrahedron Lett. 40(28) (1999) 5227-5230. doi: 10.1016/S0040-4039(99)00944-2

[12] J.E. Baldwin, R.A. Field, C.C. Lawrence, V. Lee, J.K. Robinson, C.J. Schofield, Substrate-Specificity of Proline 4-Hydroxylase: Chemical and Enzymatic-Synthesis of 2*S*,3*R*,4*S*-Epoxyproline, Tetrahedron Lett. 35(26) (1994) 4649-4652. doi: 10.1016/S0040-4039(00)60753-0

[13] K. Koketsu, Y. Shomura, K. Moriwaki, M. Hayashi, S. Mitsuhashi, R. Hara, K. Kino, Y. Higuchi, Refined Regio- and Stereoselective Hydroxylation of L-Pipecolic Acid by Protein Engineering of L-Proline *cis*-4-Hydroxylase Based on the X-Ray Crystal Structure., ACS Synth. Biol. 4(4) (2015) 383-392. doi: 10.1021/sb500247a
